# Accelerating the insertion reactions of (NHC)Cu–H *via* remote ligand functionalization†

**DOI:** 10.1039/d1sc01911b

**Published:** 2021-07-29

**Authors:** Amy L. Speelman, Ba L. Tran, Jeremy D. Erickson, Monica Vasiliu, David A. Dixon, R. Morris Bullock

**Affiliations:** Institute for Integrated Catalysis, Pacific Northwest National Laboratory Richland WA 99352 USA morris.bullock@pnnl.gov ba.tran@pnnl.gov; Department of Chemistry and Biochemistry, University of Alabama Tuscaloosa AL 35487 USA

## Abstract

Most ligand designs for reactions catalyzed by (NHC)Cu–H (NHC = N-heterocyclic carbene ligand) have focused on introducing steric bulk near the Cu center. Here, we evaluate the effect of remote ligand modification in a series of [(NHC)CuH]_2_ in which the *para* substituent (R) on the *N*-aryl groups of the NHC is Me, Et, ^*t*^Bu, OMe or Cl. Although the R group is distant (6 bonds away) from the reactive Cu center, the complexes have different spectroscopic signatures. Kinetics studies of the insertion of ketone, aldimine, alkyne, and unactivated α-olefin substrates reveal that Cu–H complexes with bulky or electron-rich R groups undergo faster substrate insertion. The predominant cause of this phenomenon is destabilization of the [(NHC)CuH]_2_ dimer relative to the (NHC)Cu–H monomer, resulting in faster formation of Cu–H monomer. These findings indicate that remote functionalization of NHCs is a compelling strategy for accelerating the rate of substrate insertion with Cu–H species.

## Abstract 2

Remote modification of an N-heterocyclic carbene ligand with bulky or electron-rich groups in [(NHC)Cu(μ-H)]_2_ increases the rate of substrate insertion, which kinetics studies suggest arises from changes in the Cu–H monomer–dimer equilibrium.

## Graphical abstract

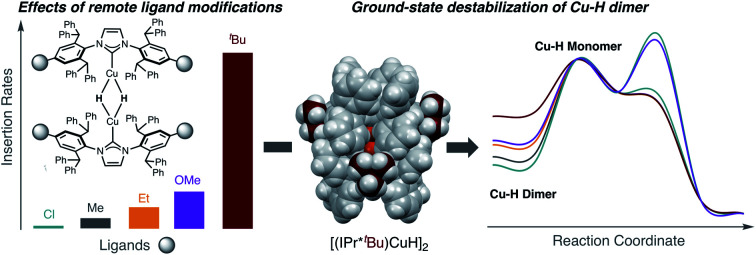

## Introduction

The insertion of π-bonds into metal hydrides is a crucial elementary step in many metal-catalyzed transformations.^1–9^ Copper hydrides are among the most poorly understood metal hydrides, despite their key role in hydrofunctionalization reactions. Insertions of π-bonds into Cu–H can determine the enantioselectivity, regioselectivity, and chemoselectivity of catalytic reactions.^10–12^ Reactions of alkynes, olefins and dienes with Cu–H under mild conditions have led to versatile approaches for upgrading feedstocks to value-added products.^13^ Although there have been impressive advances in methodology using Cu–H complexes, the instability of these species in solution has hampered mechanistic studies of substrate insertion into Cu–H bonds, which is a foundational elementary step in all proposed mechanisms for Cu–H catalyzed reactions.^10,11,14–25^ Recent investigations have circumvented some of these solution stability problems by sterically protecting the Cu–H using bulky diphosphine,^10,26,27^ cyclic alkyl amino carbene (CAAC)^28,29^ or N-heterocyclic carbene (NHC)^30–33^ ligands.

**Fig. 1:**
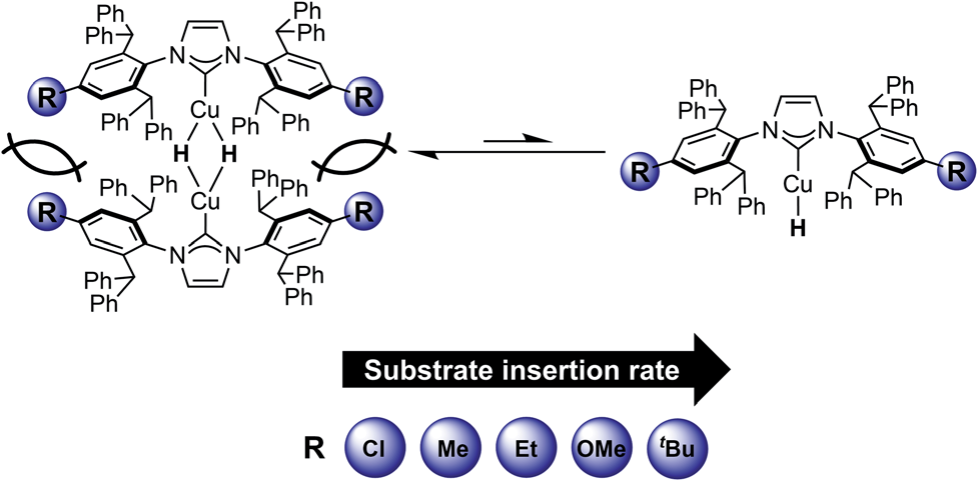
Effect of remote ligand modifications on the rate of substrate insertion with [(IPr*R)CuH]_2_ complexes studied in this work.

Cu–H complexes aggregate in solution,^11^ and the enhanced reactivity observed with bulky ligands has been suggested to result from faster formation of reactive Cu–H monomers.^26,34–36^ Providing direct evidence for this hypothesis, we recently reported kinetics studies on the insertion of carbonyl substrates with [(IPr*Me)CuH]_2_ (IPr*Me = *N*,*N*′-bis(2,6-bis(diphenylmethyl)-4-methylphenyl)imidazole-2-ylidene, Fig. 1 with R = Me) which showed that the use of bulkier NHCs both dramatically increases the rates of substrate insertion and allows insertion of less reactive substrates, such as unactivated esters and amides.^32^

Structural analysis of dimeric [(NHC)Cu(μ-H)]_2_ complexes stabilized by ring-expanded and bulky aryl NHCs demonstrates that the two NHC ligands are in close proximity.^30,32,33^ This observation suggests that substitutions on the periphery of NHC ligands could potentially influence the Cu–H monomer–dimer equilibrium, thereby enhancing reactivity. An additional advantage of remote substitution is that the added steric bulk is far from the metal, especially in the Cu–H monomer, diminishing the likelihood of inhibiting the approach of the substrate to the metal hydride. Although the impacts of remote variation of the electronic and steric properties of NHC ligands on the structure and reactivity of transition metal complexes have been reported,^37–42^ their effects on Cu–H insertion reactions have not been systematically examined. Installing steric bulk on the periphery of a ligand can impact monomer-dimer equilibria, as demonstrated by β-diketiminate Fe, Ni, and Co chloride complexes,^43^ which suggests that remote ligand modification could be effective for tuning the reactivity of Cu–H systems. Modification of NHC donor properties through installation of electron-donating or electron-withdrawing substituents could also have an impact on the Cu–H monomer–dimer equilibrium and the rate of hydride transfer, but has not been studied in detail. The IPr*R ligand platform (Fig. 1) is well-suited for examining the systematic effects of NHC properties on Cu–H reactivity because it is synthetically tunable^40,44,45^ and sufficiently bulky to stabilize copper hydrides.

Here, we report the synthesis and characterization of a series of [(IPr*R)CuH]_2_ (R = Me, Et, ^*t*^Bu, OMe, Cl) complexes (Fig. 1) in which modifications are made to the *para* position of the NHC aryl groups, six bonds away from the Cu center. Our kinetics studies demonstrate that remote modification of the NHC ligand with bulky and electron-donating groups can produce more than an order of magnitude change in rate for insertion reactions with carbonyl, aldimine, alkyne, and α-olefin substrates. Density functional theory (DFT) calculations provide additional structural, spectroscopic, and mechanistic insights. These studies of a well-defined class of [(NHC)Cu(μ-H)]_2_ complexes contribute to understanding of the factors influencing the key hydrocupration step in the functionalization of unsaturated substrates using (NHC)Cu–H, and demonstrate the ability of remote ligand modification of molecular Cu–H complexes to accelerate insertion reactions.

## Results and discussion

### Analysis of the structural and electronic properties of the IPr*R ligand family

A series of IPr*R·HCl salts (R = Me, Et, ^*t*^Bu, OMe, Cl) was readily prepared on a multigram scale by one-pot reactions of *para*-substituted 2,6-dibenzhydrylanilines with glyoxal, formaldehyde, and HCl.^46^ Subsequent reactions of IPr*R·HCl with Cu_2_O (1 equiv.) in toluene at 100 °C for 4–20 h produced [(IPr*R)CuCl] complexes in 60–85% yield. These complexes have been characterized by NMR spectroscopy and single crystal X-ray diffraction (XRD) (Fig. S3–S6†).

The difference in quantitative steric descriptors (%*V*_bur_ and solid angles) within the IPr*R ligand family is small (see ESI† for details).^47,48^ The primary coordination sphere (*i.e.* within 5 Å of the Cu center) remains approximately the same across the series, and the trend in steric bulk is IPr*Cl ≈ IPr*Me < IPr*Et ≈ IPr*OMe ≪ IPr*^t^Bu.

**Fig. 2:**
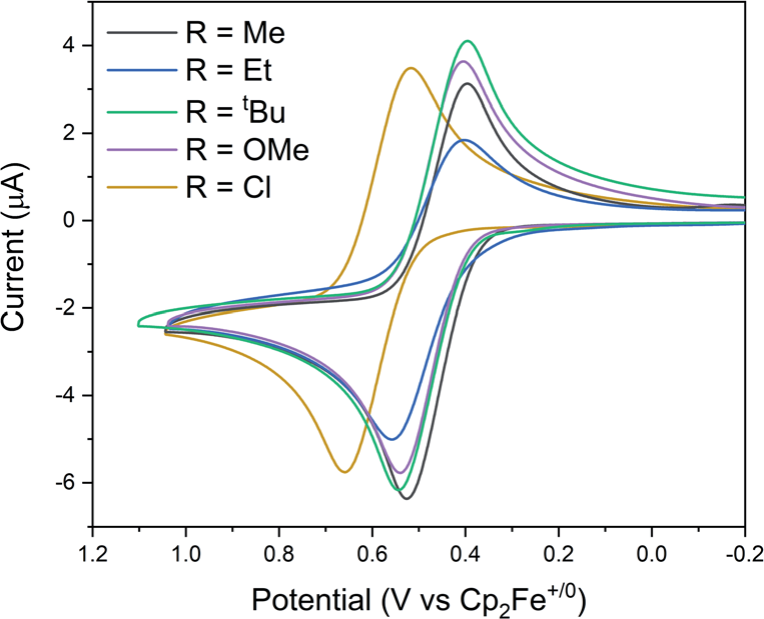
Cyclic voltammograms of [(IPr*R)Rh(COD)Cl] in CH_2_Cl_2_ containing 0.1 M NBu_4_PF_6_ as supporting electrolyte. Scan rate = 100 mV s^−1^.

To determine whether the remote substituents significantly alter the electronic properties of the IPr*R ligands, we measured the electrochemical potential of the Rh(i/ii) couple of [(IPr*R)Rh(COD)Cl] by cyclic voltammetry (Fig. 2); this method can be more sensitive to subtle changes than the Tolman electronic parameter (TEP).^38,39,49^ The voltammograms of the Rh complexes with R = Me, Et, ^*t*^Bu, and OMe show reversible Rh(i/ii) couples at similar potentials (*E*_1/2_ = 475, 481, 469, and 472 mV *vs.* Cp_2_Fe^+/0^, respectively) indicating effectively identical donor properties, consistent with the similar Hammett *σ*_p_ parameters of these substituents (−0.17, −0.15, −0.20, and −0.27, respectively).^50^ We note that IPr*OMe has been suggested to be slightly more electron-donating than IPr*Me based on TEP values (2051.1 cm^−1^ and 2052.7 cm^−1^, respectively).^51,52^ The Rh(i/ii) couple for [(IPr*Cl)Rh(COD)Cl] is significantly more positive (*E*_1/2_ = 588 mV *vs.* Cp_2_Fe^+/0^), consistent with introduction of an electron-withdrawing Cl (*σ*_p_ = 0.23), resulting in a less strongly donating ligand. The trend in electron-donating abilities of the IPr*R series is therefore IPr*Cl < IPr*Me ≈ IPr*Et ≈ IPr*OMe ≈ IPr*^t^Bu.

### Preparation and characterization of [(IPr*R)CuH]_2_

**Scheme 1:**
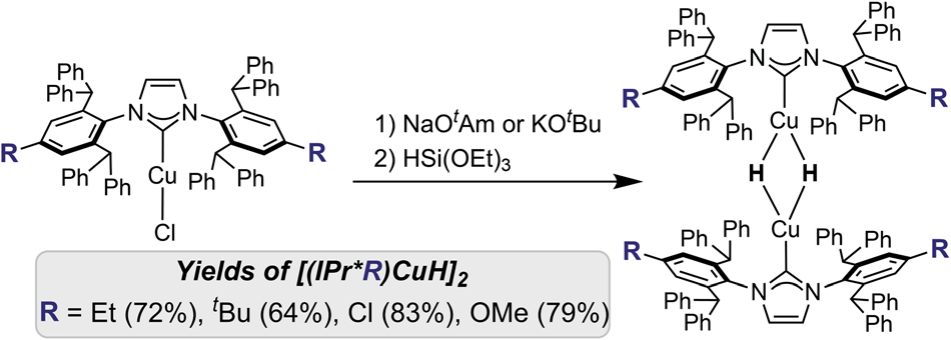
Synthesis of [(IPr*R)CuH]_2_.

**Fig. 3:**
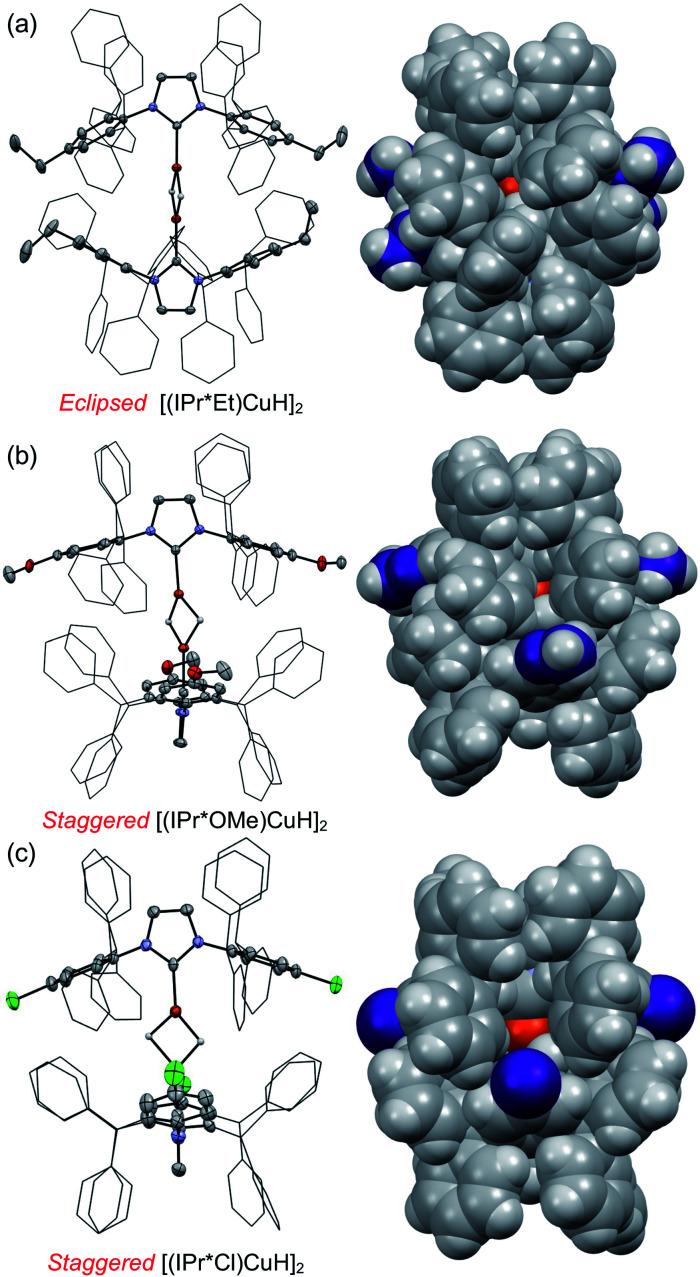
XRD structures of [(IPr*R)CuH]_2_ with R = Et (a), R = OMe (b), and R = Cl (c). On the left, thermal ellipsoids are shown at the 50% probability level. Hydrogen atoms other than the Cu–H are omitted, and the CHPh_2_ groups are shown in a wireframe representation. On the right, space-filling models of the [(IPr*R)CuH]_2_ are shown in the same orientation as the structures on the left; the R-groups are shown in purple. The hydrides in these structures were located in the difference map, and their locations were refined without restraints.

Treatment of [(IPr*R)CuCl] with NaO^*t*^Am (O^*t*^Am = *tert*-amoxide) or KO^*t*^Bu, followed by HSi(OEt)_3_ at room temperature, provided the [(IPr*R)CuH]_2_ complexes as bright yellow to orange solids in 60–80% isolated yield (Scheme 1). XRD structures of [(IPr*R)CuH]_2_ (R = Et, OMe, Cl) are shown in Fig. 3, along with space-filling models that highlight the steric congestion in these complexes. A connectivity structure of [(IPr*^t^Bu)CuH]_2_ is shown in Fig. S20.†

**Fig. 4:**
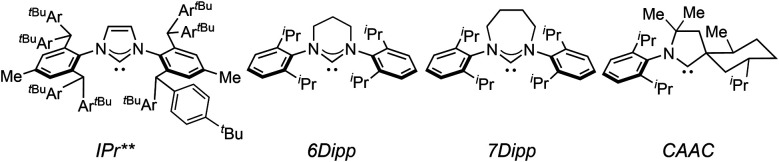
Ligands for structurally characterized [(carbene)CuH]_2_ complexes (see Table 1).

The XRD structures of [(IPr*R)CuH]_2_ closely resemble those of other [(carbene)CuH]_2_ complexes (Table 1). The Cu–Cu distances of 2.32–2.36 Å are comparable to those of reported [Cu(μ-H)]_2_ structures, which have remarkably similar Cu–Cu distances regardless of the ancillary ligands.^26,28,30,32,33,53^ The short Cu–Cu distances are likely a result of geometric constraints arising from the short Cu–H bond lengths of the dimeric [Cu(μ-H)]_2_ core. This conclusion is supported by a comparison of [(IPr)CuH]_2_ (Cu–Cu distance = 2.304(6) Å) to the dicopper monohydride [(IPr)Cu(μ-H)Cu(IPr)]BF_4_, which has a longer Cu–Cu distance of 2.5331(15) Å (IPr = 1,3-bis(2,6-diisopropylphenyl)-imidazol-2-ylidene).^54^ The Cu–C_carbene_ bond distances differ among the [(IPr*R)CuH]_2_ complexes, but there is no apparent correlation between the identity of the IPr*R ligand and the Cu–carbene bond length. The C_carbene_–Cu–Cu–C_carbene_ units are nearly linear for [(IPr*R)CuH]_2_, as indicated by the average C_carbene_–Cu–Cu angles of 176°–177°. In contrast, the Cu–NHC units of [(IPr)Cu(μ-H)]_2_ and [(IPr**)Cu(μ-H)]_2_ are slightly more tilted relative to each other, with C_carbene_–Cu–Cu angles of 166–172° and 170°, respectively (the IPr** ligand is shown in Fig. 4). For R = Cl, OMe, and ^*t*^Bu, the IPr*R ligands adopt a staggered conformation in the solid state, as indicated by the ∼90° twist angle between the Cu–carbene planes. For [(IPr*Et)CuH]_2_ the NHC ligands are closer to eclipsed, with a 16° angle between the Cu–carbene units. An eclipsed conformation of the carbene ligands was also observed for [(IPr)Cu(μ-H)]_2_,^33^ [(6Dipp)Cu(μ-H)]_2_,^32^ and [(CAAC)Cu(μ-H)]_2_.^28^ In contrast, [(IPr**)Cu(μ-H)]_2_ (ref. 30) and [(7Dipp)Cu(μ-H)]_2_ (ref. 32) adopt intermediate conformations, with twist angles of 44° and 28°, respectively, between the Cu–carbene units.

**Table 1:** Comparison of geometric parameters of [(IPr*R)CuH]_2_ to other [(carbene)CuH]_2_

Ligand^a^	Cu–Cu (Å)	C–Cu^b^ (Å)	C–Cu–Cu^c^ (°)	Carbene twist angle^d^ (°)	Ref.
IPr*Et	2.32(1)	1.903(6)	177(2)	16.4(4)	This work
IPr*OMe	2.3533(8)	1.930(7)	176(2)	89.21(6)	This work
IPr*Cl	2.3561(8)	1.909(5)	177(1)	89.7(1)	This work
IPr**	2.324(1)	1.895(6)	170.4(2)	44.3(3)	29
IPr	2.304(6)	1.88(2)	170(2)	10(3)	35
6Dipp	2.3286(5)	1.908(2)	174.76(5)	0	31
7Dipp	2.329(1)	1.916(3)	179.96(7)	27.71(7)	31
CAAC	2.3058(5)	1.862(4)	177(1)	10.7(2)	28

a The ligands IPr**, 6Dipp, 7Dipp, and CAAC are shown in Fig. 4.

b Average C_carbene_–Cu bond length.

c Average C_carbene_–Cu–Cu angle.

d Angle between the two planes defined by the Cu–imidazole (IPr derivatives), N–C_carbene_–N–Cu (6Dipp and 7Dipp), or N–C_carbene_–Cu (CAAC) units.

We reasoned that the observed differences in ligand orientation among structurally characterized [(carbene)Cu(μ-H)]_2_ could be solely a solid-state phenomenon. To determine the energy difference between the different structures observed for [(IPr*R)CuH]_2_, DFT calculations were performed on staggered and eclipsed conformers with R = H, Cl, Me, and OMe. At the ωB97XD level in benzene at 298 K, the free energies of the eclipsed conformers are calculated to be only 1.4–2.8 kcal mol^−1^ lower than those of the staggered conformers (Table S8†), implying that both conformers would be present in solution at room temperature. As discussed below, the distinct spectroscopic features of each of the complexes in solution corroborate this interpretation.

**Fig. 5:**
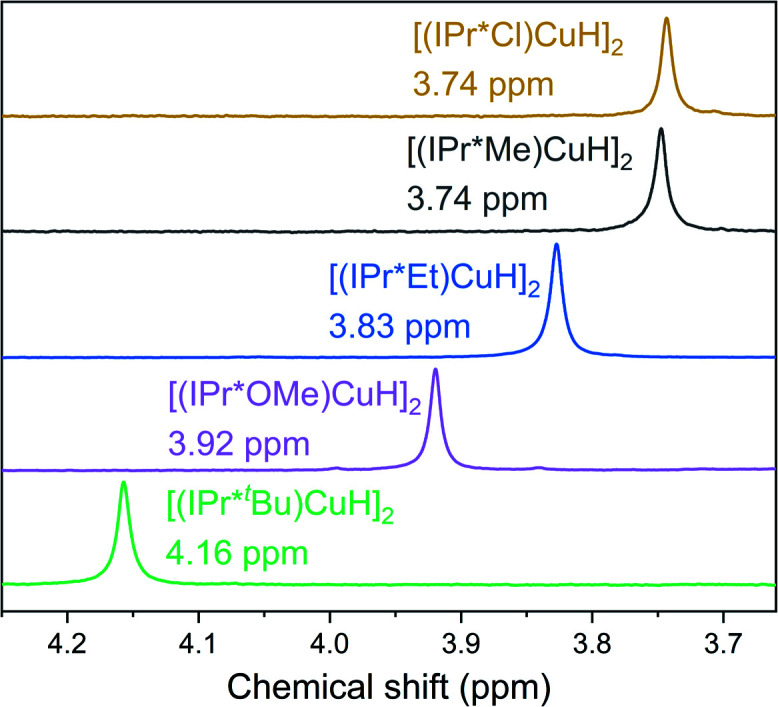
^1^H NMR spectra (∼3 mM in C_6_D_6_ at 25 °C) showing the Cu–H resonances of [(IPr*R)CuH]_2_.

Only a single Cu–H resonance, which we assign to the dimeric form of the complex, is observed in the ^1^H NMR spectra of the [(IPr*R)CuH]_2_ complexes (Fig. 5). We find no evidence for formation of discrete Cu–H monomers in any of the [(IPr*R)CuH]_2_ complexes in C_6_D_6_ at 25 °C, as is generally the case for [(carbene)Cu(μ-H)]_2_.^28,31–34^ Two exceptions are the Cu–H complex with IPr**, which exists as a mixture of monomer and dimer in solution at 25 °C,^30^ and a Cu–H complex with an NHC-capped cyclodextrin, which is monomeric in solution.^55^ DFT calculations (ωB97XD) predict that the free energies for the dimeric structures at 298 K in benzene are 23–25 kcal mol^−1^ lower than the corresponding Cu–H monomers for R = Cl, Me, and OMe. The M06 functional predicts that this energy difference is smaller, but the dimer is still favored. The B3LYP functional predicts that the monomer is significantly more stable than the dimer, which we attribute to the lack of dispersion correction in this functional since dispersive interactions between the ligands are expected to stabilize the dimer.

Despite the similar solid-state structures of the [(IPr*R)CuH]_2_, significant differences are observed in the chemical shifts of the hydride resonances. The high sensitivity of Cu–H chemical shifts to ligand identity in other systems has been attributed to changes in both NHC donor strength and to shielding of the hydrides as a result of the positioning of the ligand *N*-aryl groups,^31^ which implies that if the solid-state structures observed for the [(IPr*R)CuH]_2_ with R = OMe, Cl, and ^*t*^Bu were retained in solution, the chemical shift of the Cu–H resonance would be similar for all of these complexes. We hypothesize that the observed Cu–H resonance represents the ensemble average of multiple rapidly exchanging conformers. Because we do not fully understand the structural dynamics in this system, we cannot quantitatively interpret the experimentally observed changes in hydride chemical shift as a function of R. It is unlikely, however, that this phenomenon is the result of a change in the relative population of rapidly exchanging monomeric and dimeric complexes. If that were the case, the hydride chemical shift should decrease with increasing steric bulk by analogy to [(IPr**)CuH]_2_, which has Cu–H resonances at 2.14 and 4.26 ppm for monomer and dimer, respectively.^30^ Instead, we observe an increase in the chemical shift of the hydride resonance with bulkier ligands. We tentatively attribute the change in chemical shift for the Cu–H resonances to changes in the relative populations of [(IPr*R)CuH]_2_ conformers with different ligand orientations, which leads to differences in the average exposure of the hydrides to shielding and deshielding regions of nearby aryl rings, and hence different chemical shifts.

**Fig. 6:**
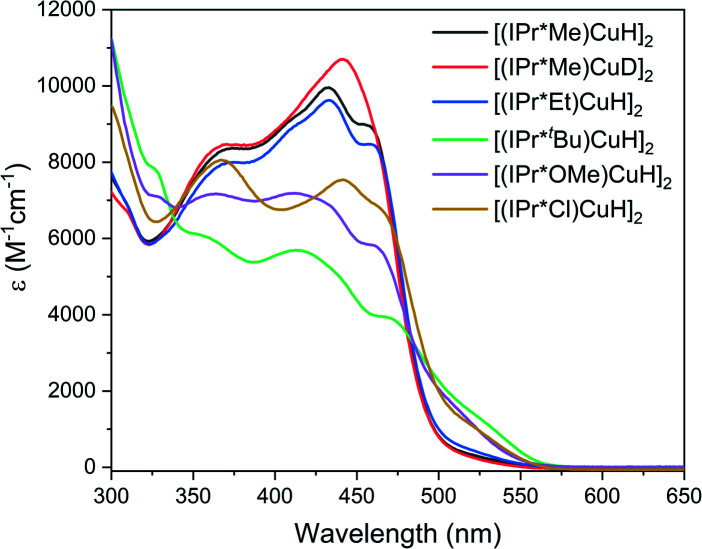
UV-visible spectra of [(IPr*R)CuH]_2_ in toluene at 25 °C.

The UV-visible spectra of the [(IPr*R)CuH]_2_ complexes in toluene at 25 °C (Fig. 6) exhibit multiple absorptions in the visible region, with *ε* between 5000 and 10 000 M^−1^ cm^−1^. These features give rise to the intense yellow color that is typical of [(NHC)Cu(μ-H)]_2_.^28,30–33,56^ In contrast, the dicopper monohydride [(IPr)Cu(μ-H)Cu(IPr)]BF_4_, in which the Cu–Cu distance is elongated by 0.23 Å, is colorless^54^ which suggests that the transitions are a result of the short Cu–Cu distance enforced by the bridging hydrides.

The UV-visible spectra of the [(IPr*R)CuH]_2_ complexes differ as a function of the electronic and the steric properties of the ligands. The absorption features of [(IPr*Me)CuH]_2_ and [(IPr*Cl)CuH]_2_, which have sterically comparable but electronically distinct ligands, are similar but appear at different energies. In contrast, the spectra of [(IPr*Me)CuH]_2_ and [(IPr*Et)CuH]_2_, which have electronically equivalent but sterically different ligands, are nearly identical. [(IPr*^t^Bu)CuH]_2_ has a broader spectrum with lower-intensity features; we attribute this phenomenon to a more significant change in solution speciation arising from the very bulky *tert*-butyl groups. The overall lower-intensity features of [(IPr*^t^Bu)CuH]_2_ could also indicate that some monomeric Cu–H (which is not expected to absorb significantly in the visible region) is formed at the ∼10× lower concentration used in UV-visible studies compared to ^1^H NMR experiments. We have not been able to further investigate this possibility due to the instability of this complex at low concentration (<50 μM). Interestingly, there is a small change in the shape of the absorption profile for [(IPr*Me)CuH]_2_ compared to [(IPr*Me)CuD]_2_, which could arise from the difference in the Cu–Cu distance enforced by Cu–D bonds compared to Cu–H bonds.

The features in the UV-visible absorption spectra of [(NHC)Cu(μ-H)]_2_ have not been studied in detail previously, but have been proposed to arise from transitions between a Cu–Cu dσ* orbital and a Cu–Cu pσ orbital by analogy to bimetallic Pt and Pd complexes.^31^ To further understand the nature of the transitions in the UV-visible spectra of [(IPr*R)CuH]_2_, we performed TD-DFT calculations on the staggered and eclipsed conformers of [(IPr*H)CuH]_2_.

For the eclipsed conformer, the calculations predict two intense bands centered at 460 nm and 375 nm, in good agreement with the experimental UV-visible spectra (Fig. S89†). The 460 nm feature arises from transitions between the HOMO, which is an orbital localized on the Cu_2_H_2_ unit, and the LUMO and LUMO + 2, which are π* orbitals on the *N*-aryl rings of the IPr*R ligand (Fig. S94†). The feature at 375 nm arises from transitions between the HOMO and higher-energy unoccupied orbitals primarily composed of π* orbitals on the CHPh_2_ groups (Fig. S94†). Changes in the R-group result in small shifts in the predicted energies of these transitions, particularly for the lower-energy feature. In support of these assignments, in the experimental spectra of the [(IPr*R)CuH]_2_ complexes the higher-energy feature (which arises from transitions to the CHPh_2_ groups and therefore should not be very sensitive to ligand identity) is observed at approximately 365 nm for all complexes. In contrast, the most intense lower-energy features are sensitive to ligand identity; in the experimental UV-visible spectrum, these features are redshifted by ∼10 nm for [(IPr*Cl)CuH]_2_ compared to the other [(IPr*R)CuH]_2_, which can be attributed to the significantly different electronic properties of IPr*Cl compared to the other IPr*R ligands. Furthermore, the UV-visible spectrum of [(6Dipp)CuH]_2_, which lacks CHPh_2_ groups, features only a single high-intensity band at 453 nm.^31^

In the staggered conformation, the lowest-energy absorption feature in the predicted spectrum is redshifted by 60 nm (Fig. S90†). This finding suggests that the UV-visible spectrum is sensitive to ligand conformation; the observed changes in the absorption spectra for the different [(IPr*R)CuH]_2_ could therefore also reflect changes in the relative population of different conformers in solution.

### Insertion reactions and mechanistic studies with [(IPr*Me)CuH]_2_

**Scheme 2:**
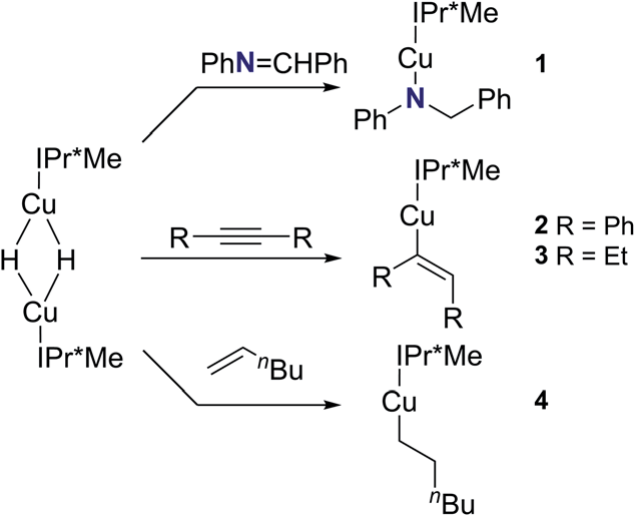
Insertion products for reactions of [(IPr*Me)CuH]_2_ with aldimine, alkyne, and α-olefin substrates.

**Fig. 7:**
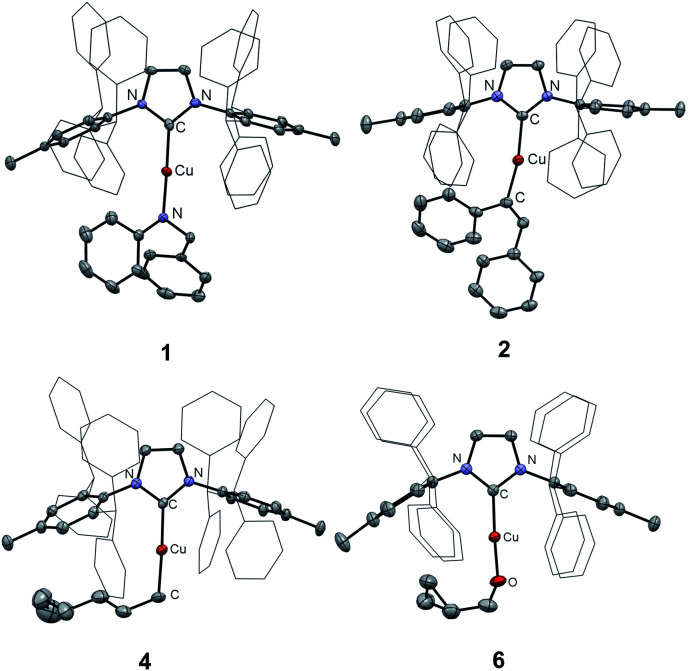
XRD structures for complexes **1**, **2**, **4**, and **6** with thermal ellipsoids shown at the 50% probability level. The ligand CHPh_2_ groups are shown in wireframe representation. Hydrogen atoms and solvent molecules are omitted.

We examined the reactions of [(IPr*Me)CuH]_2_ with *N*-benzylideaniline, diphenylacetylene, 3-hexyne, and 1-hexene (Scheme 2). The insertion products, [(IPr*Me)Cu–N(Ph)CH_2_Ph] (**1**), [(IPr*Me)Cu–C(Ph)=CH(Ph)] (**2**), [(IPr*Me)Cu–C(Et)=CH(Et)] (**3**), and [(IPr*Me)Cu–hexyl] (**4**), were cleanly generated *in situ* at 25 °C in C_6_D_6_, as demonstrated by ^1^H NMR spectroscopy (Fig. S36–S38†). On preparative scale, **1–4** were isolated as colorless to yellow crystalline solids in 65–80% yield. These species were characterized by ^1^H and ^13^C NMR spectroscopy, and XRD structures were obtained for **1**, **2**, and **4** (Fig. 7). Reactions of [(NHC)Cu(μ-H)]_2_ with diphenylacetylene, 3-hexyne, or 1-hexene to produce [(IPr)Cu–C(Ph)=CH(Ph)],^57^ [(IPr)Cu–C(Et)=CH(Et)],^33^ and [(6Dipp)Cu–hexyl],^31^ which are analogous to **2**, **3**, and **4**, respectively, have been reported previously.

**Scheme 3:**
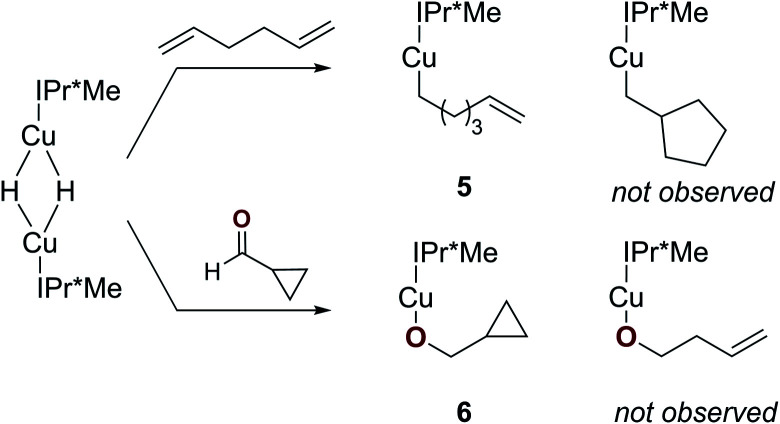
Reactions of [(IPr*Me)CuH]_2_ with radical clock substrates.

To determine whether a radical mechanism for Cu–H insertion may be occurring, we examined the reactivity of [(IPr*Me)CuH]_2_ with radical clock substrates (Scheme 3).^58,59^ Reaction of [(IPr*Me)CuH]_2_ with 1,5-hexadiene generated [(IPr*Me)Cu–CH_2_(CH_2_)_3_C(H)=CH_2_] (**5**), with no evidence for rearrangement to a ring-closed product based on ^1^H NMR spectroscopy. Similarly, the reaction of [(IPr*Me)CuH]_2_ with cyclopropanecarboxaldehyde cleanly produced [(IPr*Me)Cu–OCH_2_C_3_H_5_] (**6**) based on ^1^H NMR spectroscopy, with no evidence for a ring-opened product that would have provided evidence for a radical pathway. The identity of **6** was further confirmed by XRD (Fig. 7). These results suggest that the insertions proceed by hydride transfer rather than a pathway involving hydrogen atom transfer.

We previously identified two possible rate-limiting steps for the insertions of carbonyl substrates with [(IPr*Me)CuH]_2_: formation of a Cu–H monomer (kinetic regime 1) and hydride transfer from a Cu–H monomer (kinetic regime 2).^32^ To determine the kinetic behavior for aldimine, alkyne, and α-olefin substrates, we monitored the reactions of [(IPr*Me)CuH]_2_ with these substrates in toluene at 25 °C using UV-visible spectroscopy. The results are summarized in Table 2.

**Table 2:** Summary of rates of reaction of [(IPr*Me)CuH]_2_ with substrates in the two kinetic regimes

Substrate	Regime 1 *k*_obs_^a^ (×10^−3^ s^−1^)	Regime 2 *k*_3/2_^b^ (×10^−5^ M^−0.5^ s^−1^)
3-Hexyne	7.4	
*N*-Benzylideneaniline	7.2	
Benzophenone	7.8	
1-Hexene		5.0 (3.4^c^)
1,5-Hexadiene		18

a Average *k*_obs_ for reactions of 0.1 mM [(IPr*Me)CuH]_2_ with substrate at two different concentrations.

b Composite rate constant determined from reactions of 0.2 mM [(IPr*Me)CuH]_2_ with substrate at 3–4 different concentrations.

c For reaction with [(IPr*Me)CuD]_2_. The KIE for reactions in kinetic regime 1 (*k*_H_/*k*_D_ = 0.86 ± 0.07) was previously reported.^32^

**Fig. 8:**
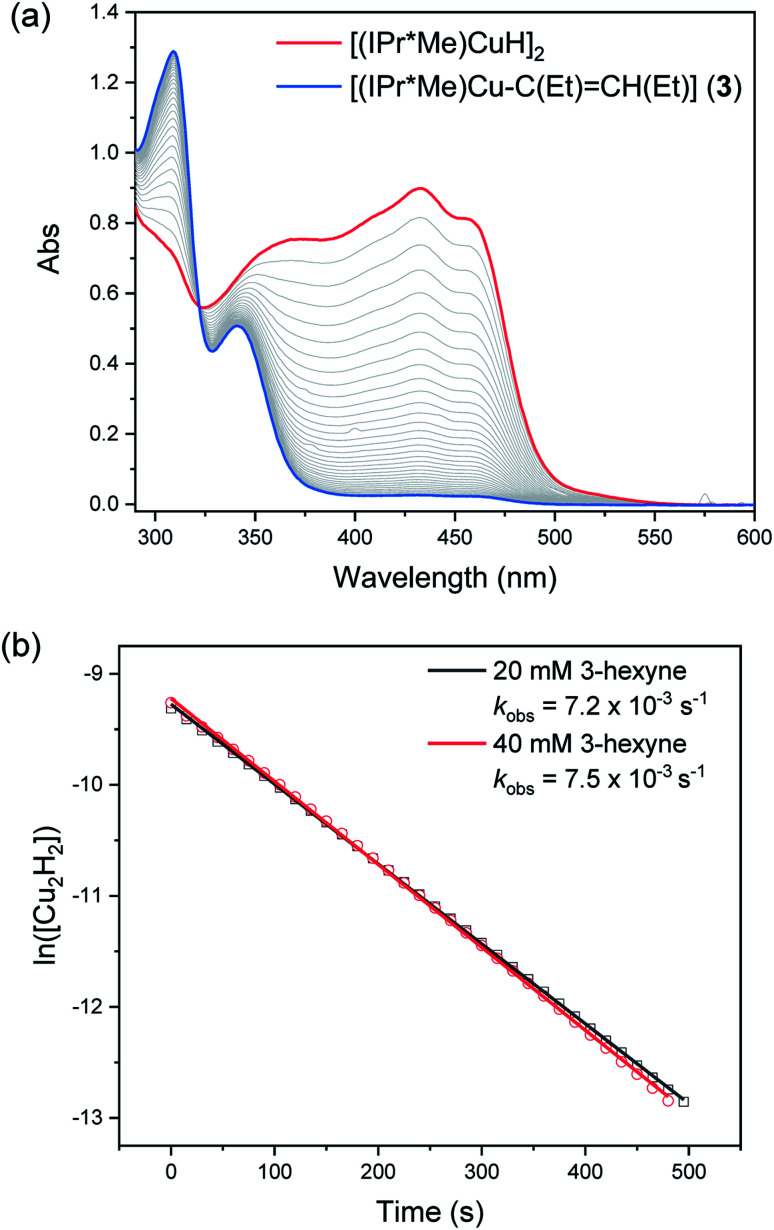
(a) UV-visible spectra of the reaction of 0.1 mM [(IPr*Me)CuH]_2_ with 20 mM 3-hexyne in toluene at 25 °C. (b) First-order fits of kinetics data for reactions of 0.1 mM [(IPr*Me)CuH]_2_ with 3-hexyne.

The reaction of 0.1 mM [(IPr*Me)CuH]_2_ with 20 or 40 mM 3-hexyne was complete within 10 minutes. Representative kinetics traces are shown in Fig. 8. The reaction is first-order in [(IPr*Me)CuH]_2_ and zero-order in 3-hexyne. A similar rate was observed for insertion of *N*-benzylideneaniline (see ESI†) and for the previously reported insertions of activated carbonyls.^32^ These results indicate that the insertions of alkyne and aldimine substrates with [(IPr*Me)CuH]_2_ fall within kinetic regime 1. We note that these results do not rigorously rule out rate-limiting opening of a single Cu–H bond to give [(IPr*Me)Cu(H)(μ-H)Cu(IPr*Me)], as suggested for alkyne insertion with an iron hydride dimer bearing a β-diketiminate ligand.^60^ The half-order kinetics determined in reactions with other substrates (see below), however, and the observation that the mixed [(6Dipp/7Dipp)CuH]_2_ species is formed upon mixing [(6Dipp)CuH]_2_ and [(7Dipp)CuH]_2_,^32^ both support our proposal of formation of a transient Cu–H monomer in (NHC)Cu–H systems.

**Fig. 9:**
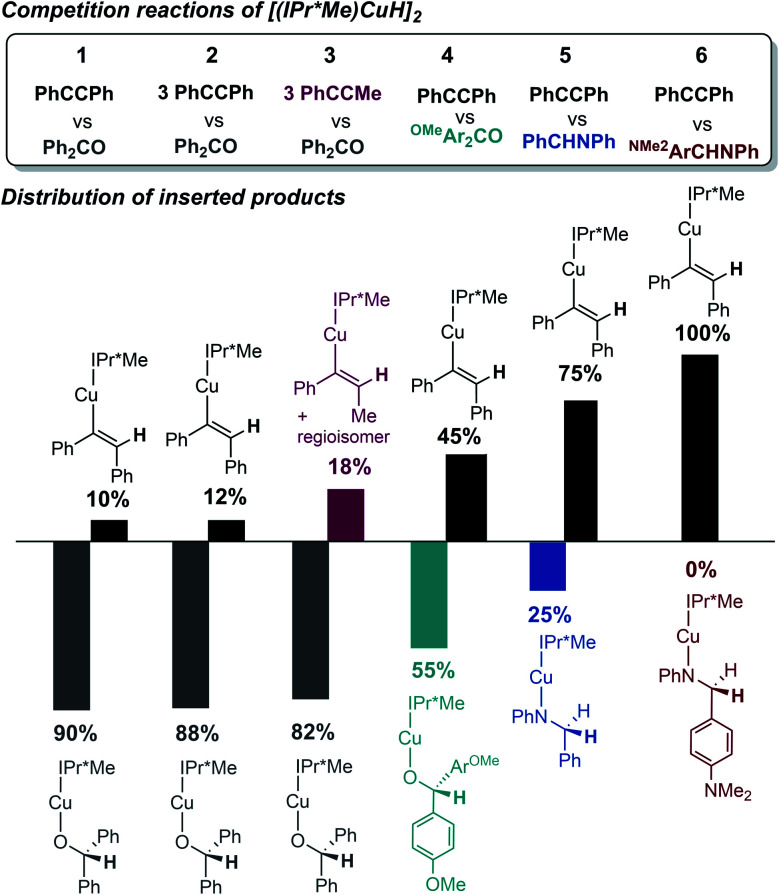
Product distributions (determined by ^1^H NMR spectroscopy in C_6_D_6_) for competition reactions of [(IPr*Me)CuH]_2_ with ketone, alkyne, and aldimine substrates.

Since the *k*_obs_ values in regime 1 reflect the rate of formation of Cu–H monomer, competition experiments of [(IPr*Me)CuH]_2_ with equimolar ratios of benzophenone, diphenylacetylene, and *N*-benzylideneaniline were performed to gauge the relative insertion rates of these substrates. As shown in Fig. 9, the trend for insertion into Cu–H is ketone > alkyne > aldimine (entries 1 and 5). Neither increasing the amount of a less reactive substrate nor changing its steric profile (entry 1 *vs.* 2 and 3) resulted in a significant erosion in chemoselectivity. As shown in our previous studies of insertion of benzophenone derivatives,^32^ however, introduction of electron-donating groups in substrates decreased their relative insertion rate, resulting in a change in product distribution (entries 1 *vs.* 4 and 5 *vs.* 6 in Fig. 9).

**Fig. 10:**
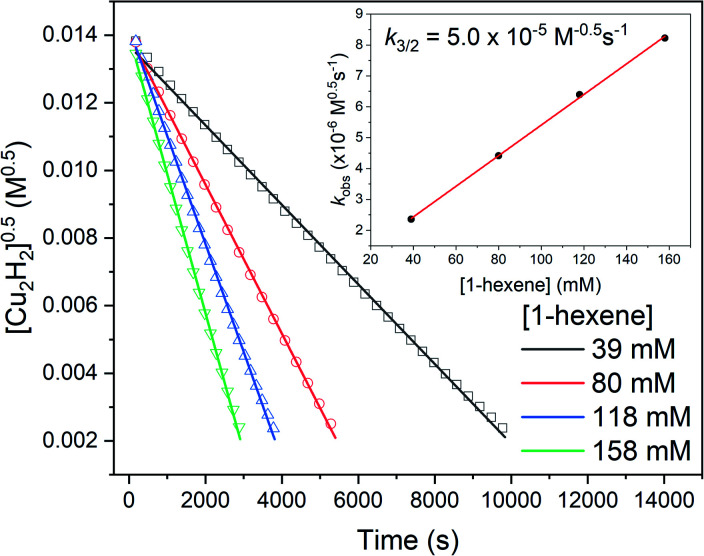
Half-order fits of kinetics data for the reactions of 0.2 mM [(IPr*Me)CuH]_2_ with different concentrations of 1-hexene. The inset shows the determination of *k*_3/2_.

The reactions of 0.2 mM [(IPr*Me)CuH]_2_ with 40–160 mM 1-hexene occur over several hours and exhibit half-order kinetics in [(IPr*Me)CuH]_2_ (Fig. 10). The *k*_obs_ values show a first-order dependence on 1-hexene concentration, with a composite three-halves order rate constant *k*_3/2_ = 5.0 × 10^−5^ M^−0.5^ s^−1^. These findings indicate the reaction falls within kinetic regime 2. In contrast to the substrates that fall into regime 1, which insert at the same rate regardless of substrate identity, substrates that fall in regime 2 should react at different rates, depending on their hydride-accepting ability. Accordingly, the reaction of 1,5-hexadiene with [(IPr*Me)CuH]_2_ is 3.6× faster than the reaction with 1-hexene (*k*_3/2_ = 18 × 10^−5^ M^−0.5^ s^−1^, see Fig. S55†).

The reaction orders of [(IPr*Me)CuH]_2_^0.5^ and [1-hexene]^1^ cannot distinguish whether hydride transfer or substrate binding to a transient Cu–H monomer is the rate-limiting step for α-olefin insertion. We therefore determined the rates of insertion of 1-hexene with [(IPr*Me)CuH]_2_ and [(IPr*Me)CuD]_2_ in separate reactions, and obtained a normal primary kinetic isotope effect (KIE) of *k*_H_/*k*_D_ = 1.4 (Fig. S53†). Although it is somewhat small for a primary KIE, this value is similar to those found for insertion reactions in some other transition metal hydride systems.^61–63^ We note that an inverse equilibrium isotope effect (EIE) has been observed for the monomer–dimer equilibrium of (NHC)Cu–H complexes.^32^ Since *k*_3/2_ is proportional to the dimer–monomer equilibrium constant *K*_eq_^0.5^, (see below), the inverse EIE also influences the magnitude of the observed KIE for insertion. The observation of a primary KIE is consistent with rate-limiting hydride transfer, but not with rate-limiting substrate coordination.

### DFT calculations of insertions into Cu–H

**Fig. 11:**
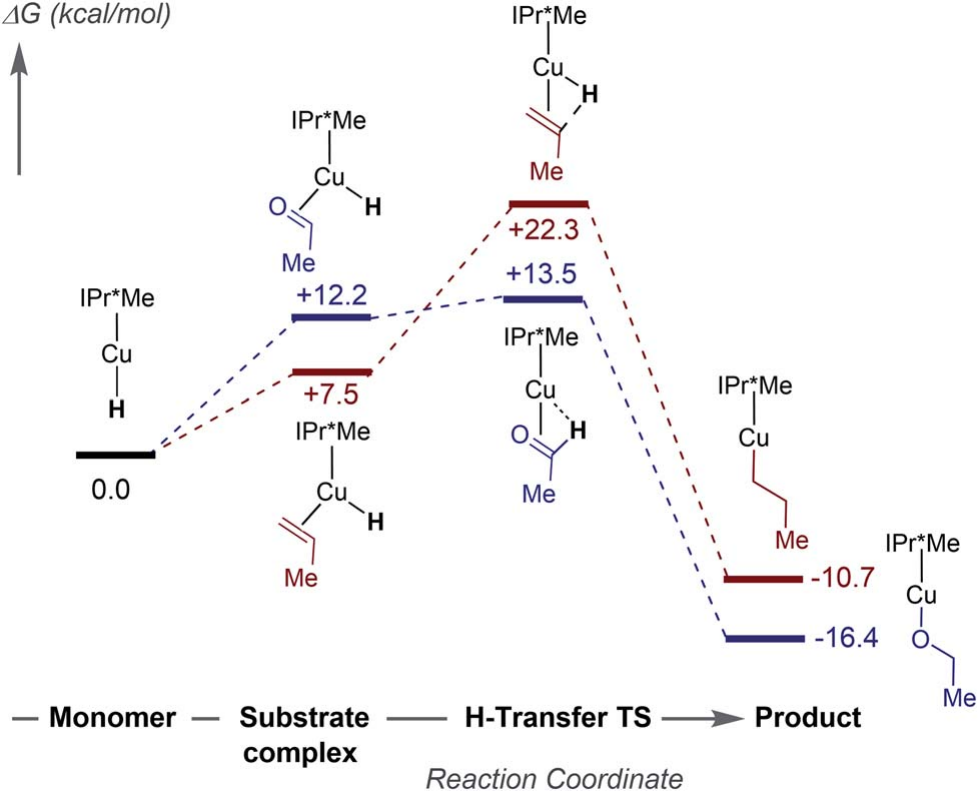
Computed (ωB97XD) free energies in kcal mol^−1^ for insertion of acetaldehyde (blue) and propene (red) with [(IPr*Me)CuH]_2_ at 298 K in benzene.

To further understand the mechanism of substrate insertion into Cu–H bonds, we performed DFT calculations using acetaldehyde and propene as model substrates for kinetic regimes 1 and 2. The first step of insertion is monomerization of the [(IPr*Me)CuH]_2_ dimer. As discussed above, the calculated energy differences between the [(IPr*R)CuH]_2_ dimers and the corresponding monomers show a strong dependence on the choice of functional. Furthermore, because of the size and conformational flexibility of this system as well as the endothermicity of the reaction, we were unable to calculate the energy of the transition state for monomerization, if one exists. The calculations therefore do not establish the rate-limiting step for insertion, but they do provide insight into the nature of the insertion reactions and the reason for the substrate-dependent change in kinetic behavior. The free energy profile for insertion at 298 K in benzene (calculated with the ωB97XD functional) is shown in Fig. 11. Additional diagrams calculated with M06 and B3LYP are in the ESI (Fig. S85–S86†).

The calculations predict that the substrates bind η^2^ to a Cu–H monomer prior to insertion (Fig. S87–S88†). Although substrate binding is unfavorable on the free energy scale, it is enthalpically favorable (Fig. S84†). In contrast, a previous study suggested formation of a weak van der Waals complex before insertion.^36,64^ Substrate binding results in elongation of the Cu–H bond and bending of the C_NHC_–Cu–H angle from 180° to ∼110°. In the transition state, the Cu–H bond is elongated by ∼0.04 Å compared to the CuH–substrate complex, and the C_NHC_–Cu–H bond angle increases (Tables S11 and S12†). These structural changes move the hydride towards the β-carbon of the substrate. Natural Population Analysis of the Natural Bond Orbitals (NBOs) indicates an increase in positive charge on the Cu center and a decrease in negative charge on the hydride for the transition state compared to the Cu–H monomer (Table S13†), in agreement with previous calculations on (IPr)CuH.^64^ The NBO analysis of the bonding in the transition state for propene insertion shows that the hydride is still mostly interacting with the Cu, whereas in the acetaldehyde transition state, the hydride has mostly transferred to the substrate, forming a C–H bond. The transition state for propene insertion is ∼9 kcal mol^−1^ higher than that for acetaldehyde insertion, which is consistent with our observation that insertion of α-olefins is slower than insertion of activated carbonyls and has a different rate-limiting step. The calculated KIEs for insertion of acetaldehyde (*k*_H_/*k*_D_ = 1.39) and propene (*k*_H_/*k*_D_ = 1.32) are similar. The small primary KIE for propene insertion is in good agreement with our experimental observations for 1-hexene insertion. Finally, formation of the Cu–alkoxide or Cu–alkyl product is ∼30 kcal mol^−1^ downhill from the transition state.

### Effect of remote ligand modifications in [(IPr*R)CuH]_2_ on the rates of Cu–H insertion reactions

**Fig. 12:**
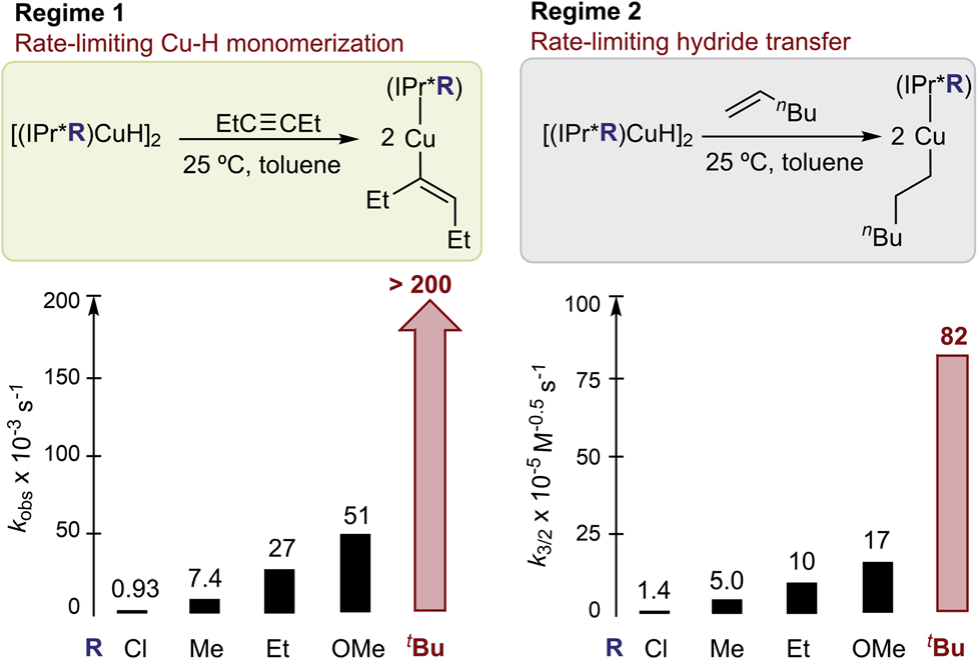
Summary of kinetics data for insertions of [(IPr*R)CuH]_2_ at 25 °C in toluene. Average *k*_obs_ for reactions of 0.1 mM [(IPr*R)CuH]_2_ with 20–40 mM 3-hexyne are shown on the left, and *k*_3/2_ determined from reactions of 0.2 mM [(IPr*R)CuH]_2_ with 3–4 concentrations of 1-hexene (10 mM-160 mM) are shown on the right.

To determine the impact of the remote substituents on reactivity, we examined the reactions of the [(IPr*R)CuH]_2_ series with 3-hexyne, *N*-benzylideneaniline, benzophenone, and 1-hexene. Based on ^1^H NMR spectroscopy, *in situ* reactions of [(IPr*R)CuH]_2_ with these substrates (10–50 equiv.) in C_6_D_6_ at 25 °C quantitatively produce the corresponding Cu–alkenyl, Cu–anilide, Cu–alkoxide, and Cu–hexyl complexes (Fig. S41–S44†). The assigned identities of the products are strongly corroborated by comparison of their ^1^H NMR data to those of compounds **1**, **3**, **4**, and [(IPr*Me)Cu–OCHPh_2_].^32^ UV-visible kinetics studies of the [(IPr*R)CuH]_2_ series were conducted under the same conditions as the experiments with [(IPr*Me)CuH]_2_ described above. The *k*_obs_ values for insertion of 3-hexyne (a representative regime 1 substrate) and *k*_3/2_ values for insertion of 1-hexene (a regime 2 substrate) are shown in Fig. 12. Detailed analyses of the kinetics data are presented in the ESI.†

For insertion of 3-hexyne, *N*-benzylideneaniline, and benzophenone, the reactions are all zero-order in substrate and first-order in [(IPr*R)CuH]_2_, supporting a conserved rate-limiting step of Cu–H monomerization within the Cu–H series. The rates of Cu–H monomerization, however, differ among the [(IPr*R)CuH]_2_ complexes. For the reactions of 3-hexyne with [(IPr*R)CuH]_2_, the *k*_obs_ increases from 7.4 s^−1^ for R = Me to 27 s^−1^ for R = Et, and becomes too fast to measure accurately (*t*_1/2_ < 6 s) for R = ^*t*^Bu, demonstrating a significant steric effect. Our data also show evidence for an electronic effect; the *k*_obs_ for insertion of 3-hexyne decreases from 7.4 s^−1^ for R = Me to 0.93 s^−1^ for R = Cl. Conversely, a rate increase from 27 s^−1^ for R = Et to 51 s^−1^ for R = OMe is observed for insertion of 3-hexyne. This finding is consistent with prior studies indicating that the kinetic hydricity of transition metal hydrides is increased by electron-rich ligands.^65,66^

Next, we examined the reactions of [(IPr*R)CuH]_2_ with 1-hexene. In all cases, the kinetics data fit well to half-order in [(IPr*R)CuH]_2_ and first-order in 1-hexene, indicating that the rate-limiting step remains hydride transfer (insertion into the Cu–H bond) for all complexes. Although the trend in insertion rates is the same (*k*_3/2_ for IPr*Cl < IPr*Me < IPr*Et < IPr*OMe ≪ IPr*^t^Bu), the magnitude of the change in rates is smaller than that observed in the reactions with 3-hexyne.

**Scheme 4:**
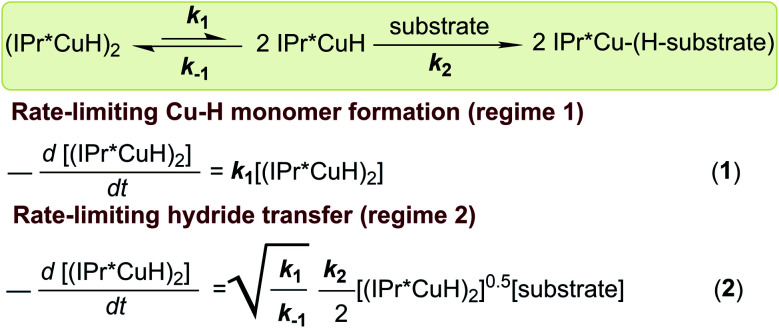
Rate laws for kinetic regimes 1 and 2.

**Fig. 13:**
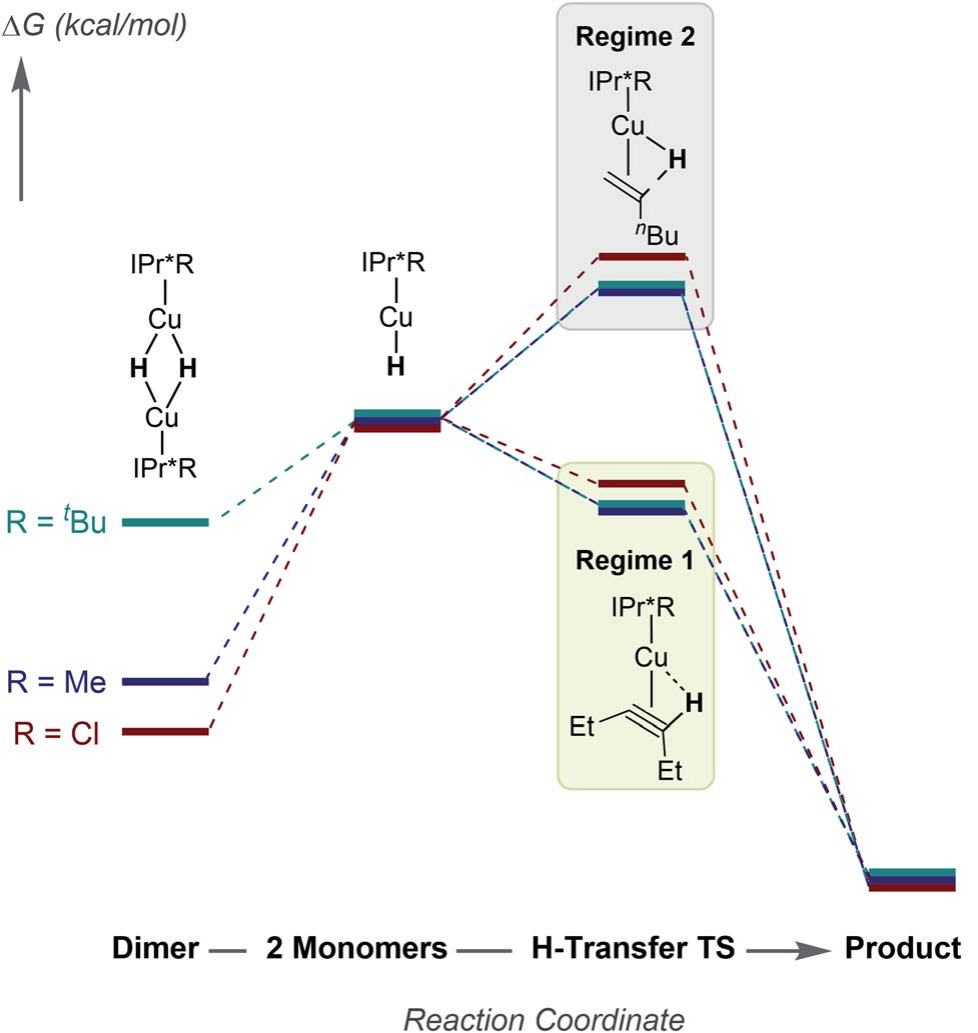
Qualitative reaction coordinate diagrams showing the impact of changes in the steric and electronic properties of IPr*R ligands on the energetics of substrate insertion with [(IPr*R)CuH]_2_.

Based on DFT calculations, changing the NHC *para* substituent has only a small (<2 kcal mol^−1^) effect on the energy difference between Cu–H dimer and Cu–H monomer and for substrate insertion (see Tables S7 and S9†). This finding is not surprising, considering that the changes in rates measured experimentally correspond to changes in barrier heights of a few kcal mol^−1^, and there are many complex interactions in these species. Although the DFT calculations are not accurate enough to provide a quantitative interpretation of the changes in reaction rates, our experimental results can be rationalized based on the rate laws for the kinetic regimes of rate-limiting Cu–H monomer formation (**1**) and rate-limiting hydride transfer (**2**), as shown in Scheme 4.^32,67,68^ Qualitative reaction coordinate diagrams illustrating the impact of changes in ligand steric and electronic properties on insertion are shown in Fig. 13.

The kinetics data indicate that the observed rate constants in both kinetic regimes increase for IPr*R with more bulky R substituents. For reactions in kinetic regime 1, the changes in rate arise from changes in *k*_1_ (the rate constant for Cu–H monomerization) indicating that ligands with bulky R groups promote faster formation of Cu–H monomer. For reactions in kinetic regime 2, the changes in rate could arise from changes in the square root of the Cu–H dimer–monomer equilibrium constant (see Scheme 4; *K*_eq_ = *k*_1_/*k*_−1_) and/or changes in *k*_2_ (the rate constant for insertion). Since the electron-donating properties of IPr*Me, IPr*Et, and IPr*^t^Bu are indistinguishable and the R-groups are too far from the Cu center to interact directly with substrate, it is likely that *k*_2_ remains relatively constant for these complexes. For regime 2 reactions, the change in *k*_3/2_ for different ligands must then arise from changes in *K*_eq_. Sterically bulky ligands therefore increase insertion rates in regime 2 by destabilizing the Cu–H dimer relative to monomer. These results demonstrate that even when they are far from the Cu center, bulky groups on NHC ligands can improve the reactivity in two ways: by shifting the monomer–dimer equilibrium towards monomer and by promoting faster formation of Cu–H monomers.

Due to the pronounced steric effects in this system, the impact of ligand electronic properties on insertion rate can only be ascertained by comparing sterically similar ligands. Since [(IPr*Cl)CuH]_2_ and [(IPr*Me)CuH]_2_ are sterically similar, we interpret the change in the insertion rates in both kinetic regimes as being due to the less-electron donating IPr*Cl ligand. Bridging hydrides are typically considered to have 3-center, 2-electron bonding.^69^ The dimeric [Cu–H]_2_ core in the [(IPr*R)CuH]_2_ series can also be considered as a Lewis basic Cu–H bond from one complex donating to the Lewis acidic site of the other Cu center. Because the IPr*Cl ligand is less electron-donating, the Cu center in [(IPr*Cl)CuH]_2_ is more Lewis acidic than the Cu center in [(IPr*Me)CuH]_2_. The monomer–dimer equilibrium should therefore lie further towards dimer for [(IPr*Cl)CuH]_2_ (*i.e. K*_eq_ is smaller). Additionally, as discussed above, NBO analysis suggests that a partial positive charge forms at the Cu center during hydride transfer, in agreement with other studies on Cu–H systems.^63,64,70–72^ The transition state for insertion would therefore be destabilized by the less electron-donating IPr*Cl ligand, resulting in a decrease in *k*_2_. From the data, it is not clear whether the decrease in *K*_eq_ or the decrease in *k*_2_ is the dominant factor in determining the overall decreased *k*_3/2_ for insertion of 1-hexene with [(IPr*Cl)CuH]_2_ in comparison to [(IPr*Me)CuH]_2_. For the reactions in regime 1, the decrease in *k*_obs_ for [(IPr*Cl)CuH]_2_ is likely a result of a smaller *K*_eq_ for this complex compared to [(IPr*Me)CuH]_2_, which results in slower monomerization. An analogous effect may explain the increase in insertion rate for [(IPr*OMe)CuH]_2_ compared to [(IPr*Et)CuH]_2_, since IPr*OMe has been suggested to be slightly more electron-donating than IPr*Et based on TEP values, although the change in rates is much smaller and could arise from other subtle differences between these two complexes.

## Conclusions

Our studies of the series of [(IPr*R)CuH]_2_ dimers (R = Cl, Me, Et, OMe, ^*t*^Bu) constitute the first systematic examination of the effect of remote ligand substitution in Cu–H complexes. The XRD structures of the [(IPr*R)CuH]_2_ complexes demonstrate that R groups in the *para* position of the NHC aryl ring on one ligand are in close proximity to the CHPh_2_ and/or R-groups on the other NHC ligand in the dimer, which explains how steric changes so far from the Cu–H center can have such a dramatic influence on the properties of the complexes. Although the solid-state structures of all of the complexes are very similar, analysis of their solution spectra, coupled to DFT calculations, suggests that multiple different conformations of the NHC ligands are accessible in solution.

Moreover, kinetics studies demonstrate that the R groups influence the rate of substrate insertion. The magnitude of this effect is more pronounced for reactions in which Cu–H monomerization is the rate-limiting step than for those in which insertion is the rate-limiting step. It is clear that [(IPr*R)CuH]_2_ with bulky or electron-donating R-groups undergo faster insertion. The analysis of the kinetics data indicates that this phenomenon is caused predominantly by destabilization of the [(IPr*R)CuH]_2_ dimer, which both shifts the monomer/dimer equilibrium towards monomer and accelerates formation of the [(IPr*R)CuH] monomer. Our findings provide compelling evidence that remote functionalization of NHC ligands with bulky organic groups is an attractive method for accelerating the rate of substrate insertion in Cu–H complexes that is complementary to the traditional approach of modifying the immediate environment surrounding the Cu center. This strategy may lead to rapid construction of new Cu–H complexes for hydrofunctionalization reactions in which hydrocupration is the rate-limiting step.

## Author contributions

BLT and RMB conceived the project. ALS and BLT performed experiments and analysed data with input from RMB. JDE collected XRD data and solved the structures. MV and DAD performed DFT calculations and analyzed computational results. ALS and BLT prepared the manuscript with input from RMB.

## Conflicts of interest

There are no conflicts to declare.

## Supplementary Material

SC-012-D1SC01911B-s001

SC-012-D1SC01911B-s002

SC-012-D1SC01911B-s003
