# Supplementary material for: Accelerating the insertion reactions of (NHC)Cu–H via remote ligand functionalization
Source: Chem Sci. 2021 Jul 29;12(34):11495–505. doi: 10.1039/d1sc01911b (PMC8409461; doi:10.1039/d1sc01911b)
Supplement: SC-012-D1SC01911B-s002 [file SC-012-D1SC01911B-s002.pdf]

Electronic Supplementary Material (ESI) for Chemical Science.
This journal is © The Royal Society of Chemistry 2021

Accelerating Insertion Reactions of (NHC)Cu-H via Remote Ligand Functionalization

Amy L. Speelman‡, Ba L. Tran‡*, Jeremy D. Erickson‡, Monica Vasiliu†, David A. Dixon†, and R. Morris Bullock‡*

‡*Institute for Integrated Catalysis, Pacific Northwest National Laboratory, Richland, WA 99352 (USA)*

†*Department of Chemistry and Biochemistry, University of Alabama, Tuscaloosa, AL 35487 (USA)*

Email: morris.bullock@pnnl.gov; ba.tran@pnnl.gov

Table of Contents

General considerations S3

Synthesis and characterization of IPr*R•HCl

- Synthetic procedures S4
- ^{1}H and ^{13}C{^{1}H} NMR spectra S6

Synthesis and characterization of [(IPr*R)CuCl]

- Synthetic procedures S8
- XRD structures S11
- ^{1}H and ^{13}C{^{1}H} NMR spectra S13

Calculation of %V_{bur} and solid angles S16

Synthesis and characterization of [(IPr*R)Rh(COD)Cl]

- Synthetic procedures S18
- ^{1}H and ^{13}C{^{1}H} NMR spectra S20

Cyclic voltammograms of [(IPr*R)Rh(COD)Cl] S24

Synthesis and characterization of copper hydride complexes

- Synthetic procedures S27
- XRD structure of [(IPr*^{t}Bu)CuH]$_2$ S31
- ^{1}H and ^{13}C{^{1}H} NMR spectra S32
- Comparison of ^{1}H NMR and UV-Visible spectra of [(IPr*OMe)CuH]$_2$ and [(IPr*OMe)Cu(OH)] S38
- ^{1}H NMR spectra showing decomposition of [(IPr*^{t}Bu)CuH]$_2$ S39

Synthesis and characterization of insertion products with IPr*Me

- Synthetic procedures S40
- ^{1}H and ^{13}C{^{1}H} NMR spectra of insertion products S43

^{1}H NMR spectra of insertion products generated *in situ* S49

UV-Visible kinetics experiments

Experimental procedure S55
Reactions with $[(IPr^*Me)CuH]_2$ and $[(IPr^*Me)CuD]_2$ S57
Reactions with $[(IPr^*Et)CuH]_2$ S63
Reactions with $[(IPr^{*t}Bu)CuH]_2$ S66
Reactions with $[(IPr^*OMe)CuH]_2$ S68
Reactions with $[(IPr^*Cl)CuH]_2$ S71
Competition experiments for reactions of $[(IPr^*Me)CuH]_2$ with carbonyls, imines, and alkynes S75
DFT calculations
Computational methods S77
Analysis of DFT-optimized (IPr*R)Cu-H structures S76
Mechanistic calculations S80
NBO analysis S86
TD-DFT Calculations S92
References S96

General Considerations

All reactions were conducted under a nitrogen atmosphere in a Vacuum Atmospheres glovebox or using standard Schlenk techniques unless otherwise noted. Reagents were purchased from Sigma-Aldrich, Alfa Aesar, TCI America, Combi-Blocks, or Oakwood Chemicals, and used as received unless otherwise noted. Toluene, THF, diethyl ether, pentane, and hexanes were sparged with N_2, purified by passage through neutral alumina using an Innovative Technology, Inc., PureSolv solvent purification system, and stored over activated 4 Å molecular sieves. 1-hexene and 1,5-hexadiene were purified by passage through a plug of silica or alumina, freeze-pump-thaw degassed, and stored over activated 4 Å molecular sieves. 3-hexyne and C_6D_6 were freeze-pump-thaw degassed and dried over activated 4 Å molecular sieves.

NMR spectra were acquired on Varian NMR spectrometers at 25 °C unless otherwise noted. The chemical shifts are referenced to tetramethylsilane (0.0 ppm) using internal $CDCl_3$ (^1H: 7.26 ppm; ^{13}C: 77.16 ppm) or C_6D_6 (^1H: 7.16 ppm; ^{13}C: 128.06 ppm) solvent resonances. UV-Visible spectra were recorded at 25 °C on a Cary 60 spectrophotometer.

XRD data were collected on a Bruker APEX-II diffractometer. For more detailed information, see the relevant CIFs for the structures. The CCDC deposition numbers are:

[(IPr*Et)CuCl]: 2050209
[(IPr*OMe)CuCl]: 2050208
[(IPr**t*Bu)CuCl]: 2050213
[(IPr*Et)CuH]$_2$: 2062532
[(IPr*OMe)CuH]$_2$: 2062776
[(IPr*Cl)CuH]$_2$: 2049358
[(IPr*Me)Cu-N(Ph)CH_2Ph] (**1**): 2050212
[(IPr*Me)Cu-C(Ph)=CH(Ph)] (**2**): 2050211
[(IPr*Me)Cu-hexyl] (**4**): 2049359
[(IPr*Me)Cu-$OCH_2C_3H_5$] (**6**): 2050210

Elemental analyses were performed by Atlantic Microlab, Inc. (Norcross, GA, USA).

Synthesis and characterization of IPr*R•HCl

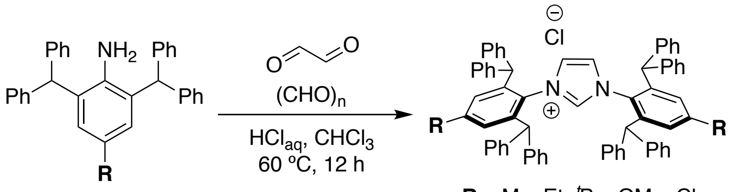

NH_2Dipp*-R

R = Me, Et, ^{t}Bu, OMe, Cl

General procedure for the synthesis of IPr*R•HCl (*R* = Me, Et, ^{t}Bu, OMe, Cl)

The preparation of IPr*R•HCl was adapted from a published procedure with minor modifications.[1] To a 150 mL round-bottom flask were added NH_2Dipp*-R (9.50 mmol), 40% aqueous glyoxal (1.50 g, 10.5 mmol) by weight, paraformaldehyde (600 mg, 10.5 mmol), $CHCl_3$ (30 mL), and a large stir bar. The reaction mixture was heated to 60 ºC, and concentrated aqueous HCl (900 μL, 10.5 mmol) was slowly added. The reaction mixture was stirred at 60 ºC for 12 h, and then all volatiles were evaporated to give a brown foam and white solid. Et_2O (50 mL) was added to the crude mixture and a spatula was used to scratch the bottom of the round-bottom flask to induce precipitation of the product. The flask was fitted with a reflux condenser and heated at 50 ºC with vigorous stirring to pulverize the solid, giving a fine off-white precipitate. After cooling, the solid was collected on a medium porousity glass frit and washed with Et_2O or hexanes (20 mL) at room temperature to give an off-white solid that was dried under vacuum.

Note: *In some cases the isolated solid was slightly brown. Additional purification was performed by adding EtOAc to the solid in a round-bottom flask fitted with a reflux condenser and heating to 60 ºC. The solid was collected from the warm solution by vacuum filtration, washed with hexanes, and dried under vacuum*.

Characterization of IPr*Me•HCl

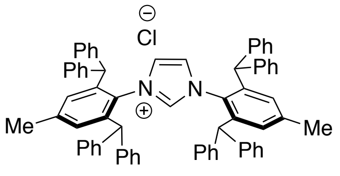

Yield = 74% (3.33 g, 3.51 mmol). **^{1}H NMR** (500 MHz, 25 ºC, $CDCl_3$): δ 12.58 (br s, 1H, imidazolium C-*H*), 7.28-7.12 (m, 36H, Ar-*H*), 6.79 (s, 8H, Ar-*H*), 6.76 (d, *J* = 7.5 Hz, 6H, Ar-*H*), 5.51 (s, 2H, *H*-C=C-*H*), 5.25 (s, 4H, C*H*(Ph)$_2$), 2.20 (s, 6H, Ar-C*H*$_3$). The ^{1}H NMR data are consistent with those published in the literature.[2]

Characterization of IPr*Et•HCl

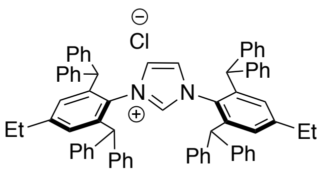

Yield = 70% (3.30 g, 3.40 mmol). **^{1}H NMR** (500 MHz, 25 ºC, $CDCl_3$): δ 12.44 (br s, 1H, imidazolium C-*H*), 7.28-7.25 (m, 10H, Ar-*H*), 7.20-7.12 (m, 26H, Ar-*H*), 6.80-6.78 (m, 8H, Ar-*H*), 5.54 (s, 2H, *H*-C=C-*H*), 5.25 (s, 4H, C*H*(Ph)$_2$), 2.48 (quartet, *J* = 7.5 Hz, 4H, C*H*$_2$CH$_3$), 1.02 (quartet, *J* = 7.5 Hz, 6H, CH$_2$C*H*$_3$). ^{1}H NMR data are consistent with those published in the literature.[3]

Characterization of IPr*^{t}Bu•HCl

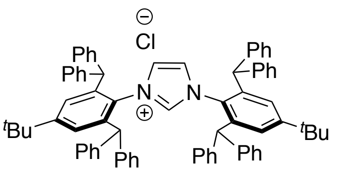

Yield = 78% (3.85 g, 3.72 mmol). **^{1}H NMR** (500 MHz, 25 ºC, $CDCl_3$): δ 12.58 (br s, 1H, imidazolium C-*H*), 7.28-7.14 (m, 34H, Ar-*H*), 6.98 (s, 4H, Ar-*H*), 6.76 (d, *J* = 7.5 Hz, 6H, Ar-*H*), 5.50 (s, 2H, *H*-C=C-*H*), 5.32 (s, 4H, C*H*(Ph)$_2$), 1.04 (s, 18H, ^{t}Bu).

^{13}C{^{1}H} NMR (126 MHz, $CDCl_3$): δ 154.18, 142.44, 142.0, 141.6, 140.2, 129.8, 129.1, 128.6, 127.4, 127.1, 126.9, 123.6, 51.5, 30.8.

Characterization of IPr*OMe•HCl

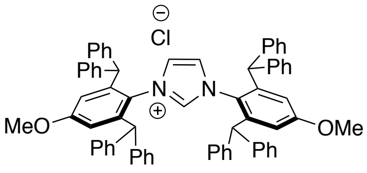

Yield = 72% (3.40 g, 3.46 mmol). **^{1}H NMR** (500 MHz, 25 °C, $CDCl_3$): δ 12.38 (br s, 1H, imidazolium C-*H*), 7.28-7.25 (m, 12H, Ar-*H*), 7.21-7.12 (m, 22H, Ar-*H*), 2.81 (d, *J* = 7.5 Hz, 6H, Ar-*H*), 6.47 (s, 4H, Ar-*H*), 5.45 (s, 2H, *H*-C=C-*H*), 5.25 (s, 4H, C*H*$(Ph)_2$), 3.52 (s, 6H, O*Me*). The ^{1}H NMR data are consistent with those published in the literature.[4]

Synthesis of IPr*Cl•HCl

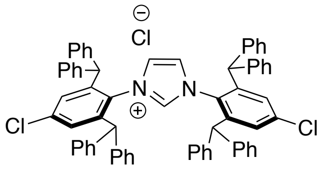

To a 500 mL round-bottom flask was added 2,6-dibenzhydryl-4-chloroaniline (30.6 g, 66.5 mmol), 40% glyoxal (10.5 g, 73.5 mmol), solid paraformaldehyde (4.20 g, 73.5 mmol), 200 mL $CHCl_3$, and a large stir bar. The reaction mixture was heated to 70 °C, and HCl (6.30 mL, 73.5 mmol) was slowly added. The reaction mixture was stirred at 70 °C for 24 h, and then all volatiles were removed to give a brown foam. To the crude mixture was added EtOAc (20 mL) and hexanes (150 mL). The mother liquor was decanted, leaving a sticky brown residue. EtOAc (20 mL) was added to the flask. The flask was fitted with a reflux condenser and heated at 70 °C with vigorous stirring for 1 hr producing a white precipitate in a brown solution. The hot mixture was filtered on a medium porosity glass frit and washed with room temperature EtOAc (20 mL x 2) and hexanes (10 mL) to give a white solid that was dried under vacuum. Yield = 11% (3.50 g, 3.53 mmol).

^{1}H NMR (500 MHz, 25 °C, d_6-DMSO): δ 12.98 (br s, 1H, imidazolium C-*H*), 7.30-7.28 (m, 10H, Ar-*H*), 7.23-7.17 (m, 16H, Ar-*H*), 7.14 (t, *J* = 7.5 Hz, 8H, Ar-*H*), 6.97 (s, 4H, Ar-*H*), 6.76 (d, *J* = 7.5 Hz, 6H, Ar-*H*), 5.48 (s, 2H, *H*-C=C-*H*), 5.29 (s, 4H, C*H*$(Ph)_2$).

^{13}C{^{1}H} NMR (126 MHz, d_6-DMSO): δ 163.5, 143.3, 141.2, 140.9, 137.0, 131.3, 129.8, 129.7, 129.2, 128.0, 127.9, 125.5, 51.3.

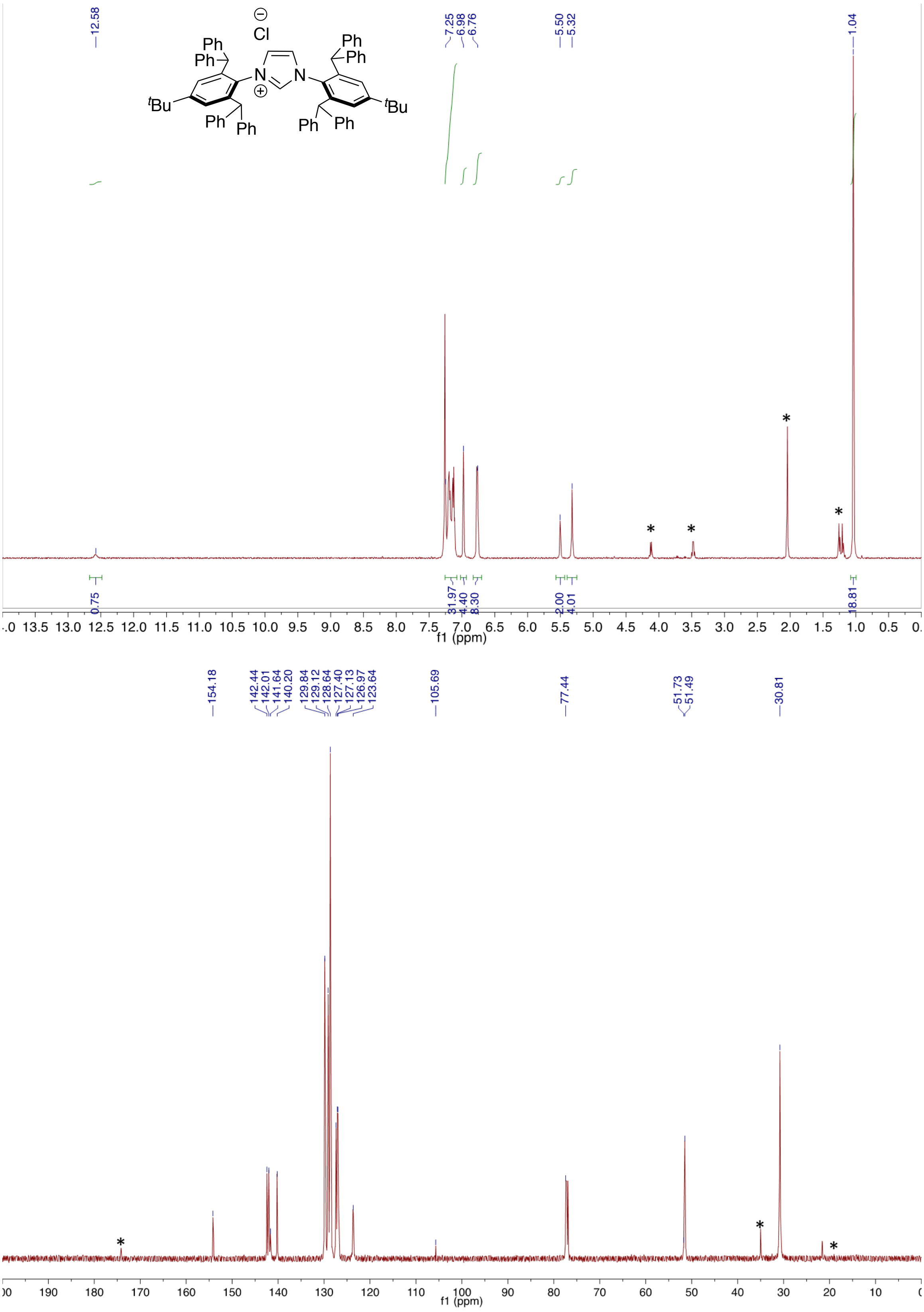

Figure S1. ^{1}H (top) and $^{13}C\{^{1}H\}$ (bottom) NMR spectra of IPr*^{t}Bu-HCl in $CDCl_3$. *Et_2O, EtOAc

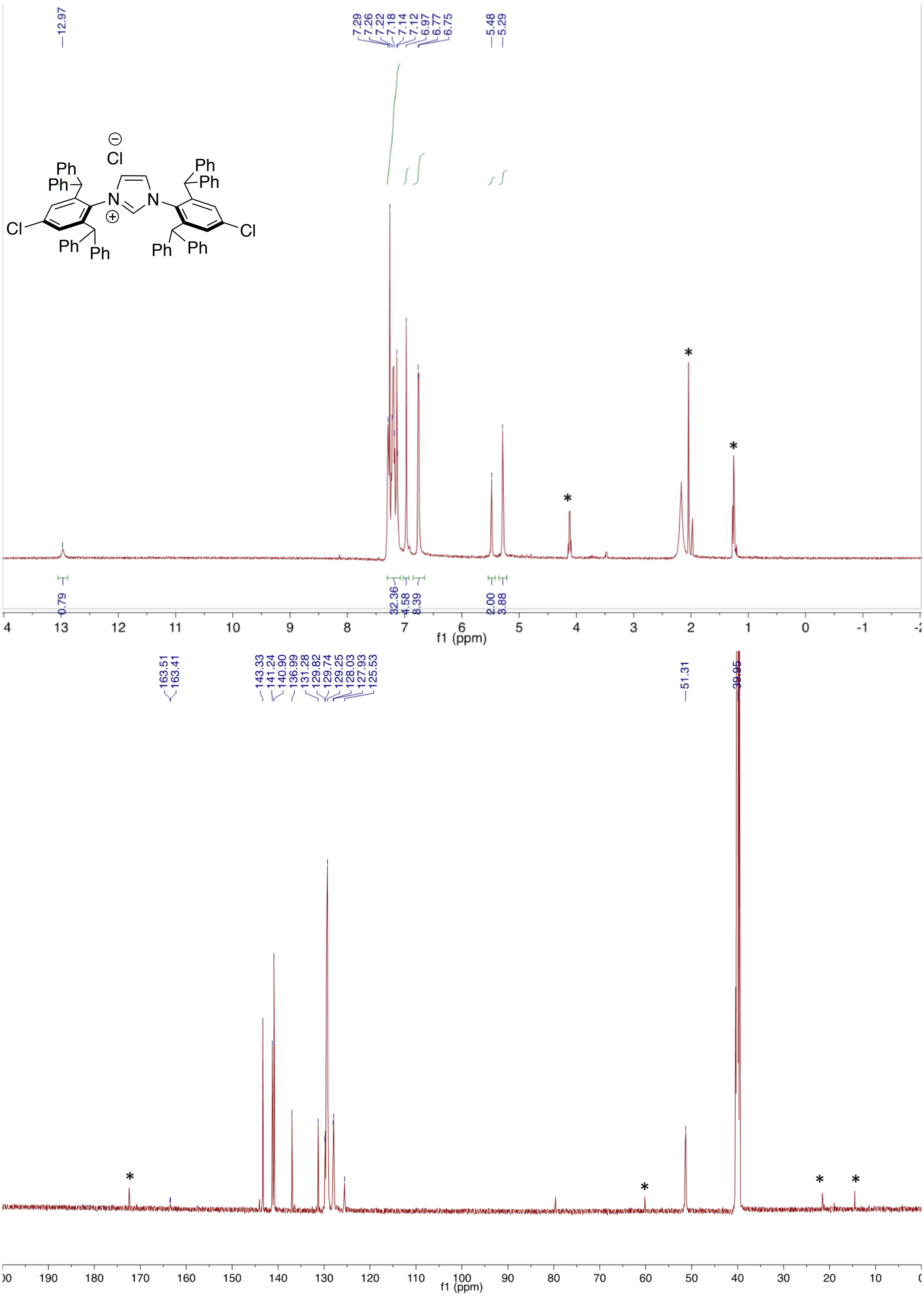

Figure S2. ^{1}H (top) and $^{13}C\{^{1}H\}$ (bottom) NMR spectra of IPr*Cl-HCl in DMSO-d_6. *EtOAc

Synthesis and Characterization of [(IPr*R)CuCl]

General notes

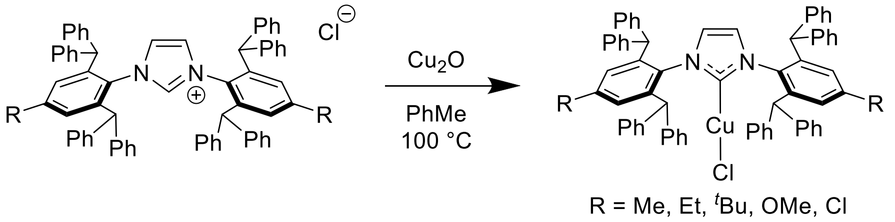

Yields:
R = Me 78%
R = Et 60%
R = ^{t}Bu 60%
R = OMe 84%
R = Cl 62%

Metallation of IPr*R•HCl to Cu was performed as described in the literature with minor modifications.[5] This reaction has been performed successfully on a 10-20 g scale. Representative procedures for the different IPr*R•HCl are given below. In some reactions, the isolated product appeared brown rather than off-white. Additional purification was performed either by triturating with methanol or by dissolving in CH_2Cl_2 and passing through a silica plug.

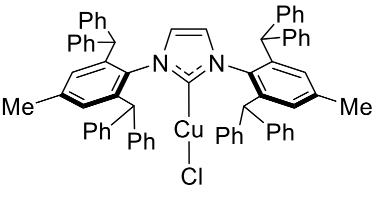

Synthesis of [(IPr*Me)CuCl]. In a nitrogen-filled glovebox, 7.00 g IPr*Me•HCl (7.37 mmol) and 1.05 g (7.37 mmol, 1 equiv) copper(I) oxide were combined in 150 mL toluene. The flask was brought out of the glovebox, attached to an N_2-purged reflux condenser, and heated at 100 °C overnight. After cooling, subsequent workup steps were performed in air. The reaction mixture was filtered through Celite, and the Celite pad was further washed with dichloromethane. The volatile materials were removed by rotary evaporation. The crude solid was vigorously stirred with 30 mL methanol for 30 min. The suspension was filtered, and the resulting off-white solid was washed with 50 mL hexanes, then dried under vacuum. Yield: 5.82 g (78%)

^{1}H NMR (500 MHz, C_6D_6): δ 7.25 (s, 4H), 7.19-7.13 (m, 20H, Ar-*H*), 7.00 (d, *J* = 7.5 Hz, 8H, Ar-*H*), 6.90-6.87 (m, 6H, Ar-*H*), 6.84 (s, 6H, Ar-*H*), 5.80 (s, 2H, *H*-C=C-*H*), 5.19 (s, 4H, C*H*(Ph)$_2$), 2.21 (s, 6H, C*H*$_3$). The ^{1}H NMR data are consistent with the literature.[6]

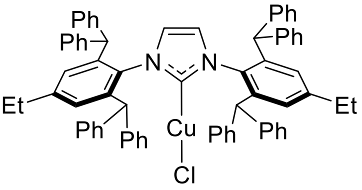

Synthesis of [(IPr*Et)CuCl]. In a glovebox, 2.17 g (2.22 mmol) IPr*Et•HCl and 325 mg (2.27 mmol) copper (I) oxide were combined in 50 mL toluene in a Schlenk flask. The flask was brought out of the glovebox, attached to an N_2-purged reflux condenser, and heated at 100 °C for 18 hours. Subsequent workup steps were performed in air. The reaction mixture was filtered through Celite, and the Celite pad was washed with another 50 mL of toluene. The solvent was removed from the filtrate via rotary evaporation, and the resulting brown solid was washed with toluene, giving an off-white solid. A second crop was collected by adding hexanes to the filtrate. The combined solids were dried under vacuum to afford the title compound as an off-white solid. Yield: 1.39 g (60%)

^{1}H NMR (500 MHz, $CDCl_3$): δ 7.23-7.09 (m, 24H, Ar-*H*), 7.01 (d, *J* = 6.9 Hz, 8H, Ar-*H*), 6.94-6.82 (m, 12H, Ar-*H*), 5.82 (s, 2H, *H*-C=C-*H*), 5.21 (s, 4H, C*H*Ph$_2$), 2.51 (q, *J* = 7.6 Hz, 4H, Ar-C*H*$_2$CH$_3$), 1.07 (t, *J* = 7.6 Hz, 6H, Ar-CH$_2$C*H*$_3$).

^{13}C{^{1}H} NMR (126 MHz, $CDCl_3$): δ 180.2 (carbene-Cu), 145.9, 143.1, 142.4, 140.8, 134.3, 129.5, 129.4, 128.9, 128.6, 128.4, 126.6, 123.2, 51.2, 28.7, 14.8.

Crystals suitable for XRD were grown at room temperature by layering a concentrated CH_2Cl_2 solution of [(IPr*Et)CuCl] with pentane.

Anal. Calcd. for $C_{71}H_{68}ClCuN_2$ C: 81.98; H: 5.81; N: 2.69; Found: C: 81.70; H: 5.79; N: 2.86

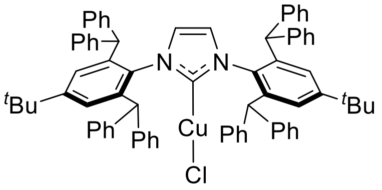

Synthesis of [(IPr*^{t}Bu)CuCl]. In an N_2-filled glovebox, 5.03 g (4.87 mmol) of IPr*^{t}Bu•HCl and 703 mg (4.91 mmol, 1 equiv) of copper(I) oxide were combined in 150 mL of toluene in a Schlenk flask. The Schlenk flask was brought out of the glovebox, attached to an N_2-purged reflux condenser, and heated at 100 °C under N_2. After 4 hours, the flask was cooled to room temperature. The reaction mixture was filtered through a Celite pad, and the Celite was washed with 100 mL of toluene. The solvent was removed from the filtrate under vacuum. The resulting brown solid was washed with toluene until the washes became colorless, giving a white solid. The white solid was further washed with 200 mL hexanes and dried under vacuum. Yield: 3.22 g (60%).

^{1}H NMR (500 MHz, CDCl$_3$, 25 °C): δ 7.25-7.15 (m, 24H, Ar-*H*), 7.08 (s, 4H, Ar-*H*), 7.02 (d, J = 7.0 Hz, 8H, Ar-*H*), 6.93-6.83 (m, 8H, Ar-*H*), 5.82 (s, 2H, *H*-C=C-*H*), 5.23 (s, 4H, C*H*Ph$_2$), 1.10 (s, 18H, (C*H*$_3$)$_3$C).

^{13}C{^{1}H} NMR (126 MHz, CDCl$_3$, 25 °C): δ 180.26 (carbene-Cu), 152.76, 143.41, 142.61, 140.48, 134.23, 129.65, 129.49, 128.71, 128.50, 126.76, 126.73, 126.70, 123.30, 51.57 (*C*HPh$_2$), 35.03, 31.10.

Crystals suitable for XRD were grown at room temperature by layering a concentrated CH_2Cl_2 solution of [(IPr*^{t}Bu)CuCl] with hexanes.

Anal. Calcd. for $C_{75}H_{68}ClCuN_2$ C: 82.16; H: 6.25; N: 2.56; Found: C: 81.89; H: 6.33; N: 2.71

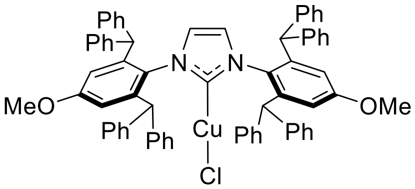

Synthesis of [(IPr*OMe)CuCl]. In a glovebox, 2.00 g (2.03 mmol) of IPr*OMe•HCl and 302 mg (2.11 mmol, 1.04 equiv) copper (I) oxide were combined in 50 mL of toluene in a Schlenk flask. The flask was brought out of the glovebox, attached to an N_2-purged reflux condenser, and heated at 100 °C for 4 hours. The flask was then cooled to room temperature, and the workup was performed in air. The reaction mixture was filtered through Celite and the Celite pad was washed with 100 mL toluene. The filtrate was concentrated to 50 mL on a rotary evaporator and 200 mL of hexanes was added, resulting in the immediate precipitation of an off-white solid. The precipitate was collected by filtration and dried under vacuum. Yield: 1.84 g (84%)

^{1}H NMR (500 MHz, C_6D_6): δ 7.24-7.10 (m, 24H, Ar-H), 7.03 (d, J = 7.1 Hz, 8H, Ar-*H*), 6.94-6.85 (m, 8H, Ar-*H*), 6.54 (s, 4H, Ar-*H*), 5.76 (s, 2H, *H*-C=C-*H*), 5.19 (s, 4H, C*H*Ph$_2$), 3.57 (s, 6H, Ar-OC*H*$_3$). The ^{1}H NMR data are consistent with the literature.[7]

Crystals suitable for XRD were grown at room temperature by slow diffusion of pentane into a concentrated CH_2Cl_2 solution of [(IPr*OMe)CuCl].

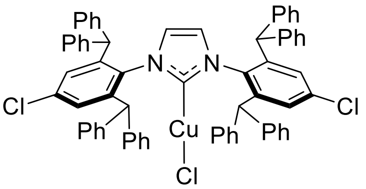

Synthesis of [(IPr*Cl)CuCl]. In a glovebox, 602 mg (0.608 mmol) IPr*Cl•HCl and 87.6 mg (0.612 mmol, 2 equiv Cu) copper(I) oxide were suspended in 15 mL of dry toluene in a Schlenk flask. The flask was brought out of the glovebox, attached to an N_2-purged reflux condenser, and heated at 100 °C for 20 hours. Subsequent workup steps were performed in air. The reaction mixture was cooled to room temperature and filtered through Celite to remove unreacted copper(I) oxide. The Celite pad was washed with 40 mL of toluene. The solvent was removed from the filtrate via rotary evaporation. The resulting material was washed with toluene (3x 5 mL) and dried under vacuum. A second crop was isolated by adding 100 mL hexanes to the filtrate, collecting the resulting off-white precipitate, and drying under vacuum. Yield: 398 mg (62%)

^{1}H NMR (500 MHz, 25 °C, CDCl$_3$): δ 7.24-7.13 (m, 24H, Ar-*H*), 7.04 (s, 4H, Ar-*H*), 6.98 (d, J = 7.2 Hz, 8H, Ar-*H*), 6.90-6.82 (m, 8H, Ar-*H*), 5.82 (s, 2H, H-C=C-H), 5.16 (s, 4H, C*H*Ph$_2$)

$^{13}C\{^{1}H\}$ NMR (126 MHz, $CDCl_3$): δ 180.4 (carbene-Cu), 143.3, 142.1, 141.2, 136.5, 134.9, 129.8, 129.4, 129.2, 128.9, 128.7, 127.1, 123.2, 51.3.

Crystals suitable for XRD were grown at room temperature by layering a concentrated dichloromethane solution of [(IPr*Cl)CuCl] with pentane.

Anal. Calcd. for $C_{67}H_{50}Cl_3CuN_2$ C: 76.42; H: 4.79; N: 2.66; Found: C: 76.20; H: 4.86: N: 2.74

XRD structures of [(IPr*R)CuCl]

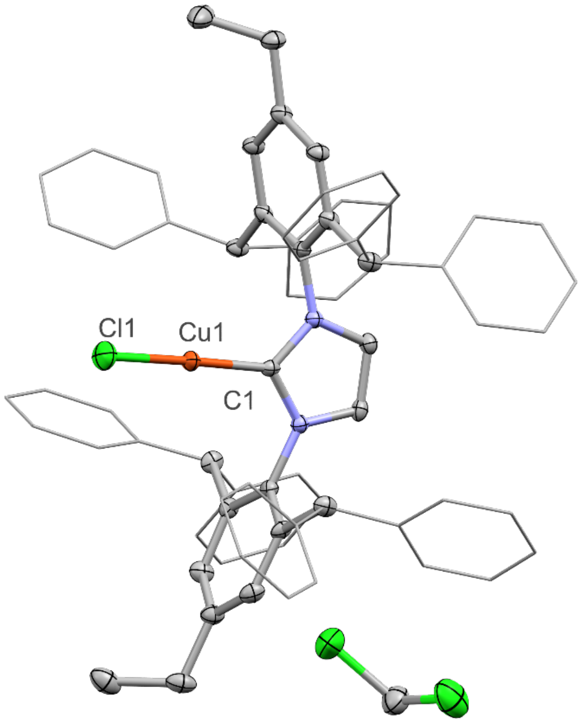

Figure S3. Thermal ellipsoid plot (50% probability) of [(IPr*Et)CuCl]•CH_2Cl_2. Hydrogen atoms are not shown, and the flanking phenyl groups are shown in wireframe representation.

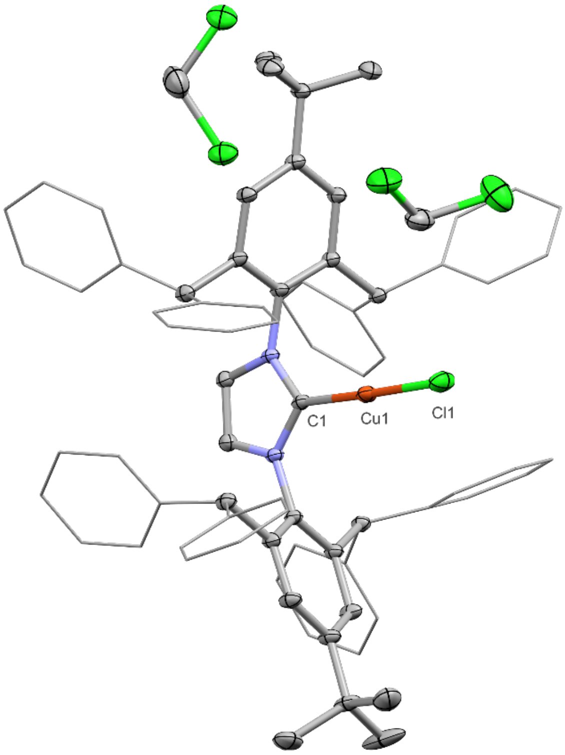

Figure S4. Thermal ellipsoid plot (50% probability) of [(IPr*^{t}Bu)CuCl]•2 CH_2Cl_2. Hydrogen atoms are not shown and the flanking phenyl groups are shown in wireframe representation.

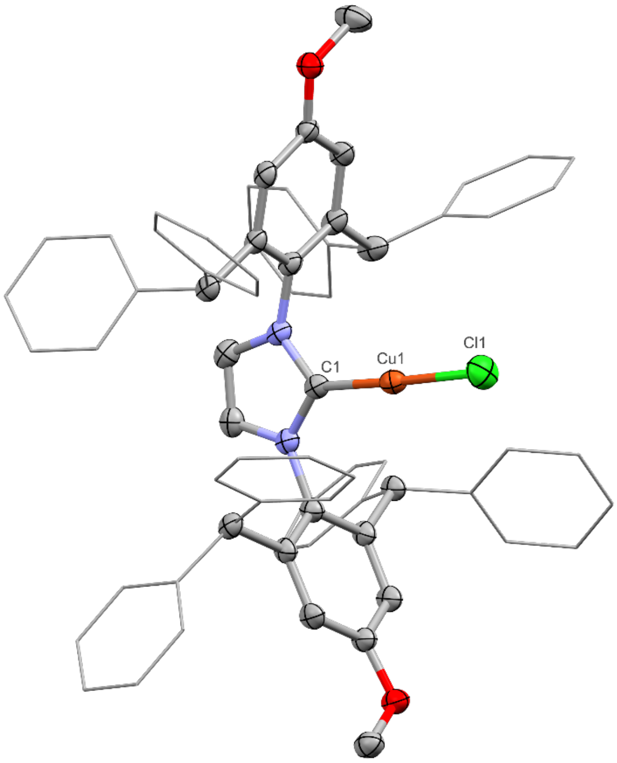

Figure S5. Thermal ellipsoid plot (50% probability) of [(IPr*OMe)CuCl]. Hydrogen atoms are not shown, and the flanking phenyl groups are shown in wireframe representation. The disorder of methoxy groups and flanking phenyl groups is not shown. A second molecule with identical connectivity is also not shown.

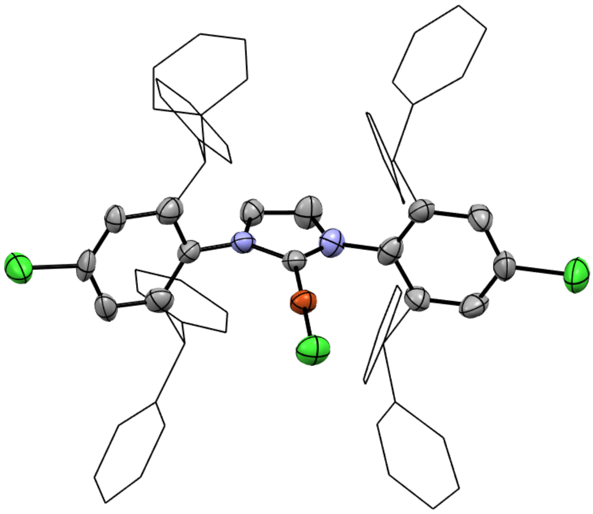

Figure S6. Thermal ellipsoid plot (50% probability) of [(IPr*Cl)CuCl]. Hydrogen atoms are not shown, the flanking phenyl groups are shown in wireframe representation, and the disorder of the flanking phenyl groups is not shown. The data for this complex was of poor quality, and the structure is presented to show connectivity, not as a representation of accurate bond lengths and angles.

^{1}H and $^{13}C\{^{1}H\}$ NMR spectra of [(IPr*R)CuCl]

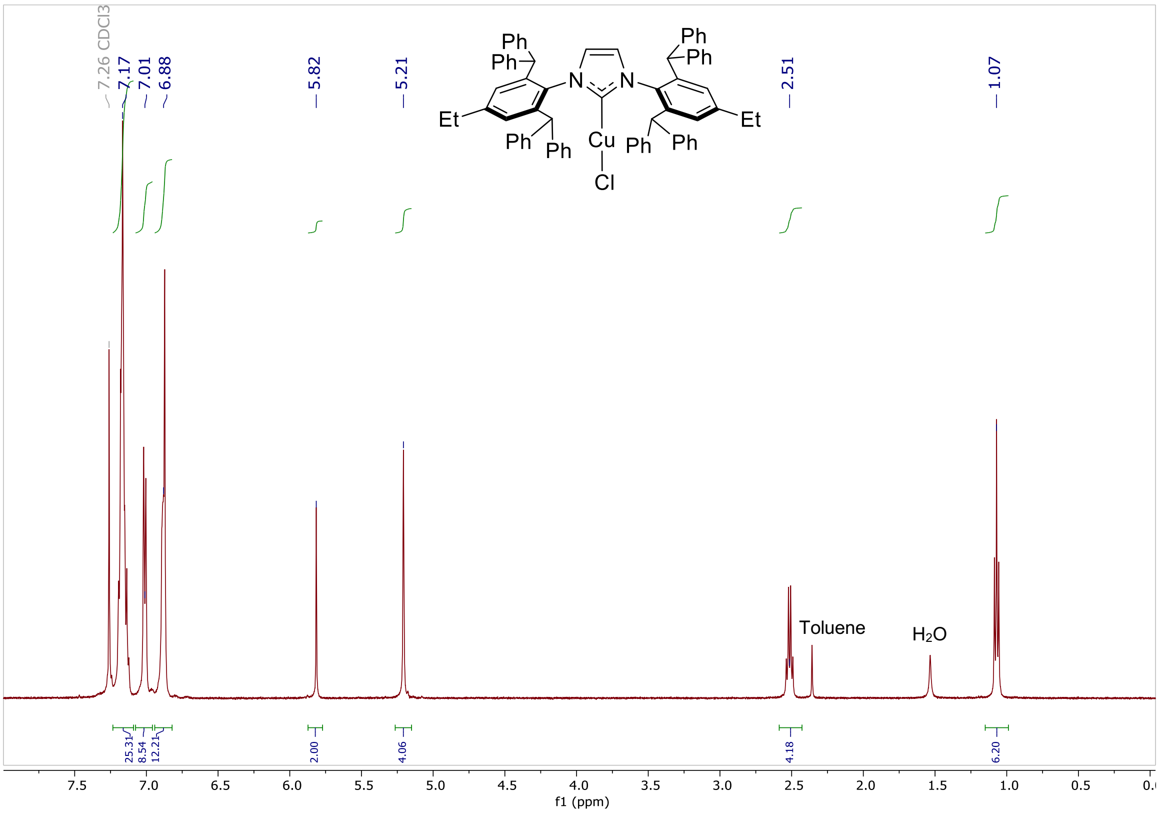

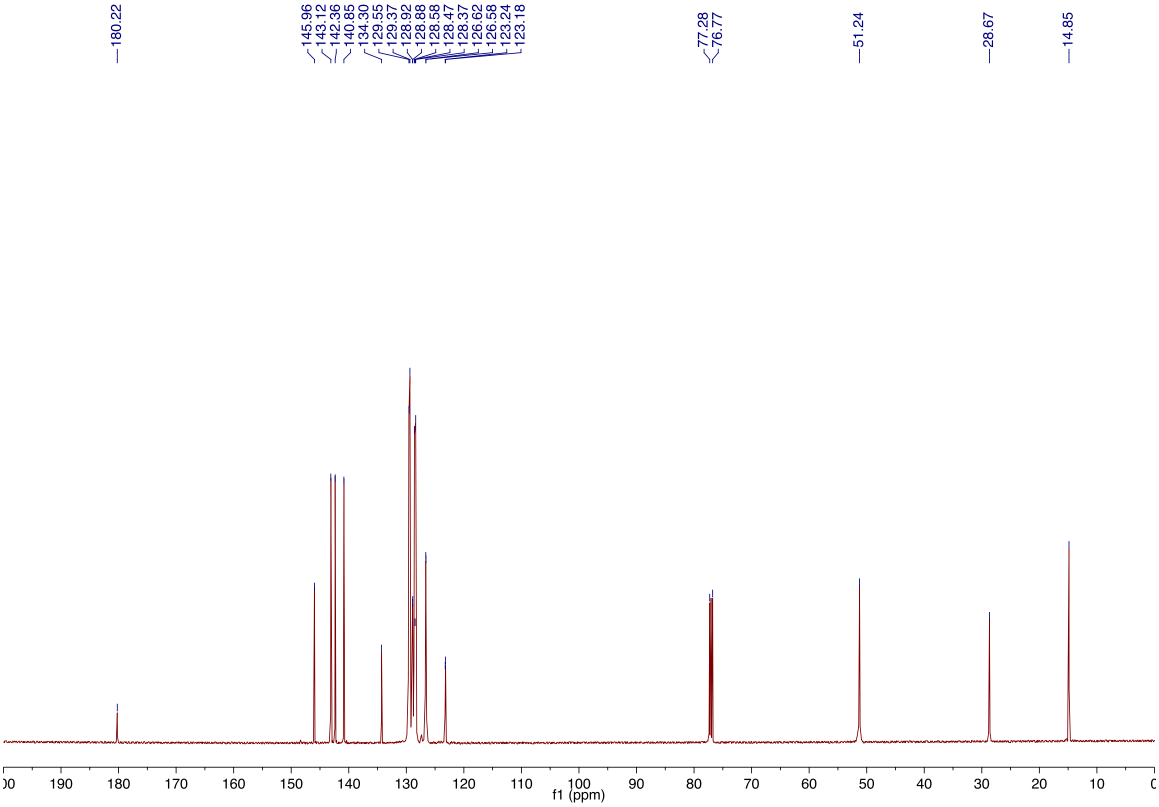

Figure S7. ^{1}H (top) and $^{13}C\{^{1}H\}$ (bottom) NMR spectra of [(IPr*Et)CuCl] in $CDCl_3$.

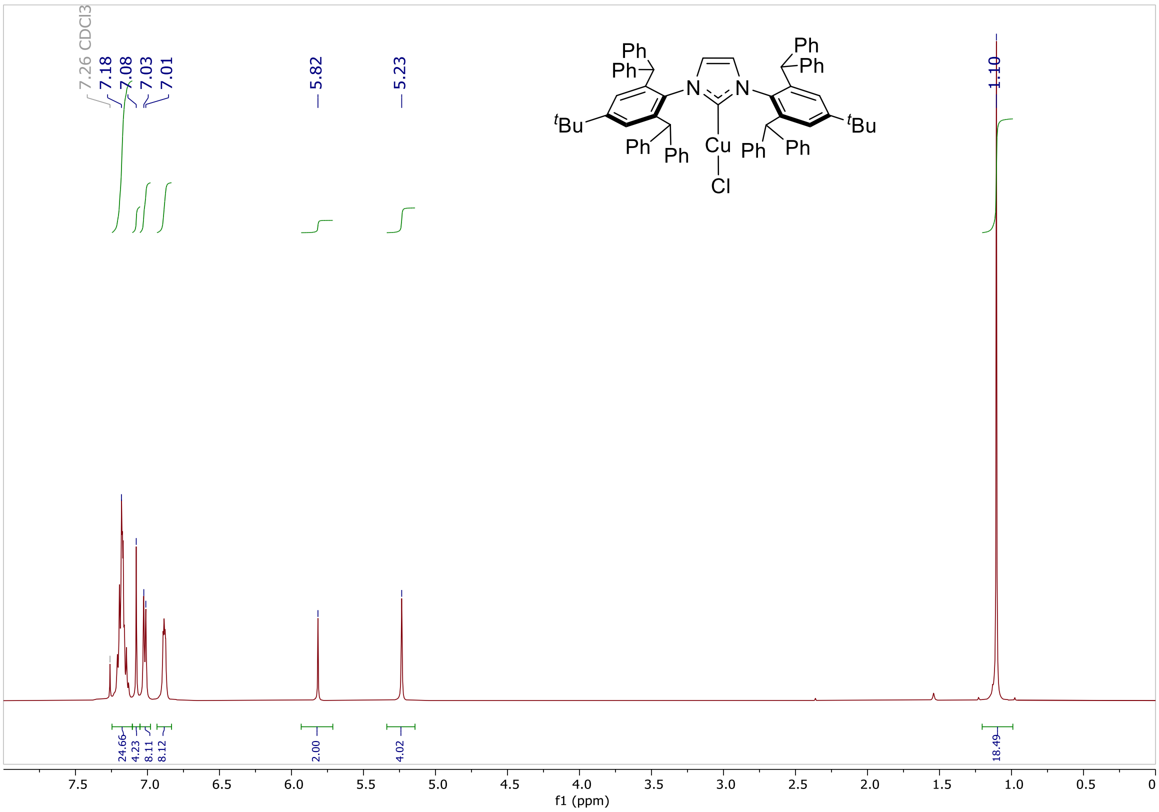

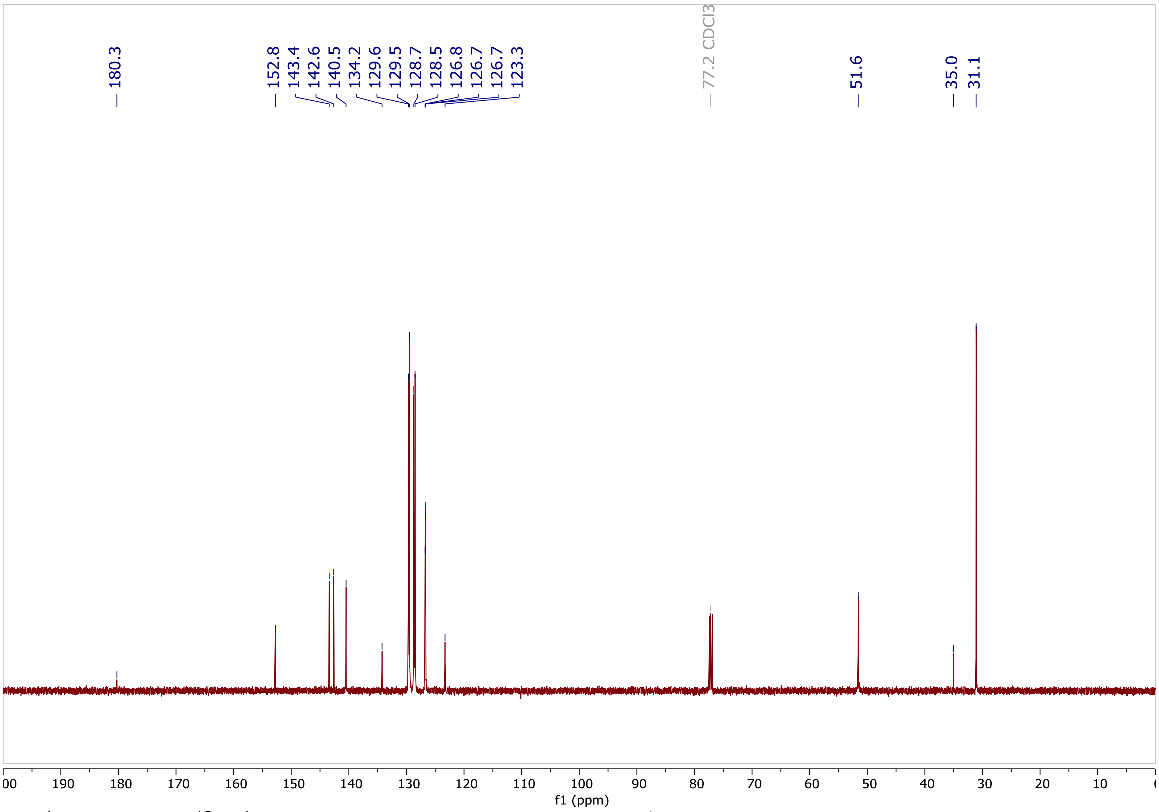

Figure S8. ^{1}H (top) and ^{13}C{^{1}H} (bottom) NMR spectra of [(IPr*^{t}Bu)CuCl] in $CDCl_3$.

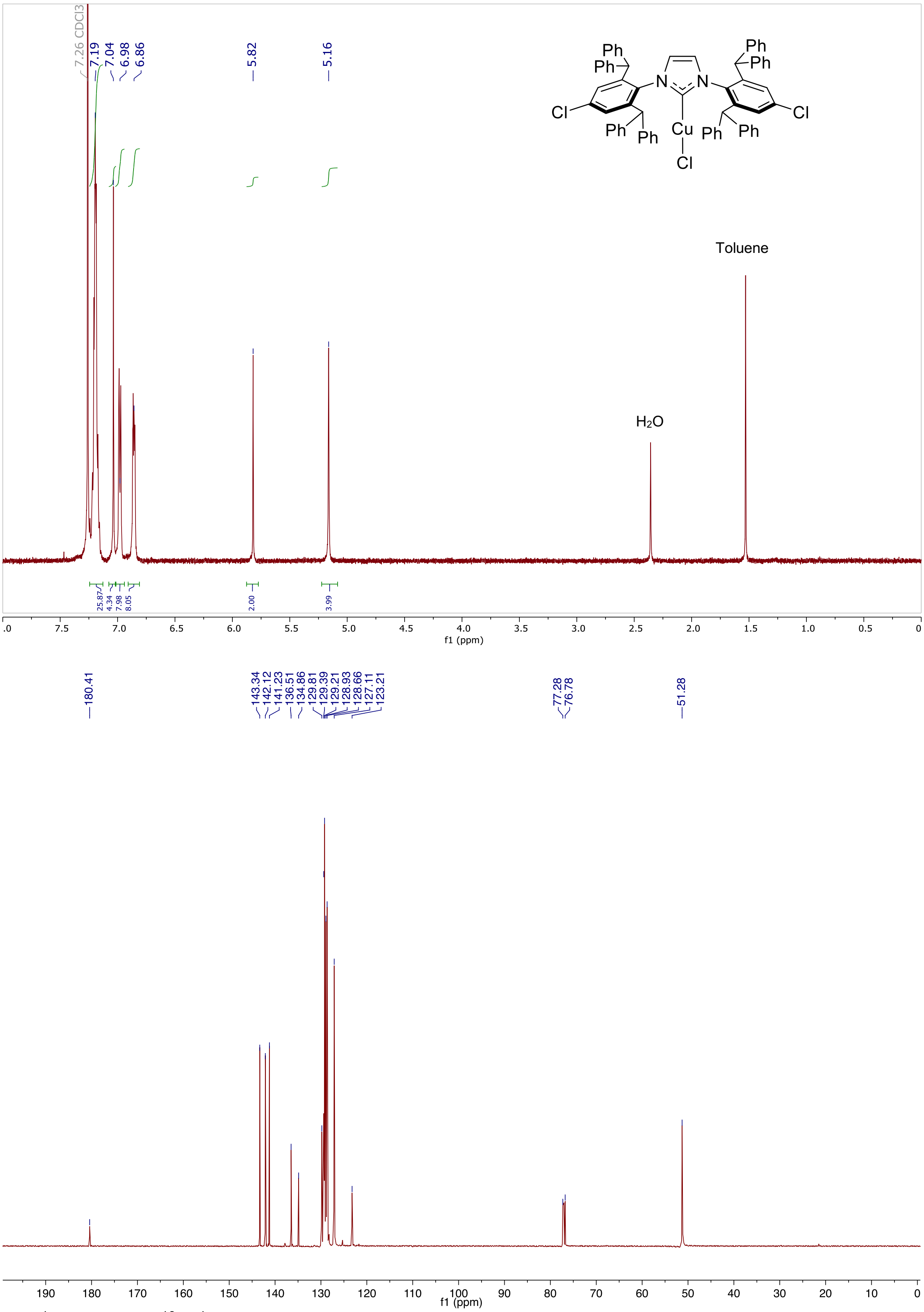

Figure S9. ^{1}H (top) and ^{13}C{^{1}H} (bottom) NMR spectra of [(IPr*Cl)CuCl] in $CDCl_3$.

Calculation of %V_{bur} and G

To quantify steric effects, we calculated %V_{bur} and G (solid angles) using the XRD structures of [(IPr*R)CuCl] (*R* = Cl, Et, OMe, ^{t}Bu) as well as the structure of [(IPr*Me)CuCl] from the literature.[8]

%V_{bur} was calculated with SambVca 2.1[9] (https://www.molnac.unisa.it/OMtools/sambvca2.1/index.html) using a 5.5 Å sphere radius with the metal-NHC bond length normalized to 2 Å and H atoms omitted. The results of these calculations are shown in Table S1. The calculations indicate ~58% %V_{bur} for R = Cl, Me, Et, and OMe with only a 1.3% increase along the series. For context, Nolan and co-workers suggest that a range of ~1.2% can be expected for different conformations of a flexible ligand.[10] A slightly higher value (%V_{bur} = 61.5%) is observed for R = ^{t}Bu. However, the steric maps (Figure S10) suggest that the changes may be largely due to minor differences in the orientation of the $CHPh_2$ substituents. Accordingly, when the $CHPh_2$ groups are removed and %V_{bur} is reevaluated, the values are identical within error, since the R-groups are not contained in the coordination sphere evaluated in the calculation. This finding also indicates that there is no significant change in the primary coordination sphere of Cu as a function of the remote steric bulk.

Solid angles (G) have been proposed as an alternative to %V_{bur} in situations where the steric bulk is further from the metal center.[11, 12] G values were calculated using the SolidG program (https://xray.chem.wisc.edu/solid-g/) with the metal-NHC bond length was normalized to 2 Å. The results of these calculations are shown in Table S1. Based on the difference in G calculated for the two crystallographically inequivalent molecules in the XRD structure of [(IPr*OMe)CuCl], changes of ~1.5% are likely insignificant. The values of G for IPr*Cl, IPr*Me, IPr*Et, and IPr*OMe span a very small range and G for IPr*Cl ≈ IPr*Me < IPr*OMe ≈ IPr*Et << IPr*^{t}Bu, as expected. The same trend in G is observed for models where the $CHPh_2$ groups are removed.

Overall, this analysis indicates that %V_{bur} and (to a lesser extent) G are significantly influenced by the orientations of the $CHPh_2$ groups, which are subject to influences from crystal packing. The calculated steric parameters are roughly the same within error. These methods are therefore not suitable for analysis of structures like the IPr*R ligands reported here that have only subtle structural differences far from the metal center and contain conformationally flexible groups.

Table S1. Comparison of %V_{bur} and G calculated with metal-ligand bond length normalized to 2 Å for IPr*R

	**Full Model**		**Truncated Model[a]**	
	**%V_{bur}**	**G**	**%V_{bur}**	**G**
**IPr*Cl**	57.6	57.6	27.7	31.7
**IPr*Me**	57.9	58.1	27.5	31.5
**IPr*Et**	58.2	59.5	27.8	33.2
**IPr*OMe A[b]**	58.9	60.5	28.0	32.6
**IPr*OMe B[b]**	58.5	59.1	27.7	32.1
**IPr*^{t}Bu**	61.5	64.0	27.9	33.9

[a] For model truncated by removing $CHPh_2$ groups [b] Values calculated for the two crystallographically inequivalent molecules in the XRD structure of [(IPr*OMe)CuCl].

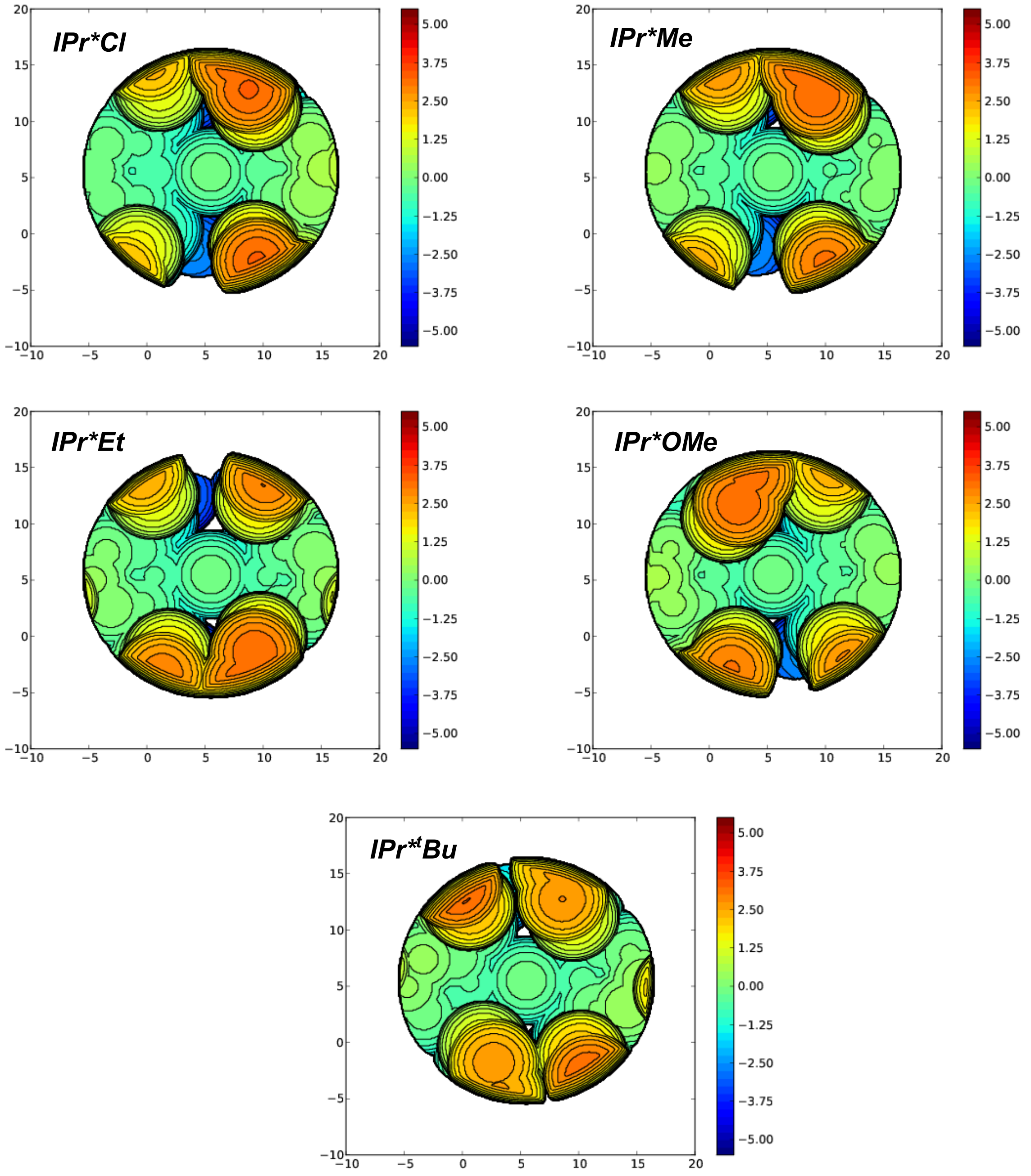

Figure S10. Steric maps for IPr*R calculated from the [(IPr*R)CuCl] XRD structures using SambVca 2.1 at 5.5 Å with the metal-ligand bond length normalized to 2 Å.

Synthesis and characterization of [(IPr*R)Rh(COD)Cl]

General Procedure for synthesis of [(IPr*R)Rh(COD)Cl] complexes

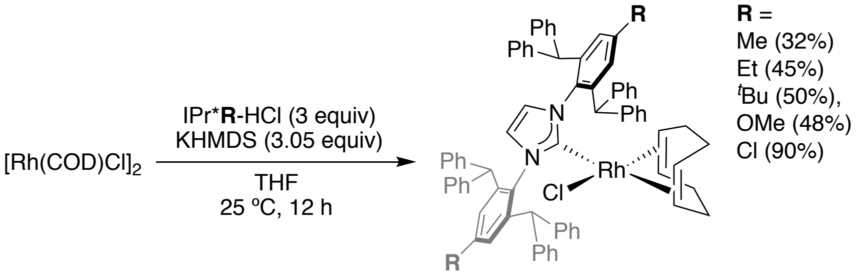

Inside a nitrogen-filled glovebox, KHMDS (122 mg, 0.610 mmol), IPr*R•HCl (0.60 mmol), a small stirring bar, and THF (10 mL) were added to a 20 mL scintillation vial. The reaction mixture was stirred at 25 ºC for 0.5 h to give a brownish yellow solution. This solution of free carbene was added to a separate 40 mL scintillation vial containing $[Rh(COD)Cl]_2$ (99.0 mg, 0.20 mmol) in 10 mL of THF to give an orange solution. The reaction was stirred overnight (12 h) at ambient temperature. The vial was removed from the glovebox and all subsequent workup steps were performed in air. All volatiles were removed via a rotary evaporator. Et_2O (10 mL) was added to the crude mixture, and all volatiles were again removed. The crude product was extracted into *ca.* 15 mL of dichloromethane and was filtered through a glass pipette containing a fiberglass plug and Celite. All volatiles were removed from the filtrate on a rotary evaporator. The crude product was transferred to a round-bottomed flask containing a stir bar, suspended in MeOH (10 mL), and warmed at 70 ºC for 15 minutes. The resulting yellow solid in the warm solution was collected on a medium porosity frit by vacuum filtration, washed with hexanes at room temperature (10 mL), and dried under vacuum.

Characterization of [(IPr*Me)Rh(cod)Cl]

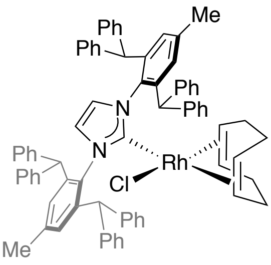

Yield = 32% based on $[Rh(COD)Cl]_2$. **^{1}H NMR** (500 MHz, 25 ºC, $CDCl_3$): δ 7.52 (d, *J* = 7.0 Hz ,4H, Ar-*H*), 7.21-6.97 (m, 34H), 6.93 (br s, 2H, Ar-H), 6.83 (br s, 2H, Ar-H), 6.74-6.69 (m, 6H, Ar-H), 5.32 (br s, 1H, vinylic cod C-H), 4.99 (m, 1H, vinylic cod C-H), 4.77 (s, 2H, C-H backbone), 3.58 (br s, 2H, vinylic cod C-H), 2.27 (s, 6H, Me), 2.10-2.03 (m, 2H, allylic cod C-H), 1.76-1.65 (m, 2H, allylic cod C-H), 1.55-1.48 (m, 4H, allylic cod C-H). The ^{1}H NMR data are consistent with those published in the literature.[2]

Characterization of [(IPr*Et)Rh(cod)Cl]

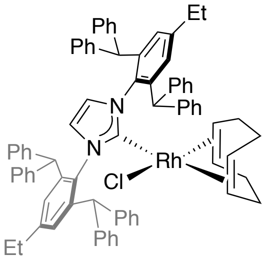

Yield = 50% based on $[Rh(COD)Cl]_2$. **^{1}H NMR** (500 MHz, 25 ºC, $CDCl_3$): δ 7.52 (d, *J* = 6.5 ,4H, Ar-*H*), 7.26-6.96 (m, 30H), 6.85-681(m, 2H, Ar-H), 6.72-6.66 (m, 10H, Ar-H), 6.57 (br s, 2H, Ar-H), 5.27 (br s, 1H, vinylic cod C-H), 4.96 (m, 1H, vinylic cod C-H), 4.74 (s, 2H, C-H backbone), 3.59 (br s, 2H, vinylic cod C-H), 2.56 (q, *J* = 7.5 Hz, 4H, C*H*$_2$Me), 2.05-2.00 (m, 2H, allylic cod C-H), 1.73-1.65 (m, 4H, allylic cod C-H), 1.56-1.49 (m, 2H, allylic cod C-H), 1.09 (t, *J* = 7.5 Hz, 6H, CH$_2$*Me*).

^{13}C{^{1}H} NMR (126 MHz, $CDCl_3$): δ 184.0 (d, *J* = 52.0 Hz, Rh-carbene) , 163.8, 144.9, 144.3, 144.2, 144.1, 143.8, 143.2, 140.5, 136.3, 130.9, 130.2, 129.7, 129.5, 129.2, 128.2, 128.0, 127.9, 127.6, 126.3, 125.8, 123.6, 123.5, 96.5, 68.9 (d, *J* = 14.0 Hz, vinylic C-H cod), 51.5 (*C*HPh$_2$), 50.8 (*C*HPh$_2$), 32.4 (allylic CH$_2$ cod), 28.8 (allylic CH$_2$ cod), 28.3, 15.6.

Characterization of [(IPr*OMe)Rh(cod)Cl]

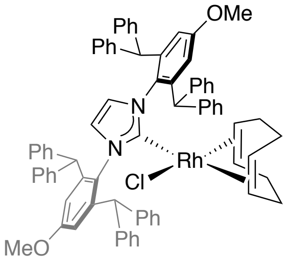

Yield = 48% based on $[Rh(COD)Cl]_2$. **^{1}H NMR** (500 MHz, 25 ºC, $CDCl_3$): δ 7.53 (d, *J* = 6.5 ,4H, Ar-*H*), 7.26-6.99 (m, 30H), 6.82 (br s, 2H, Ar-H), 6.76-6.66 (m, 10H, Ar-H), 6.57 (br s, 2H, Ar-H), 5.26 (br s, 1H, vinylic cod C-H), 4.98 (m, 1H, vinylic cod C-H), 4.70 (s, 2H, C-H backbone), 3.64 (br s, 2H, vinylic cod C-H), 3.61 (s, 6H, OMe), 2.10-2.03 (m, 2H, allylic cod C-H), 1.76-1.65 (m, 4H, allylic cod C-H), 1.55-1.46 (m, 2H, allylic cod C-H).

^{13}C{^{1}H} NMR (126 MHz, $CDCl_3$): δ 184.3 (d, *J* = 51.9 Hz, Rh-carbene), 158.7, 146.0, 144.5, 144.0, 143.9, 142.9, 142.3, 131.8, 130.8, 130.1, 129.6, 129.1, 128.1, 127.9, 127.7, 126.6, 126.45, 125.97, 125.8, 123.7, 123.6, 115.3, 114.3, 96.6, 68.9 (d, *J* = 14.1 Hz, vinylic C-H cod), 55.1 (O*Me*), 51.7 (*C*HPh_2), 50.9 (*C*HPh_2), 32.5 (allylic CH_2 cod), 28.4 (allylic CH_2 cod).

Characterization of [(IPr*^{t}Bu)Rh(cod)Cl]

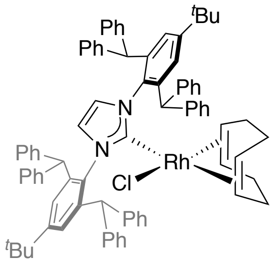

Yield = 50% based on $[Rh(COD)Cl]_2$. **^{1}H NMR** (500 MHz, 25 ºC, $CDCl_3$): δ 7.51 (d, *J* = 6.5, 4H, Ar-*H*), 7.24-7.01 (m, 32H), 6.94 (br s, 4H, Ar-H), 6.75 (m, 4H, Ar-H), 6.65 (br s, 4H, Ar-H), 5.19 (br s, 1H, vinylic cod C-H), 4.91 (m, 1H, vinylic cod C-H), 4.71 (s, 2H, C-H backbone), 3.58 (br s, 2H, vinylic cod C-H), 1.98-1.91 (m, 2H, allylic cod C-H), 1.67-1.59 (m, 4H, allylic cod C-H), 1.55-1.46 (m, 2H, allylic cod C-H). 1.14 (s, 18H, ^{t}Bu).

^{13}C{^{1}H} NMR (126 MHz, $CDCl_3$): δ 184.3 (d, *J* = 51.7 Hz, Rh-carbene), 150.9, 145.0, 144.6, 144.2, 143.9, 143.2, 139.9, 135.9, 130.8, 130.1, 129.7, 129.1, 128.0, 127.8, 127.5, 127.2, 126.5, 126.3, 125.8, 125.7, 125.6, 125.5, 123.5, 123.4, 96.4, 68.6 (d, *J* = 14.0 Hz, vinylic C-H cod), 51.8 (*C*HPh_2), 50.9 (*C*HPh_2), 32.4 (allylic CH_2 cod), 31.0 (^{t}Bu), 28.3 (allylic CH_2 cod).

Anal. Calcd for $C_{83}H_{80}ClN_2Rh$: C, 80.14; H, 6.48; N, 2.25. Found: C, 80.05; H, 6.62; N, 2.33.

Characterization of [(IPr*Cl)Rh(cod)Cl]

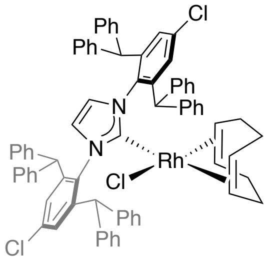

Yield = 90% based on $[Rh(COD)Cl]_2$. **^{1}H NMR** (500 MHz, 25 ºC, $CDCl_3$): δ 7.51 (d, *J* = 6.5 ,4H, Ar-*H*), 7.28-7.00 (m, 34H), 6.77 (br s, 2H, Ar-H), 6.83 (br s, 2H, Ar-H), 6.69-6.85 (m, 6H, Ar-H), 5.29 (br s, 1H, vinylic cod C-H), 5.04 (m, 1H, vinylic cod C-H), 4.73 (s, 2H, C-H backbone), 3.55 (br s, 2H, vinylic cod C-H), 2.13-2.06 (m, 2H, allylic cod C-H), 1.81-1.72 (m, 2H, allylic cod C-H), 1.60-1.48 (m, 4H, allylic cod C-H).

^{13}C{^{1}H} NMR (126 MHz, $CDCl_3$): δ 184.5 (d, *J* = 51.7 Hz, Rh-carbene), 146.6, 144.0, 143.3, 143.1, 142.9, 142.1, 137.1 (Ar), 134.8 (Ar), 130.7 (*C*HPh_2), 130.2 (*C*HPh_2), 129.9 (*C*HPh_2), 129.5, 128.9, 128.7, 128.3, 128.0, 127.0, 126.8, 126.3, 126.1, 123.6, 123.5, 97.7 (vinylic C-H cod), 69.3 (d, *J* = 14.0 Hz, vinylic C-H cod), 51.5 (*C*HPh_2), 50.9 (*C*HPh_2), 32.5 (allylic CH_2 cod), 28.4 (allylic CH_2 cod).

Anal. Calcd for $C_{75}H_{62}Cl_3N_2Rh$: C, 75.03; H, 5.21; N, 2.33. Found: C, 74.81; H, 5.21; N, 2.50.

^{1}H and ^{13}C{^{1}H} NMR spectra of [(IPr*R)Rh(COD)Cl]

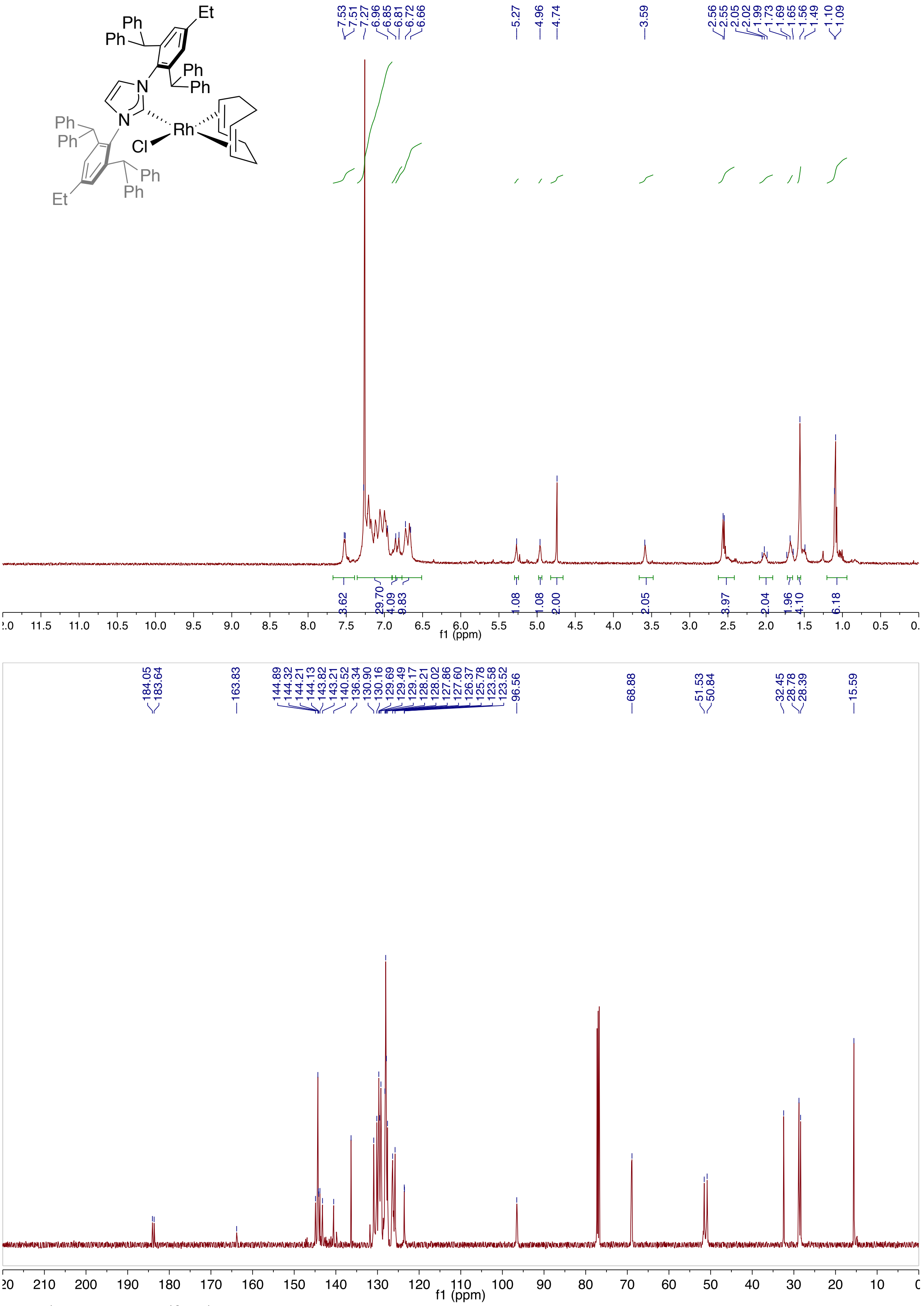

Figure S11. ^{1}H (top) and ^{13}C{^{1}H} (bottom) NMR spectra of [(IPr*Et)Rh(COD)Cl] in $CDCl_3$.

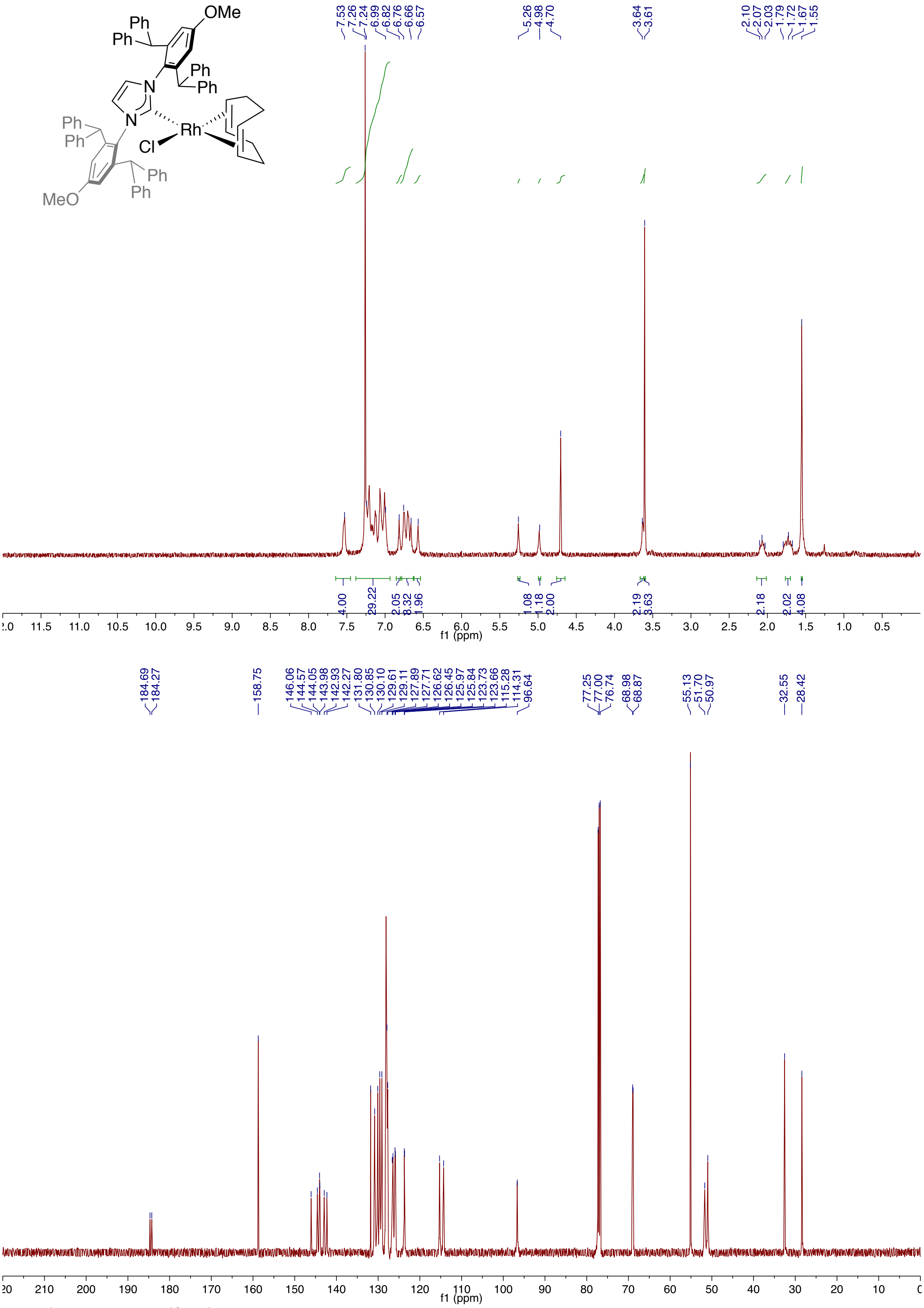

Figure S12. ^{1}H (top) and $^{13}C\{^{1}H\}$ (bottom) NMR spectra of [(IPr*OMe)Rh(COD)Cl] in $CDCl_3$.

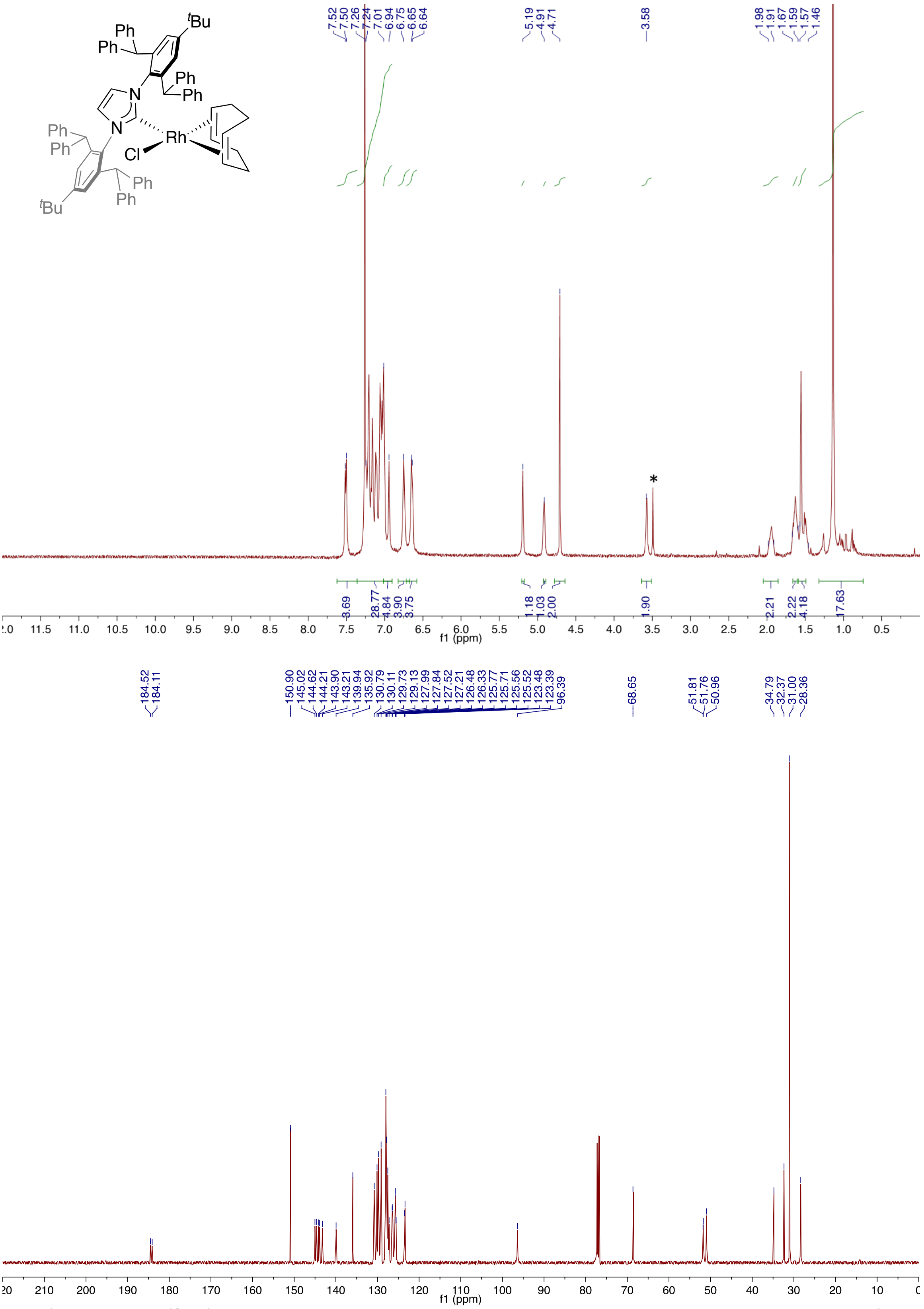

Figure S13. ^{1}H (top) and ^{13}C{^{1}H} (bottom) NMR spectra of [(IPr*^{t}Bu)Rh(COD)Cl] in $CDCl_3$. *MeOH in ^{1}H NMR spectrum

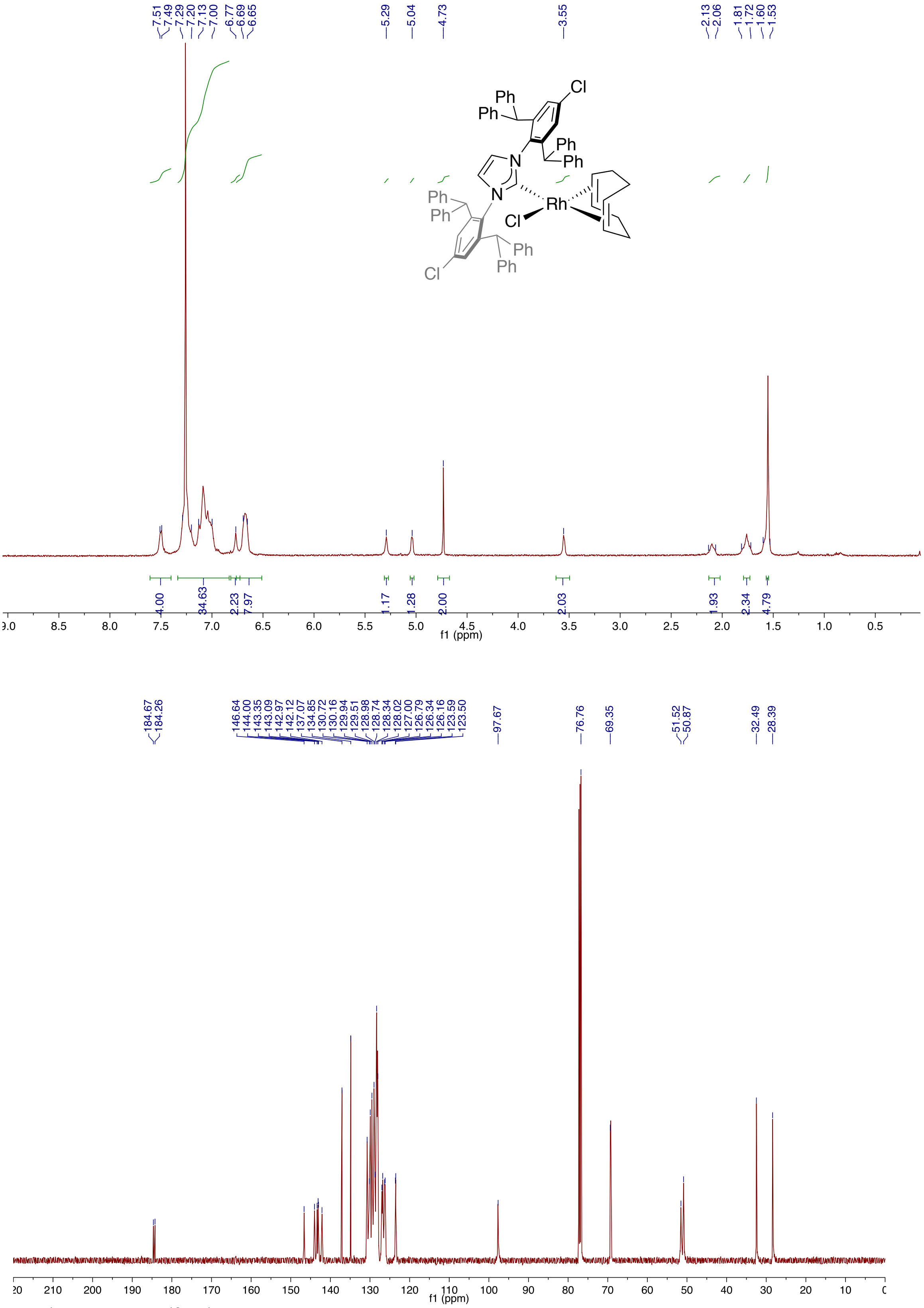

Figure S14. ^{1}H (top) and $^{13}C\{^{1}H\}$ (bottom) NMR spectra of [(IPr*Cl)Rh(COD)Cl] in $CDCl_3$.

Cyclic Voltammograms of [(IPr*R)Rh(COD)Cl]

Cyclic voltammograms were recorded on ~4 mM solutions of [(IPr*R)Rh(COD)Cl] in CH_2Cl_2 containing 0.1 mM NBu_4PF_6 as supporting electrolyte. Measurements were performed on a CH Instruments 700D potentiostat using a standard three-electrode cell consisting of a glassy carbon working electrode, glassy carbon rod counter electrode, and silver wire pseudo-reference electrode. The potentials are reported versus $Cp_2Fe^{0/+}$.

Table S2. Comparison of ratio of anodic to cathodic current and peak-to-peak separation of ferrocene and [(IPr*R)Rh(COD)Cl] (scan rate = 100 mV/s).

	**IPr*Cl**	**IPr*Me**	**IPr*Et**	**IPr*OMe**	**IPr*^{t}Bu**
**Ferrocene i_{pa}/i_{pc}**	1.03	1.05	0.969	1.15	1.11
**Rh i_{pa}/i_{pc}**	1.04	1.16	1.45	0.985	0.961
**Ferrocene ΔE_p (mV)**	107	104	107	106	113
**Rh ΔE_p (mV)**	140	132	146	134	137
**$Rh^{I/II}$ (mV vs $Fc^{0/+}$)**	588	475	481	472	469

Table S3. Comparison of ratio of anodic to cathodic current and peak-to-peak separation of ferrocene and [(IPr*R)Rh(COD)Cl] (scan rate = 250 mV/s).

	**IPr*Cl**	**IPr*Me**	**IPr*Et**	**IPr*OMe**	**IPr*^{t}Bu**
**Ferrocene i_{pa}/i_{pc}**	1.02	1.11	1.09	1.16	1.13
**Rh i_{pa}/i_{pc}**	1.11	1.12	1.56	1.04	0.99
**Ferrocene ΔE_p (mV)**	128	132	128	125	136
**Rh ΔE_p (mV)**	174	172	193	168	173
**$Rh^{I/II}$ (mV vs $Fc^{0/+}$)**	593	478	482	475	474

Table S4. Comparison of ratio of anodic to cathodic current and peak-to-peak separation of ferrocene and [(IPr*R)Rh(COD)Cl] (scan rate = 500 mV/s).

	**IPr*Cl**	**IPr*Me**	**IPr*Et**	**IPr*OMe**	**IPr*^{t}Bu**
**Ferrocene i_{pa}/i_{pc}**	1.08	1.13	1.10	1.19	1.15
**Rh i_{pa}/i_{pc}**	1.23	1.16	1.85	1.12	1.06
**Ferrocene ΔE_p (mV)**	156	163	157	149	162
**Rh ΔE_p (mV)**	213	215	236	206	210
**$Rh^{I/II}$ (mV vs $Fc^{0/+}$)**	598	483	488	478	478

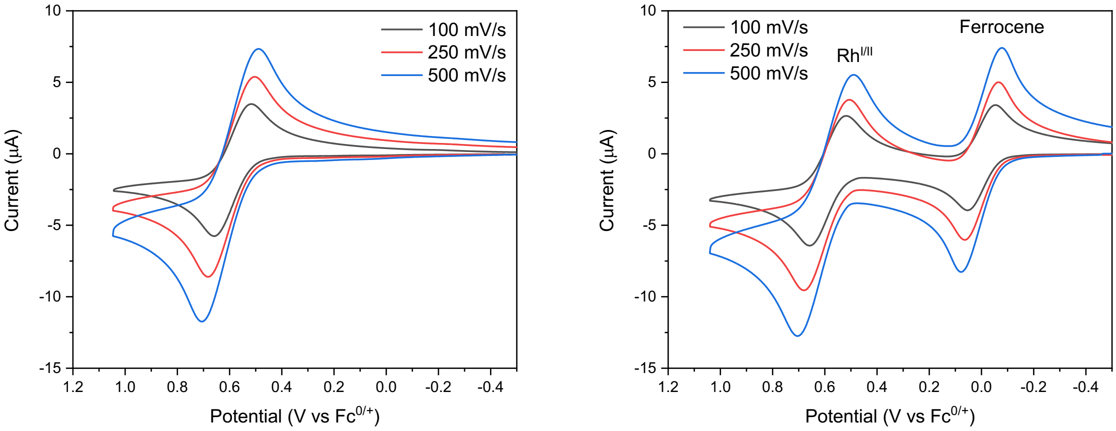

Figure S15. Cyclic voltammograms of [(IPr*Cl)Rh(COD)Cl] without (left) and with (right) ferrocene internal standard.

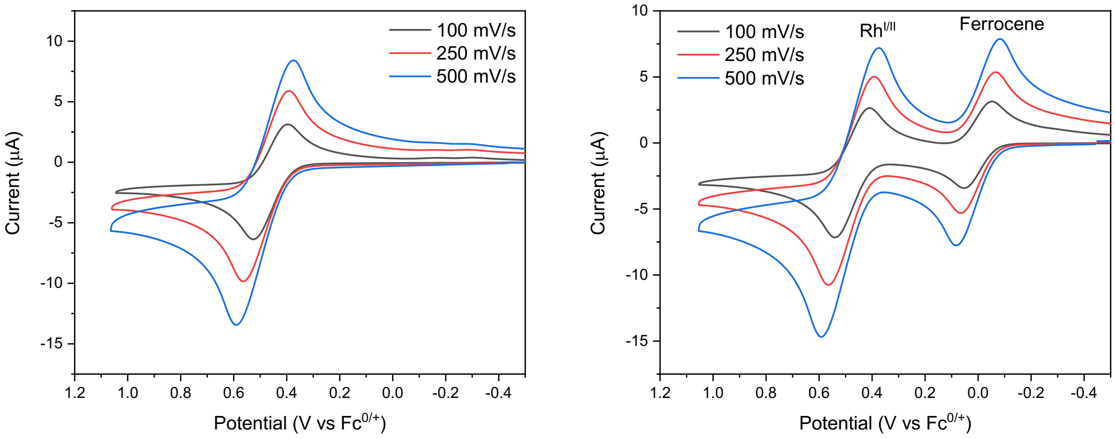

Figure S16. Cyclic voltammograms of [(IPr*Me)Rh(COD)Cl] without (left) and with (right) ferrocene internal standard.

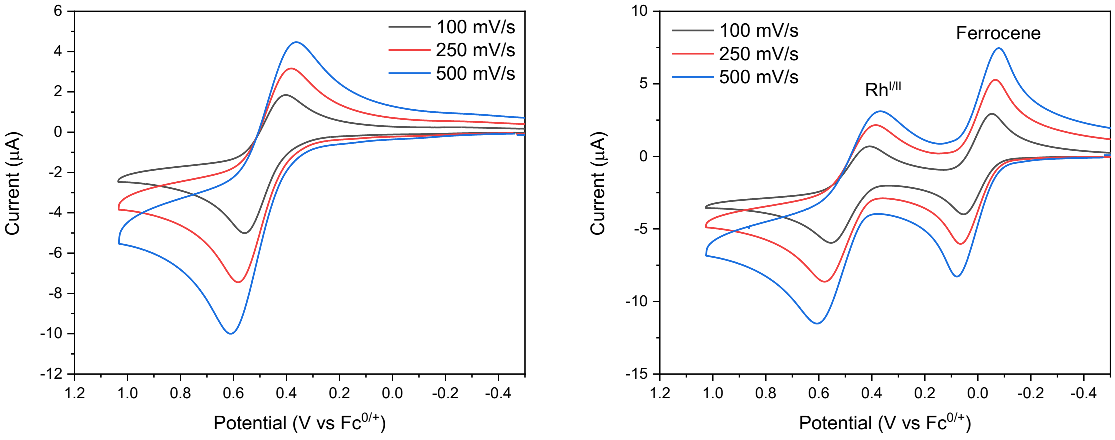

Figure S17. Cyclic voltammograms of [(IPr*Et)Rh(COD)Cl] without (left) and with (right) ferrocene internal standard.

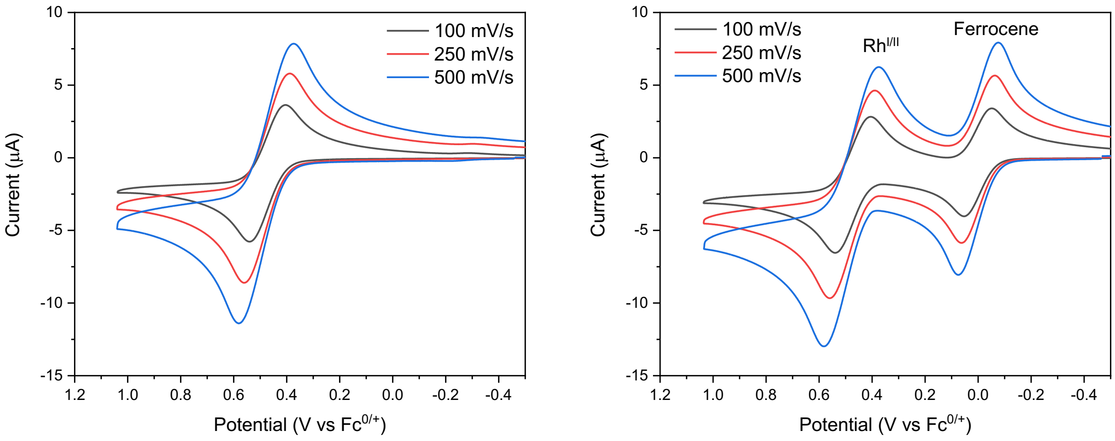

Figure S18. Cyclic voltammograms of [(IPr*OMe)Rh(COD)Cl] without (left) and with (right) ferrocene internal standard.

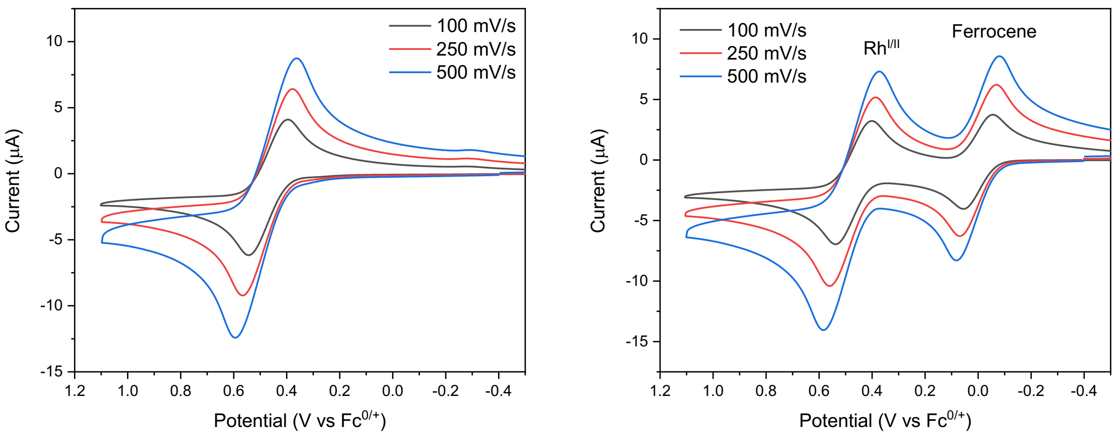

Figure S19. Cyclic voltammograms of [(IPr*tBu)Rh(COD)Cl] without (left) and with (right) ferrocene internal standard.

Synthesis and characterization of copper hydride complexes

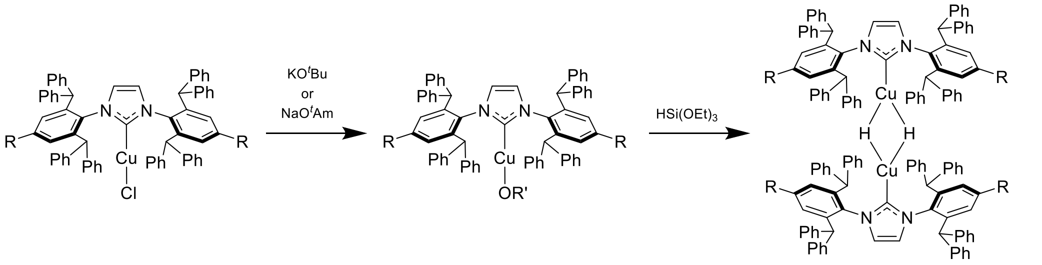

Yields:
R = Et (72%)
R = ^{t}Bu (68% yield over 2 steps)
R = OMe (80%)
R = Cl (83%)

General Notes

[(IPr*Me)CuH]$_2$ was prepared as previously described.[13] With the exception of [(IPr*^{t}Bu)CuH]$_2$, which was prepared from isolated [(IPr*^{t}Bu)Cu(O^{t}Bu)], all [(IPr*R)CuH]$_2$ were synthesized by addition of HSi(OEt)$_3$ to Cu alkoxide complexes generated *in situ*. The copper hydride complexes are extremely sensitive to air and moisture, which prevents thorough drying and recrystallization. We have therefore not attempted to obtain elemental analysis data. The purity of the copper hydrides is estimated at ~90-95% based on ^{1}H NMR spectroscopy.

During attempts at recrystallization of [(IPr*R)CuH]$_2$ or upon storage of isolated material for more than a few weeks, even in a glovebox freezer at −30 °C, [(IPr*R)Cu(OH)] was observed as a major decomposition product. The Cu-OH complexes can be identified using ^{1}H NMR spectroscopy. Their assignment is based on comparison to [(IPr*Me)Cu(OH)], which has been reported in the literature.[14] We also describe the isolation of [(IPr*OMe)Cu(OH)] below. In the Cu-OH spectra, characteristic singlets appear in between the resonances corresponding to C<u>*H*</u>Ph$_2$ and <u>*H*</u>-C=C-<u>*H*</u> in the corresponding copper hydrides. A singlet corresponding to the Cu-O<u>*H*</u> is also present at approximately −0.5 ppm but is broadened beyond detection in some cases. We have not isolated and fully characterized all of the Cu-OH complexes, but the key resonances described above are provided for each complex to allow identification of this common impurity.

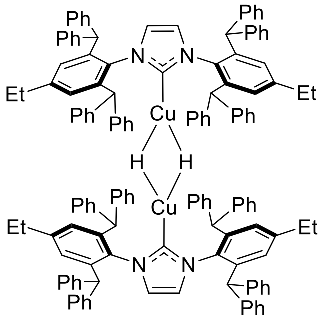

Synthesis of [(IPr*Et)CuH]$_2$. In a glovebox, 100 mg (0.096 mmol) of [(IPr*Et)CuCl] was dissolved in 2 mL toluene and 30 μL (0.10 mmol, 1.04 equiv) of 40% sodium tert-pentoxide in toluene was added. After 2 hours, the suspension was filtered through Celite. The filtrate was concentrated to 2 mL, and 30 μL (0.16 mmol. 1.7 equiv) triethoxysilane was added, resulting in rapid formation of an orange solution. After 5 minutes, 10 mL of pentane was added causing precipitation of a yellow solid. The solid was isolated by vacuum filtration, washed with pentane and dried under vacuum. Yield: 70 mg (72%)

Yellow crystals suitable for XRD were grown by layering a concentrated THF solution of [(IPr*Et)CuH]$_2$ in the presence of excess (~3 equiv) HSi(OEt)$_3$ with hexanes at room temperature.

^{1}H NMR (500 MHz, C$_6$D$_6$): δ 7.66 (d, *J* = 6.0 Hz, 8H, Ar-*H*), 7.10 (s, 4H, Ar-*H*), 7.02-6.83 (m, 32H, Ar-*H*), 6.05 (s, 4H, C*H*Ph$_2$), 5.27 (s, 2H, H-C=C-H), 3.83 (s, 2H, Cu-*H*), 2.19 (q, *J* = 7.6 Hz, 8H, Ar-*CH$_2$*CH$_3$), 0.99 (t, *J* = 7.6 Hz, 12H, Ar-CH$_2$*CH$_3$*).

^{13}C NMR (126 MHz, C$_6$D$_6$): δ 194.27 (carbene-Cu), 144.79, 144.37, 141.94, 136.93, 130.99, 130.93, 129.94, 129.00, 126.33, 126.27, 122.05, 121.96, 51.82, 51.79, 28.78, 15.11.

Key ^{1}H NMR resonances of [(IPr*Et)Cu(OH)] (500 MHz, C$_6$D$_6$): δ 5.71 (s, 4H, C*H*Ph$_2$), 5.58 (s, 2H, *H*-C=C-*H*), -0.47 (br s, 1H, Cu-O*H*).

UV-Vis (toluene, 25 °C): λ = 374 nm (ε = 8,000 M^{-1}cm^{-1}), 433 nm (ε = 9,600 M^{-1}cm^{-1}), 455 nm (ε = 8,500 M^{-1}cm^{-1})

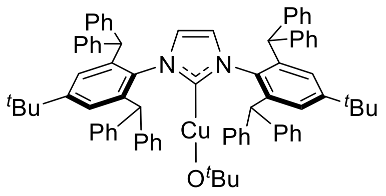

Synthesis of [Cu(IPr*^{t}Bu)(O^{t}Bu)]. In an N_2-filled glovebox, a solution of 500 mg (0.46 mmol) [Cu(IPr*^{t}Bu)(Cl)] in 10 mL THF was treated with a solution of 54 mg (0.48 mmol, 1.05 equiv) potassium tert-butoxide in 5 mL THF. After 1 hour, the solvent was removed under vacuum. The resulting residue was redissolved in toluene and filtered through Celite. The filtrate was concentrated to 5 mL, and 80 mL pentane was added. This solution was stored in a –30 °C freezer overnight, resulting in the precipitation of a white solid. The solid was collected on a fritted glass funnel, washed with 3x10 mL pentane, and dried under vacuum. Yield: 440 mg (85%).

^{1}H NMR (500 MHz, C_6D_6): δ 7.56 (d, *J* = 7.7 Hz, 8H, Ar-*H*), 7.34 (s, 4H, Ar-*H*), 7.26 (t, *J* = 7.6 Hz, 8H, Ar-*H*), 7.10-6.92 (m, 24H, Ar-*H*), 5.74 (s, 4H, C*H*Ph$_2$), 5.48 (s, 2H, *H*-C=C-*H*), 1.44 (s, 9H, -O(C*H*$_3$)$_3$), 0.99 (s, 18H, -C(C*H*$_3$)$_3$).

^{13}C{^{1}H} NMR (126 MHz, C_6D_6): δ 183.93 (carbene-Cu), 152.88, 143.97, 143.74, 141.25, 135.39, 130.43, 130.37, 129.85, 129.03, 128.64, 127.11, 127.09, 127.01, 126.97, 126.94, 126.75, 122.87, 122.78, 52.13, 52.08, 37.46, 34.95, 30.98.

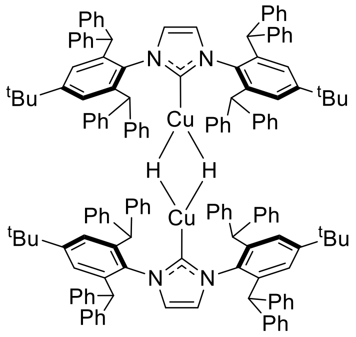

Synthesis of [(IPr*^{t}Bu)CuH]$_2$. A slurry of 202 mg (0.18 mmol) [(IPr*^{t}Bu)Cu(O^{t}Bu)] in 5 mL diethyl ether was treated with 50 mmol (0.27 mmol, 1.5 equiv) HSi(OEt)$_3$ causing formation of a homogeneous orange solution. The solution was filtered through a fiberglass plug, and the volatile materials were removed under vacuum. Upon addition of 10 mL pentane, a yellow solid precipitated. The solid was isolated by vacuum filtration, washed with 3 x 3 mL pentane, and dried under vacuum to afford 160 mg of crude [(IPr*^{t}Bu)CuH]$_2$ (approximately 80% yield) as a yellow solid. Based on ^{1}H NMR spectroscopy, this material is 90-95% pure, and contains pentane as well as other minor unknown impurities. We have been unable to fully purify [(IPr*^{t}Bu)CuH]$_2$ due to its thermal, moisture, and light sensitivity (see Figure S29).

Orange crystals suitable for XRD were grown in a –30 °C freezer by vapor diffusion of pentane into a concentrated THF solution of [(IPr*^{t}Bu)CuH]$_2$ in the presence of excess (~3 equiv) HSi(OEt)$_3$.

^{1}H NMR (500 MHz, C_6D_6): δ 7.65 (d, *J* = 7.4 Hz, 16H, Ar-*H*), 7.56 (s, 8H, Ar-*H*), 6.99-6.87 (m, 40H, Ar-*H*), 6.78 (t, *J* = 7.2 Hz, 16H, Ar-*H*), 6.71 (t, *J* = 7.1 Hz, 8H, Ar-*H*), 5.98 (s, 8H, C*H*Ph$_2$), 5.40 (s, 4H, *H*-C=C-*H*), 4.16 (s, 2H, Cu-*H*), 1.29 (s, 36H, -C(C*H*$_3$)$_3$).

^{13}C{^{1}H} NMR (126 MHz, $CDCl_3$): δ 192.66 (carbene-Cu), 151.30, 144.75, 143.79, 141.61, 137.13, 130.72, 130.67, 129.85, 128.99, 126.69, 126.64, 126.35, 126.30, 122.73, 122.66, 51.80 (*C*HPh$_2$), 35.22, 31.48.

Key ^{1}H NMR resonances of [(IPr*^{t}Bu)Cu-OH] (500 MHz, C_6D_6): δ 5.75 (s, 4H, C*H*Ph$_2$), 5.61 (s, 2H, *H*-C=C-*H*), 0.96 (s, 18H, Ar-^{t}*Bu*), -0.54 (br s, 1H, Cu-O*H*).

UV-Vis (toluene, 25 °C): λ = 325 nm (ε = 7,900 $M^{-1}cm^{-1}$), 350 nm (ε = 6,100 $M^{-1}cm^{-1}$), 413 nm (ε = 5,700 $M^{-1}cm^{-1}$), 466 nm (4,000 $M^{-1}cm^{-1}$)

Note on the synthesis of [(IPr*OMe)Cu(μ-H)]$_2$. During our attempts to isolate [(IPr*OMe)Cu(μ-H)]$_2$, [(IPr*OMe)Cu(OH)] was consistently present as a 5-10% impurity. We were unable to develop a reproducible procedure to remove it. We verified that the impurity arises from adventitious water by treating as-isolated [(IPr*OMe)CuH]$_2$ with a solution of H_2O in THF and isolating and characterizing the product, as described below. The ^{1}H NMR spectrum of the resulting material is identical to the impurity in the as-isolated [(IPr*OMe)CuH]$_2$ (Figure S27). Based on ^{1}H NMR spectroscopy, quantitative conversion (based on Cu-H) to inserted products is observed for the reaction of the as-isolated [(IPr*OMe)CuH]$_2$ with substrates; the small amount of [(IPr*OMe)Cu(OH)] remains unreacted. Furthermore, [(IPr*OMe)Cu(OH)] has no detectable absorbance in the visible region (Figure S28). Based

on these control experiments, we are confident that the presence of the minor Cu-OH impurity does not impact the results of our reactivity studies with [(IPr*OMe)CuH]$_2$.

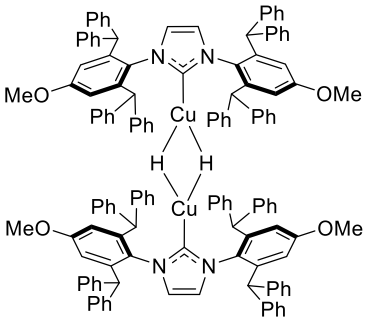

Synthesis of [(IPr*OMe)CuH]$_2$. A slurry of 251 mg (0.24 mmol) [(IPr*OMe)CuCl] in 3 mL of toluene was treated with 75 μL (0.25 mmol, 1.04 equiv) of 40% sodium *tert*-pentoxide in toluene. A white solid precipitated over the course of 10 minutes. A 75 μL (0.41 mmol, 1.7 equiv) portion of HSi(OEt)$_3$ was added, causing formation of an orange solution. The resulting suspension was filtered through a Celite pad, and the pad was washed with toluene until the washes became pale yellow (approximately 15 mL). The filtrate was concentrated to 5 mL, and 25 μL HSi(OEt)$_3$ and 15 mL pentane were added, causing precipitation of an orange solid. The solid was isolated on a fritted glass filter funnel, washed with 10 mL pentane, and dried under vacuum to afford 221 mg of a bright orange solid. Based on ^{1}H NMR spectroscopy, this material contains residual pentane and toluene, as well as 0.1 equiv [Cu(IPr*OMe)(OH)]. The corrected yield is 194 mg (80% yield).

Orange crystals suitable for XRD were grown by layering a solution of 10 mg [(IPr*OMe)CuH]$_2$ in 2 mL THF with 15 mL hexanes in the presence of excess (~3 equiv) HSi(OEt)$_3$ at room temperature.

^{1}H NMR (500 MHz, C$_6$D$_6$): δ 7.59 (d, *J* = 6.7 Hz, 16H, Ar-*H*), 7.04-6.80 (m, 72H, Ar-*H*), 5.92 (s, 8H, C*H*Ph$_2$), 5.36 (s, 4H, *H*-C=C-*H*), 3.92 (s, 2H, Cu-*H*), 3.32 (s, 12H, Ar-OC*H*$_3$).

^{13}C{^{1}H} NMR (126 MHz, C$_6$D$_6$): δ 194.24 (carbene-Cu), 159.86, 144.57, 143.95, 143.56, 143.42, 132.57, 130.82, 130.75, 129.87, 128.89, 126.37, 122.41, 122.32, 115.15, 54.84, 54.81, 51.94, 51.90.

UV-Vis (toluene, 25 °C): λ = 328 nm (ε = 7,100 M^{-1}cm^{-1}), 364 nm (ε = 7,200 M^{-1}cm^{-1}), 412 nm (ε = 7,200 M^{-1}cm^{-1}), 459 nm (5,800 M^{-1}cm^{-1})

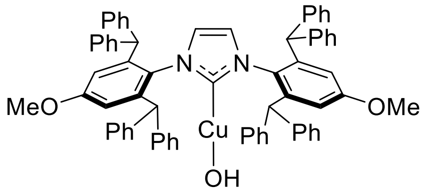

Synthesis of [(IPr*OMe)Cu(OH)]. A 91 mg portion of [(IPr*OMe)CuH]$_2$ containing 15% [(IPr*OMe)Cu(OH)] (0.089 total mmol Cu) was dissolved in 5 mL of THF and treated with 1 mL of 0.11 M H_2O in THF (0.11 mmol, 1.2 equiv). After 3 hours, the solution became colorless. The solvent was removed under vacuum, and the resulting material was redissolved in 2 mL THF and filtered through a fiberglass plug. The filtrate was layered with 15 mL hexanes and stored in a –35 °C freezer overnight resulting in precipitation of a white solid. The solid was collected on a fritted glass filter funnel, washed with pentane, and dried under vacuum. Yield: 53.6 mg (59%)

^{1}H NMR (500 MHz, C$_6$D$_6$): δ 7.48 (d, *J* = 7.7 Hz, 8H, Ar-*H*), 7.16 (m overlapping with solvent residual, 8H, Ar), 7.06-6.91 (m, 24H, Ar-*H*), 6.77 (s, 4H, Ar-*H*), 5.67 (s, 4*H*, C*H*Ph$_2$), 5.51 (s, 2H, *H*-C=C-*H*), 2.98 (s, 6H, -OC*H$_3$*), -0.39 (broad s, 1H, Cu-O*H*).

^{13}C{^{1}H} NMR (126 MHz, C$_6$D$_6$): δ 184.59 (carbene-Cu), 160.58, 143.52, 143.41, 143.38, 130.72, 130.21, 129.85, 129.03, 128.65, 127.10, 126.80, 123.39, 123.36, 123.34, 115.38, 54.58, 54.56, 51.99, 51.97.

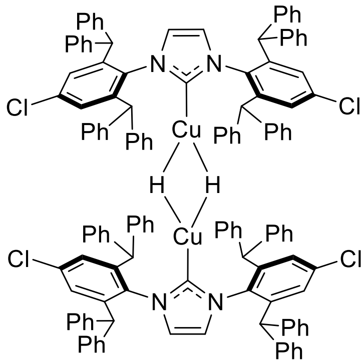

Synthesis of [(IPr*Cl)CuH]$_2$. In an N_2-filled glovebox, a suspension of 99 mg (0.094 mmol) [(IPr*Cl)CuCl] in 2 mL toluene was treated with 30 μL (0.10 mmol, 1.05 equiv) of 40% sodium *tert*-pentoxide in toluene . After 15 minutes, 25 μL (0.14 mmol. 1.4 equiv) of triethoxysilane was added, resulting in formation of an orange solution. The reaction mixture was filtered through Celite and the filtrate was concentrated to 5 mL under vacuum. Pentane (15 mL) was added, causing precipitation of a yellow solid. The solution was left in a –30 °C freezer for 1 hour. The solid was then collected on a glass frit, washed with 3 x 5 mL pentane, and dried under vacuum. Yield: 80 mg (83%).

Orange crystals suitable for XRD were grown by vapor diffusion of pentane in to a concentrated toluene solution of [(IPr*Cl)CuH]$_2$ in the presence of excess (~3 equiv) $HSi(OEt)_3$ at room temperature.

^{1}H NMR (500 MHz, C_6D_6): δ 7.49 (d, *J* = 7.6 Hz, 16H, Ar-*H*), 7.43 (s, 8H, Ar-*H*), 6.95 (t, *J* = 7.2 Hz, 8H, Ar-*H*), 6.90-6.77 (m, 56H, Ar-*H*), 5.91 (s, 8H, C*H*Ph$_2$), 5.17 (s, 4H, H-C=C-H), 3.74 (s, 2H, Cu-*H*).

^{13}C NMR (126 MHz, C_6D_6): δ 193.81 (carbene-Cu), 144.67, 143.84, 142.90, 137.40, 135.62, 130.65, 130.59, 129.91, 129.84, 129.70, 129.11, 128.49, 126.98, 126.59, 121.95, 121.86, 51.84, 51.81.

Key ^{1}H NMR resonances of [(IPr*Cl)Cu-OH] (500 MHz, C_6D_6): δ 5.54 (s, 4H, C*H*Ph$_2$), 5.46 (s, 2H, *H*-C=C-*H*), 0.96 (s, 18H, Ar-^{t}*Bu*), -0.38 (br s, 1H, Cu-O*H*).

UV-Vis (toluene, 25 °C): λ = 367 nm (ε = 8,100 $M^{-1}cm^{-1}$), 441 nm (ε = 7,500 $M^{-1}cm^{-1}$), 466 nm (ε = 6,700 $M^{-1}cm^{-1}$)

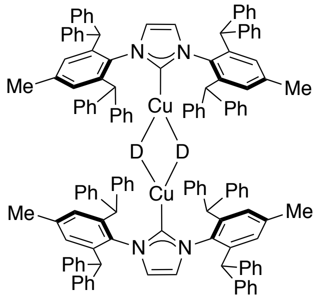

Synthesis of [(IPr*Me)CuD]$_2$. Inside a nitrogen-filled glovebox, to a 20 mL scintillation vial was added [(IPr*Me)CuCl] (500 mg, 0.490 mmol), a small stirring bar, and toluene (10 mL). To the slightly soluble mixture was added 150 μL of sodium pentoxide (33% in toluene). The reaction was stirred at ambient temperature for 30 min to give a yellow solution with no remaining insoluble materials. The reaction mixture was filtered through a medium porous glass frit containing Celite and washed with 3 mL of toluene. The filtrate was transferred to a 100 mL round-bottom flask and 15 mL of THF was added. To this mixture was added $LiAlD(O^tBu)_3$ (138 mg, 0.540 mmol) in 5 mL of THF. The reaction mixture was stirred at ambient temperature during which time it turned intensely yellow, then orange. After 3 h, the volume of the reaction was reduced by 1/3 and the suspension was stirred for another 1 h. The resulting bright yellow precipitate was collected on a medium porous glass frit, washed with 10 mL of THF, washed with 10 mL of pentane, and dried under vacuum to give a yellow-orange solid. Yield = 35% (168 mg, 0.350 mmol). ^{1}H NMR data of [(IPr*Me)CuD]$_2$ matches that of [(IPr*Me)CuH]$_2$ except that the hydride resonance is not observed.

UV-Vis (toluene, 25 °C): λ = 370 nm (ε = 8,500 $M^{-1}cm^{-1}$), 442 nm (ε = 10,700 $M^{-1}cm^{-1}$)

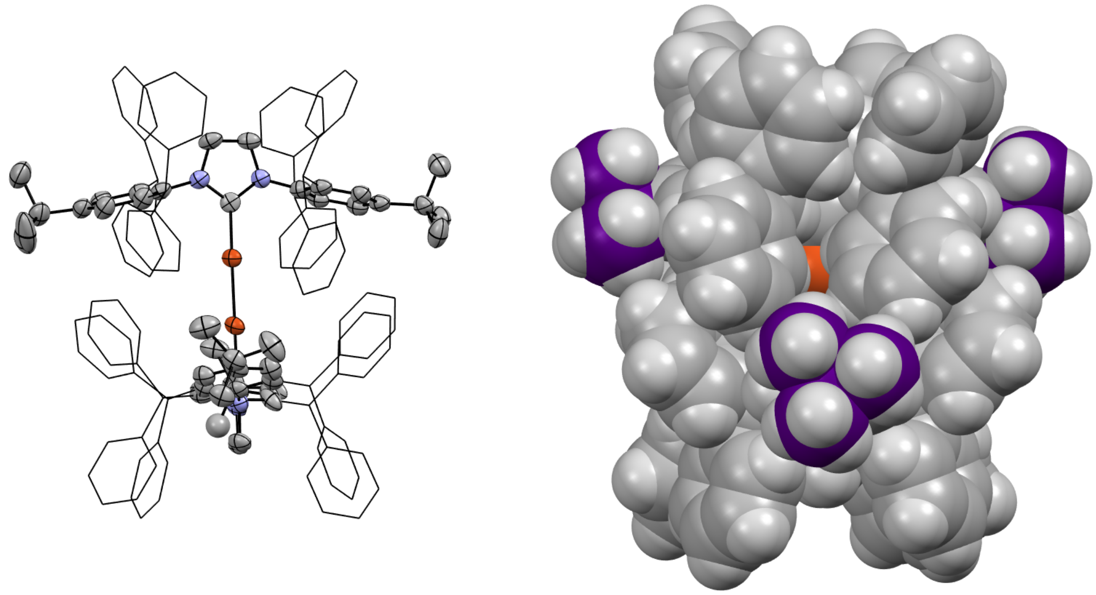

Figure S20. (Left) Thermal ellipsoid plot (50% probability) of [(IPr*^{t}Bu)CuH]$_2$. Hydrogen atoms are not shown, the flanking phenyl groups are shown in wireframe representation, and the disorder of one of the flanking phenyl groups and one of the *tert*-butyl groupis not shown. A second molecule of identical connectivity is not shown. The data for this complex was of poor quality, and the structure is presented to illustrate connectivity, not as a representation of accurate bond lengths and angles. (Right) Space-filling model in the same orientation as the structure on the left; the *tert*-butyl groups are shown in purple.

^{1}H and $^{13}C\{^{1}H\}$ NMR spectra of [(IPr*R)CuH]$_2$

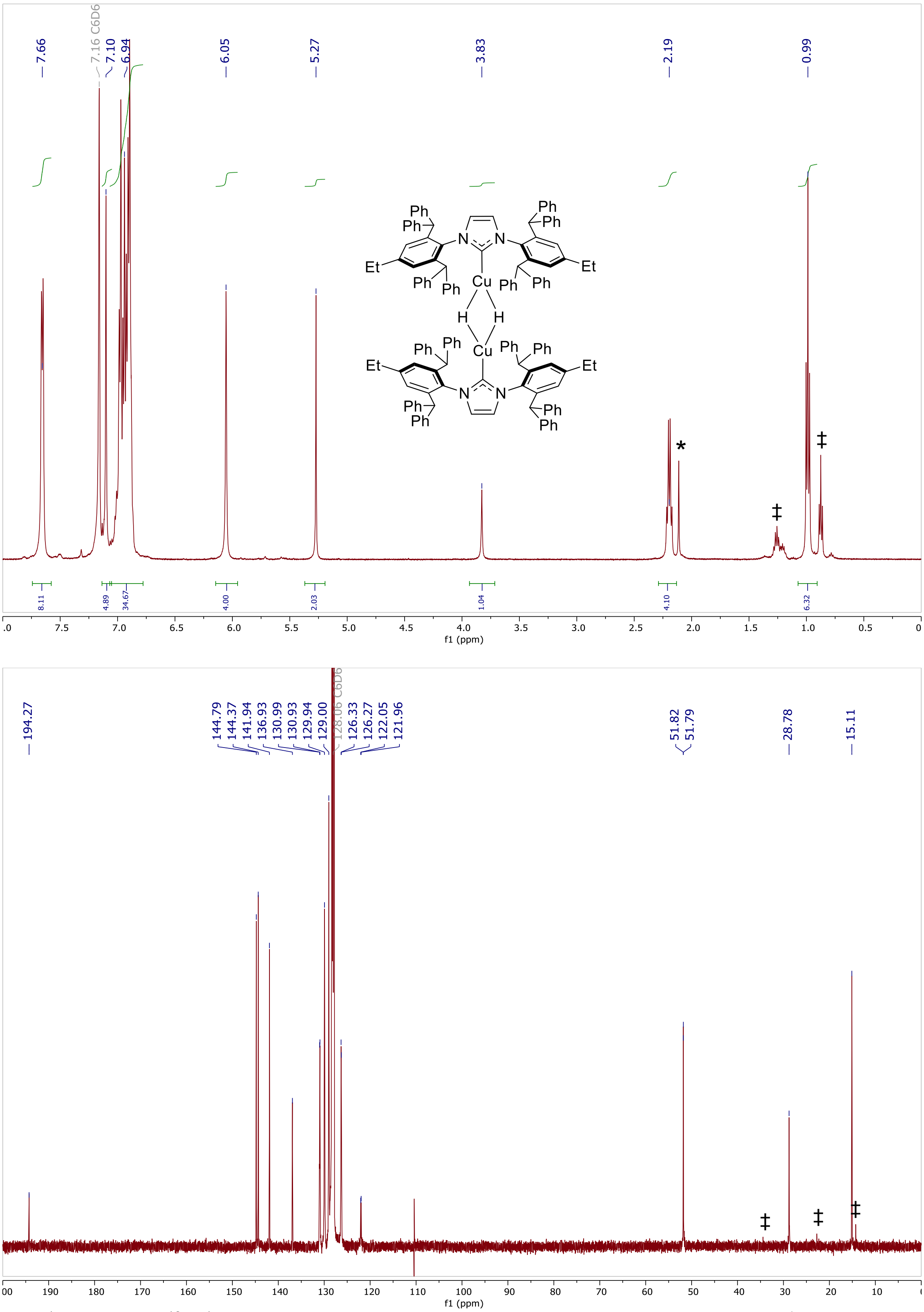

Figure S21. ^{1}H (top) and $^{13}C\{^{1}H\}$ (bottom) NMR spectra of [(IPr*Et)CuH]$_2$ in C_6D_6. *Toluene ‡Pentane

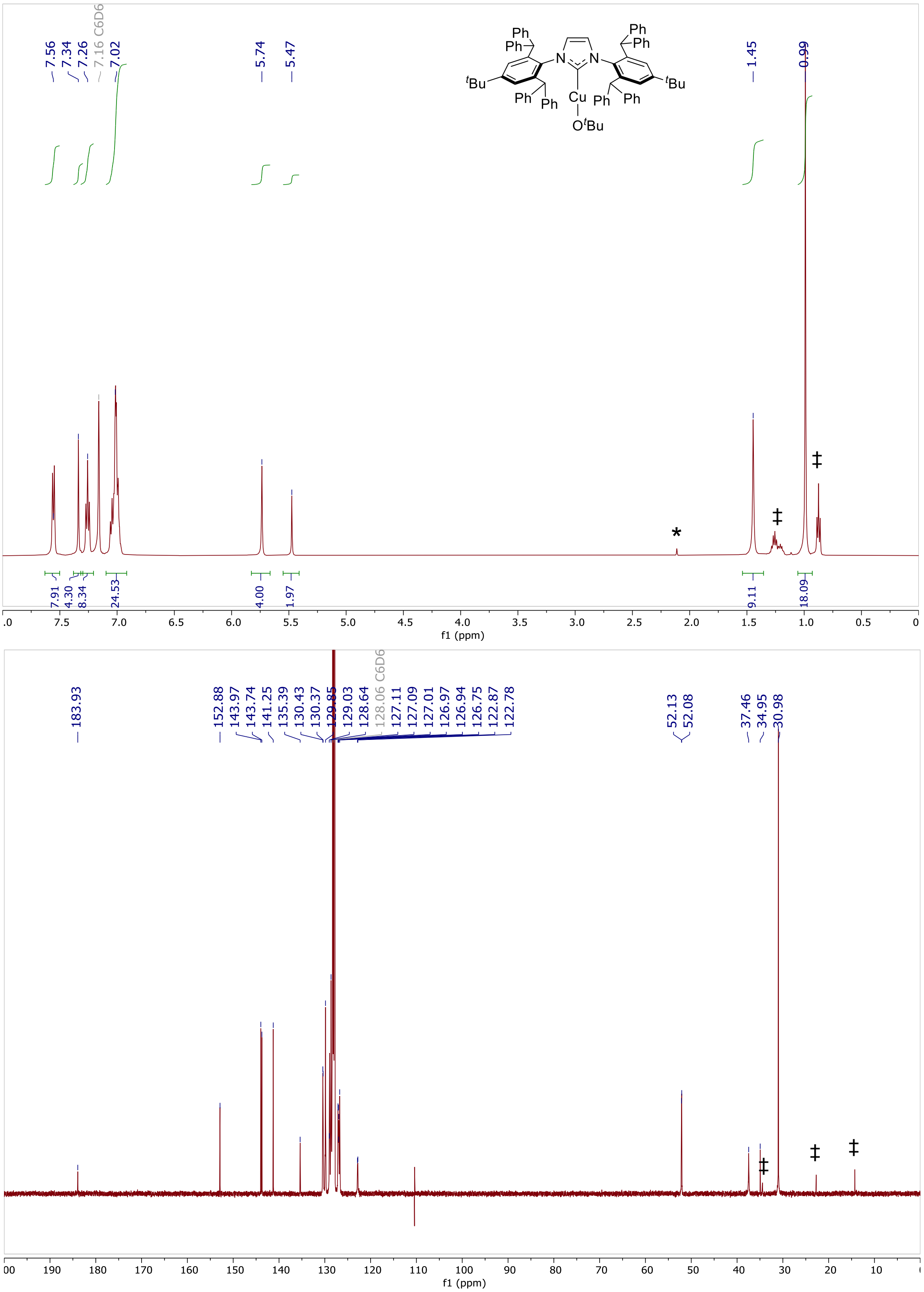

Figure S22. ^{1}H (top) and $^{13}C\{^{1}H\}$ (bottom) NMR spectra of [(IPr*^{t}Bu)Cu(O^{t}Bu)] in C_6D_6. *Toluene ‡Pentane

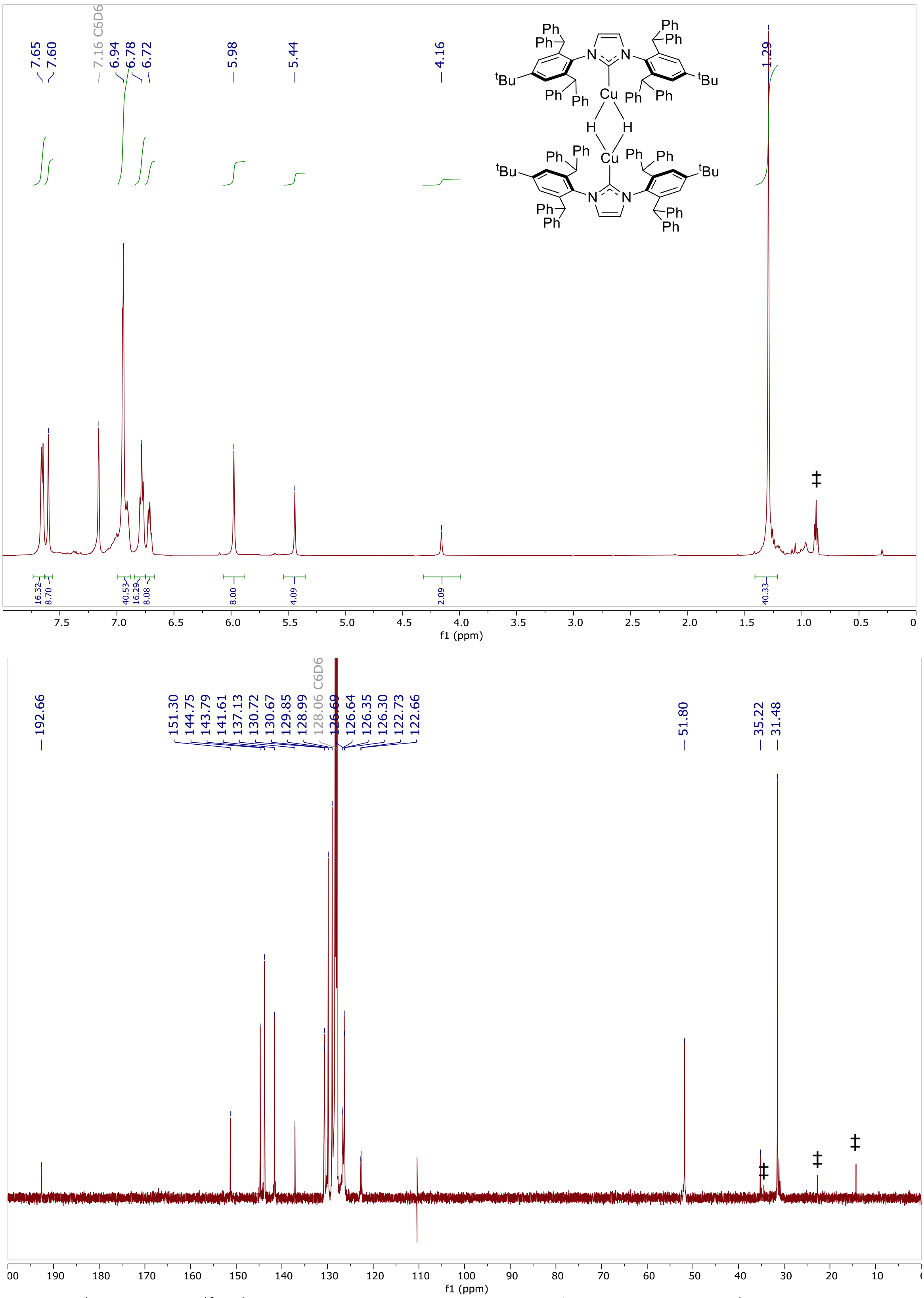

Figure S23. ^{1}H (top) and $^{13}C\{^{1}H\}$ (bottom) NMR spectra of $[(IPr^{*tBu})CuH]_2$ in C_6D_6. ‡Pentane

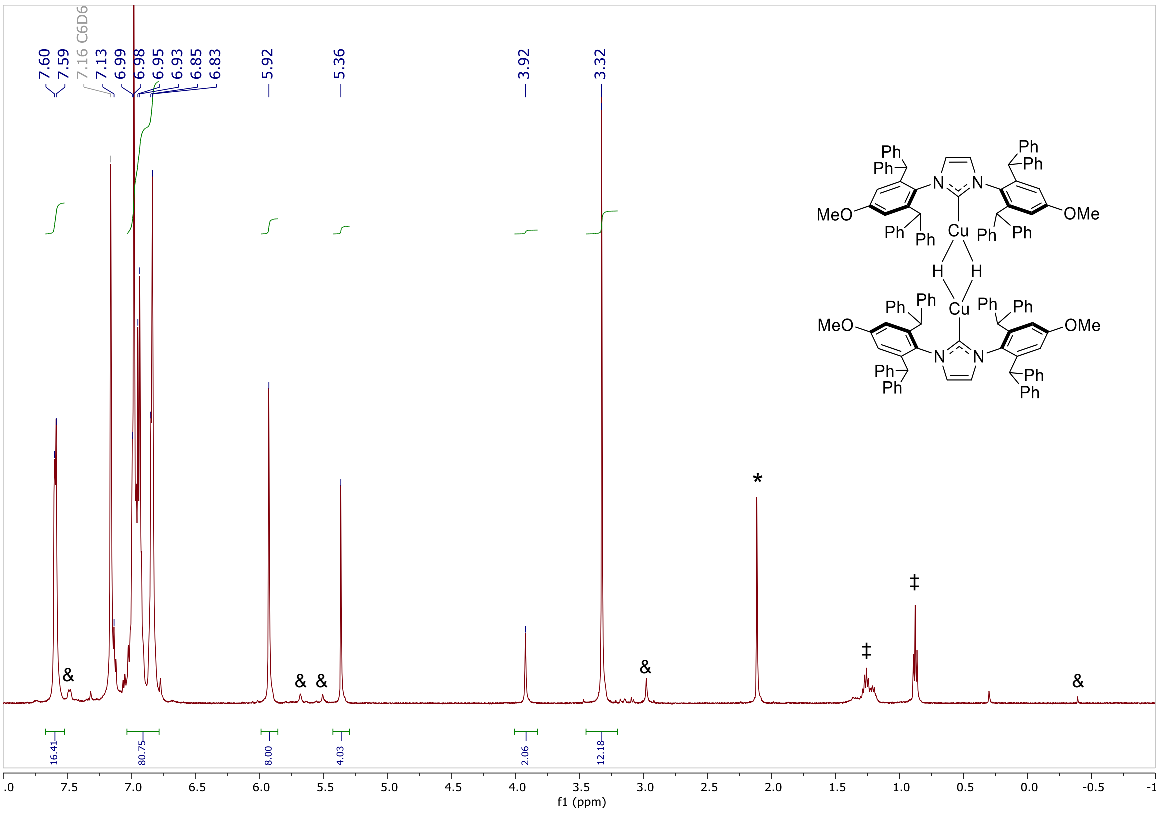

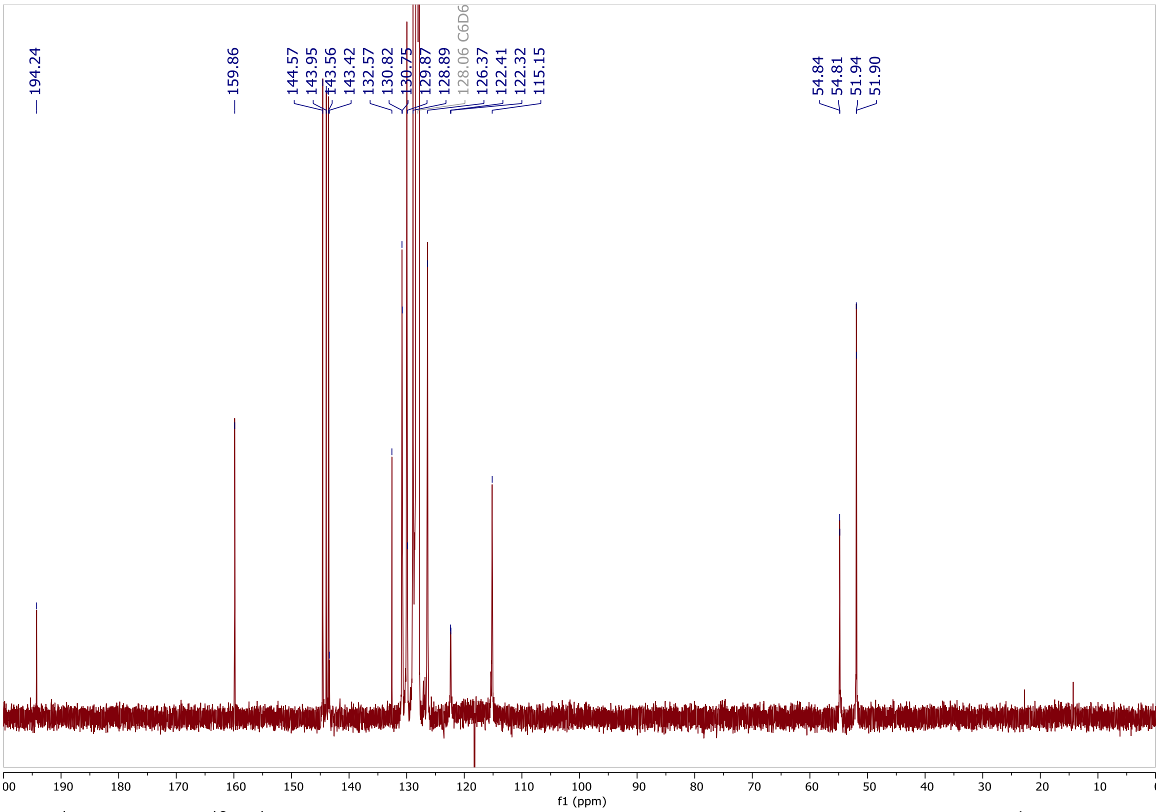

Figure S24. ^{1}H (top) and $^{13}C\{^{1}H\}$ (bottom) NMR spectra of $[(IPr^{*}OMe)CuH]_2$ in C_6D_6. *Toluene ‡Pentane &[(IPr*OMe)CuOH]

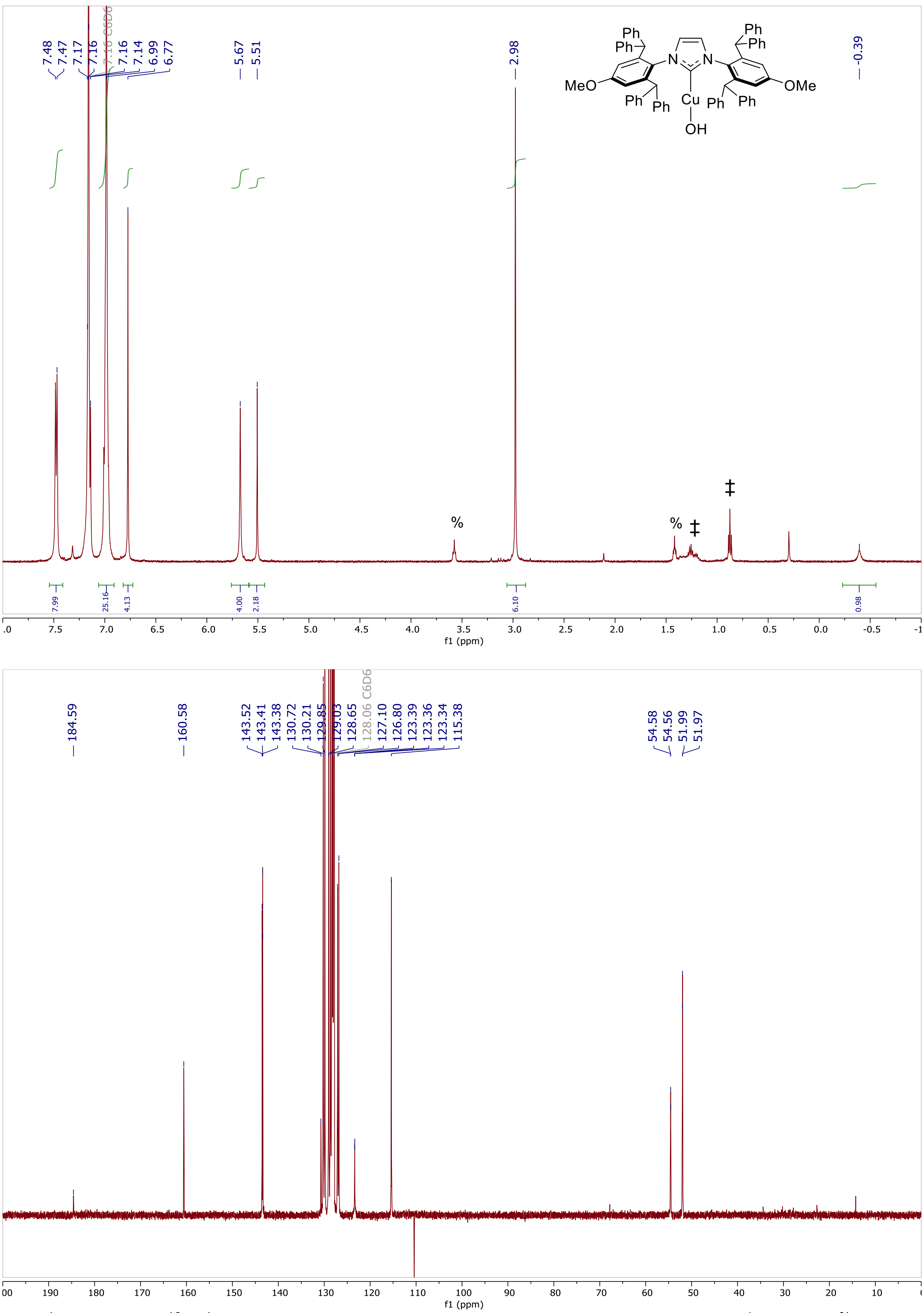

Figure S25. ^{1}H (top) and $^{13}C\{^{1}H\}$ (bottom) NMR spectra of [(IPr*OMe)Cu(OH)] in C_6D_6. ‡Pentane %THF

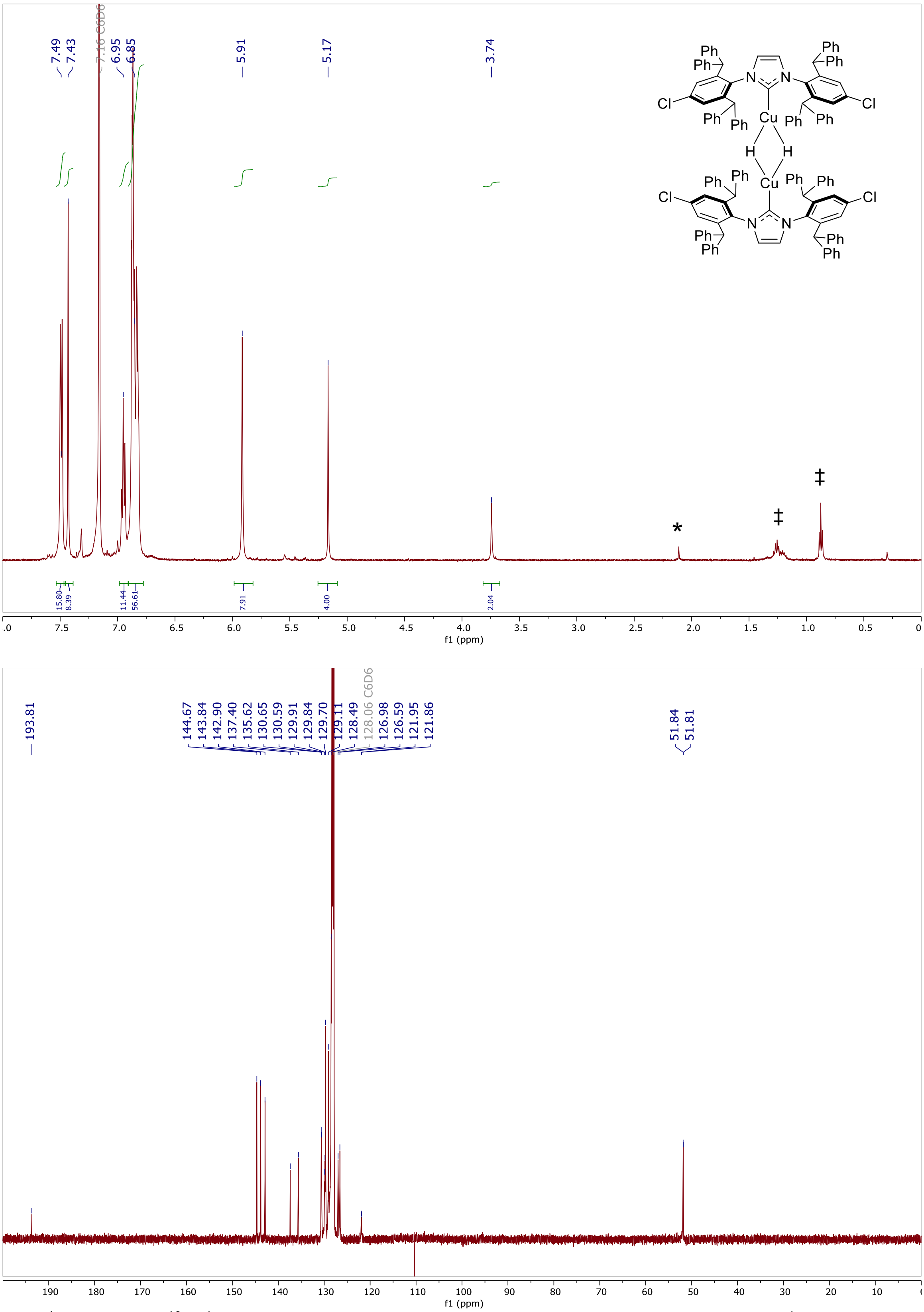

Figure S26. ^{1}H (top) and $^{13}C\{^{1}H\}$ (bottom) NMR spectra of $[(IPr^{*}Cl)CuH]_2$ in C_6D_6. *Toluene ‡Pentane

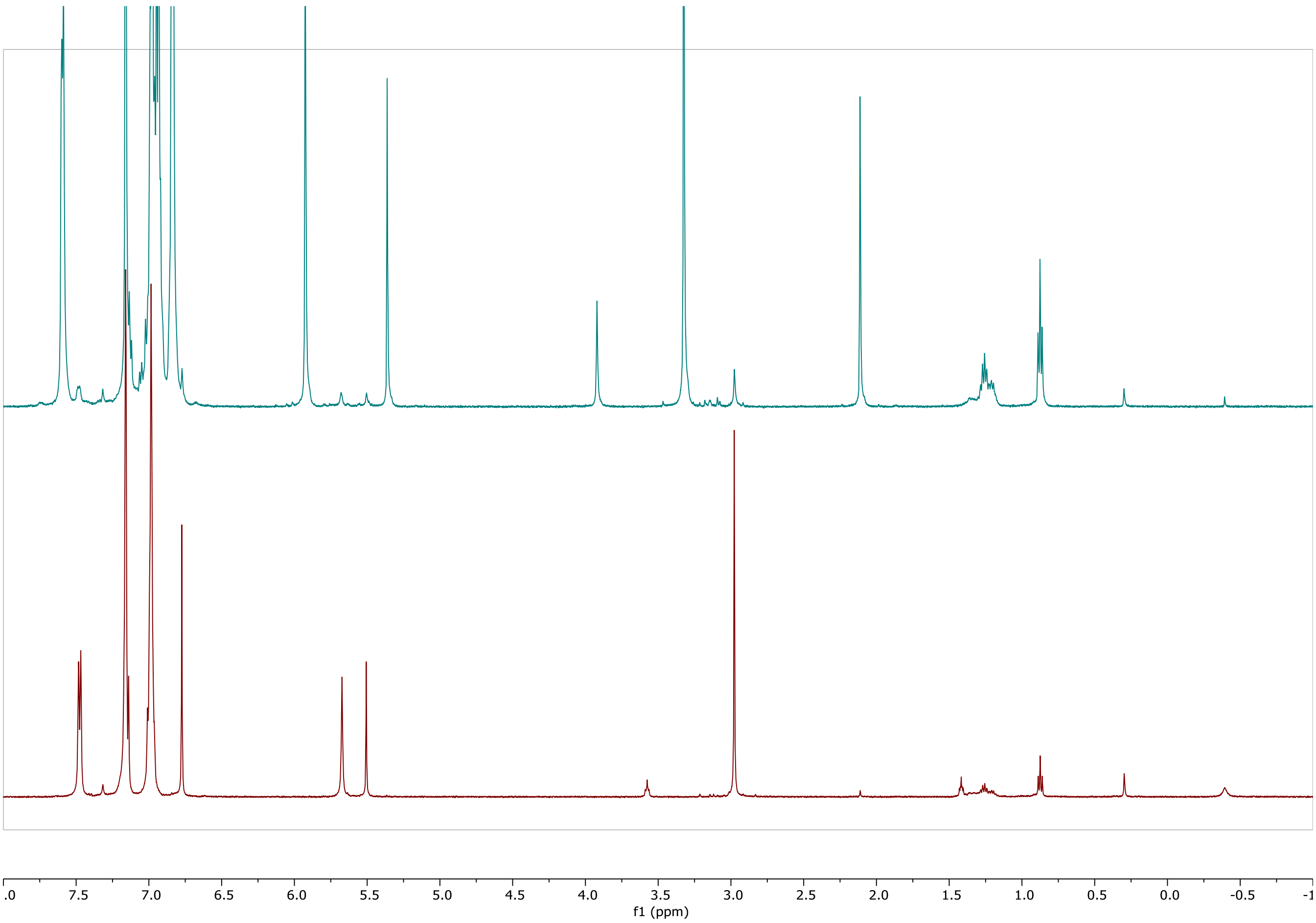

Figure S27. Comparison of the ^{1}H NMR spectra (500 MHz, C_6D_6) of as-isolated [(IPr*OMe)CuH]$_2$ (top) and [(IPr*OMe)Cu(OH)] (bottom).

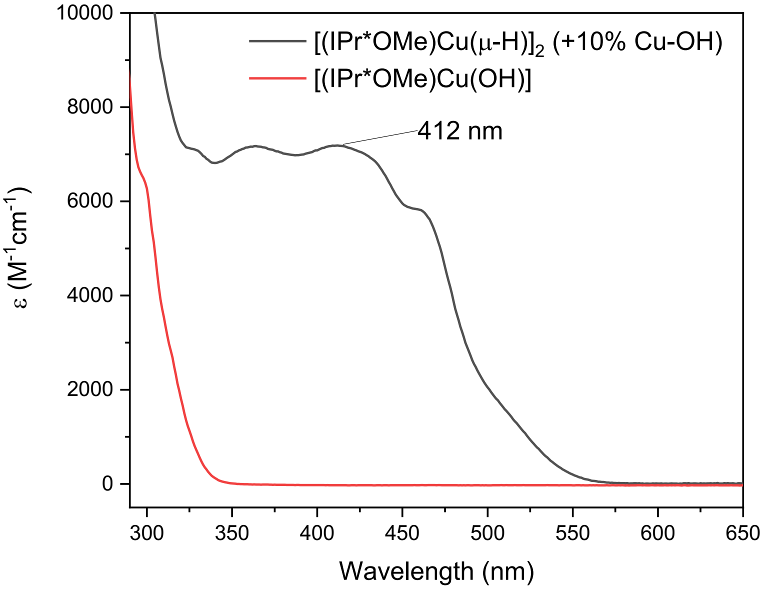

Figure S28. Comparison of the UV-Visible spectra of as-isolated [(IPr*OMe)CuH]$_2$ (black) and [(IPr*OMe)Cu(OH)] (red) in toluene at 25 °C.

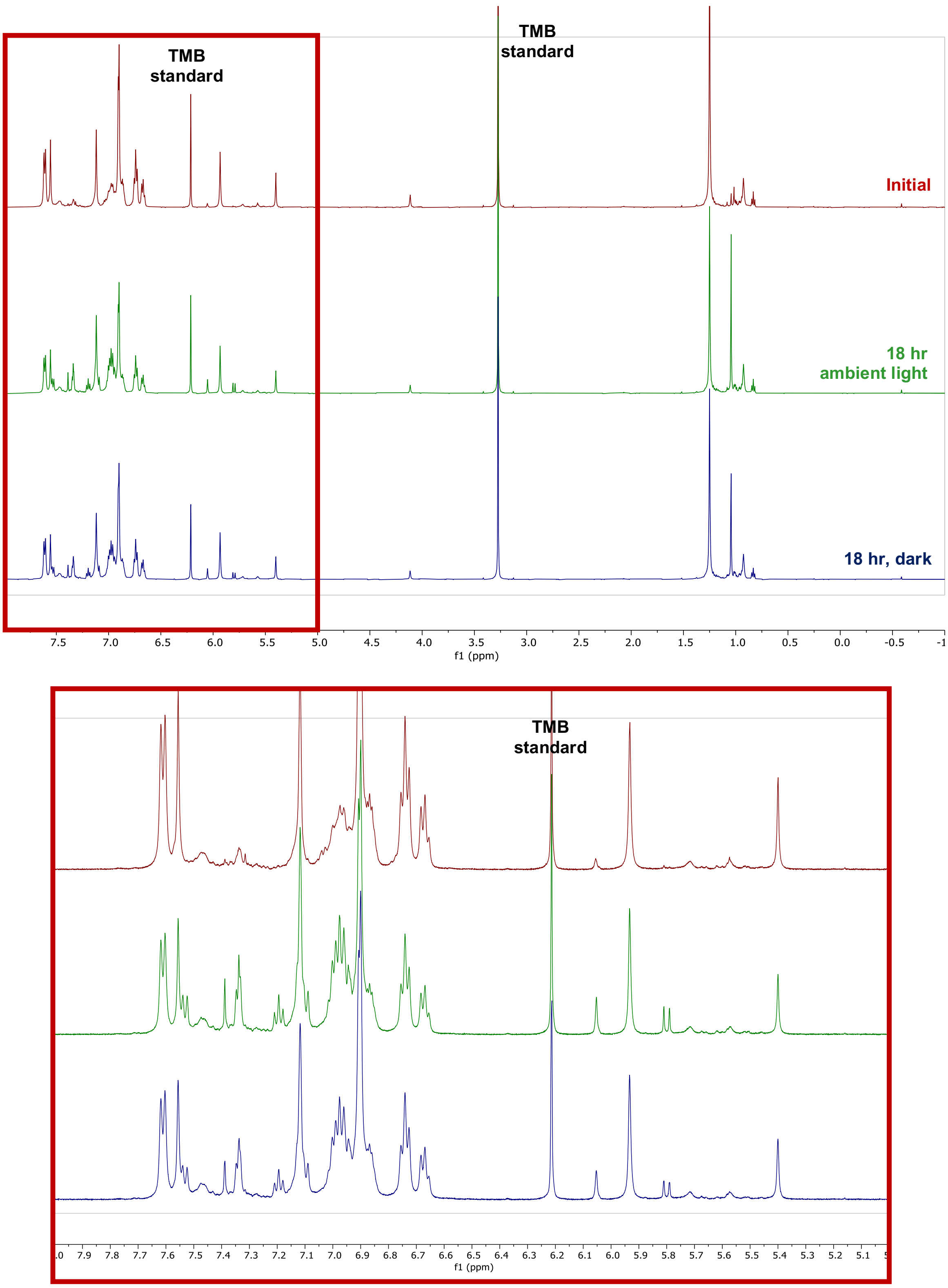

Figure S29. ^{1}H NMR spectra showing decomposition of [(IPr*^{t}Bu)CuH]$_2$ in C_6D_6 after 18 hours at room temperature. The initial spectrum is shown in the top frame. The middle and bottom frames show spectra recorded after 18 hours at room temperature in ambient light and in dark, respectively. Comparison to a 1,3,5-trimethoxybenzene (TMB) internal standard indicates 26% decomposition in light and 18% decomposition in dark.

Synthesis and characterization of insertion products

Synthesis of [(IPr*Me)Cu-NPh(CH_2Ph)] (1)

1 was independently synthesized from [(IPr*Me)CuCl] and NaNPh(CH_2Ph).

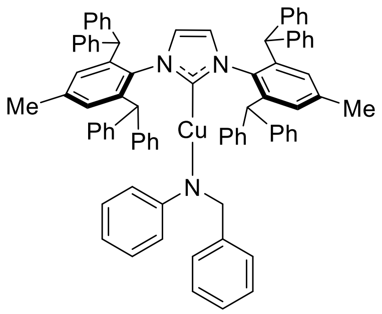

Inside a nitrogen-filled glovebox, [(IPr*Me)CuCl] (250 mg, 0.250 mmol), a small stirring bar, and THF (5 mL) were added to a 20 mL scintillation vial. To this mixture was added a 3 mL THF solution of NaN(Ph)CH_2Ph (56.0 mg, 0.270 mmol) leading a yellow solution. After 2 h, the yellow solution was filtered into a new 20 mL scintillation vial through a glass pipette containing Celite. This saturated THF solution was layered with pentane and kept at ambient temperature, resulting in precipitation of a light yellow crystalline solid which was collected on a medium porousity glass frit, dried under vacuum, and stored in the freezer at -30 ºC. Yield = 70% (200 mg, 0.172 mmol)

^{1}H NMR (500 MHz, 25 ºC, C_6D_6): δ 7.40 (d, *J* = 7.0 Hz, 2H, Ar-*H*), 7.32 (d, *J* = 7.5 Hz, 6H, Ar-*H*), 7.12 (s, 4H, Ar-*H*), 7.07-7.04 (m, 2H), 7.02-6.99 (m, 10H, Ar-*H*), 6.96-6.89 (m, 20H, Ar-*H*), 6.58 (d, *J* = 8.0 Hz, 2H, Ar-*H*), 6.50 (t, *J* = 7.0 Hz, 2H, Ar-*H*), 5.62 (s, 4H, C*H*Ph_2), 5.40 (s, 2H, *H*-C=C-*H*), 4.63 (s, 2H, NCH_2Ph), 1.75 (s, 6H, *Me*).

^{13}C NMR (126 MHz, C_6D_6): δ 183.1 (Cu-carbene), 159.5, 144.6, 143.3, 142.8, 141.5, 140.0, 134.7, 130.1, 129.8, 129.4, 128.6, 128.2, 127.7, 127.3, 126.8, 126.4, 125.0, 122.8, 122.8, 115.1, 111.1, 54.6 (NCH_2Ph), 51.5, 20.9 (*Me*).

Single crystals for XRD measurement were obtained from layering pentane over a saturated THF solution containing the complex at 25 ºC.

Synthesis of [(IPr*Me)Cu-C(Ph)=CH(Ph)] (2)

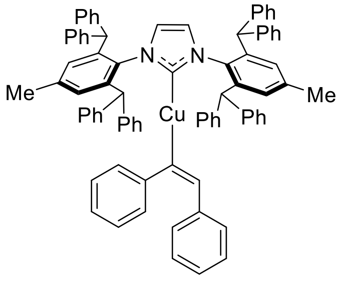

Inside a nitrogen-filled glovebox, to a 20 mL oven-dried scintillation vial was added [(IPr*Me)CuH]$_2$ (100 mg, 0.0510 mmol), a small stirring bar, and THF (5 mL). To this yellow reaction mixture was added diphenylacetylene (19.0 mg, 0.107 mmol) leading to formation of a colorless mixture. After 12 h, the reaction mixture was filtered through as glass pipette into a new 20 mL scintillation vial, and was layered with pentane (15 mL). An off-white crystalline solid precipitated and was isolated by collected by decanting the mother liquor, rinsing with pentane (2 mL), and drying under vacuum. Yield = 65%. (169 mg, 0.150 mmol).

^{1}H NMR (500 MHz, 25 ºC, C_6D_6): δ 7.8 (d, *J* = 7.0 Hz, 8H, Ar-*H*), 7.28 (d, *J* = 8.0 Hz, 2H, Ar-*H*), 7.20 (s, 1H, Cu-CPh=C*H*Ph), 7.11-7.08 (m, 10H, Ar-*H*), 7.01 (s, 6H, Ar-*H*), 6.96-6.89 (m, 28H, Ar-*H*), 5.59 (s, 4H, C*H*Ph_2), 5.41 (s, 2H, *H*-C=C-*H*), 1.75 (s, 6H, *Me*).

^{13}C{^{1}H} NMR (126 MHz, 25 ºC, C_6D_6): δ 184.6 (Cu-carbene), 176.8, 154.2, 143.2, 143.0, 141.3, 141.1, 139.9, 134.8, 134.7, 134.5, 129.9, 129.5, 129.3, 128.7, 128.2, 126.7, 126.4, 126.1, 124.1, 122.8, 122.7, 121.6, 51.4, 20.9.

Single crystals for XRD measurement were obtained by the slow diffusion of pentane into a saturated THF solution containing the complex at 25 ºC.

Synthesis of [(IPr*Me)Cu-C(Et)=CH(Et)] (3)

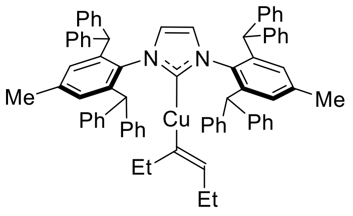

Inside a nitrogen-filled glovebox, a 20 mL scintillation vial was charged with [(IPr*Me)CuH]$_2$ (52 mg, 0.026 mmol), a small stirring bar, and toluene (2 mL). Neat 3-hexyne (150 µL, 1.32 mmol, 50 equiv) was added to the resulting orange suspension. The reaction mixture was stirred overnight at ambient temperature to give a colorless solution which was filtered through a fiberglass plug, layered with 15 mL pentane, and placed in a –30 °C freezer. After 5 days, colorless crystals formed. The mother liquor was decanted off, and the crystals were washed with pentane and dried under vacuum. Yield: 40 mg (70%).

^{1}H NMR (500 MHz, C_6D_6): 7.58 (d, *J* = 7.6 Hz, 8H, Ar-*H*), 7.24 (t, *J* = 7.8 Hz, 8H, Ar-H), 7.09-6.90 (m, 28H, Ar-*H*), 5.93 (tt, *J* = 6.4 Hz, 1.8 Hz, Cβ-*H*), 5.74 (s, 4H, C*H*Ph$_2$), 5.44 (s, 2H, *H*-C=C-*H*), 2.71 (qd, *J* = 7.5, 1.8 Hz, 2H, Cα-C*H*$_2$), 2.58 (pentet, *J* = 7.3 Hz, 2H, Cβ-C*H*$_2$), 1.73 (s, 6H, Ar-C*H*$_3$), 1.30 (t, *J* = 7.5 Hz, 3H, -CH$_2$C*H*$_3$, 1.06 (t, *J* = 7.5 Hz, 3H, -CH$_2$C*H*$_3$).

^{13}C{^{1}H} NMR (126 MHz, C_6D_6): δ 186.15 (carbene-Cu), 170.68, 143.91, 143.59, 141.81, 140.11, 136.79, 136.76, 135.35, 130.53, 130.48, 130.41, 130.36, 129.88, 128.91, 128.57, 127.07, 126.80, 122.96, 122.88, 51.82, 51.78, 29.33, 21.95, 21.30, 21.26, 18.38, 16.63, 16.62.

Synthesis of [(IPr*Me)Cu(*n*-hexyl)] (4)

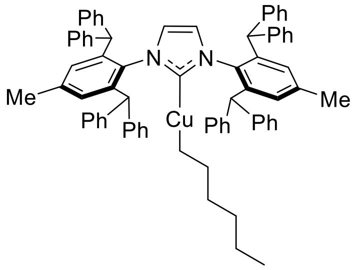

Inside a nitrogen-filled glovebox, a 20 mL scintillation vial was charged with [(IPr*Me)CuH]$_2$ (52 mg, 0.026 mmol), a small stirring bar, and toluene (2 mL). Neat 1-hexene (350 µL, 2.8 mmol, 100 equiv) was added to the resulting orange suspension. The reaction mixture was stirred for 5 hours at ambient temperature to give a colorless solution which was filtered through a fiberglass plug, layered with 15 mL pentane, and placed in a –30 °C freezer. Colorless crystals formed overnight. The mother liquor was decanted off, and the crystals were washed with pentane and dried under vacuum. Yield: 45 mg (79%).

^{1}H NMR (500 MHz, C_6D_6): δ 7.54 (d, *J* = 7.7 Hz, 8H, Ar-*H*), 7.23 (t, *J* = 7.6 Hz, 8H, Ar-*H*), 7.11-6.89 (m, 28H, Ar-*H*), 5.72 (s, 4H, C*H*Ph$_2$), 5.45 (s, 2H, *H*-C=C-*H*), 2.17 (pentet, *J* = 7.2 Hz, -CH$_2$(C*H*$_2$)$_4$CH$_3$, 1.77 (s, 6H, Ar-C*H*$_3$), 1.52-1.39 (m, 4H, -CH$_2$(C*H*$_2$)$_4$CH$_3$), 1.24 (m, 2H, -CH$_2$(C*H*$_2$)$_4$CH$_3$), 0.93 (t, J = 7.4 Hz, 2H, -C*H*$_2$(CH$_2$)$_4$CH$_3$), 0.87 (t, *J* = 7.3 Hz, 3H, -CH$_2$(CH$_2$)$_4$C*H*$_3$).

^{13}C NMR (126 MHz, C_6D_6): δ 185.91, 143.90, 143.65, 141.76, 140.01, 135.39, 130.41, 130.37, 129.90, 128.89, 128.60, 127.04, 126.92, 126.72, 122.78, 122.70, 51.80, 51.76, 38.89, 33.25, 31.71, 23.47, 21.36, 21.32, 14.79, 13.68.

Colorless single crystals suitable for XRD were grown by layering a concentrated THF solution of **4** with pentane and storing overnight in a –30 °C freezer.

Synthesis of [(IPr*Me)Cu-$(CH_2)_4CH=CH_2$] (5)

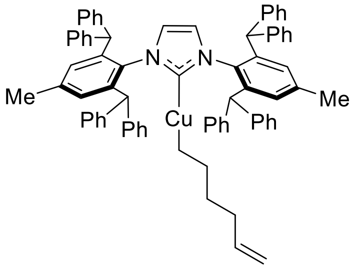

Inside a nitrogen-filled glovebox, a 20 mL scintillation vial was charged with $[(IPr^*Me)CuH]_2$ (52 mg, 0.027 mmol), a small stirring bar, and toluene (2 mL). Neat 1,5-hexadiene (160 μL, 1.3 mmol, 50 equiv) was added to the resulting orange suspension. The reaction mixture was stirred for 4 hours at ambient temperature to give a colorless solution which was filtered through a fiberglass plug, layered with 15 mL pentane, and placed in a −30 °C freezer. After 12 hours, a white precipitate formed. The solid was isolated on a medium porosity glass frit, washed with pentane, and dried under vacuum. Yield: 34 mg (63%).

^{1}H NMR (500 MHz, C_6D_6): δ 7.53 (d, *J* = 7.7 Hz, 8H, Ar-*H*), 7.22 (t, *J* = 7.8 Hz, 8H, Ar-*H*), 7.12-6.88 (m, 28H, Ar-*H*), 5.89-5.61 (m, 1H, -$CH_2(CH_2)_4CH$=CH_2), 5.71 (s, 4H, C*H*Ph_2), 5.45 (s, 2H, *H*-C=C-*H*), 4.97 (d, *J* = 18.6 Hz, 1H, -$CH_2(CH_2)_3CH=CH_2$), 4.89 (d, *J* = 10.3 Hz, 1H, -$CH_2(CH_2)_3CH=CH_2$), 2.18 (m, 4H, -$CH_2(CH_2)_3CH=CH_2$), 1.77 (s, 6H, Ar-CH_3), 1.51 (pentet, *J* = 7.7 Hz, 2H, -$CH_2(CH_2)_3CH=CH_2$), 0.90 (t, *J* = 7.4 Hz, 2H, -$CH_2(CH_2)_3CH=CH_2$)).

^{13}C{^{1}H} NMR (126 MHz, C_6D_6): δ 185.44, 143.46, 143.24, 141.35, 140.73, 140.70, 139.64, 134.94, 129.98, 129.93, 129.47, 128.50, 128.18, 126.64, 126.31, 122.37, 122.29, 112.74, 51.39, 51.35, 37.73, 34.79, 30.92, 20.97, 20.93, 12.91.

Synthesis of [(IPr*Me)Cu-$OCH_2C_3H_6$] (6)

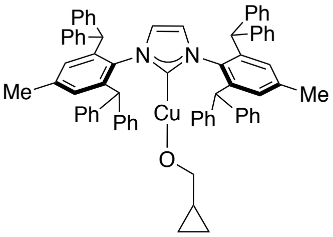

6 was independently synthesized from [(IPr*Me)Cu-Cl] and $NaOCH_2C_3H_6$.

$NaOCH_2C_3H_6$ was prepared from the reaction of $HOCH_2C_3H_6$ (750 mg, 10.4 mmol) and NaHMDS (2.10 g, 11.4 mmol) in toluene (20 mL) at ambient temperature. After 3 h, the reaction mixture was concentrated to dryness to give a white solid that was triturated with hexanes, collected on a medium porosity glass frit, washed with hexanes (10 mL), and dried under vacuum. Yield = 82% (800 mg, 8.50 mmol).

Inside a nitrogen-filled glovebox, to a 20 mL scintillation vial was added [(IPr*Me)CuCl] (250 mg, 0.250 mmol), a small stirring bar, and THF (5 mL). To this mixture was added a 3 mL THF solution of $NaOCH_2C_3H_6$ (25.5 mg, 0.270 mmol) leading a yellow solution. After 2 h, the yellow solution was filtered through a glass pipette containing Celite into a new 20 mL scintillation vial. This saturated THF solution was layered with pentane. After 2 days at 25 °C, the resulting crystalline solid was collected on a porous glass frit and dried under vacuum. Yield = 58% (156 mg, 0.149 mmol)

^{1}H NMR (500 MHz, 25 °C, C_6D_6): δ 7.43 (d, *J* = 7.5 Hz, 6H, Ar-*H*), 7.19 (t, *J* = 7.5 Hz, 8H, Ar-*H*), 7.11 (s, 4H, Ar-*H*), 76.99-6.93 (m, 26H, Ar-*H*), 5.60 (s, 4H, C*H*Ph_2), 5.45 (s, 2H, *H*-C=C-*H*), 4.11 (br s, 2H, $OCH_2C_3H_5$), 1.72 (s, 6H, *Me*), 1.07 (m, 1H, $OCH_2CH(CH_2)_2$), 0.23 (m, 2H, $OCH_2CH(CH_2)_2$), 0.16 (m, 2H, $OCH_2CH(CH_2)_2$).

^{13}C{^{1}H} NMR (126 MHz, 25 °C, C_6D_6): δ 182.9, 143.2, 143.0, 141.3, 139.9, 134.8, 130.0, 129.8, 129.5, 128.6, 128.2, 127.8, 127.6, 127.4, 126.7, 126.4, 122.8, 122.7, 70.9 (broad), 51.5, 20.9, 2.69.

Single crystals for XRD measurement were obtained by layering of pentane into a saturated THF solution containing the complex at 25 °C.

^{1}H and ^{13}C{^{1}H} NMR spectra of insertion products with IPr*Me

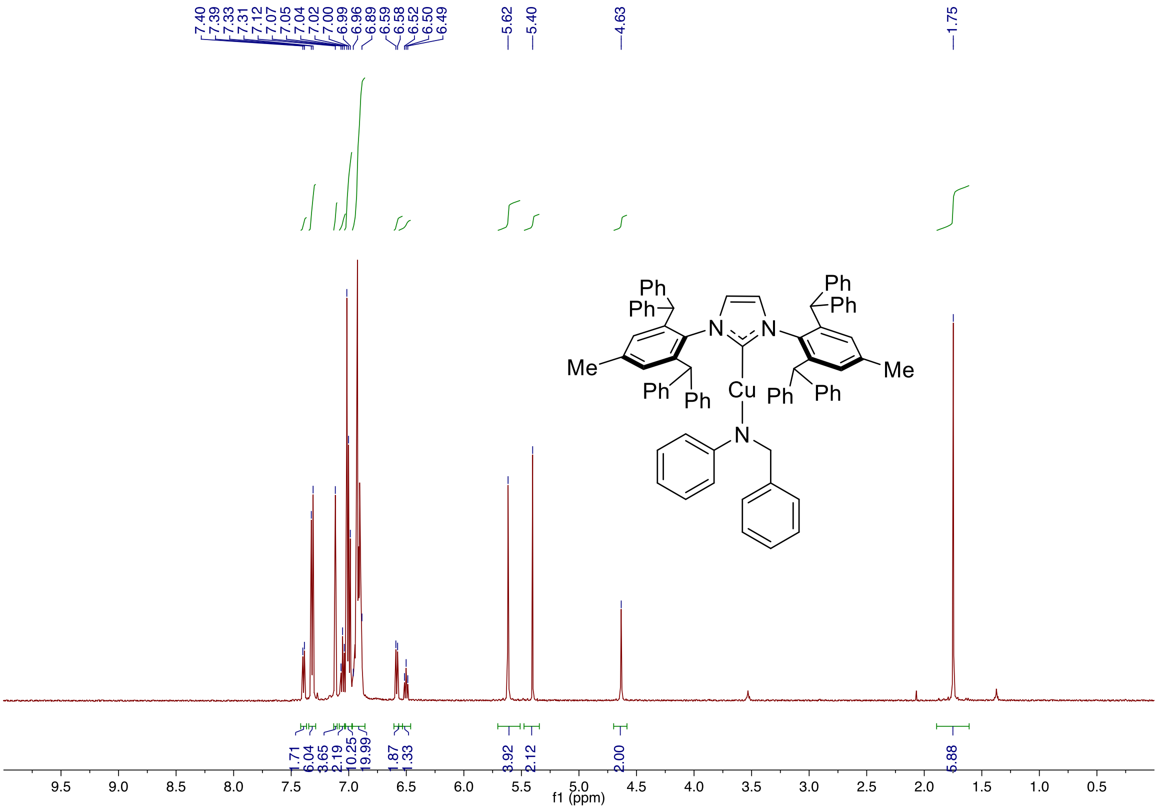

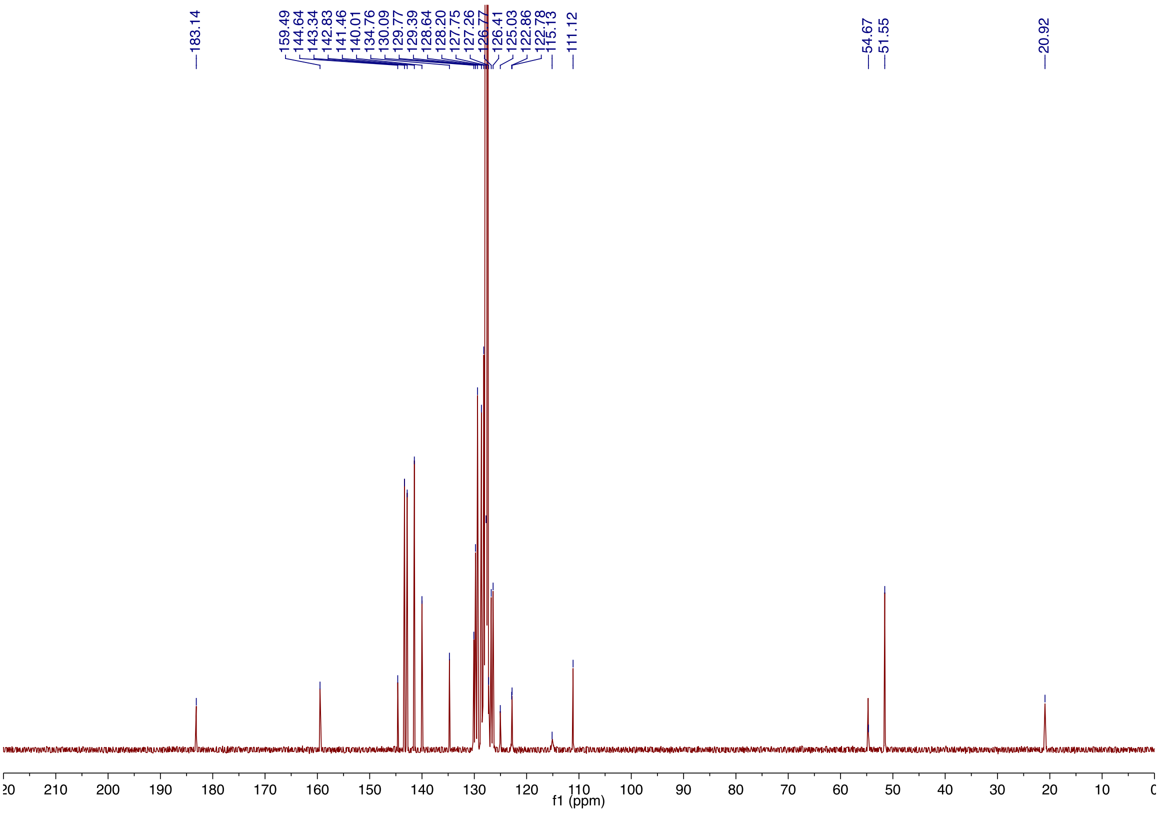

Figure S30. ^{1}H (top) and ^{13}C{^{1}H} (bottom) NMR spectra of **1** in C_6D_6.

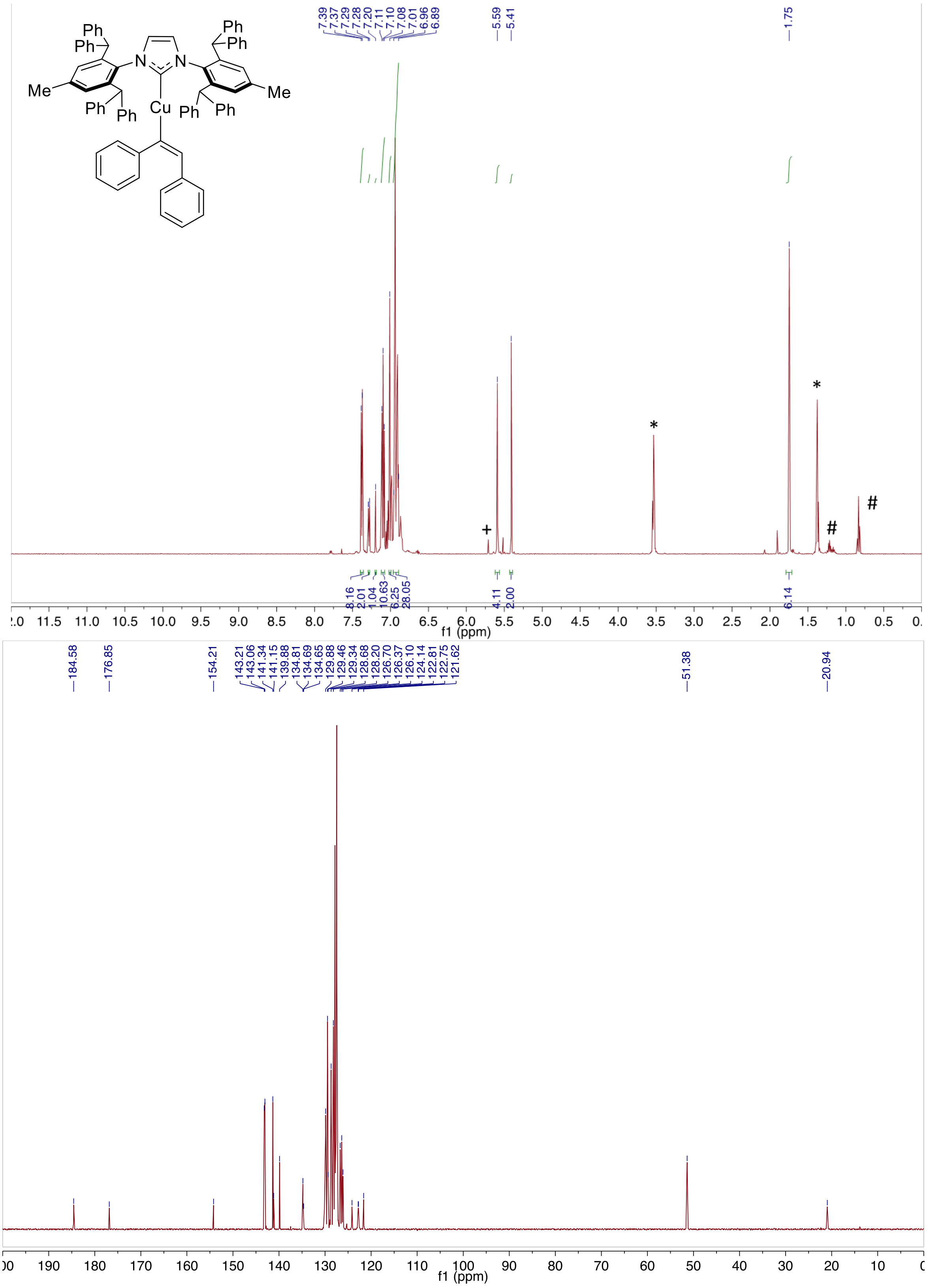

Figure S31. ^{1}H (top) and $^{13}C\{^{1}H\}$ (bottom) NMR spectra of **2** in C_6D_6. $^{+}$ [(IPr*Me)CuOH], * THF, $^{\#}$pentane

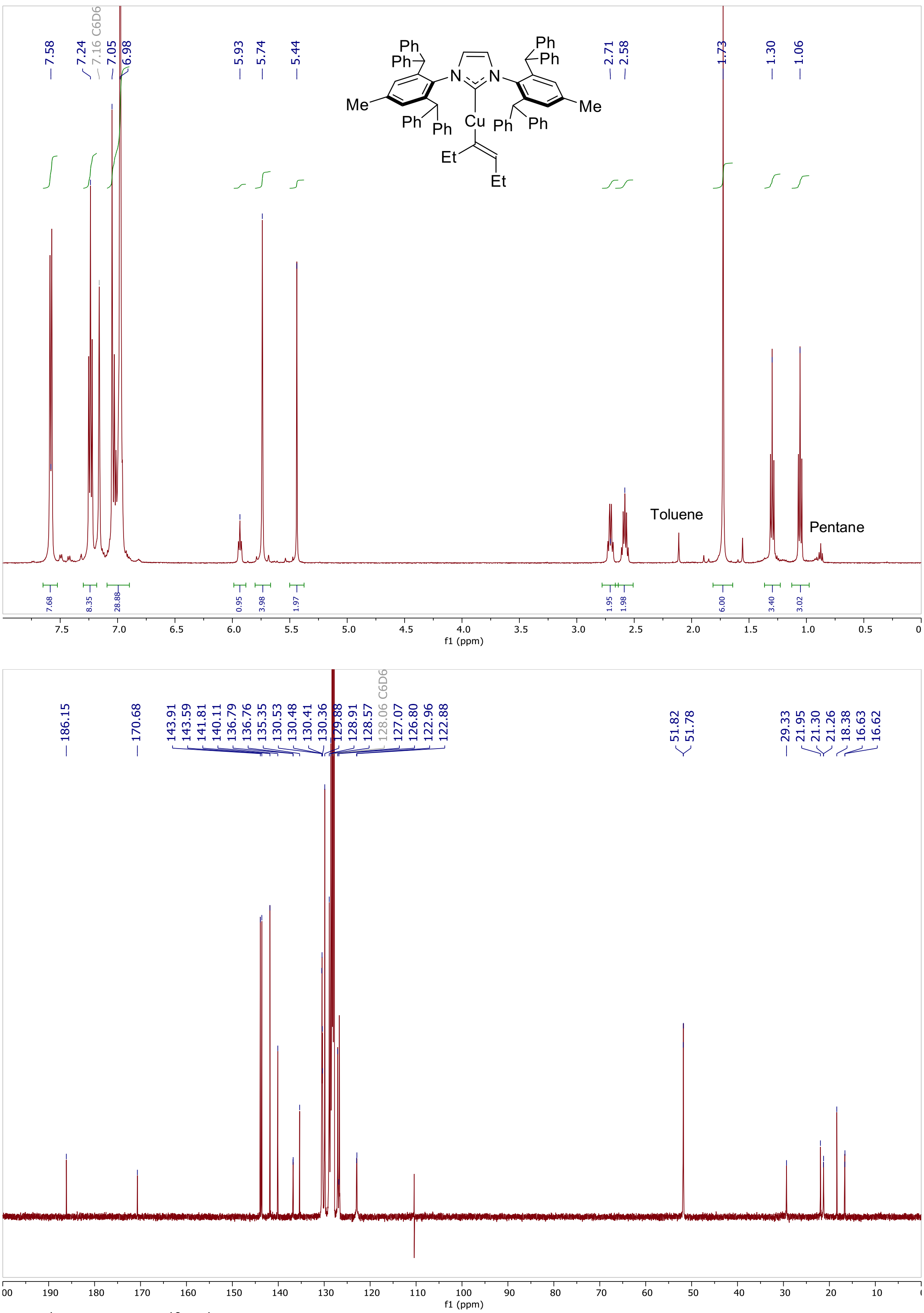

Figure S32. ^{1}H (top) and $^{13}C\{^{1}H\}$ (bottom) NMR spectra of **3** in C_6D_6.

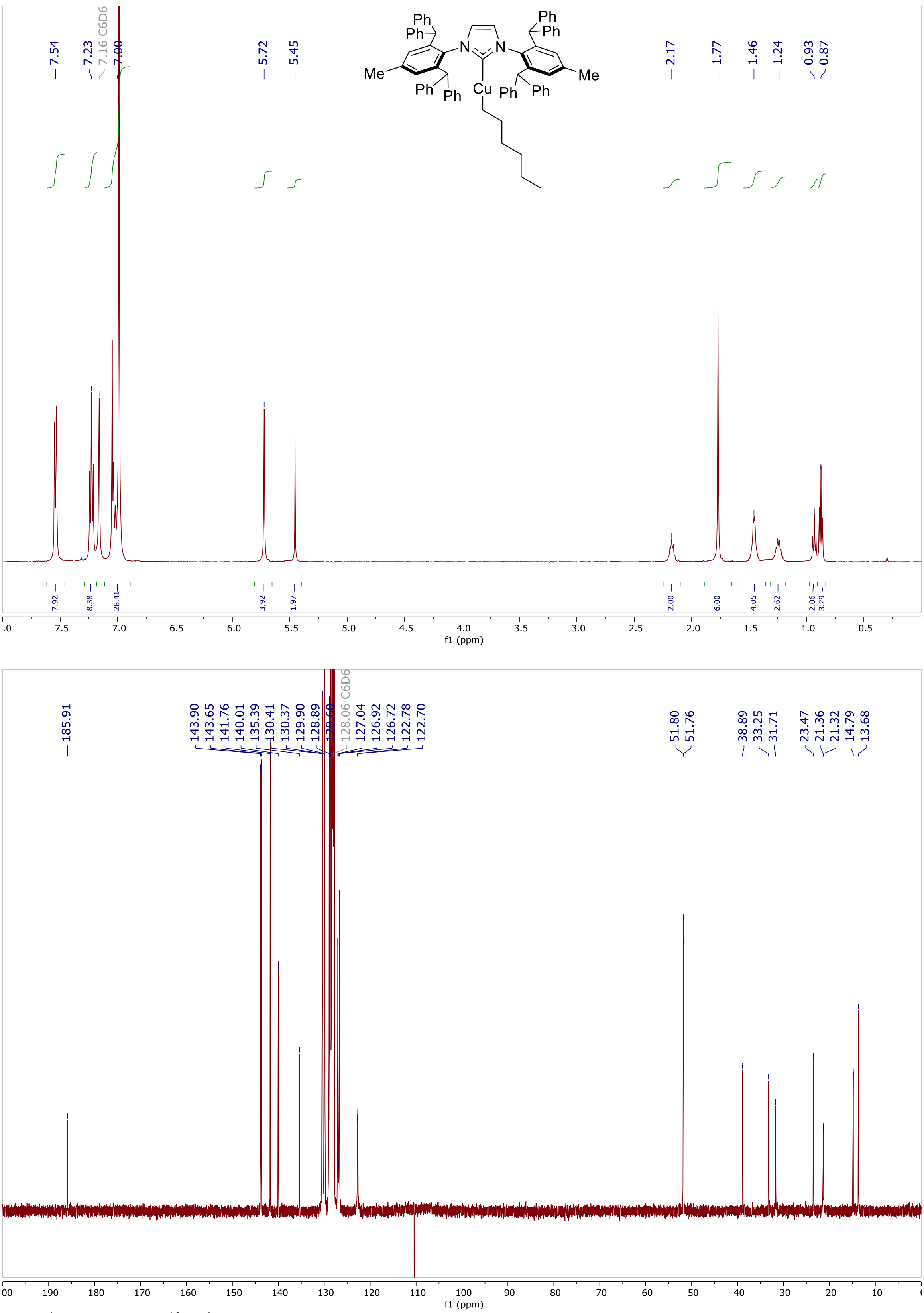

Figure S33. ^{1}H (top) and $^{13}C\{^{1}H\}$ (bottom) NMR spectra of **4** in C_6D_6.

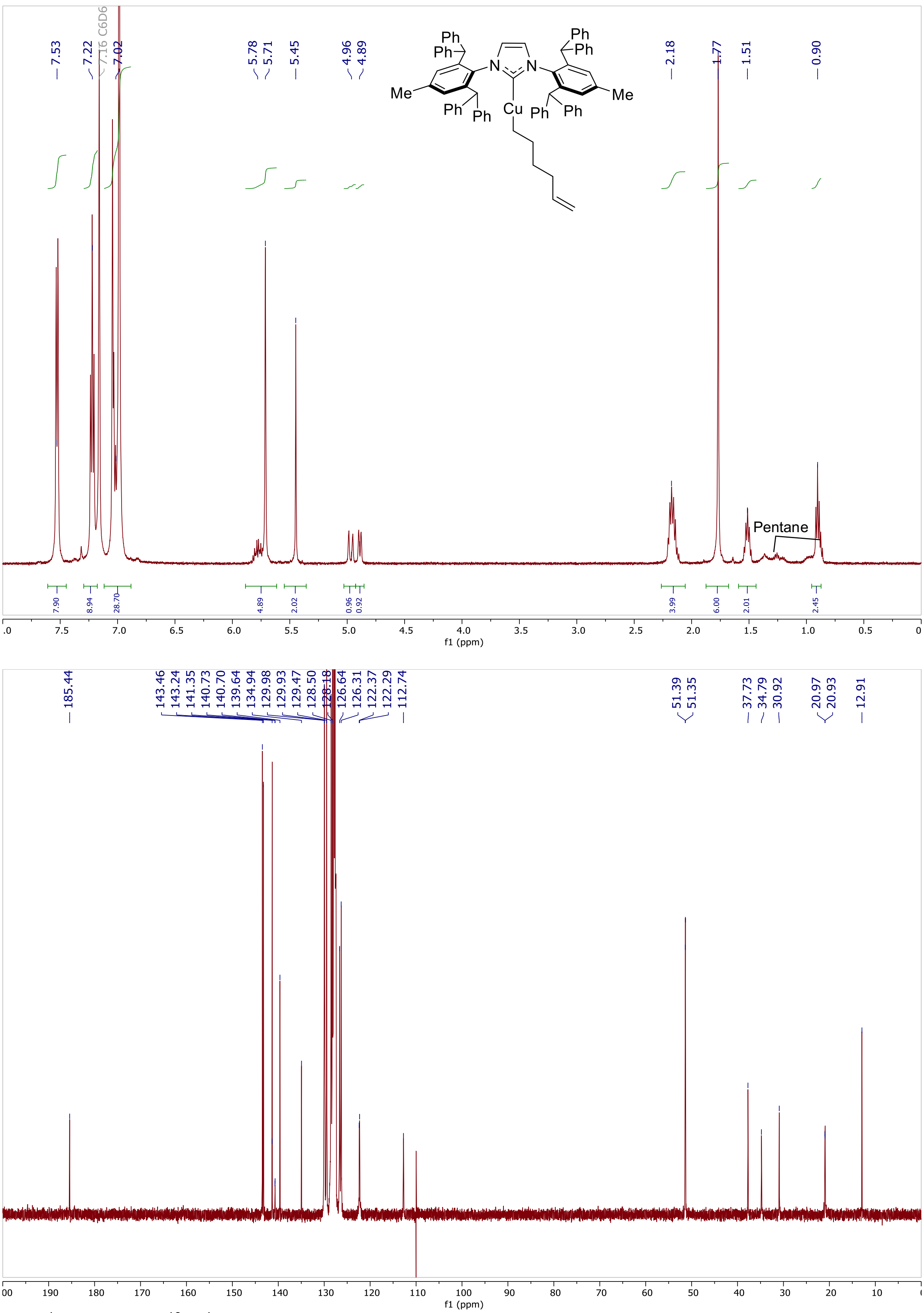

Figure S34. ^{1}H (top) and $^{13}C\{^{1}H\}$ (bottom) NMR spectra of **5** in C_6D_6.

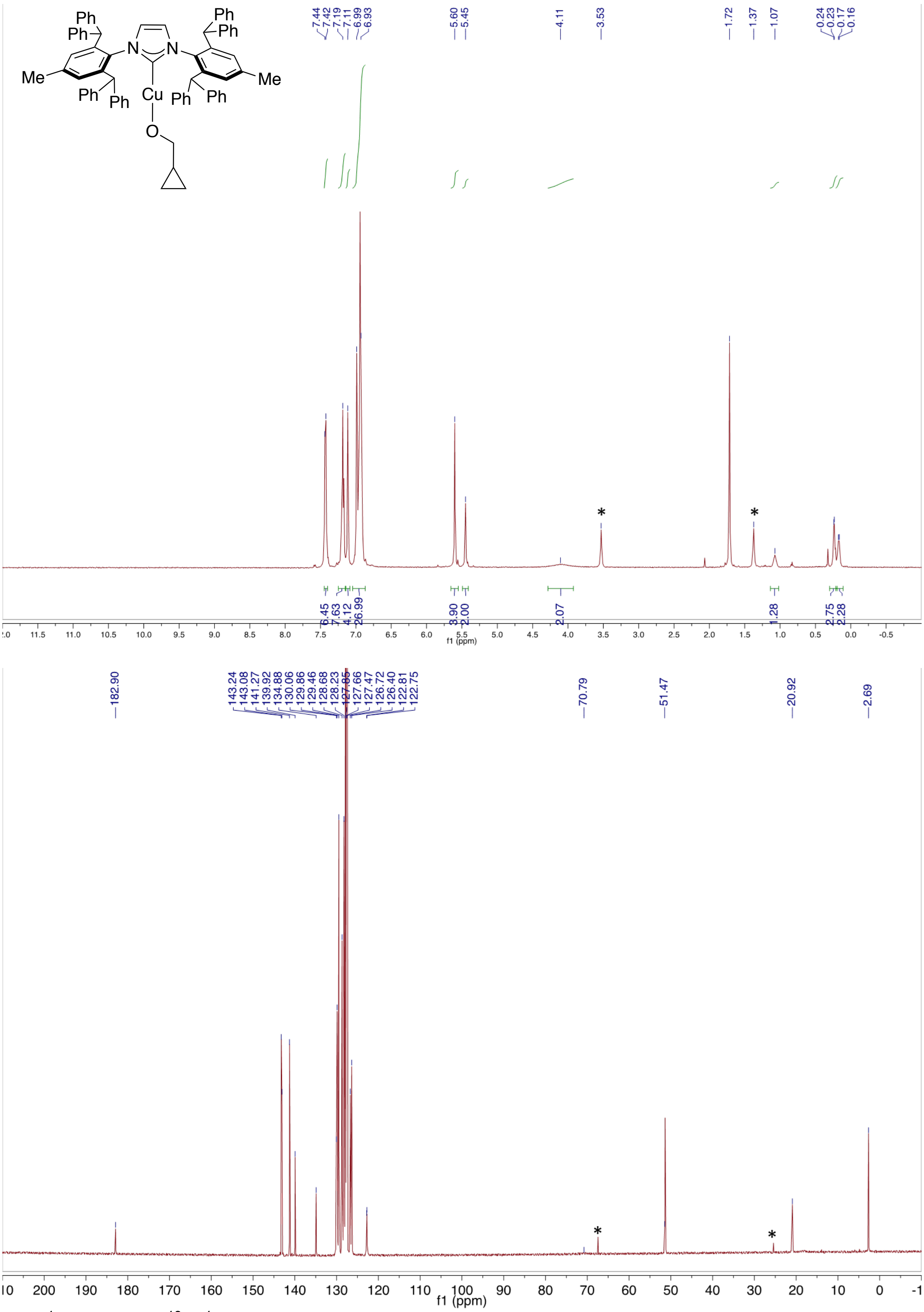

Figure S35. ^{1}H (top) and $^{13}C\{^{1}H\}$ (bottom) NMR spectra of **6** in C_6D_6. *THF.

^{1}H NMR spectra of insertion products generated *in situ*

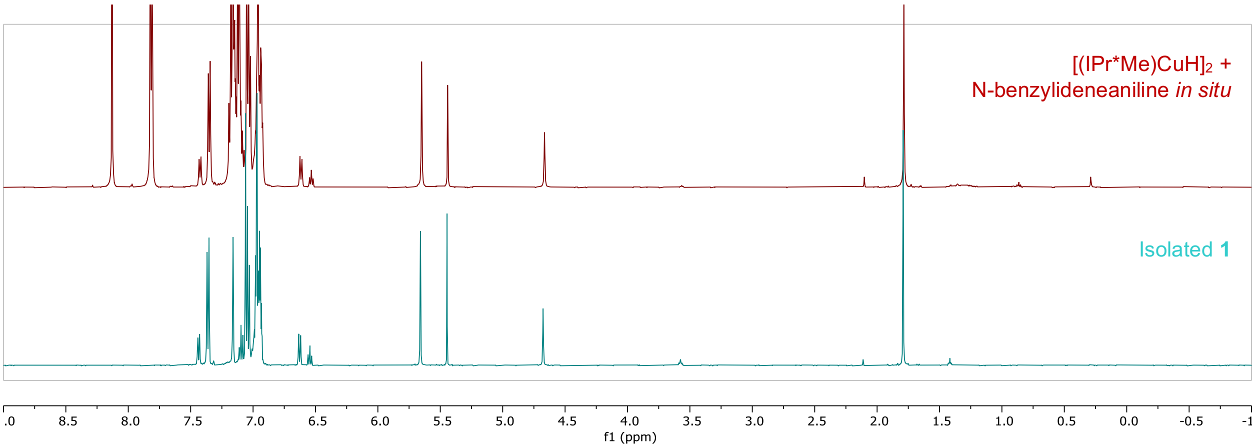

Figure S36. Comparison of the ^{1}H NMR spectra of isolated (top) and *in situ*-generated **1**.

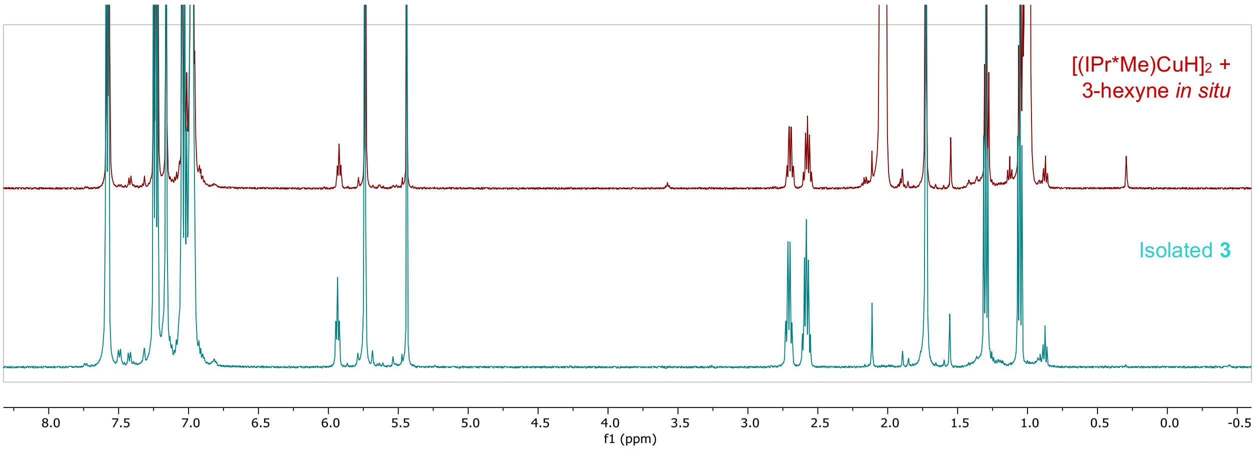

Figure S37. Comparison of the ^{1}H NMR spectra of isolated and *in situ*-generated **3**.

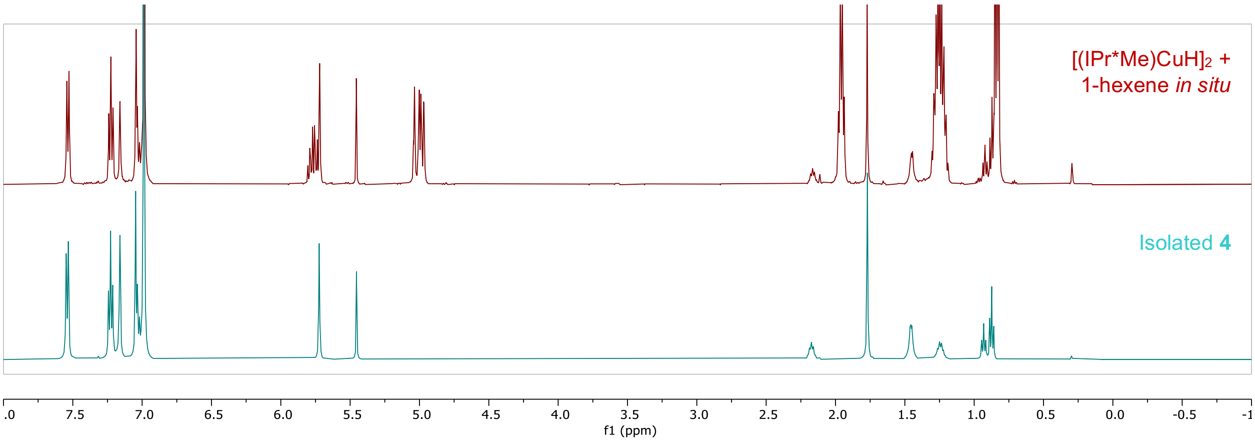

Figure S38. Comparison of the ^{1}H NMR spectra of isolated and *in situ*-generated **4**.

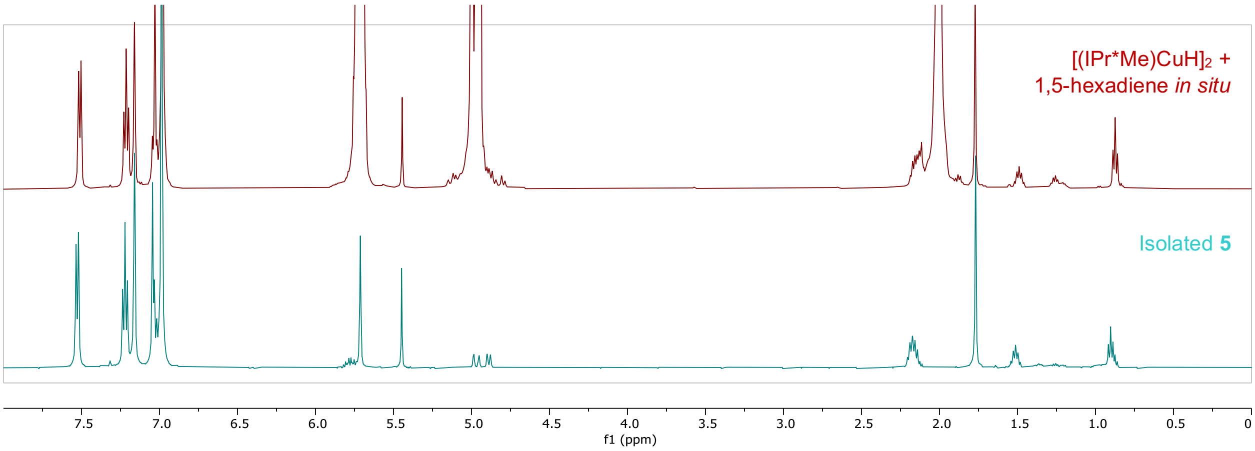

Figure S39. Comparison of the ^{1}H NMR spectra of isolated and *in situ*-generated **5**.

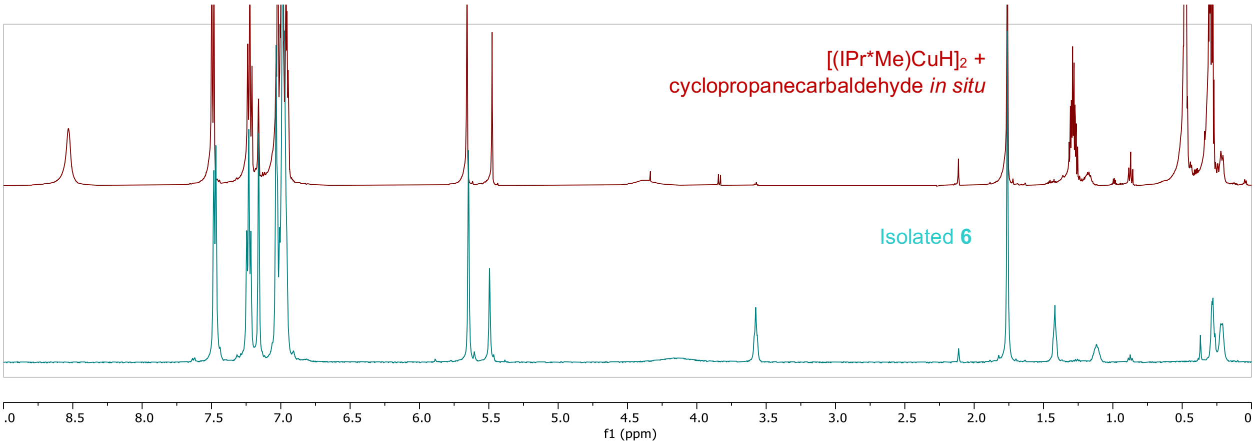

Figure S40. Comparison of the ^{1}H NMR spectra of isolated and *in situ*-generated **6**.

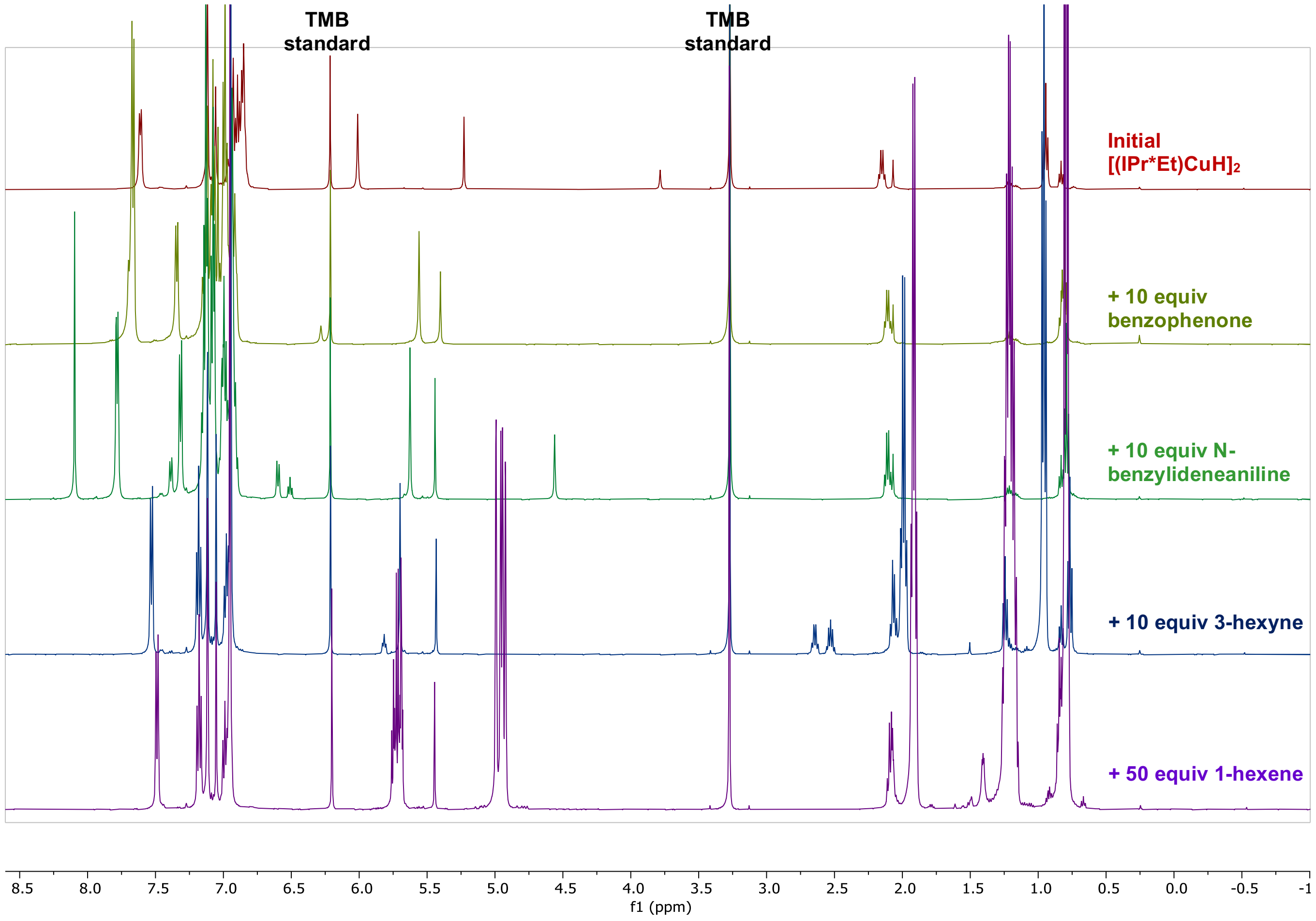

Figure S41. ^{1}H NMR spectra (500 MHz, C_6D_6) of $[(IPr^*Et)CuH]_2$ (top, red) and of insertion products generated *in situ* by addition of excess substrate. In all cases, comparison to a 1,3,5-trimethoxybenzene (TMB) internal standard indicates quantitative conversion to a single new species.

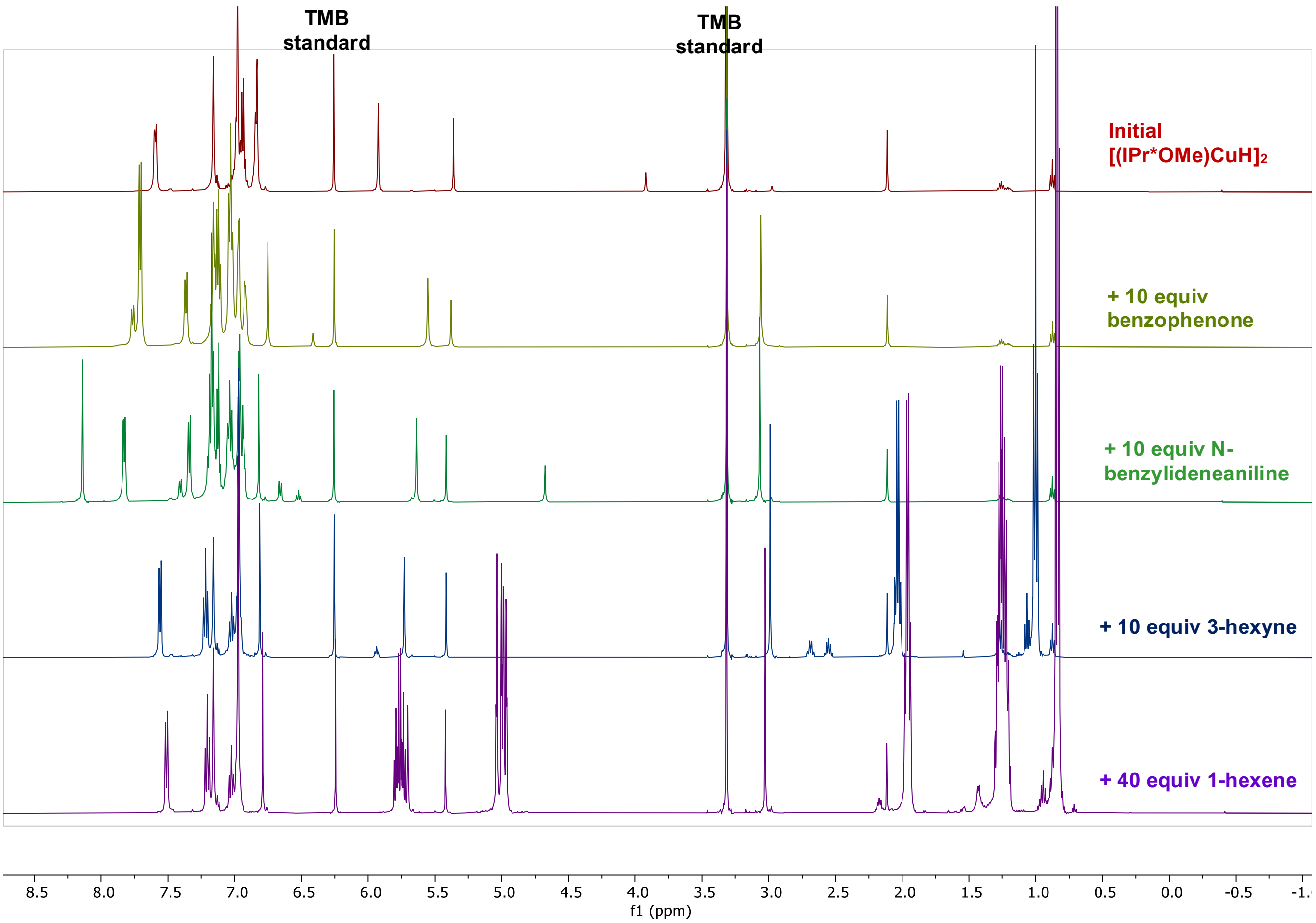

Figure S42. ^{1}H NMR spectra (500 MHz, C_6D_6) of $[(IPr^{*}OMe)CuH]_2$ (top, red) and of insertion products generated *in situ* by addition of excess substrate. In all cases, comparison to a 1,3,5-trimethoxybenzene (TMB) internal standard indicates quantitative conversion to a single new species.

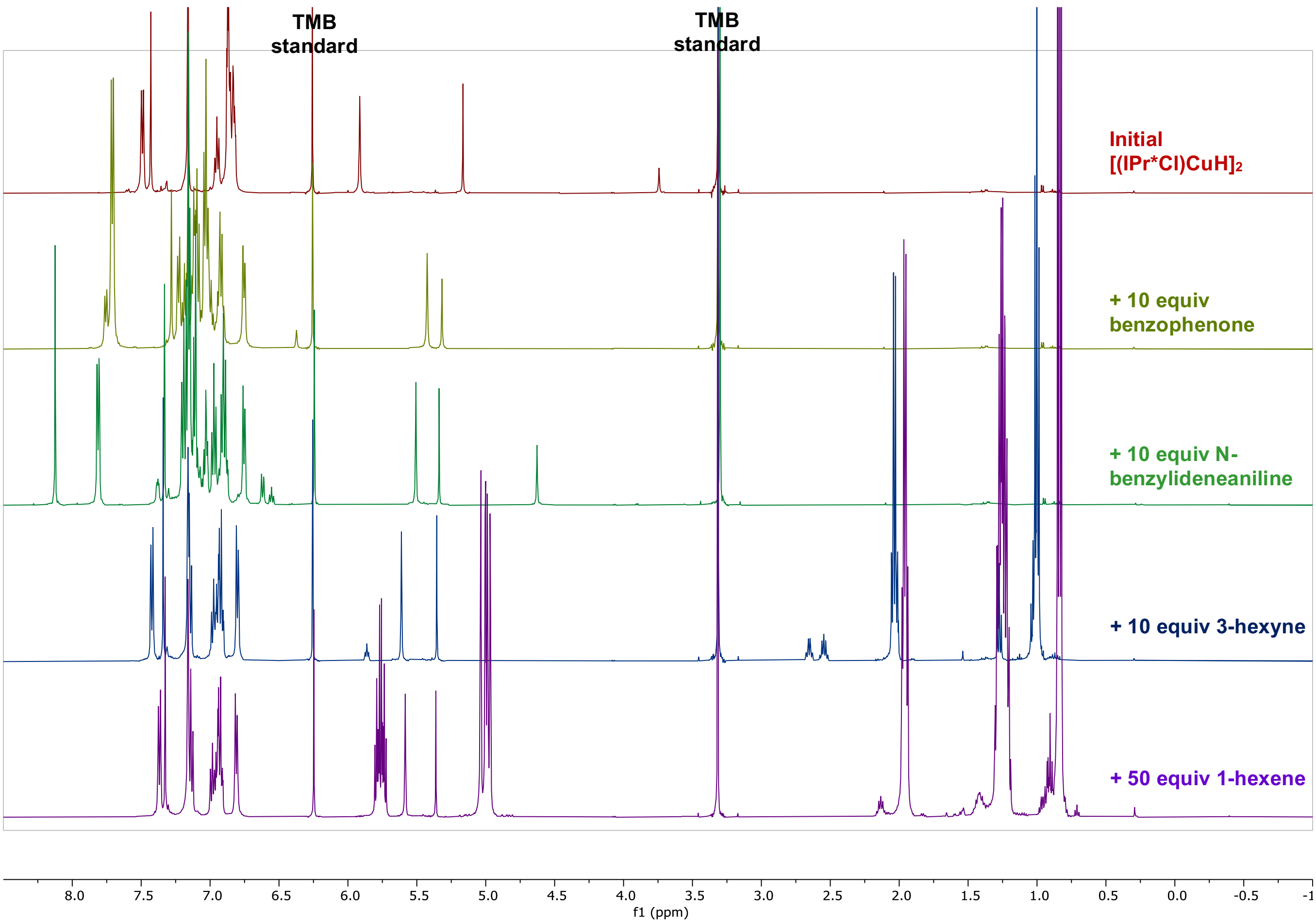

Figure S43. ^{1}H NMR spectra (500 MHz, C_6D_6) of $[(IPr^*Cl)CuH]_2$ (top, red) and of insertion products generated *in situ* by addition of excess substrate. In all cases, comparison to a 1,3,5-trimethoxybenzene (TMB) internal standard indicates quantitative conversion to a single new species.

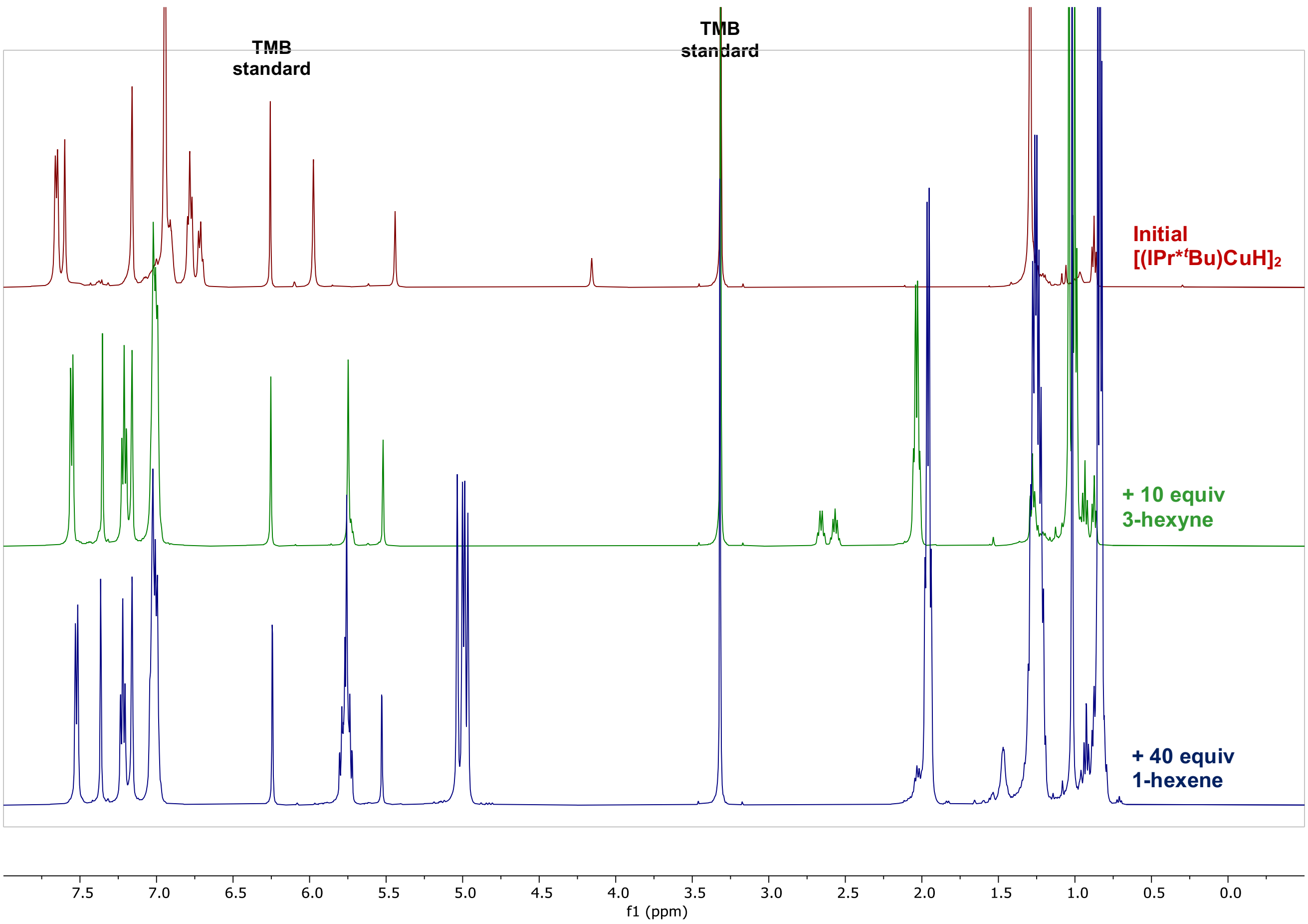

Figure S44. ^{1}H NMR spectra (500 MHz, C_6D_6) showing the products from the reaction of [(IPr*^{t}Bu)CuH]$_2$ (top spectrum) with excess substrate. Comparison to a 1,3,5-trimethoxybenzene (TMB) internal standard indicates quantitative conversion to a single product.

UV-Visible Kinetics Experiments

UV-Visible kinetics studies were performed at 25 °C on a Cary 60 UV-Visible spectrophotometer equipped with a Peltier temperature-controlled cuvette holder. Reactions were monitored using the scanning kinetics function (280 nm – 650 nm) until at least 95% of the initial UV-Visible signal had decayed. For reactions with 3-hexyne, N-benzylideneaniline, and benzophenone, k_{obs} was determined from linear fits of ln([Cu_2H_2]) vs time ($k_{obs} = -1$ x slope). For reactions with 1-hexene and 1,5-hexadiene, k_{obs} was determined from linear fits of $[Cu_2H_2]^{0.5}$ vs time at 3 – 4 substrate concentrations ($k_{obs} = -2$ x slope), and $k_{3/2}$ was calculated from a linear fit of k_{obs} vs substrate concentration ($k_{3/2}$ = slope).

For reactions of 0.1 mM $[(IPr^*R)CuH]_2$ (R = Me, Et, OMe, ^{t}Bu) with 3-hexyne, N-benzylideneaniline, and benzophenone, experiments were performed by injecting substrate solutions in to 1 cm cuvettes sealed with a rubber septum. In each case, control reactions (injection of neat toluene) confirm that negligible background decomposition occurs in this setup on the timescale of the insertion. For R = Cl, the longer reaction time resulted in more significant background decay, so a modified cuvette sealed with a Kontes valve was used. The very similar rates observed for different substrate identities and concentrations demonstrate the high reproducibility of this methodology (Table S5).

Reactions of $[(IPr^*R)CuH]_2$ with 1-hexene and 1,5-hexadiene were performed at slightly higher concentration of copper hydride (0.2 mM) to minimize the impact of background decomposition during the longer reaction times needed for these substrates. Reactions were performed in 5 mm cuvettes sealed with Kontes valves. Substrate was injected through a sidearm sealed with a septum that is isolated from the cuvette when the Kontes valve is sealed. In some cases, the substrate solution was injected prior to removing the cuvette from the glovebox. Negligible differences in rate were observed for these two methods. Control reactions (injection of neat toluene) were performed to confirm that negligible background decay occurs in this setup on the timescale of the insertion reactions. In addition, the absorbances of the products were similar across all substrate concentrations; since the reactions are slower at lower substrate concentrations, this suggests that reaction with substrate is much faster than background decomposition of the copper hydride. Furthermore, the plots of k_{obs} vs substrate concentration are highly linear. Taken together, this demonstrates the high reproducibility that can be achieved with this setup.

Representative procedure for reactions of $[(IPr^*R)CuH]_2$ with 3-hexyne, N-benzylideneaniline, and benzophenone

A 0.5 mM solution of $[(IPr^*R)CuH]_2$ in toluene was prepared in a 10 mL volumetric flask. A 2.3 mL aliquot of this solution was diluted to 10 mL with toluene to give a 0.12 mM solution. A 2.6 mL aliquot of this solution was transferred to a 1 cm cuvette containing a stir bar and the cuvette was sealed with a rubber septum. A 0.15 M solution of 3-hexyne was prepared by diluting 85 μL of 3-hexyne to 5 mL in a volumetric flask. A 0.4 mL portion (200 equiv 3-hexyne per dimer) of this solution was placed in a 1 mL syringe. The cuvette and syringe were brought out of the glovebox and transferred to a cuvette holder at 25 °C in the UV-Visible spectrophotometer. An initial measurement was taken to the verify the concentration of $[(IPr^*R)CuH]_2$. While stirring the reaction mixture, the 3-hexyne stock solution was syringed into the cuvette (final $[(IPr^*R)CuH]_2$ = 0.1 mM, final [3-hexyne] = 20 mM), and the kinetics measurements were started.

Representative procedure for reactions of $[(IPr^*R)CuH]_2$ with 1-hexene and 1,5-hexadiene

A 0.5 mM solution of $[(IPr^*R)CuH]_2$ in toluene was prepared in a 10 mL volumetric flask. A 2.4 mL aliquot of this solution was diluted to 5 mL with toluene to give a 0.24 mM solution. A 1.5 mL aliquot of this solution was transferred to a 5 mm cuvette equipped with a Kontes valve. A 0.24 M solution of 1-hexene was prepared by diluting 60 μL of 1-hexene to 2 mL in a volumetric flask. A 0.3 mL portion of this solution was placed in a 1 mL syringe. The cuvette and syringe were brought out of the glovebox and transferred to a cuvette holder at 25 °C in the UV-Visible spectrophotometer. An initial measurement was taken to the verify the concentration of $[(IPr^*R)CuH]_2$. The 1-hexene stock solution was syringed into the cuvette

(final $[(IPr^*R)CuH]_2$ = 0.2 mM, final [3-hexyne] = 40 mM). The Kontes valve was then closed and the cuvette was shaken to mix the solutions before beginning kinetics measurements.

Table S5. Summary of k_{obs} ($x10^{-3}$ s^{-1}) for reaction of variable concentrations of 3-hexyne, N-benzylideneaniline, and benzophenone with 0.1 mM $[(IPr^*R)CuH]_2$ in toluene at 25 °C.

	**3-hexyne**		**N-benzylideneaniline**		**Benzophenone**	
	**20 mM**	**40 mM**	**10 mM**	**20 mM**	**10 mM**	**20 mM**
**$[(IPr^*Cl)CuH]_2$**	0.90	0.95		0.85		0.92
**$[(IPr^*Me)CuH]_2$**	7.2	7.5	7.1	7.2	7.8	7.8
**$[(IPr^*Et)CuH]_2$**	26	28		25		29
**$[(IPr^*OMe)CuH]_2$**	49	52		52		59

Reactions with [(IPr*Me)CuH]₂

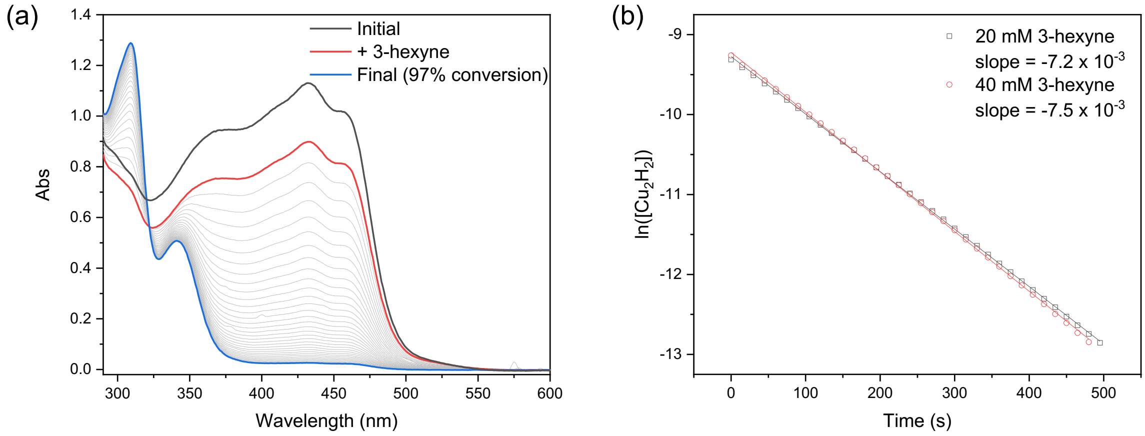

Figure S45. (a) UV-Visible spectra before (black) and after (red) addition of a stock solution of 3-hexyne to $[(IPr^*Me)CuH]_2$ in toluene at 25 °C (final $[(IPr^*Me)CuH]_2$ = 0.1 mM, [3-hexyne] = 20 mM). The gray lines show the reaction progress over five half-lives. (b) Analysis of kinetics data at 433 nm.

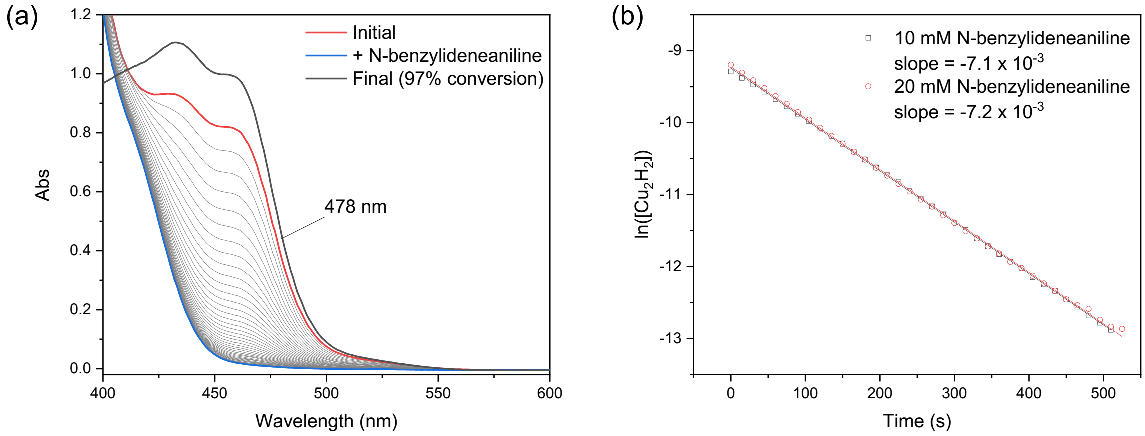

Figure S46. (a) UV-Visible spectra before (black) and after (red) addition of a stock solution of N-benzylideneaniline to $[(IPr^*Me)CuH]_2$ in toluene at 25 °C (final $[(IPr^*Me)CuH]_2$ = 0.1 mM, [N-benzylideneaniline] = 20 mM). The grey lines show the reaction progress over five half-lives. (b) Analysis of kinetics data at 478 nm.

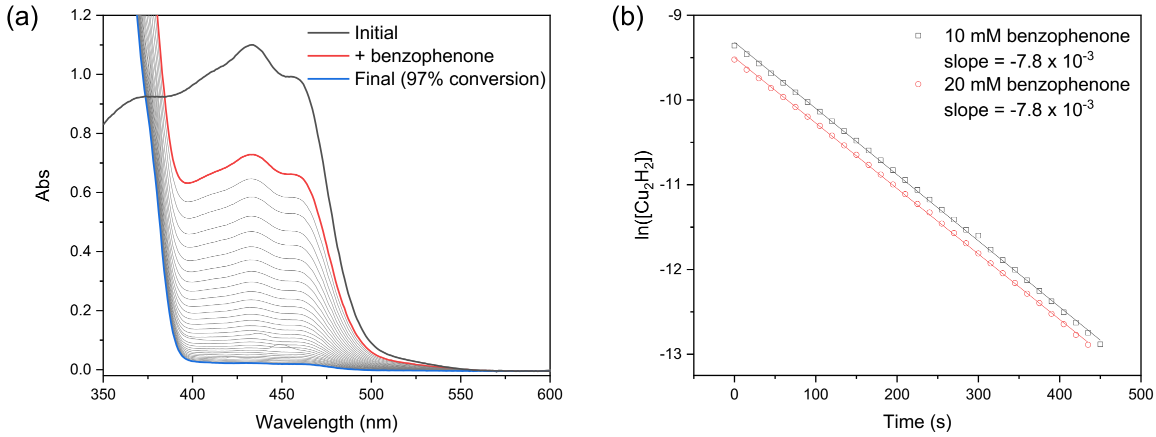

Figure S47. (a) UV-Visible spectra before (black) and after (red) addition of a stock solution of benzophenone to $[(IPr^*Me)CuH]_2$ in toluene at 25 °C (final $[(IPr^*Me)CuH]_2$ = 0.1 mM, [benzophenone] = 20 mM). The grey lines show the reaction progress over five half-lives. (b) Analysis of kinetics data at 433 nm.

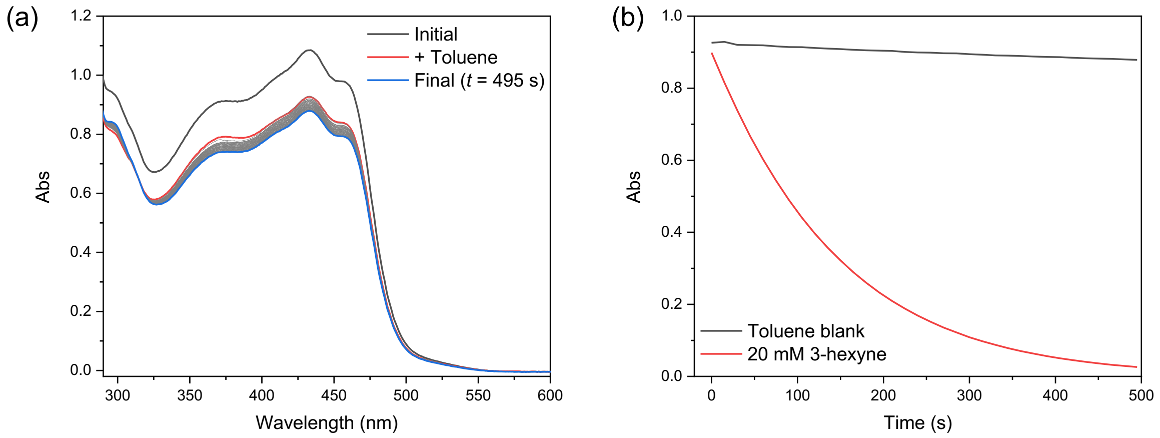

Figure S48. (a) UV-Visible spectra of 0.1 mM $[(IPr^*Me)CuH]_2$ under the conditions used for the kinetics experiments in Figures S45 – S47 in the absence of substrate. (b) Absorbance monitored at 433 nm for control (black) in comparison to reaction with 20 mM 3-hexyne (red).

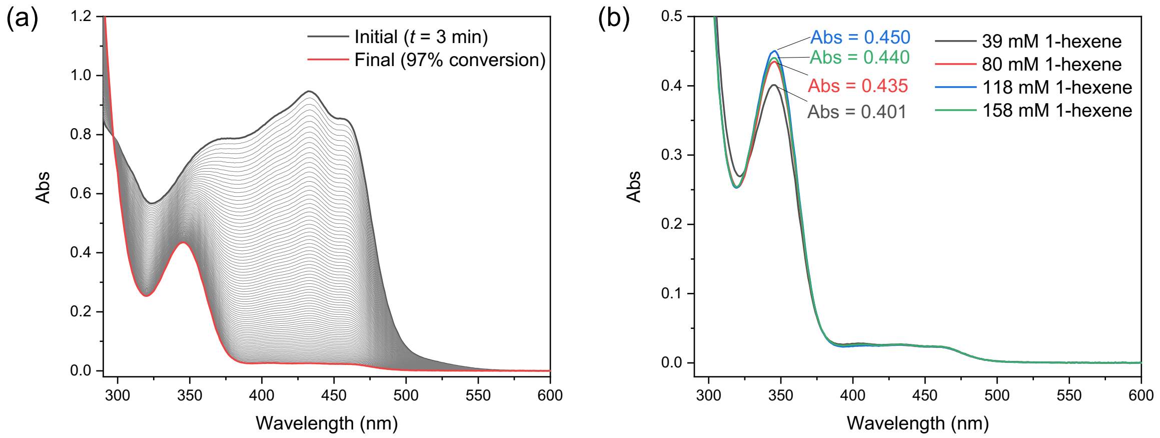

Figure S49. (a) UV-Visible spectra before after addition of a stock solution of 1-hexene to $[(IPr^*Me)CuH]_2$ in toluene at 25 °C (final $[(IPr^*Me)CuH]_2$ = 0.2 mM, [1-hexene] = 80 mM). The grey lines show the reaction progress over five half-lives. (b) Comparison of UV-Vis spectra for insertion product at different 1-hexene concentrations.

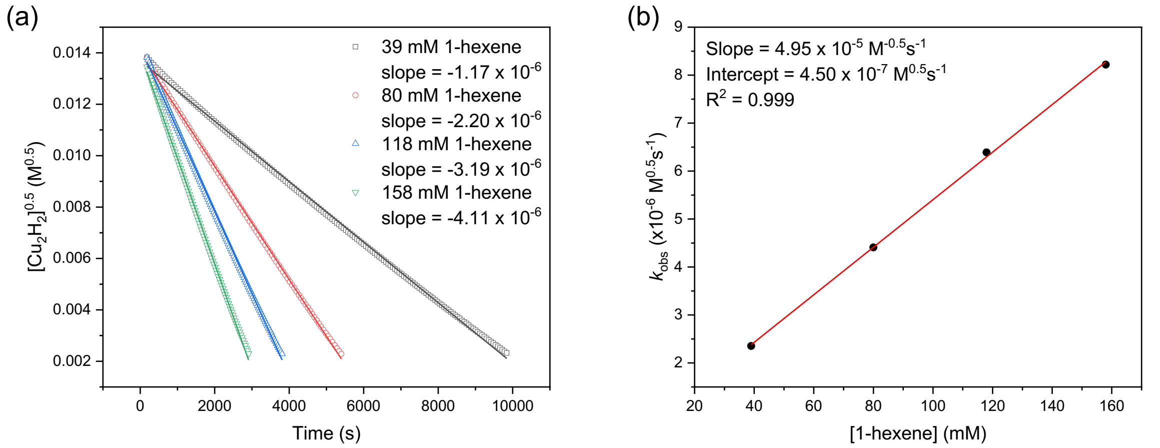

Figure S50. Analysis of kinetics data for the reaction of 0.2 mM $[(IPr^*Me)CuH]_2$ with 1-hexene. The reaction progress was followed for five half-lives at λ = 433 nm. (a) Determination of k_{obs} at variable 1-hexene concentrations. (b) Determination of $k_{3/2}$.

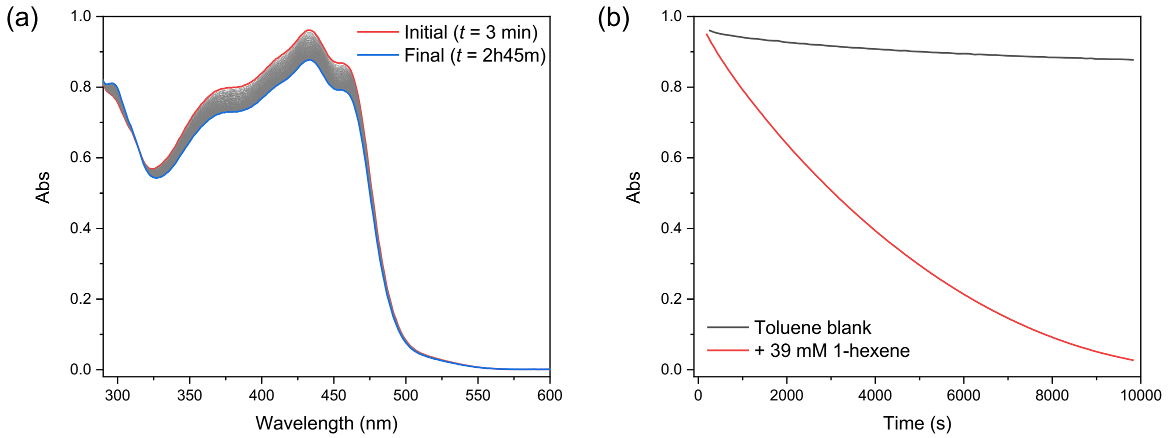

Figure S51. (a) UV-Visible spectra of 0.2 mM $[(IPr^*Me)CuH]_2$ after addition of toluene under the conditions used for the kinetics experiments in Figures S49 – S50, demonstrating minimal background decomposition on the timescale of the experiments. (b) Absorbance monitored at 433 nm for control (black) in comparison to reaction with 39 mM 1-hexene (red).

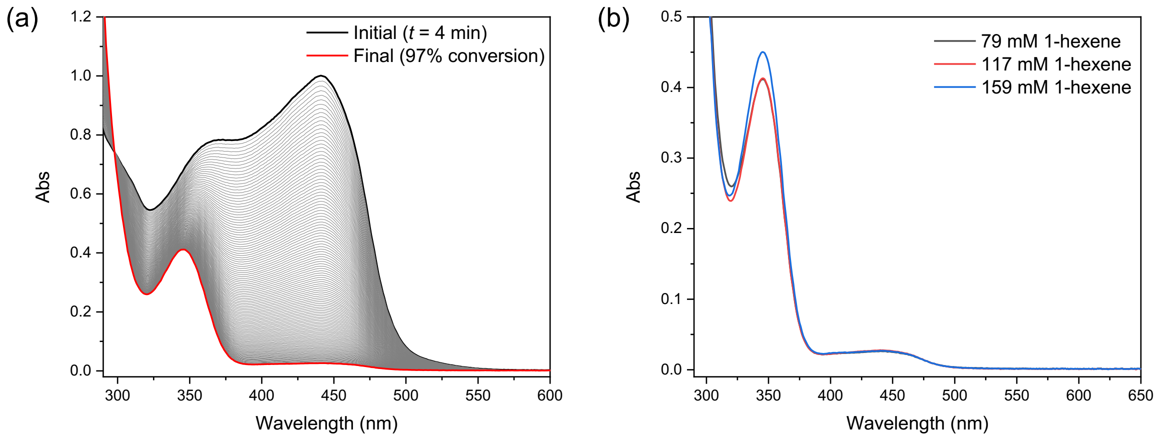

Figure S52. (a) UV-Visible spectra after addition of a stock solution of 1-hexene to $[(IPr^*Me)CuD]_2$ in toluene at 25 °C (final $[(IPr^*Me)CuD]_2$ = 0.2 mM, [1-hexene] = 79 mM). The grey lines show the reaction progress over five half-lives. (b) Comparison of UV-Vis spectra for insertion product at different 1-hexene concentrations at 97% conversion.

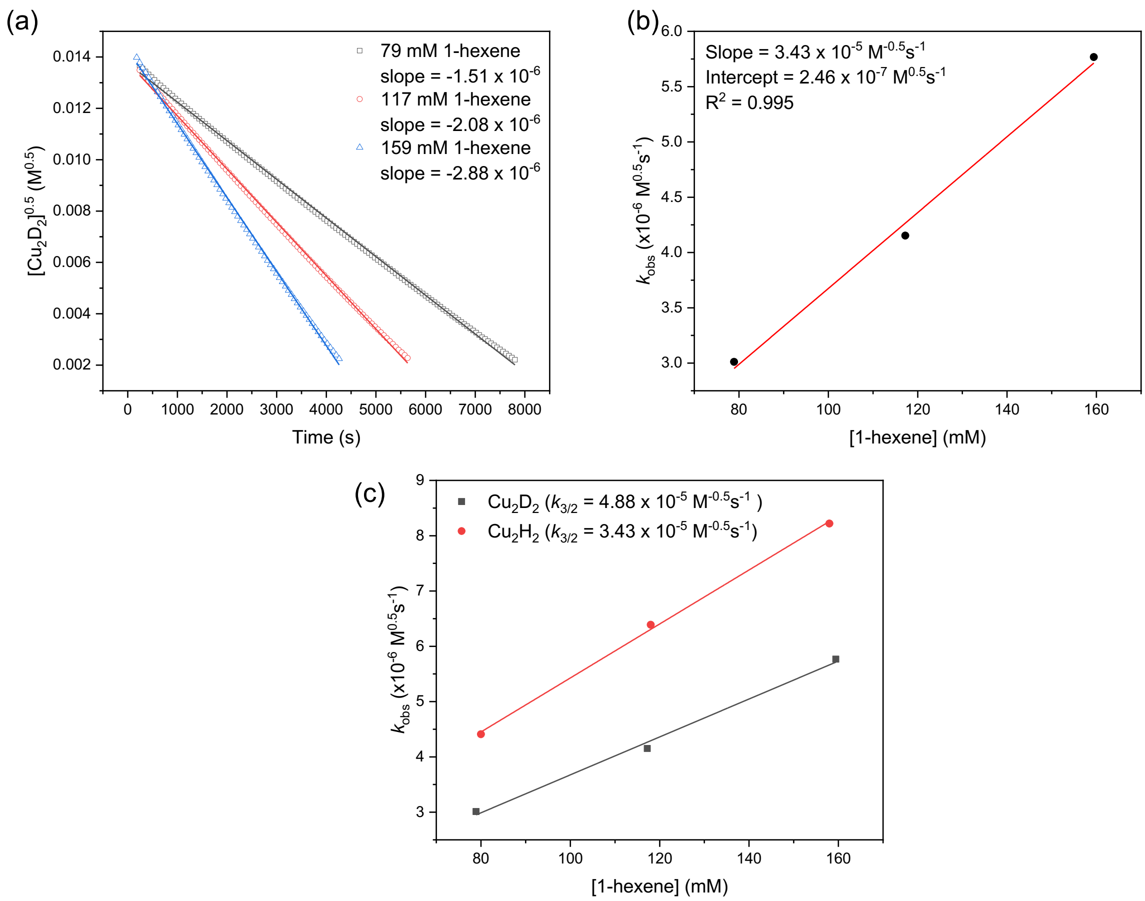

Figure S53. Analysis of kinetics data for the reaction of 0.2 mM $[(IPr^*Me)CuD]_2$ with 1-hexene. The reaction progress was followed for five half-lives at λ = 442 nm. (a) Determination of k_{obs} at variable 1-hexene concentrations. (b) Determination of $k_{3/2}$. (c) Comparison of $k_{3/2}$ for $[(IPr^*Me)CuH]_2$ and $[(IPr^*Me)CuD]_2$.

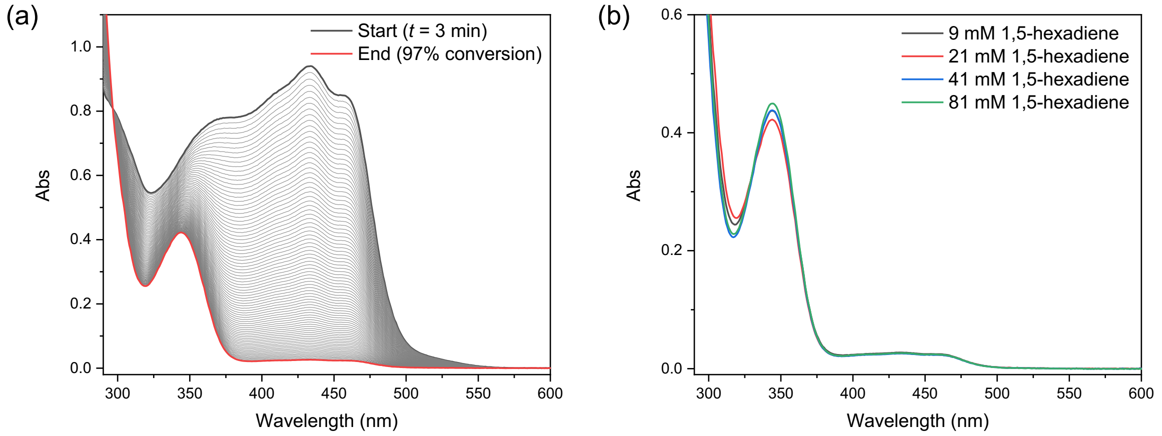

Figure S54. (a) UV-Visible spectra after addition of a stock solution of 1,5-hexadiene to [(IPr*Me)CuH]$_2$ in toluene at 25 °C (final [(IPr*Me)CuH]$_2$ = 0.2 mM, [1,5-hexadiene] = 21 mM). The grey lines show the reaction progress over five half-lives. (b) Comparison of UV-Vis spectra for the insertion product at different 1,5-hexadiene concentrations.

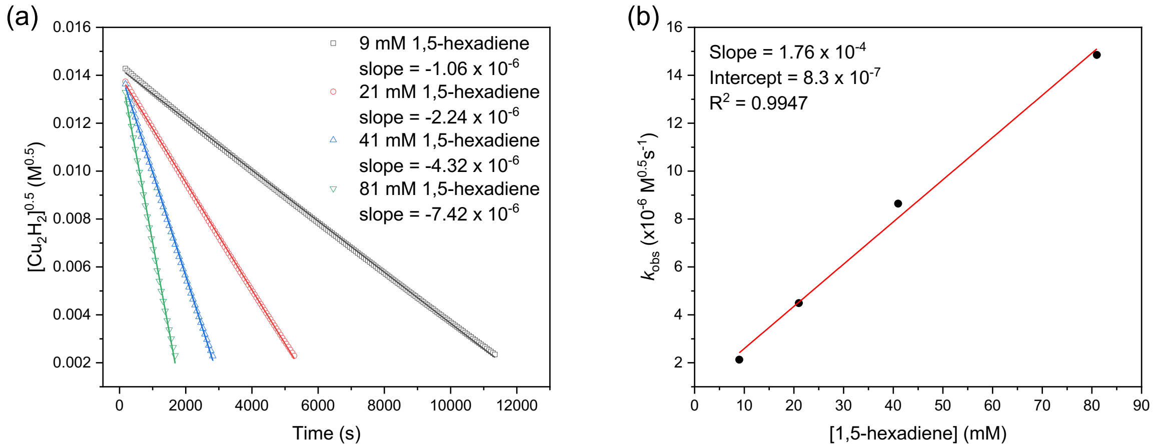

Figure S55. Analysis of kinetics data for the reaction of 0.2 mM [(IPr*Me)CuH]$_2$ with 1,5-hexadiene. The reaction progress was followed for five half-lives at λ = 433 nm. (a) Determination of k_{obs} at variable 1,5-hexadiene concentrations. (b) Determination of $k_{3/2}$.

Reactions with [(IPr*Et)CuH]$_2$

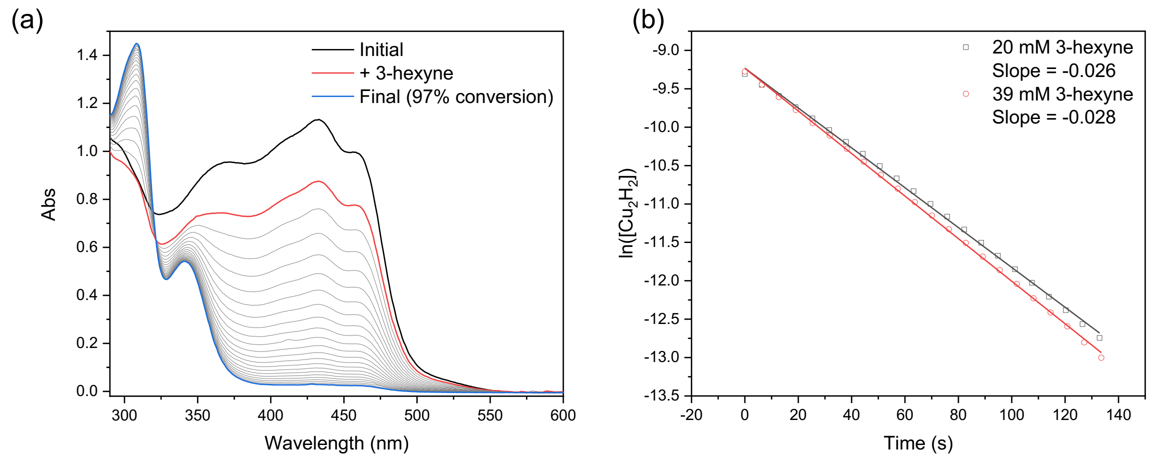

Figure S56. (a) UV-Visible spectra before (black) and after (red) addition of a stock solution of 3-hexyne to [(IPr*Et)CuH]$_2$ in toluene at 25 °C (final [(IPr*Et)CuH]$_2$ = 0.1 mM, [3-hexyne] = 20 mM). The grey lines show the reaction progress over five half-lives. (b) Analysis of kinetics data at 433 nm.

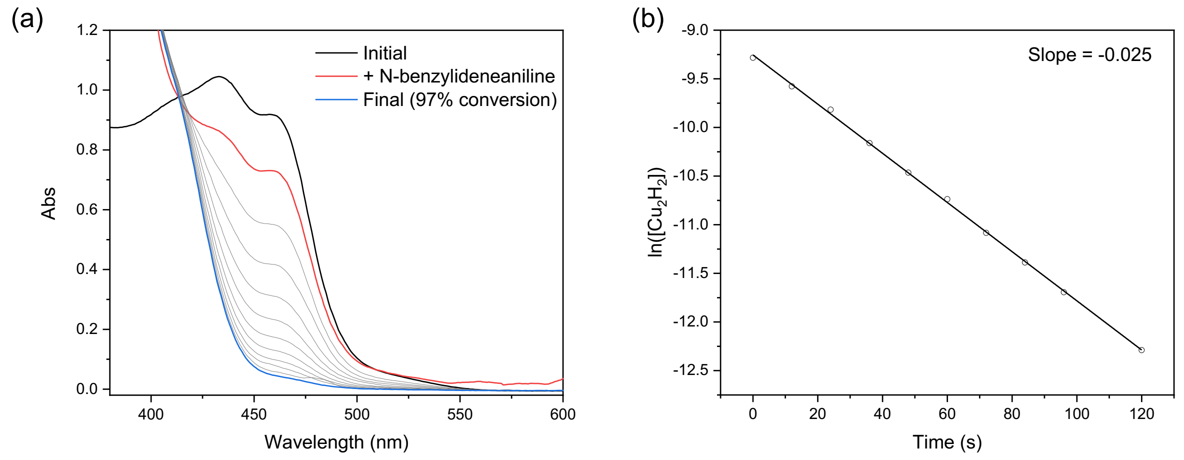

Figure S57. (a) UV-Visible spectra before (black) and after (red) addition of a stock solution of N-benzylideneaniline to [(IPr*Et)CuH]$_2$ in toluene at 25 °C (final [(IPr*Et)CuH]$_2$ = 0.1 mM, [N-benzylideneaniline] = 20 mM). The grey lines show the reaction progress over five half-lives. (b) Analysis of kinetics data at 433 nm.

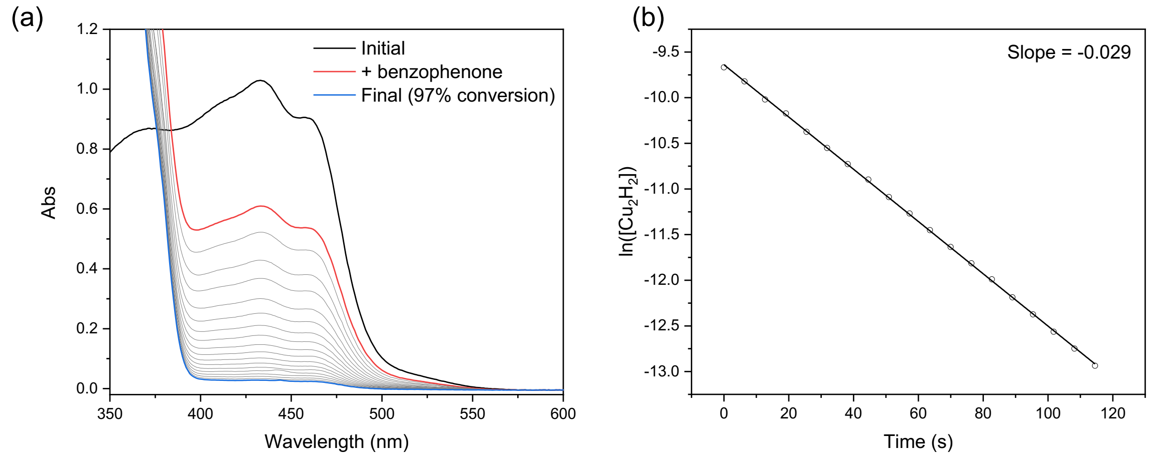

Figure S58. (a) UV-Visible spectra before (black) and after (red) addition of a stock solution of benzophenone to [(IPr*Et)CuH]$_2$ in toluene at 25 °C (final [(IPr*Et)CuH]$_2$ = 0.1 mM, [benzophenone] = 20 mM). The grey lines show the reaction progress over five half-lives. (b) Analysis of kinetics data at 433 nm.

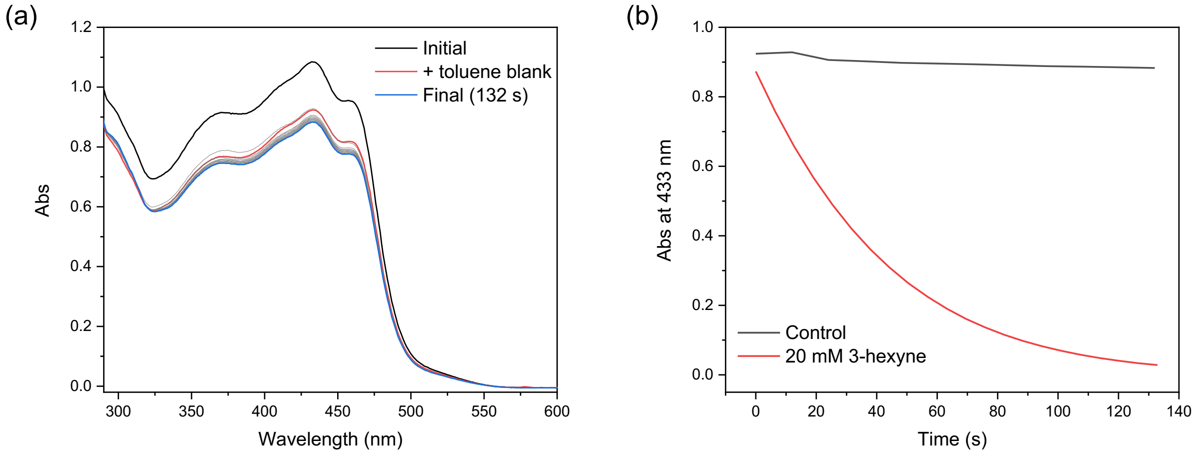

`

Figure S59. (a) UV-Visible spectra of 0.1 mM [(IPr*Et)CuH]$_2$ after addition of toluene under the conditions used for the kinetics experiments in Figures S56 – S58. (b) Absorbance monitored at 433 nm for control (black) in comparison to reaction with 20 mM 3-hexyne (red).

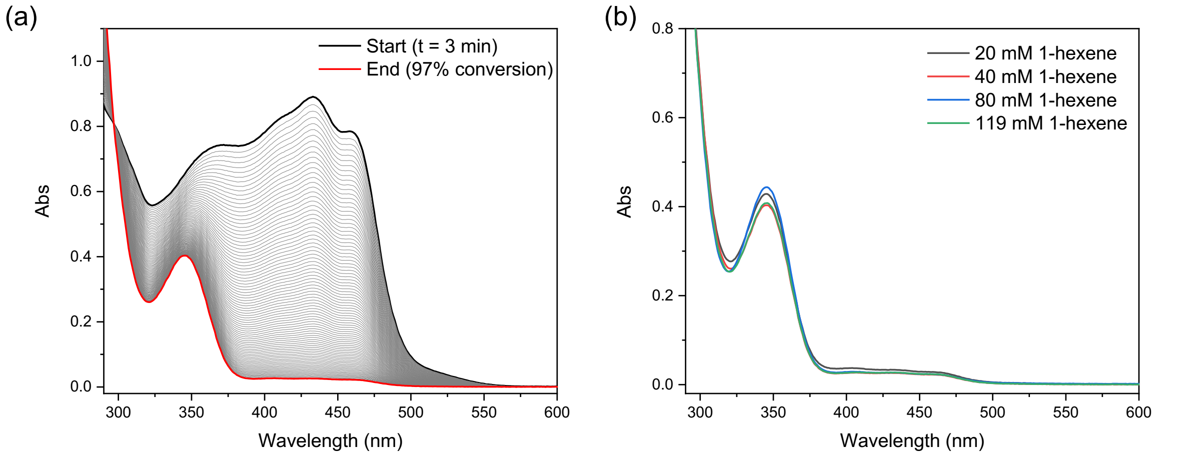

`

Figure S60. (a) UV-Visible spectra following the reaction of [(IPr*Et)CuH]$_2$ with 1-hexene in toluene at 25 °C (final [(IPr*Et)CuH]$_2$ = 0.2 mM, [1-hexene] = 40 mM). The grey lines show the reaction progress over five half-lives. (b) Comparison of UV-Vis spectra for insertion product at different 1-hexene concentrations.

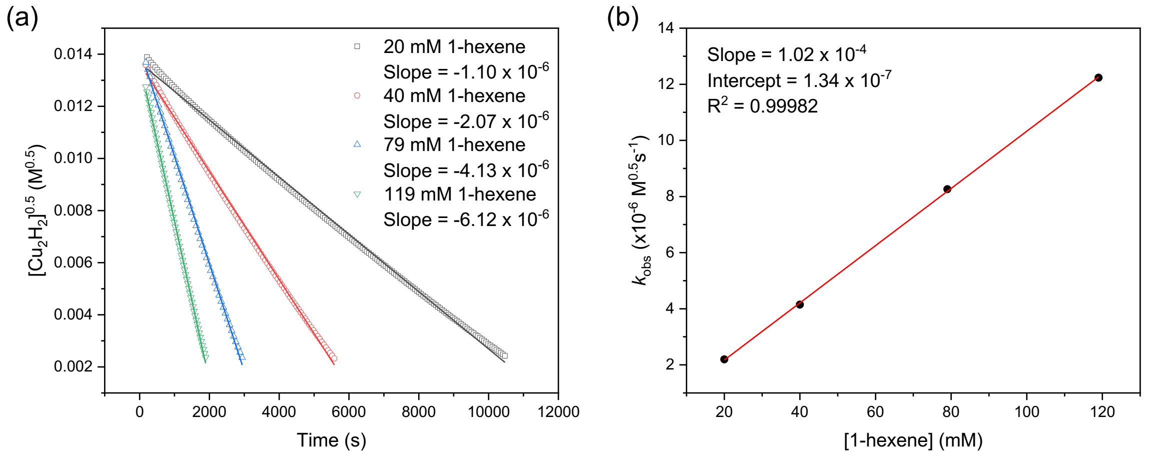

Figure S61. Analysis of kinetics data for the reaction of 0.2 mM [(IPr*Et)CuH]$_2$ with 1-hexene. The reaction progress was followed for five half-lives at λ = 433 nm. (a) Determination of k_{obs} at variable 1-hexene concentrations from plots of square root of copper hydride concentration vs time. (b) Determination of $k_{3/2}$.

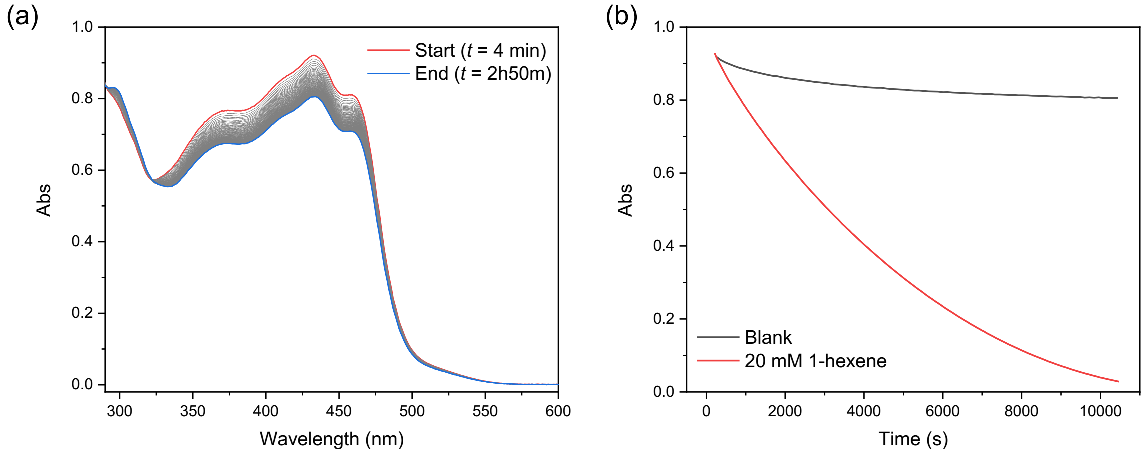

Figure S62. UV-Visible spectra of 0.2 mM [(IPr*Et)CuH]$_2$ after addition of toluene under the conditions used for the kinetics experiments in Figures S60 – S61.(b) Absorbance monitored at 433 nm for control (black) in comparison to reaction with 20 mM 1-hexene (red).

Reactions with [(IPr*^{t}Bu)CuH]$_2$

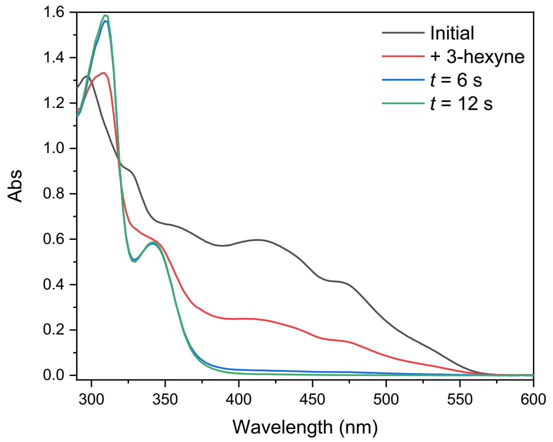

Figure S63. UV-Visible spectra showing the reaction of 0.1 mM [(IPr*^{t}Bu)CuH]$_2$ with 20 mM 3-hexyne. Reliable kinetics data could not be obtained because the reaction is complete within ~6 seconds.

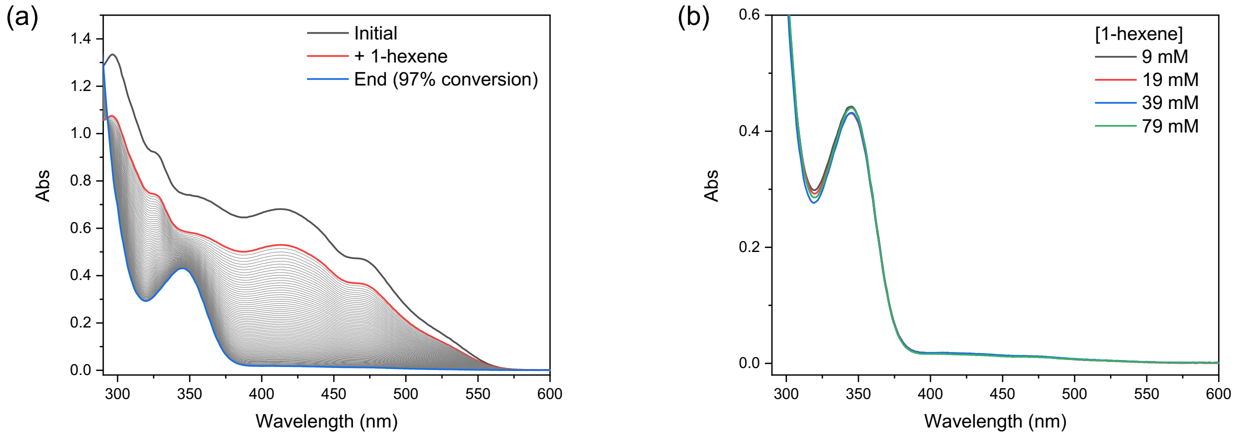

Figure S64. (a) UV-Visible spectra before (black) and after (red) addition of a stock solution of 1-hexene to [(IPr*^{t}Bu)CuH]$_2$ in toluene at 25 °C (final [(IPr*^{t}Bu)CuH]$_2$ = 0.2 mM, [1-hexene] = 19 mM). The grey lines show the reaction progress over five half-lives. (b) Comparison of UV-Vis spectra for insertion product at different 1-hexene concentrations. The nearly identical spectra demonstrate that negligible decomposition of [(IPr*^{t}Bu)CuH]$_2$ occurs under the reaction conditions.

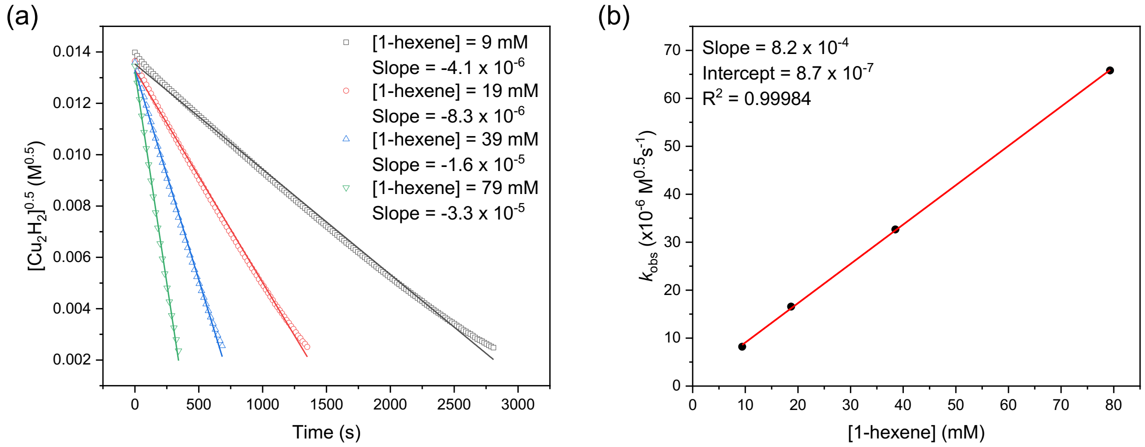

Figure S65. Analysis of kinetics data for the reaction of 0.2 mM [(IPr*^{t}Bu)CuH]$_2$ with 1-hexene. The reaction progress was followed for five half-lives at λ = 412 nm. (a) Determination of k_{obs} at variable 1-hexene concentrations from plots of square root of copper hydride concentration vs time. (b) Determination of $k_{3/2}$.

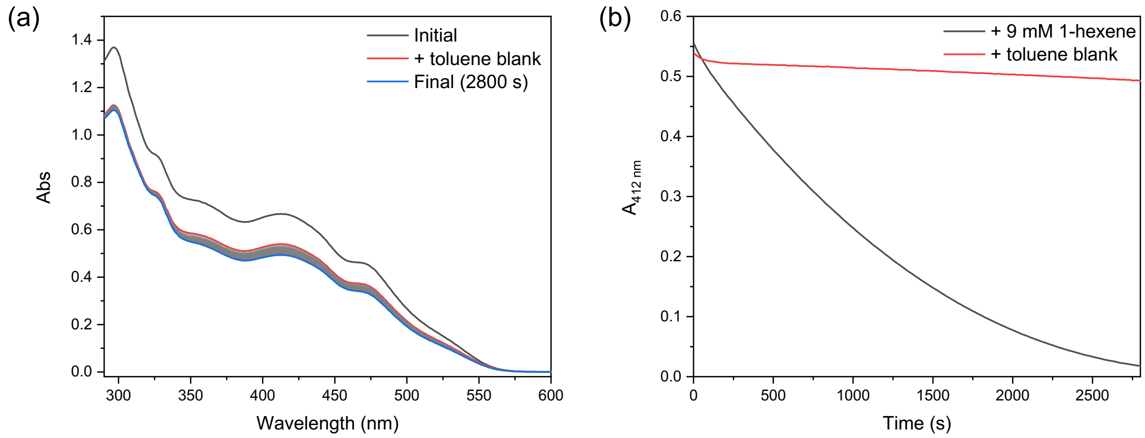

Figure S66. (a) UV-Visible spectra of 0.2 mM [(IPr*^{t}Bu)CuH]$_2$ after addition of toluene under the conditions used for the kinetics experiments in Figures S64 – S65, demonstrating minimal background decomposition on the timescale of the insertion experiments. (b) Absorbance monitored at 412 nm for control (red) in comparison to reaction with 9 mM 1-hexene (black)

Reactions with [(IPr*OMe)CuH]$_2$

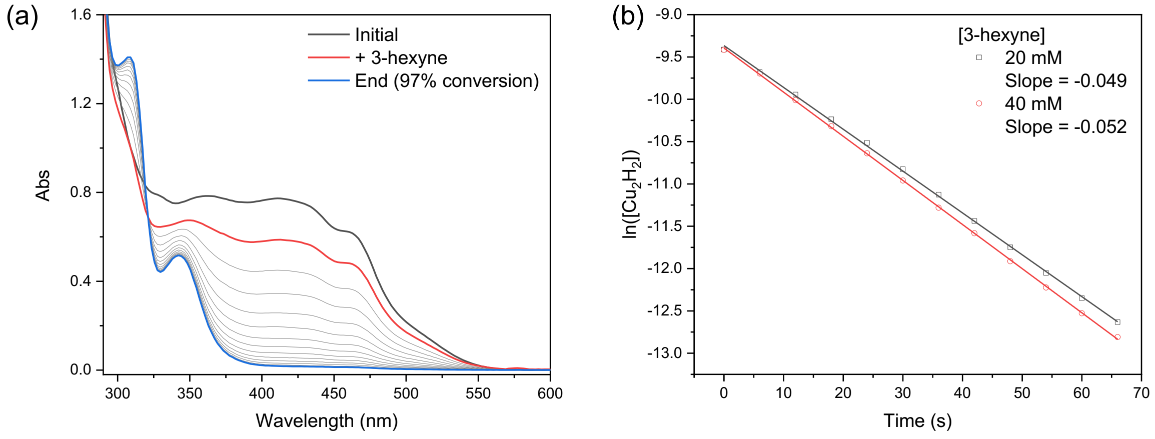

Figure S67. (a) UV-Visible spectra before (black) and after (red) addition of a stock solution of 3-hexyne to [(IPr*OMe)CuH]$_2$ in toluene at 25 °C (final [(IPr*OMe)CuH]$_2$ = 0.1 mM, [3-hexyne] = 20 mM). The grey lines show the reaction progress over five half-lives. (b) Analysis of kinetics data for reaction of 0.1 mM [(IPr*OMe)CuH]$_2$ with 20 mM (black) or 40 mM (red) 3-hexyne.

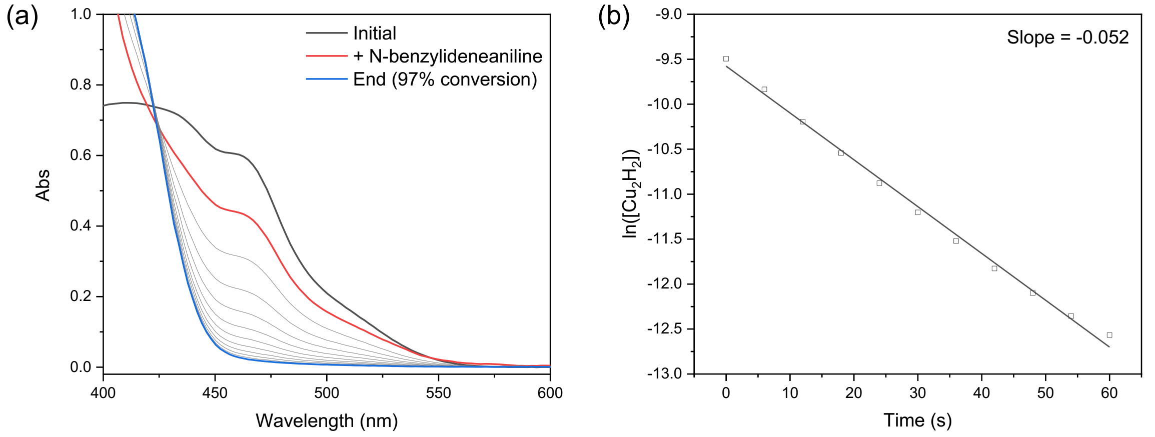

Figure S68. (a) UV-Visible spectra before (black) and after (red) addition of a stock solution of N-benzylideneaniline to [(IPr*OMe)CuH]$_2$ in toluene at 25 °C (final [(IPr*OMe)CuH]$_2$ = 0.1 mM, [N-benzylideneaniline] = 20 mM). The grey lines show the reaction progress over five half-lives. (b) Analysis of kinetics data for reaction of 0.1 mM [(IPr*OMe)CuH]$_2$ with 20 mM N-benzylideneaniline. The reaction was monitored at 469 nm due to interference from the substrate at 433 nm.

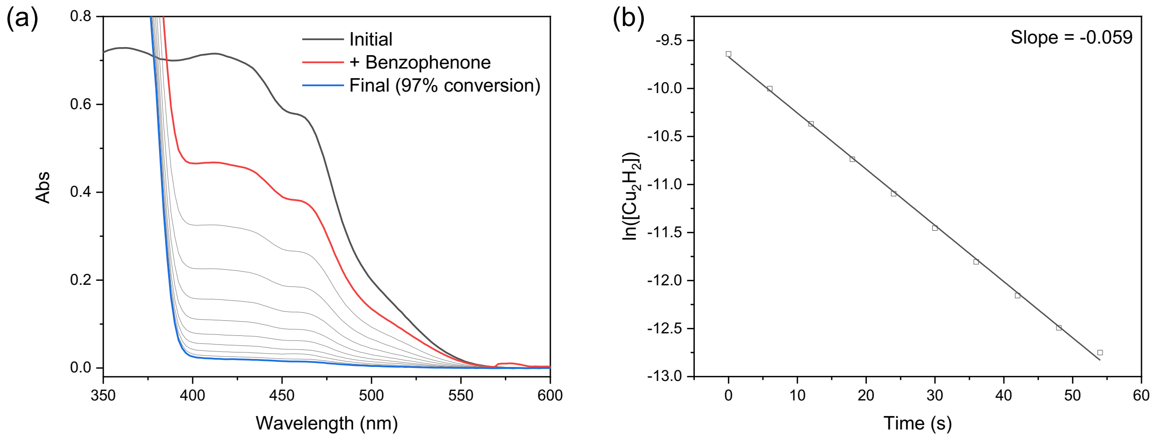

Figure S69. (a) UV-Visible spectra before (black) and after (red) addition of a stock solution of benzophenone to [(IPr*OMe)Cu(μ-H)]$_2$ in toluene at 25 °C (final [(IPr*OMe)CuH]$_2$ = 0.1 mM, [benzophenone] = 20 mM). The grey lines show the reaction progress over five half-lives. (b) Analysis of kinetics data for reaction of 0.1 mM [(IPr*OMe)CuH]$_2$ with 20 mM benzophenone.

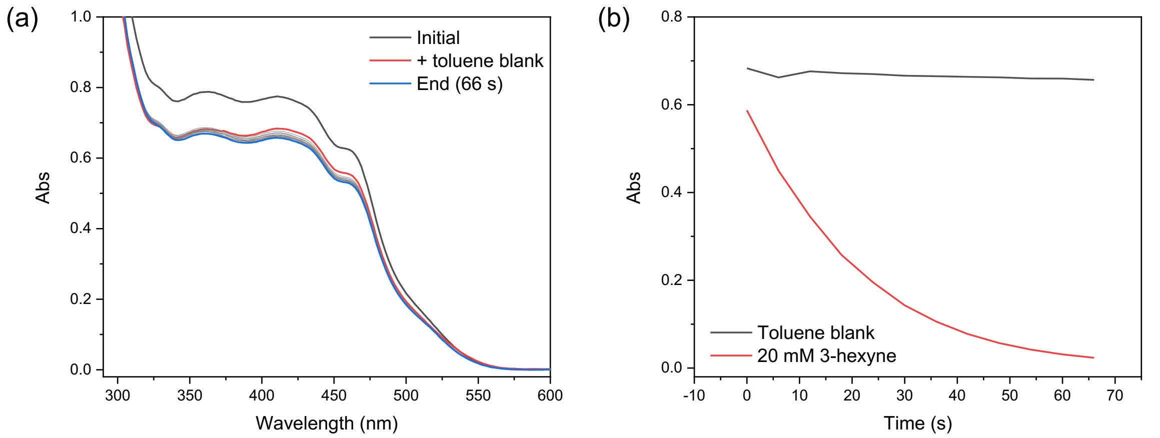

Figure S70. (a) UV-Visible spectra of 0.1 mM [(IPr*OMe)CuH]$_2$ after addition of toluene under the conditions used for the kinetics experiments in Figures S67 – S69. (b) Absorbance monitored at 412 nm for control (black) in comparison to reaction with 20 mM 3-hexyne (red).

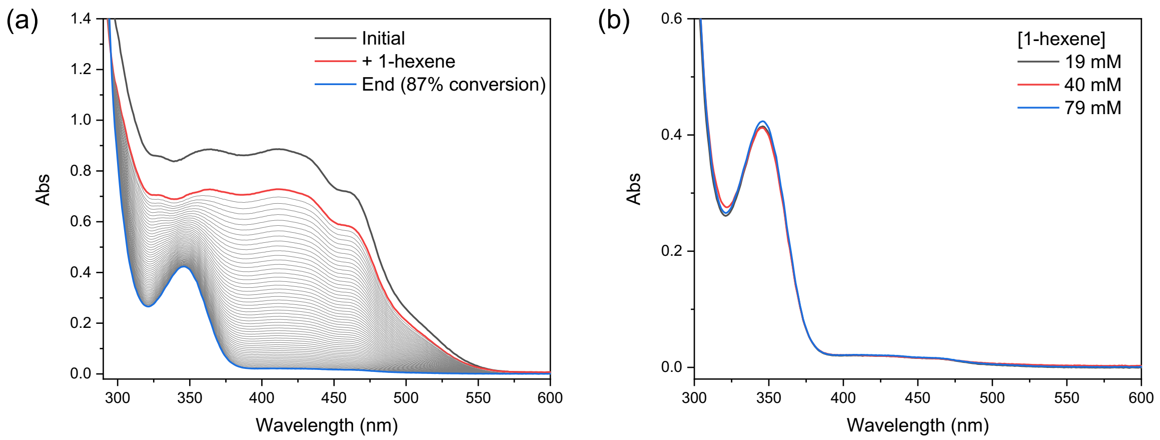

Figure S71. (a) UV-Visible spectra before (black) and after (red) addition of a stock solution of 1-hexene to [(IPr*OMe)CuH]$_2$ in toluene at 25 °C (final [(IPr*OMe)CuH]$_2$ = 0.2 mM, [1-hexene] = 79 mM). The grey lines show the reaction progress over five half-lives. (b) Comparison of UV-Vis spectra for insertion product at different 1-hexene concentrations.

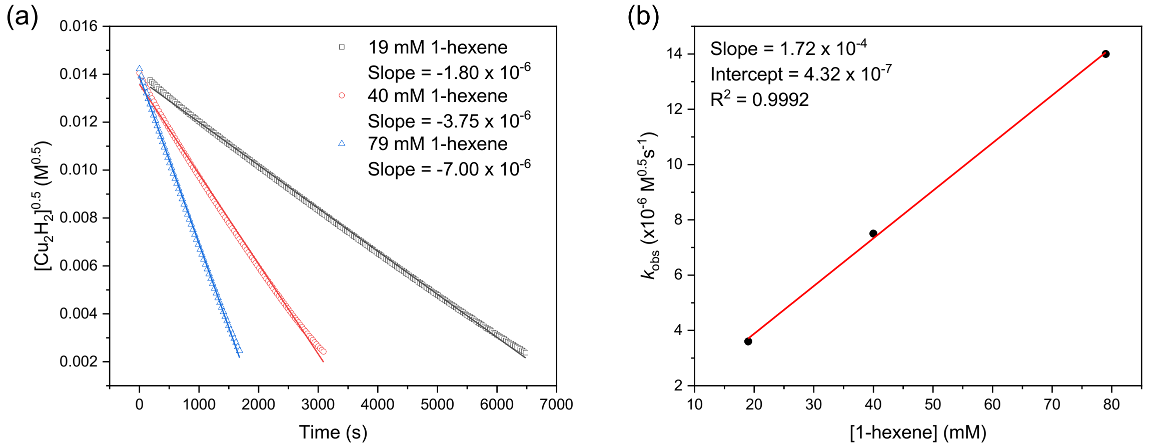

Figure S72. Analysis of kinetics data for the reaction of 0.2 mM [(IPr*OMe)CuH]$_2$ with 1-hexene. The reaction progress was followed for five half-lives at λ = 412 nm. (a) Determination of k_{obs} at variable 1-hexene concentrations from plots of square root of copper hydride concentration vs time. (b) Determination of $k_{3/2}$.

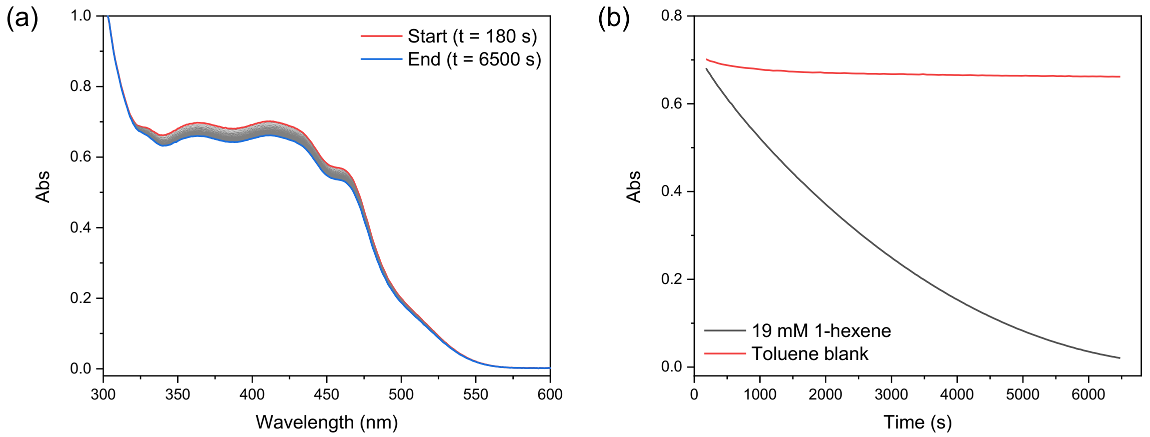

Figure S73. (a) UV-Visible spectra of 0.2 mM [(IPr*OMe)CuH]$_2$ after addition of toluene under the conditions used for the kinetics experiments in Figures S71 – S72, demonstrating minimal background decomposition on the timescale of the experiments. (b) Absorbance monitored at 412 nm for control (red) in comparison to reaction with 19 mM 1-hexene (black).

Reactions with [(IPr*Cl)CuH]$_2$

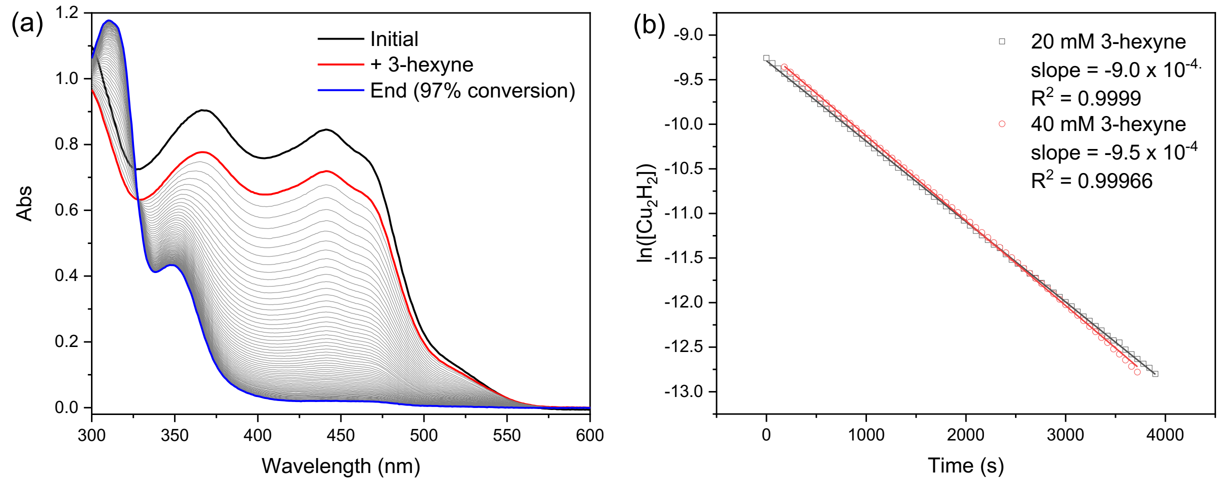

Figure S74. (a) UV-Vis spectra following the reaction of 0.1 mM [(IPr*Cl)CuH]$_2$ with 20 mM 3-hexyne in toluene. (b) First-order fits for the reaction of [(IPr*Cl)CuH]$_2$ with 20 mM (black) or 40 mM (red) 3-hexyne monitored at 441 nm.

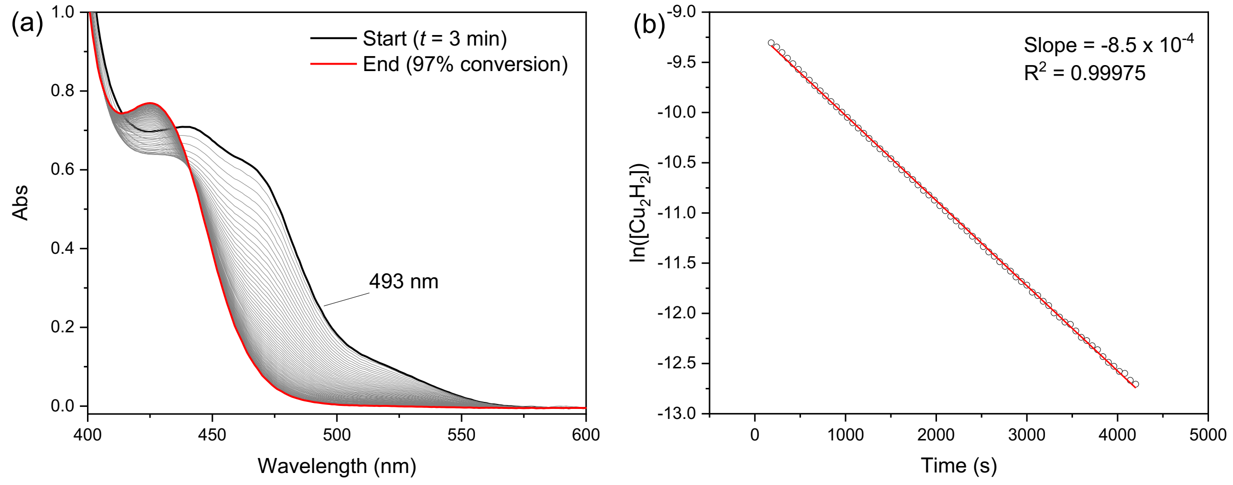

Figure S75. (a) UV-Vis spectra following the reaction of 0.1 mM [(IPr*Cl)CuH]$_2$ with 20 mM N-benzylideneaniline in toluene. (b) First-order fit for the reaction of [(IPr*Cl)CuH]$_2$ with 20 mM N-benzylideneaniline in toluene. The reaction was monitored at 493 nm due to interference from the substrate at 441 nm.

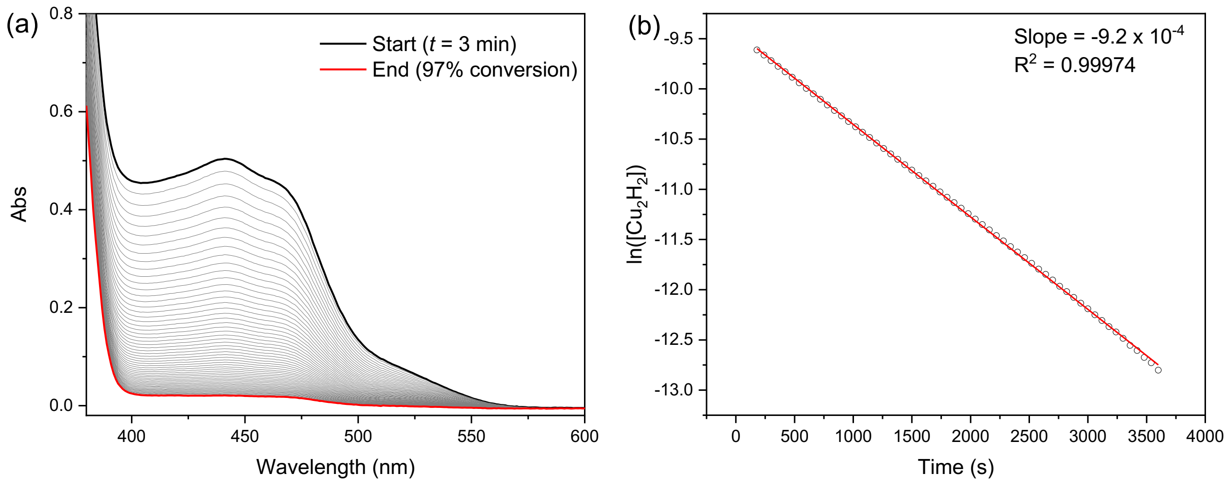

Figure S76. (a) UV-Vis spectra following the reaction of 0.1 mM [(IPr*Cl)CuH]$_2$ with 20 mM benzophenone in toluene. (b) First-order fit for the reaction of [(IPr*Cl)CuH]$_2$ with 20 mM benzophenone in toluene monitored at 441 nm.

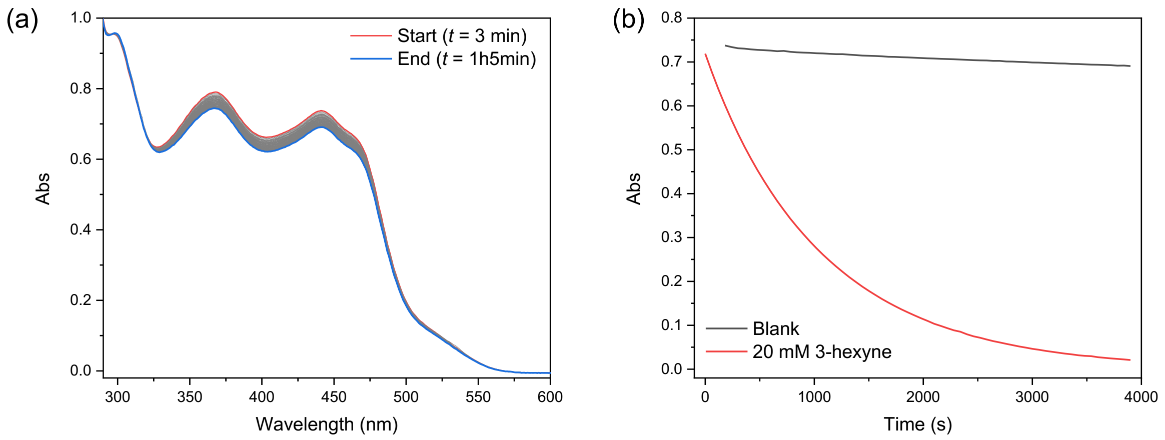

Figure S77. (a) UV-Visible spectra of 0.1 mM [(IPr*Cl)CuH]$_2$ after addition of toluene under the conditions used for the kinetics experiments in Figures S74 – S76. (b) Absorbance monitored at 441 nm for control (black) in comparison to reaction with 20 mM 3-hexyne (red).

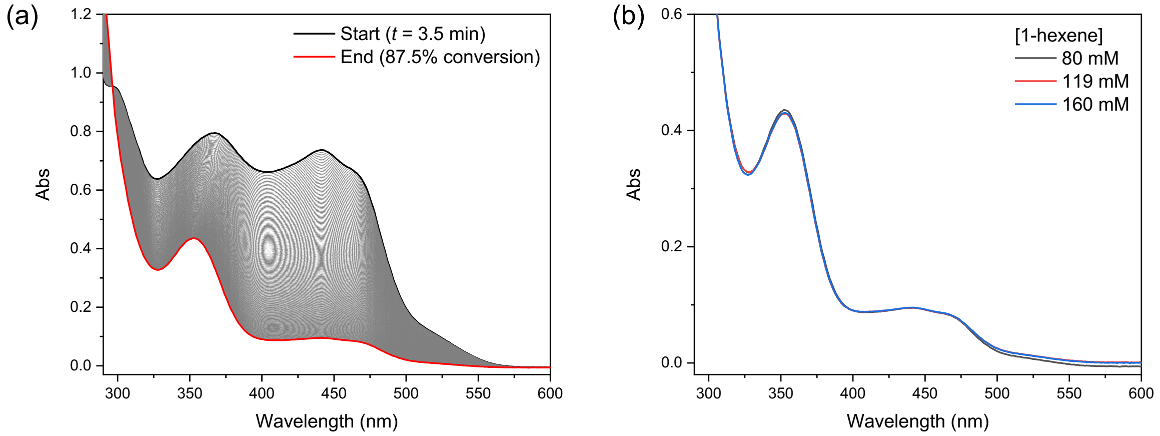

Figure S78. (a) UV-Visible spectra before (black) and after (red) addition of a stock solution of 1-hexene to [(IPr*Cl)CuH]$_2$ in toluene at 25 °C (final [(IPr*Cl)CuH]$_2$ = 0.2 mM, [1-hexene] = 80 mM). The grey lines show the reaction progress over three half-lives. (b) Comparison of UV-Vis spectra for insertion product at three half-lives for different 1-hexene concentrations.

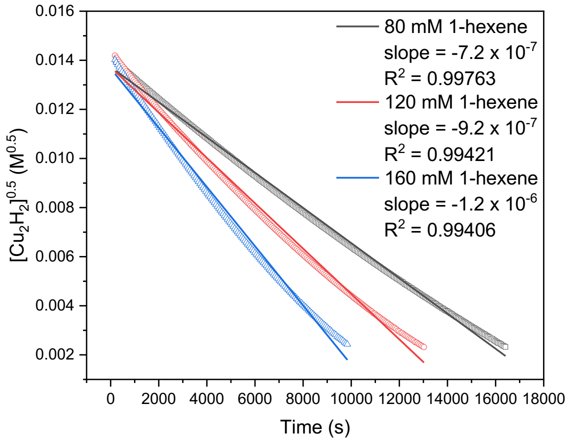

Figure S79. Attempted analysis of kinetics data for the reaction of 0.2 mM [(IPr*Cl)CuH]$_2$ with 1-hexene followed for five half-lives at λ = 441 nm. Because significant deviation from linearity is observed, particularly at the end of the reaction, the data were re-analyzed at three half-lives (see Figure S80).

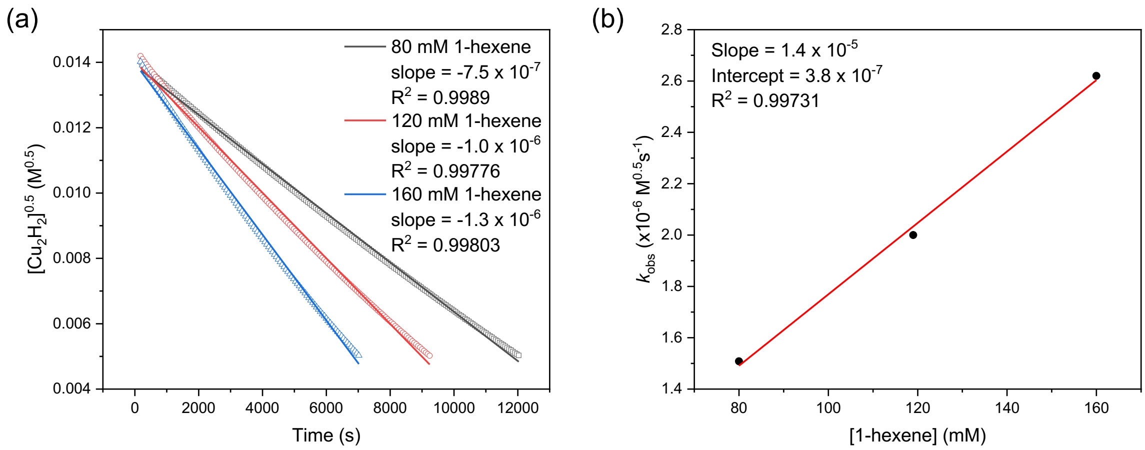

Figure S80. Analysis of kinetics data for the reaction of 0.2 mM [(IPr*Cl)CuH]$_2$ with 1-hexene. The reaction progress was followed for three half-lives at λ = 441 nm. (a) Determination of k_{obs} at variable 1-hexene concentrations from plots of square root of copper hydride concentration vs time. (b) Determination of $k_{3/2}$.

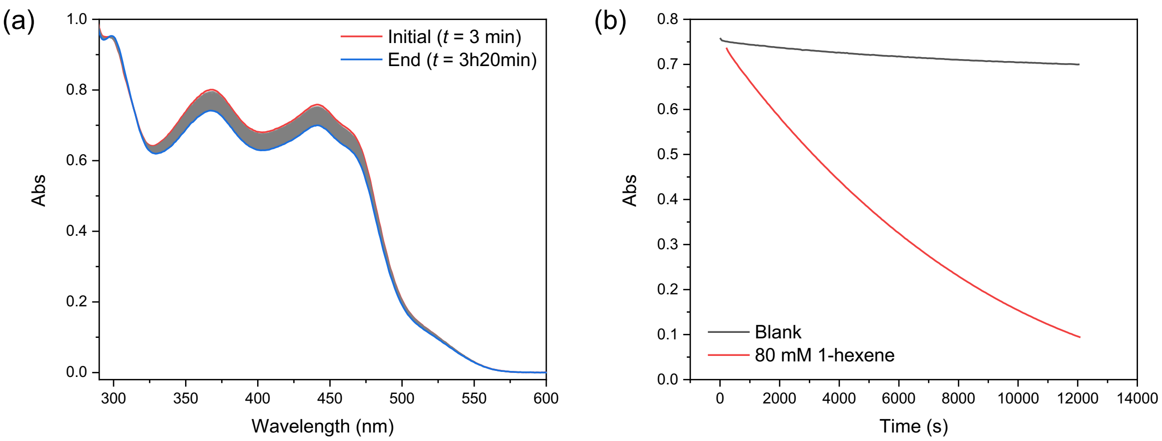

Figure S81. (a) UV-Visible spectra of 0.2 mM [(IPr*Cl)CuH]$_2$ after addition of toluene under the conditions used for the kinetics experiments in Figures S78 – S80. (b) Absorbance monitored at 441 nm for the control experiment (black) in comparison to the reaction with 80 mM 1-hexene (red).

Competition experiments for reactions of $[(IPr^*Me)CuH]_2$ with carbonyls, imines, and alkynes

General Procedure

Inside a nitrogen-filled glovebox, dry C_6D_6 (0.75 mL) was added to equimolar (25.5 mmol) amounts of the respective substrates to afford a clear homogeneous solution after stirring for 5 minutes. This solution was then added to solid $[(IPr^*Me)CuH]_2$ (5.00 mg, 2.55 mmol) in a 3 mL scintillation vial. The reaction mixture was stirred for 0.5 h at 25 ºC until the solution became colorless, and no undissolved material was observed. The solution was transferred to a J. Young NMR tube and ^1H NMR spectroscopy was performed to quantify the corresponding inserted products. ^1H NMR data for $[(IPr^*Me)Cu\text{-}OCH(Ph)_2]$ and $[(IPr^*Me)Cu\text{-}OCH(C_6H_4\text{-}4\text{-}OMe)_2]$ in C_6D_6 have been reported.[13]

Control experiments

Control experiments were also performed to assess possible reversible processes. First, the distribution of inserted products of all competition reactions remained unchanged for 48 h at 25 ºC. Second, initial *in situ* formation of $[(IPr^*)Cu\text{-}N(Ph)CH_2(C_6H_4)\text{-}4\text{-}NMe_2]$ followed by addition of PhC≡CPh did not produce [(IPr*)Cu-C(Ph)=CH(Ph)], even after 48 h. These results provide evidence against β-hydride elimination from the Cu-anilide or hydride transfer from the Cu-anilide via a mechanism akin to that of Meerwin-Poodorf-Verley (MPV) reduction[15] where a discrete Cu-H intermediate is not formed.

DFT calculations

Computational methods

The geometries were optimized in the gas phase at the density functional theory (DFT)[16] level with the hybrid B3LYP exchange-correlation functional.[17, 18] The DFT-optimized DZVP2 basis sets[19] were used for H, C, N, O and Cl atoms and aug-cc-pVDZ-PP basis set was used for Cu.[20] Vibrational frequencies were calculated to show that the structures were minima. Single point calculations using the B3LYP optimized geometries were performed at the M06[21] and ωB97X-D levels.[22, 23] These functionals were chosen as they have been used to predict thermodynamic properties of similar systems.[24] The cartesian coordinates for the optimized geometries have been provided as a separate file. The calculations were performed using the Gaussian16 program system.[25]

The quasiharmonic approximation from Truhlar and co-workers was used to correct the entropy associated with low-frequency vibrational modes. In this case, all harmonic frequencies below 100 cm^{-1} were raised to 100 cm^{-1} before evaluation of the vibrational component to the entropy.[26]

Using the gas phase optimized geometries, the solvation free energies in benzene at 298 K were calculated using the self-consistent reaction field (SCRF) approach[27] with the COSMO (B3LYP//DZVP2/aug-cc-pVDZ-PP) parameters[28, 29] as implemented in Gaussian 16. The Gibbs free energy in benzene solution, ΔG_{sol}, was calculated from:

$$\Delta G_{sol} = \Delta G_{g,298K} + \Delta\Delta G_{C6H6}$$

where $\Delta G_{g,298K}$ is the gas phase free energy and $\Delta\Delta G_{C6H6}$ is benzene solvation free energy. A dielectric constant of 2.2706 corresponding to that of bulk benzene was used in the COSMO calculations.

KIEs were calculated from thermodynamic transition state theory:

$$k = (k_B T/h)*\exp(-\Delta G^{\ddagger}/RT)$$

with $-\Delta G^{\ddagger}$ calculated at the ωB97XD//B3LYP level. Note that the solvent contributions calculate in the SCRF approximation used in the calculations, so this is determined by frequencies in the gas phase.

The Natural Population Analysis (NPA) results based on the Natural Bond Orbitals (NBOs)[30, 31] using NBO7[32, 33] are calculated using Gaussian16.

The B3LYP optimized geometries were used for the time dependent-DFT (TD-DFT) calculations[34, 35], performed to analyze the UV-Visible spectra in benzene.

The calculations were performed on our local UA Opteron- and Xeon-based Linux clusters.

Analysis of DFT-optimized (IPr*R)Cu-H structures

The calculated geometric parameters are in reasonable agreement with the XRD structures, and are similar for the staggered and eclipsed conformers (Table S6). Also in agreement with experiment, there is very little change in geometry as a function of the *para* substituent. The staggered and eclipsed conformers are predicted to be close in energy at 298 K in benzene (Tables S7 and S8). The single point calculations also indicate that the dimer is more stable than the monomer if a functional is chosen that allows for the steric interactions in the dimer to be treated properly to allow for additional stabilization (by dispersion interactions between the ligands).

The approximately equal Cu-H distances in $[(IPr^*R)CuH]_2$ suggest that each H is shared between the two Cu atoms. As a comparison to a simple bridged species, the calculated Cu-H bond length in $[Cu(\mu\text{-}H)]_2$ (*i.e.* in the absence of the NHC ligand) is 1.748 Å at the same level of theory, which is similar to that of $[(IPr^*R)CuH]_2$. In contrast, for the diatomic CuH, in which the Cu-H has 'covalent bond' character and the hydrogen is not shared with another Cu center, the Cu-H distance is shorter (1.462 Å). The Cu-Cu distance in $[(IPr^*R)CuH]_2$ is elongated compared to the calculated Cu-Cu distance in $[Cu(\mu\text{-}H)]_2$ in D_{2h} symmetry (2.170 Å). These calculations suggest that the Cu-H bonds in the dimer can be described as 3-center-2-electron bonds where the H atoms act as hydride bridges.

Table S6. Comparison of key geometric parameters for XRD and DFT-optimized structures for $[(IPr^*R)CuH]_2$.

R	Structure	Cu-Cu (Å)	Avg. C_{NHC}-Cu (Å)	Avg. C_{NHC}-Cu-Cu (°)	Carbene twist angle[a] (°)	Avg. Cu-H (Å)
**H**	DFT staggered	2.380	1.947	180	87	1.72
	DFT eclipsed	2.374	1.929	174	22	1.71
**Me**	DFT staggered	2.381	1.949	180	88	1.72
	DFT eclipsed	2.377	1.932	177	20	1.71
**Cl**	XRD (staggered)	2.356	1.909	177	90	1.80
	DFT staggered	2.378	1.946	179	87	1.72
	DFT eclipsed	2.375	1.929	175	20	1.71
**OMe**	XRD (staggered)	2.353	1.930	176	89	1.74
	DFT staggered	2.385	1.950	179	86	1.72
	DFT eclipsed	2.377	1.931	180	14	1.71

[a] Acute angle between the planes defined by the Cu-imidazole units.

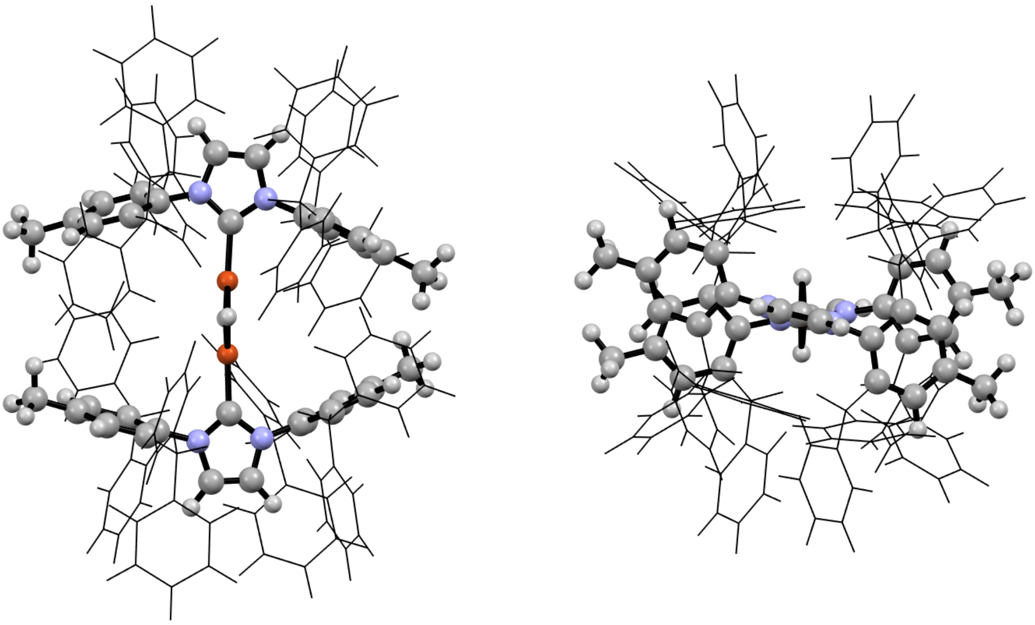

Figure S82. DFT-optimized structures of [(IPr*Me)CuH]$_2$ in the eclipsed geometry, shown in two different orientations. On the left, the Cu-Cu vector lies in the plane of the page, and on the right the Cu-Cu vector is perpendicular to the page. The $CHPh_2$ groups are shown in wireframe representation.

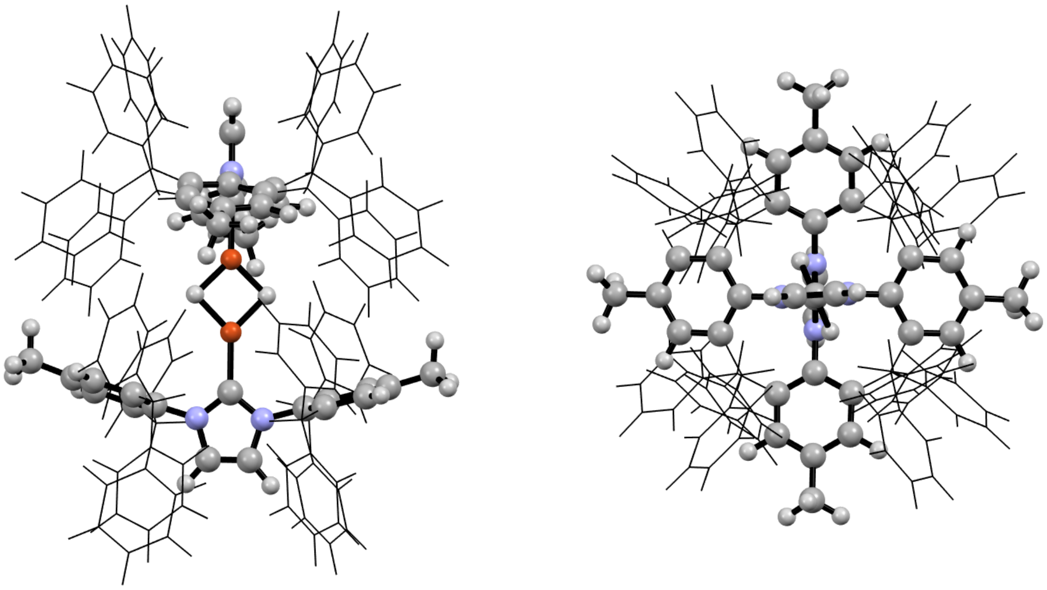

Figure S83. DFT-optimized structures of [(IPr*Me)CuH]$_2$ in the staggered geometry, shown in two different orientations. On the left, the Cu-Cu vector lies in the plane of the page, and on the right the Cu-Cu vector is perpendicular to the page. The $CHPh_2$ groups are shown in wireframe representation.

Table S7. Dissociation (dimer → 2 monomer) energies in the gas phase ($\Delta G_{g,298K}$) and in benzene (ΔG_{sol}) in kcal/mol.

Ligand orientation	R	B3LYP				M06			ωB97xD		
		$\Delta H_{g,298K}$	$\Delta G_{g,298K}$	$\Delta\Delta G_{C6H6}$	ΔG_{sol}	$\Delta H_{g,298K}$	$\Delta G_{g,298K}$	ΔG_{sol}	$\Delta H_{g,298K}$	$\Delta G_{g,298K}$	ΔG_{sol}
**Staggered**	**H**	3.7	-15.1	-1.9	-17.0	29.6	10.8	8.9	39.1	20.3	18.4
	**Cl**	5.2	-13.7	-2.0	-15.7	33.8	14.8	12.8	43.1	24.4	22.1
	**Me**	3.7	-15.2	-2.2	-17.4	33.4	14.5	12.4	43.7	24.9	22.7
	**OMe**	3.6	-15.4	-2.4	-17.8	31.4	12.4	10.0	42.8	23.8	21.4
**Eclipsed**	**H**	9.8	-9.5	-5.2	-14.7	33.8	14.5	9.4	44.7	25.4	20.2
	**Cl**	9.6	-9.9	-4.9	-14.8	37.2	17.8	12.9	47.9	28.4	23.5
	**Me**	9.8	-9.6	-5.3	-14.9	36.8	17.5	12.2	49.3	30.0	24.7
	**OMe**	5.2	-11.2	-4.4	-15.5	34.9	15.5	11.1	48.0	28.7	24.3

Table S8. Difference in dissociation energies (dimer → 2 monomer) in the gas phase ($\Delta G_{g,298K}$) and in benzene (ΔG_{sol}) for staggered and eclipsed conformers in kcal/mol.

	B3LYP				M06				ωB97xD			
R	$\Delta H_{g,0K}$	$\Delta H_{g,298K}$	$\Delta G_{g,298K}$	ΔG_{sol}	$\Delta H_{g,0K}$	$\Delta H_{g,298K}$	$\Delta G_{g,298K}$	ΔG_{sol}	$\Delta H_{g,0K}$	$\Delta H_{g,298K}$	$\Delta G_{g,298K}$	ΔG_{sol}
**H**	5.7	6.1	5.6	2.4	3.8	4.2	3.7	0.4	5.2	5.6	5.1	1.8
**Cl**	3.9	4.4	3.9	1.0	3.0	3.5	2.9	0.1	4.4	4.8	4.3	1.4
**Me**	5.7	6.1	5.6	2.4	3.1	3.5	2.9	-0.2	5.2	5.6	5.1	1.9
**OMe**	4.3	4.7	4.3	2.3	3.1	3.5	3.1	1.1	4.8	5.2	4.8	2.8

Mechanistic Calculations

Table S9. Reaction energies for (IPr*R)Cu-H monomer + substrate → complex → transition state → inserted product in the gas phase ($\Delta G_{g,298K}$) and in benzene (ΔG_{sol}) in kcal/mol (substrate = propene or acetaldehyde) starting from the Cu-H monomer. $\Delta\Delta G_{C6H6}$ is the solvation free energy.

			**B3LYP**				**M06**			**ωB97xD**		
**R**	**Substrate**	**Structure**	$\Delta H_{g,298K}$	$\Delta G_{g,298K}$	$\Delta\Delta G_{C6H6}$	ΔG_{sol}	$\Delta H_{g,298K}$	$\Delta G_{g,298K}$	ΔG_{sol}	$\Delta H_{g,298K}$	$\Delta G_{g,298K}$	ΔG_{sol}
**H**	Propene	CuH-substrate complex	4.4	17.8	1.7	19.5	-5.3	8.2	9.9	-7.4	6.1	7.8
		Transition state	15.5	29.3	2.9	32.3	5.7	20.1	23.0	5.0	19.5	22.4
		Product	-17.7	-4.4	2.7	-1.7	-25.7	-12.4	-9.7	-26.7	-13.4	-10.7
	Acetaldehyde	CuH-substrate complex	2.8	16.2	2.2	18.5	0.0	13.4	15.7	-3.4	10.0	12.2
		Transition state	1.9	15.7	4.0	19.7	-1.9	12.0	15.9	-4.3	9.5	13.5
		Product	-27.3	-13.9	2.7	-11.2	-28.3	-14.9	-12.2	-32.4	-19.1	-16.3
**Cl**	Propene	CuH-substrate complex	4.0	17.4	1.7	19.1	-5.6	7.8	9.5	-7.8	5.6	7.3
		Transition state	15.0	29.0	2.8	31.8	5.7	19.7	22.6	5.0	19.0	21.9
		Product	-17.7	-4.4	2.7	-1.7	-25.7	-12.4	-9.7	-26.8	-13.5	-10.8
	Acetaldehyde	CuH-substrate complex	2.4	15.8	2.3	18.1	-0.5	12.8	15.2	-4.1	9.3	11.6
		Transition state	1.7	15.5	4.0	19.5	-2.0	11.8	15.7	-4.6	9.2	13.2
		Product	-27.2	-13.8	2.7	-11.1	-28.2	-14.8	-12.1	-32.3	-19.0	-16.3
**Me**	Propene	CuH-substrate complex	4.5	18.0	1.6	19.5	-5.4	8.0	9.6	-7.5	6.0	7.5
		Transition state	15.4	29.5	2.9	32.3	6.2	20.2	23.0	5.5	19.5	22.3
		Product	-17.7	-4.5	2.8	-1.7	-25.8	-12.5	-9.8	-26.8	-13.5	-10.7
	Acetaldehyde	CuH-substrate complex	2.9	16.3	2.2	18.5	0.0	13.4	15.7	-3.5	9.9	12.2
		Transition state	2.0	15.8	4.0	19.7	-1.8	12.0	16.0	-4.3	9.5	13.5
		Product	-27.3	-14.0	2.8	-11.2	-28.4	-15.1	-12.3	-32.5	-19.2	-16.4

Table S10. Electronic energies in Hartrees for [(IPr*R)CuH]$_2$ and intermediates for insertion at different levels of theory.

R	Structure	B3LYP $\Delta H_{g,298K}$	B3LYP $\Delta G_{g,298K}$	B3LYP $\Delta\Delta G_{C6H6}$	M06 ΔE	ωB97xD ΔE
H	Monomer	-2891.329520	-2891.466935	-2892.413061	-2890.415103	-2891.516852
	Eclipsed dimer	-5782.674616	-5782.918734	-5784.834761	-5780.885334	-5783.106152
	Staggered dimer	-5782.664883	-5782.909803	-5784.829543	-5780.877983	-5783.096578
	Propene complex	-3009.175629	-3009.321702	-3010.344233	-3008.257435	-3009.422074
	TS propene	-3009.158189	-3009.303347	-3010.323866	-3008.238385	-3009.400696
	Product propene	-3009.210813	-3009.357114	-3010.382475	-3008.294755	-3009.457726
	HCOMe complex	-3045.139020	-3045.284872	-3046.286109	-3044.190945	-3045.342894
	TS HCOMe	-3045.140410	-3045.285620	-3046.284114	-3044.193301	-3045.343713
	Product HCOMe	-3045.186929	-3045.332898	-3046.338734	-3044.241523	-3045.394573
Cl	Monomer	-3810.545871	-3810.688338	-3811.612973	-3809.547620	-3810.681444
	Eclipsed dimer	-7621.107092	-7621.360960	-7623.234652	-7619.155762	-7621.440374
	Staggered dimer	-7621.100084	-7621.354821	-7623.231583	-7619.149569	-7621.432019
	Propene complex	-3928.39261	-3928.54377	-3929.54466	-3927.39041	-3928.58731
	TS propene	-3928.37511	-3928.52535	-3929.52427	-3927.37134	-3928.56583
	Product propene	-3928.42718	-3928.57855	-3929.58241	-3927.42721	-3928.6224
	HCOMe complex	-3964.35596	-3964.50693	-3965.4864	-3963.3243	-3964.50849
	TS HCOMe	-3964.35712	-3964.50743	-3965.48423	-3963.32597	-3964.50864
	Product HCOMe	-3964.40313	-3964.55415	-3965.53858	-3963.37389	-3964.55907
Me	Monomer	-2969.927799	-2970.072405	-2971.070350	-2969.008615	-2970.151737
	Eclipsed dimer	-5939.871169	-5940.129516	-5942.149049	-5938.077158	-5940.383343
	Staggered dimer	-5939.861417	-5940.120591	-5942.143634	-5938.070948	-5940.373726
	Propene complex	-3087.77366	-3087.92698	-3089.00133	-3086.85104	-3088.05704
	TS propene	-3087.75626	-3087.90865	-3088.98081	-3086.83153	-3088.03534
	Product propene	-3087.80913	-3087.96269	-3089.03966	-3086.88832	-3088.09256
	HCOMe complex	-3123.73718	-3123.89027	-3124.9432	-3122.78435	-3123.97777
	TS HCOMe	-3123.7386	-3123.89106	-3124.9412	-3122.78661	-3123.97848
	Product HCOMe	-3123.78529	-3123.93848	-3124.99599	-3122.83518	-3124.02963
OMe	Monomer	-3120.373998	-3120.521603	-3121.530900	-3119.406326	-3120.559311
	Eclipsed dimer	-6240.761105	-6241.025410	-6243.069191	-6238.869501	-6241.196439
	Staggered dimer	-6240.753686	-6241.018589	-6243.064244	-6238.863198	-6241.187440

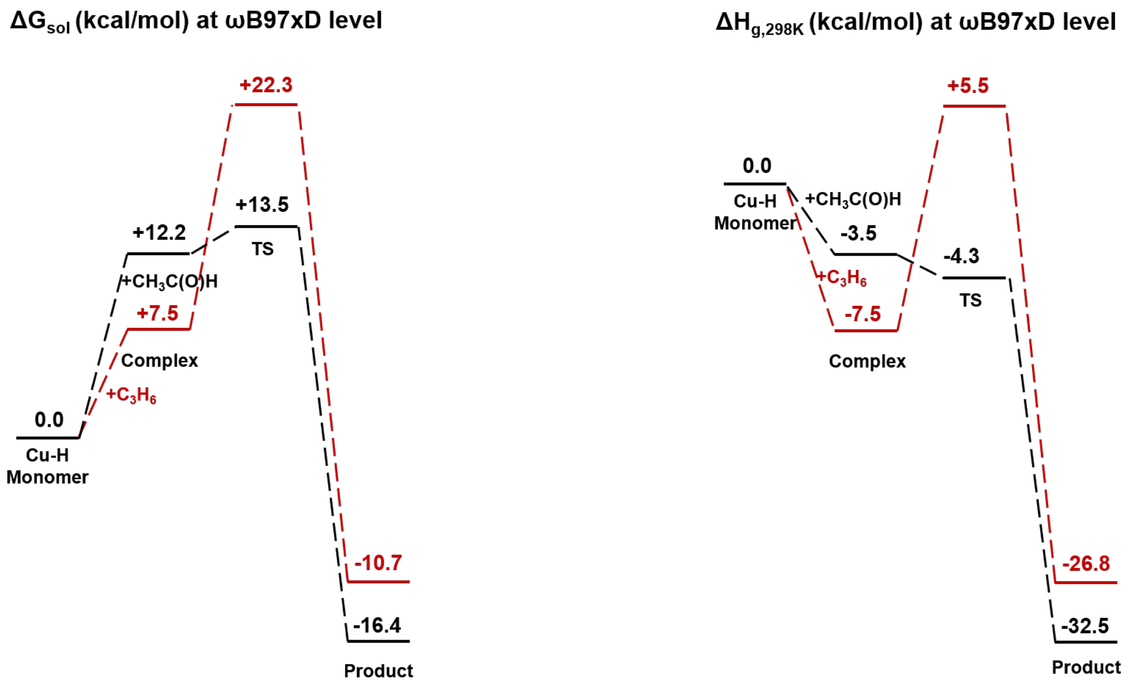

Figure S84. PES diagram for acetaldehyde (black) and propene (red) insertion with [(IPr*Me)CuH]2 calculated at the ωB97xD level. Free energies in benzene are shown on the left, and gas-phase enthalpies are shown on the right.

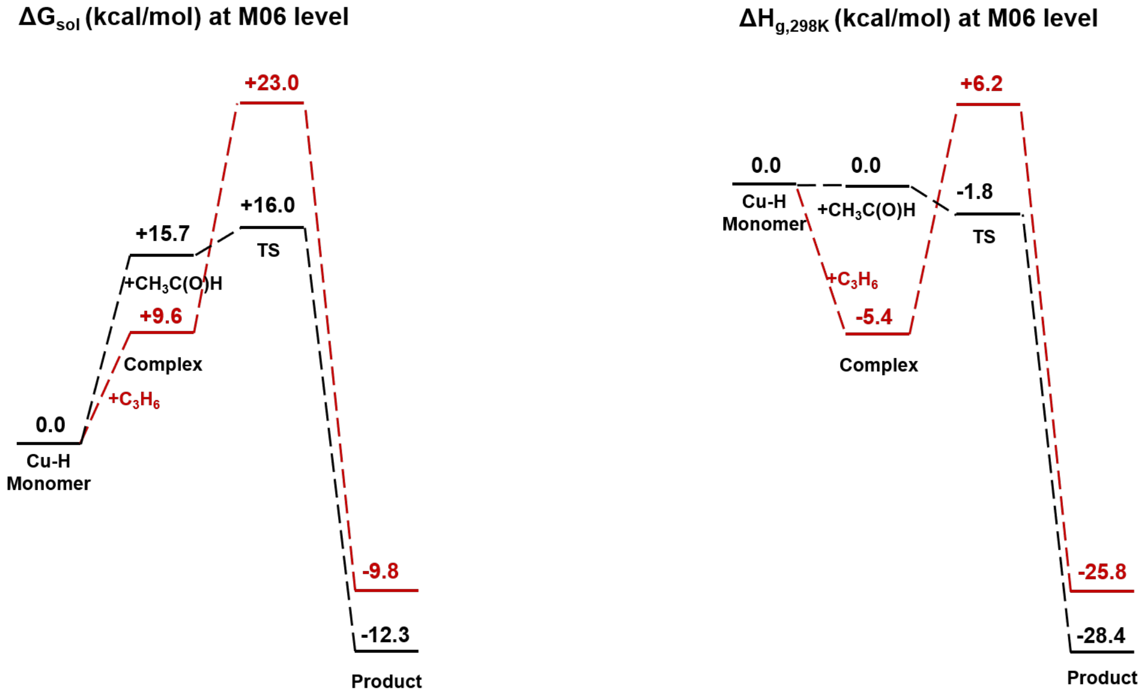

Figure S85. PES diagram for acetaldehyde (black) and propene (red) insertion with [(IPr*Me)CuH]2 calculated at the M06 level. Free energies in benzene are shown on the left, and gas-phase enthalpies are shown on the right.

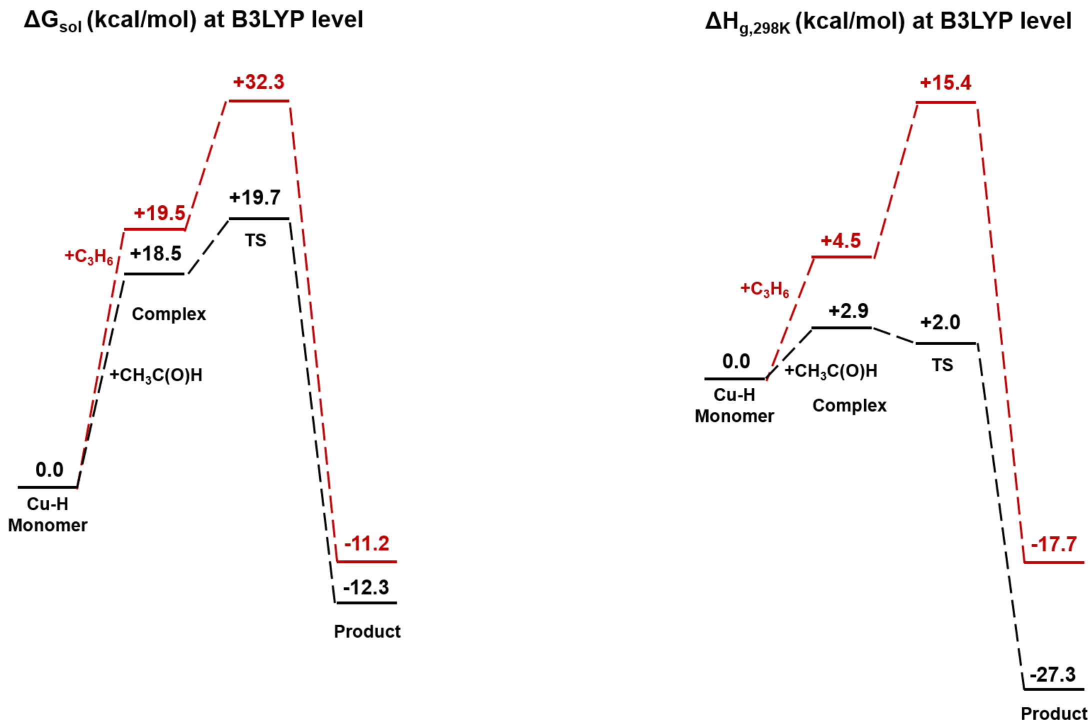

Figure S86. PES diagram for acetaldehyde (black) and propene (red) insertion with [(IPr*Me)CuH]$_2$ calculated at the B3LYP level. Free energies in benzene are shown on the left, and gas-phase enthalpies are shown on the right.

Table S11. Key geometric parameters for DFT-optimized structures of acetaldehyde complex, transition state, and Cu-alkoxide product.

	**Cu-H monomer**	**Acetaldehyde complex**	**Transition state**	**Product**
**Cu-H (Å)**	1.514	1.521	1.561	-
**Cu-C_{NHC} (Å)**	1.926	1.976	1.899	1.866
**NHC-Cu-H (°)**	180	115	143	-
**Cu-O (Å)**	-	1.975	2.059	1.797
**Cu-Cβ (Å)**	-	1.999	2.091	2.876
**O-Cβ (Å)**	-	1.296	1.309	1.407
**Cβ-H (Å)**	-	2.599	1.775	1.106

Table S12. Key geometric parameters for DFT-optimized structures of propene complex, transition state, and Cu-alkyl product.

	**Cu-H monomer**	**Propene complex**	**Transition state**	**Product**
**Cu-H (Å)**	1.514	1.555	1.597	-
**Cu-C_{NHC} (Å)**	1.926	1.960	1.888	1.922
**NHC-Cu-H (°)**	180	108	126	-
**Cu-Cα (Å)**	-	2.074	2.049	1.942
**Cu-Cβ (Å)**	-	2.040	2.065	2.978
**Cα-Cβ (Å)**	-	1.397	1.449	1.545
**Cβ-H (Å)**	-	2.732	1.613	1.099

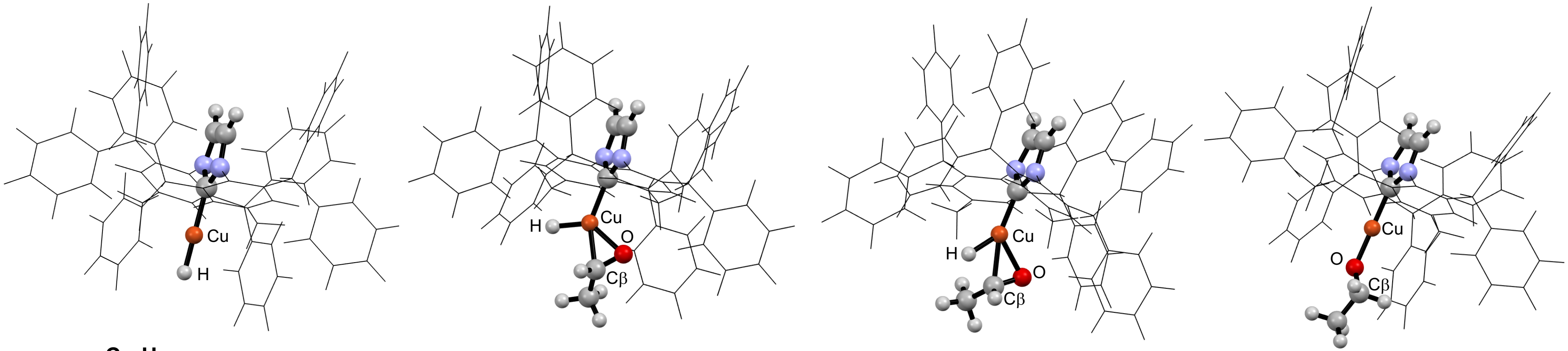

Cu-H monomer **Acetaldehyde complex** **Transition state** **Product**

Figure S87. DFT-optimized structures of CuH monomer, CuH-acetaldehyde complex, transition state for hydride insertion, and Cu-alkoxide product. The ligand aryl groups are shown in wireframe representation.

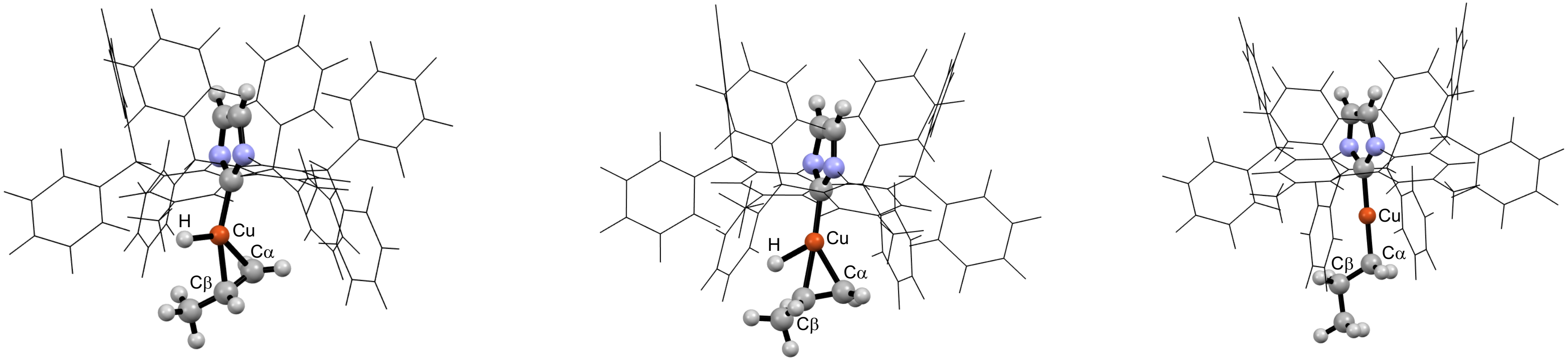

Propene complex **Transition state** **Product**

Figure S88. DFT-optimized structures of CuH-propene complex, transition state for hydride insertion, and Cu-alkyl product. The ligand aryl groups are shown in wireframe representation.

NBO Analysis

NBO analysis was performed on the (IPr*R)Cu-H monomer and dimer, with R = H, Cl, Me, and OMe, as well as the transition states for insertion of acetaldehyde and propene, with R = H, Cl, and Me. Key findings for the IPr*H system are presented below. There is no significant difference in the results for the different R-groups (see Table S13 and the next section for results with other ligands).

Table S13. NBO charges for [(IPr*R)CuH] monomer, dimer, and transition state for insertions with propene and acetaldehyde.

	IPr*H		IPr*Cl		IPr*Me		IPr*OMe	
	**Cu**	**H**	**Cu**	**H**	**Cu**	**H**	**Cu**	**H**
**Monomer**	0.327	-0.456	0.326	-0.453	0.327	-0.457	0.326	-0.458
**Dimer**	0.545	-0.574	0.543	-0.573	0.543	-0.575, -0.571	0.542	-0.570, -0.571
**Propene complex**	0.752	-0.522	0.751	-0.521	0.752	-0.522		
**TS propene**	0.737	-0.256	0.736	-0.255	0.736	-0.256		
**HCOMe complex**	0.774	-0.447	0.770	-0.447	0.774	-0.447		
**TS HCOMe**	0.696	-0.308	0.694	-0.307	0.696	-0.308		

NBO charges

- **[(IPr*H)CuH]$_2$ dimer and monomer**: There is more polarization of charge in the Cu-H dimer compared to the monomer. Additionally, in the dimer the positive charge on the Cu and negative charge on the H are approximately equal in magnitude, whereas in the monomer there is more negative charge on the H than positive charge on Cu, indicating that there is some positive charge on the NHC ligand.
- **Transition states**: Compared to the Cu-H monomer, there is a large increase in positive charge on the Cu in the transition state for hydride transfer. There is also a decrease in negative charge on the hydride. The magnitude of these changes is larger when propene is the substrate in comparison to acetaldehyde.

NBO analysis of Cu bonding

- **[(IPr*H)CuH]$_2$ dimer**: The NBO analysis assigns 9.89 e to the Cu 3d orbitals for each Cu and 1.57 e to the H 1s orbital for each H. There is a 'non-Lewis' orbital with 0.51 e in the 4s orbital on each Cu.
- **[(IPr*H)CuH] monomer:** The Cu-H monomer has 9.93 e in the Cu 3d orbitals There is a Cu-H bond with 1.98 e in it with 0.59 e on the Cu (mostly in the 4s orbital) and 1.39 e on the H in the 1s orbital.
- **[(IPr*H)CuH]-HCOMe complex**: The 3d population is reduced to 9.70 e. There is a Cu-H bond with 1.72 e which has 1.41 e on the H 1s and 0.31 mostly on the Cu 4s. There is a corresponding 'non-Lewis' bond with 0.18 e mostly on the Cu 4s.
- **[(IPr*H)CuH] acetaldehyde transition state:** The Cu 3d population increases back to 9.82 e. There is now a C-H bond involving the hydride which has 1.76 e (1.16 e on the H and 0.60 e on the C). There is 0.42 e in the Cu 4s in a 'non-Lewis' valence nonbonding orbital and there is 0.43 e in a C-H σ* with opposite polarization to the bonding orbital. Thus, the NBO analysis suggests that the hydride has mostly transferred to the C in the transition state. Note also that there is a loss of Cu 3d character upon formation of the complex that is partially regained in the transition state.
- **[(IPr*H)CuH]-propene complex**: The 3d population is reduced to 9.74 e. There is a very polar Cu-H bond with 1.75 e total electrons in it (0.26 e on the Cu and 1.49 e on the H). There is a Cu-H σ* bond with 0.2 e (0.17 in the Cu 4s orbital and 0.03 e in the H 1s orbital).
- **[(IPr*H)CuH]-propene transition state**: In the propene transition state, there are 9.81 e in the Cu 3d orbitals and 1.25 e in the H 1s orbital. There are 0.37 e in a 'non-Lewis' valence nonbonding orbital in the Cu 1s in the transition state. Unlike the HCOMe transition state, in the propene transition state the H is still mostly interacting with the Cu center and has not yet transferred to the substrate. Similar to the acetaldehyde complex, there is a loss of Cu 3d character upon formation of the complex that is partially regained in the transition state.

NBO analysis of the Cu bonding

Ligand = IPr*H

[(IPr*H)CuH]$_2$ dimer

1. (1.99878) LP (1)Cu 1 s(0.00%)p 0.00(0.00%)d 1.00(100.00%)
2. (1.99482) LP (2)Cu 1 s(1.25%)p 0.00(0.00%)d78.80(98.74%)
3. (1.98768) LP (3)Cu 1 s(0.00%)p 0.00(0.01%)d 1.00(99.99%)
4. (1.98472) LP (4)Cu 1 s(1.93%)p 0.00(0.01%)d50.68(98.06%)
5. (1.91265) LP (5)Cu 1 s(0.02%)p 4.73(0.07%)d99.99(99.91%)
6. (1.56914) LP (1) H 2 s(99.93%)p 0.00(0.07%)
7. (1.56914) LP (1) H 3 s(99.93%)p 0.00(0.07%)
8. (1.99878) LP (1)Cu 4 s(0.00%)p 0.00(0.00%)d 1.00(100.00%)
9. (1.99482) LP (2)Cu 4 s(1.25%)p 0.00(0.00%)d78.78(98.74%)
10. (1.98769) LP (3)Cu 4 s(0.00%)p 0.00(0.01%)d 1.00(99.99%)
11. (1.98472) LP (4)Cu 4 s(1.93%)p 0.00(0.01%)d50.69(98.06%)
12. (1.91263) LP (5)Cu 4 s(0.02%)p 4.72(0.07%)d99.99(99.91%)

---------------- non-Lewis --

13. (0.50828) LV (1)Cu 1 s(95.90%)p 0.00(0.29%)d 0.04(3.80%)
14. (0.50828) LV (1)Cu 4 s(95.90%)p 0.00(0.29%)d 0.04(3.80%)

[(IPr*H)CuH] monomer

1. (1.99867) LP (1)Cu 1 s(0.01%)p 0.01(0.00%)d99.99(99.99%)
2. (1.99832) LP (2)Cu 1 s(0.13%)p 0.00(0.00%)d99.99(99.87%)
3. (1.98659) LP (3)Cu 1 s(0.00%)p 1.00(0.04%)d99.99(99.95%)
4. (1.98573) LP (4)Cu 1 s(8.46%)p 0.00(0.01%)d10.82(91.54%)
5. (1.95324) LP (5)Cu 1 s(0.00%)p 1.00(0.03%)d99.99(99.97%)
6. (1.97964) BD (1)Cu 1- H121
   (29.57%) 0.5437*Cu 1 s(90.07%)p 0.01(1.03%)d 0.10(8.89%)
   (70.43%) 0.8392* H121 s(99.91%)p 0.00(0.09%)

---------------- non-Lewis ---

7. (0.20201) BD*(1)Cu 1- H121
   (70.43%) 0.8392*Cu 1 s(90.07%)p 0.01(1.03%)d 0.10(8.89%)
   (29.57%) -0.5437* H121 s(99.91%)p 0.00(0.09%)

[(IPr*H)CuH] propene complex

1. (1.99713) LP (1)Cu 8 s(0.00%)p 0.00(0.01%)d 1.00(99.99%)
2. (1.99497) LP (2)Cu 8 s(1.30%)p 0.00(0.00%)d76.17(98.70%)
3. (1.98869) LP (3)Cu 8 s(0.00%)p 1.00(0.01%)d99.99(99.98%)
4. (1.96521) LP (4)Cu 8 s(0.01%)p 0.37(0.00%)d99.99(99.99%)
5. (1.79213) LP (5)Cu 8 s(0.34%)p 0.11(0.04%)d99.99(99.62%)
6. (1.75440) BD (1)Cu 8- H 11
   (15.09%) 0.3885*Cu 8 s(93.15%)p 0.04(3.53%)d 0.04(3.30%)
   (84.91%) 0.9214* H 11 s(99.95%)p 0.00(0.05%)

---------------- non-Lewis ---

7. (0.19796) BD*(1)Cu 8- H 11
   (84.91%) 0.9214*Cu 8 s(93.15%)p 0.04(3.53%)d 0.04(3.30%)
   (15.09%) -0.3885* H 11 s(99.95%)p 0.00(0.05%)

[(IPr*H)CuH] propene insertion TS

1. (1.99781) LP (1)Cu 8 s(0.00%)p 0.00(0.00%)d 1.00(100.00%)
2. (1.99514) LP (2)Cu 8 s(1.29%)p 0.00(0.00%)d76.79(98.71%)
3. (1.98519) LP (3)Cu 8 s(0.01%)p 0.00(0.01%)d 1.00(99.99%)
4. (1.92710) LP (4)Cu 8 s(1.31%)p 0.02(0.03%)d75.17(98.66%)
5. (1.90181) LP (5)Cu 8 s(1.13%)p 0.03(0.04%)d87.09(98.83%)
6. (1.24995) LP (1) H 11 s(99.91%)p 0.00(0.09%)

---------------- non-Lewis ---

7. (0.37085) LV (1)Cu 8 s(95.80%)p 0.00(0.23%)d 0.04(3.96%)

[(IPr*H)CuH] HCOMe complex

1. (1.99816) LP (1)Cu 8 s(0.00%)p 0.00(0.00%)d 1.00(100.00%)
2. (1.99490) LP (2)Cu 8 s(1.39%)p 0.00(0.00%)d70.69(98.60%)
3. (1.98913) LP (3)Cu 8 s(0.00%)p 1.00(0.02%)d99.99(99.98%)

4. (1.97045) LP (4)Cu 8 s(0.01%)p 1.07(0.01%)d99.99(99.98%)
5. (1.74650) LP (5)Cu 8 s(0.12%)p 0.33(0.04%)d99.99(99.84%)
6. (1.71879) BD (1)Cu 8- H 12
(17.79%) 0.4217*Cu 8 s(94.04%)p 0.03(2.96%)d 0.03(2.99%)
(82.21%) 0.9067* H 12 s(99.93%)p 0.00(0.07%)
---------------- non-Lewis --
7. (0.18121) BD*(1)Cu 8- H 12
(82.21%) 0.9067*Cu 8 s(94.04%)p 0.03(2.96%)d 0.03(2.99%)
(17.79%) -0.4217* H 12 s(99.93%)p 0.00(0.07%)

[(IPr*H)CuH] HCOMe insertion TS
1. (1.99845) LP (1)Cu 8 s(0.00%)p 0.00(0.00%)d 1.00(100.00%)
2. (1.99515) LP (2)Cu 8 s(1.55%)p 0.00(0.00%)d63.40(98.44%)
3. (1.98787) LP (3)Cu 8 s(0.01%)p 1.00(0.01%)d99.99(99.98%)
4. (1.93620) LP (4)Cu 8 s(0.75%)p 0.13(0.10%)d99.99(99.16%)
5. (1.90103) LP (5)Cu 8 s(1.52%)p 0.01(0.01%)d64.86(98.47%)
6. (1.75917) BD (1) C 10- H 12
(34.13%) 0.5842* C 10 s(6.81%)p13.66(93.06%)d 0.02(0.12%)
(65.87%) 0.8116* H 12 s(99.89%)p 0.00(0.11%)
---------------- non-Lewis --
7. (0.41669) LV (1)Cu 8 s(95.14%)p 0.01(0.58%)d 0.04(4.28%)
8. (0.42548) BD*(1) C 10- H 12
(65.87%) 0.8116* C 10 s(6.81%)p13.66(93.06%)d 0.02(0.12%)
(34.13%) -0.5842* H 12 s(99.89%)p 0.00(0.11%)

*Ligand = IPr*Me*

[(IPr*Me)CuH]$_2$ dimer
1. (1.99883) LP (1)Cu 1 s(0.00%)p 0.00(0.00%)d 1.00(100.00%)
2. (1.99489) LP (2)Cu 1 s(1.25%)p 0.00(0.00%)d78.72(98.74%)
3. (1.98790) LP (3)Cu 1 s(0.01%)p 0.00(0.01%)d 1.00(99.99%)
4. (1.98465) LP (4)Cu 1 s(1.89%)p 0.00(0.01%)d51.98(98.11%)
5. (1.91192) LP (5)Cu 1 s(0.01%)p 7.46(0.08%)d99.99(99.91%)
6. (1.57038) LP (1) H 2 s(99.93%)p 0.00(0.07%)
7. (1.56690) LP (1) H 3 s(99.94%)p 0.00(0.06%)
8. (1.99879) LP (1)Cu 4 s(0.00%)p 0.00(0.00%)d 1.00(100.00%)
9. (1.99492) LP (2)Cu 4 s(1.25%)p 0.00(0.00%)d78.98(98.75%)
10. (1.98758) LP (3)Cu 4 s(0.00%)p 0.00(0.01%)d 1.00(99.99%)
11. (1.98484) LP (4)Cu 4 s(1.94%)p 0.00(0.01%)d50.64(98.06%)
12. (1.91321) LP (5)Cu 4 s(0.01%)p 1.00(0.08%)d99.99(99.92%)
---------------- non-Lewis --
13. (0.50821) LV (1)Cu 1 s(95.92%)p 0.00(0.30%)d 0.04(3.77%)
14. (0.50838) LV (1)Cu 4 s(95.89%)p 0.00(0.29%)d 0.04(3.81%)

[(IPr*Me)CuH] monomer
1. (1.99867) LP (1)Cu 1 s(0.01%)p 0.00(0.00%)d 1.00(99.99%)
2. (1.99833) LP (2)Cu 1 s(0.14%)p 0.00(0.00%)d99.99(99.86%)
3. (1.98676) LP (3)Cu 1 s(0.00%)p 1.00(0.04%)d99.99(99.96%)
4. (1.98586) LP (4)Cu 1 s(8.47%)p 0.00(0.01%)d10.81(91.53%)
5. (1.95393) LP (5)Cu 1 s(0.00%)p 1.00(0.03%)d99.99(99.97%)
6. (1.97966) BD (1)Cu 1- H121
(29.50%) 0.5431*Cu 1 s(90.05%)p 0.01(1.03%)d 0.10(8.91%)
(70.50%) 0.8396* H121 s(99.91%)p 0.00(0.09%)
---------------- non-Lewis --
7. (0.20282) BD*(1)Cu 1- H121
(70.50%) 0.8396*Cu 1 s(90.05%)p 0.01(1.03%)d 0.10(8.91%)
(29.50%) -0.5431* H121 s(99.91%)p 0.00(0.09%)

[(IPr*Me)CuH] propene complex
1. (1.99709) LP (1)Cu 8 s(0.00%)p 0.00(0.01%)d 1.00(99.99%)
2. (1.99500) LP (2)Cu 8 s(1.30%)p 0.00(0.00%)d76.12(98.70%)
3. (1.98870) LP (3)Cu 8 s(0.00%)p 1.00(0.01%)d99.99(99.98%)
4. (1.96544) LP (4)Cu 8 s(0.01%)p 0.00(0.00%)d 1.00(99.99%)

5. (1.79133) LP (5)Cu 8 s(0.34%)p 0.11(0.04%)d99.99(99.62%)
6. (1.75612) BD (1)Cu 8- H 11
(15.17%) 0.3895*Cu 8 s(93.19%)p 0.04(3.53%)d 0.03(3.26%)
(84.83%) 0.9210* H 11 s(99.95%)p 0.00(0.05%)
---------------- non-Lewis ---
7. (0.19752) BD*(1)Cu 8- H 11
(84.83%) 0.9210*Cu 8 s(93.19%)p 0.04(3.53%)d 0.03(3.26%)
(15.17%) -0.3895* H 11 s(99.95%)p 0.00(0.05%)

[(IPr*Me)CuH] propene insertion TS
1. (1.99781) LP (1)Cu 8 s(0.00%)p 0.00(0.00%)d 1.00(100.00%)
2. (1.99518) LP (2)Cu 8 s(1.29%)p 0.00(0.00%)d76.52(98.71%)
3. (1.98525) LP (3)Cu 8 s(0.01%)p 0.00(0.01%)d 1.00(99.99%)
4. (1.92758) LP (4)Cu 8 s(1.23%)p 0.02(0.03%)d80.45(98.74%)
5. (1.90190) LP (5)Cu 8 s(1.23%)p 0.03(0.04%)d80.46(98.74%)
6. (1.25025) LP (1) H 11 s(99.91%)p 0.00(0.09%)
---------------- non-Lewis ---
7. (0.37115) LV (1)Cu 8 s(95.80%)p 0.00(0.22%)d 0.04(3.97%)

[(IPr*Me)CuH] HCOMe complex
1. (1.99816) LP (1)Cu 8 s(0.00%)p 0.00(0.00%)d 1.00(100.00%)
2. (1.99492) LP (2)Cu 8 s(1.39%)p 0.00(0.00%)d70.88(98.61%)
3. (1.98914) LP (3)Cu 8 s(0.00%)p 1.00(0.02%)d99.99(99.98%)
4. (1.97099) LP (4)Cu 8 s(0.01%)p 0.99(0.01%)d99.99(99.98%)
5. (1.74514) LP (5)Cu 8 s(0.12%)p 0.33(0.04%)d99.99(99.83%)
6. (1.71786) BD (1)Cu 8- H 12
(17.77%) 0.4216*Cu 8 s(94.05%)p 0.03(2.96%)d 0.03(2.97%)
(82.23%) 0.9068* H 12 s(99.93%)p 0.00(0.07%)
---------------- non-Lewis ---
7. (0.18167) BD*(1)Cu 8- H 12
(82.23%) 0.9068*Cu 8 s(94.05%)p 0.03(2.96%)d 0.03(2.97%)
(17.77%) -0.4216* H 12 s(99.93%)p 0.00(0.07%)

[(IPr*Me)CuH] HCOMe insertion TS
1. (1.99846) LP (1)Cu 8 s(0.00%)p 0.00(0.00%)d 1.00(100.00%)
2. (1.99520) LP (2)Cu 8 s(1.55%)p 0.00(0.00%)d63.41(98.45%)
3. (1.98797) LP (3)Cu 8 s(0.01%)p 1.00(0.01%)d99.99(99.98%)
4. (1.93713) LP (4)Cu 8 s(0.71%)p 0.13(0.09%)d99.99(99.19%)
5. (1.89995) LP (5)Cu 8 s(1.56%)p 0.01(0.01%)d63.30(98.43%)
6. (1.76003) BD (1) C 10- H 12
(34.18%) 0.5846* C 10 s(6.84%)p13.59(93.03%)d 0.02(0.12%)
(65.82%) 0.8113* H 12 s(99.89%)p 0.00(0.11%)
---------------- non-Lewis ---
7. (0.41686) LV (1)Cu 8 s(95.15%)p 0.01(0.57%)d 0.04(4.27%)
8. (0.42692) BD*(1) C 10- H 12
(65.82%) 0.8113* C 10 s(6.84%)p13.59(93.03%)d 0.02(0.12%)
(34.18%) -0.5846* H 12 s(99.89%)p 0.00(0.11%)

*Ligand = IPr*Cl*

[(IPr*Cl)CuH]$_2$ dimer
1. (1.99879) LP (1)Cu 1 s(0.00%)p 0.00(0.00%)d 1.00(100.00%)
2. (1.99481) LP (2)Cu 1 s(1.26%)p 0.00(0.00%)d78.66(98.74%)
3. (1.98768) LP (3)Cu 1 s(0.00%)p 0.00(0.01%)d 1.00(99.99%)
4. (1.98480) LP (4)Cu 1 s(1.93%)p 0.00(0.01%)d50.68(98.06%)
5. (1.91208) LP (5)Cu 1 s(0.01%)p 4.86(0.07%)d99.99(99.91%)
6. (1.56800) LP (1) H 2 s(99.93%)p 0.00(0.07%)
7. (1.56802) LP (1) H 3 s(99.93%)p 0.00(0.07%)
8. (1.99879) LP (1)Cu 4 s(0.00%)p 0.00(0.00%)d 1.00(100.00%)
9. (1.99481) LP (2)Cu 4 s(1.26%)p 0.00(0.00%)d78.64(98.74%)
10. (1.98768) LP (3)Cu 4 s(0.00%)p 0.00(0.01%)d 1.00(99.99%)
11. (1.98480) LP (4)Cu 4 s(1.93%)p 0.00(0.01%)d50.69(98.06%)

12. (1.91209) LP (5)Cu 4 s(0.01%)p 4.88(0.07%)d99.99(99.91%)
---------------- non-Lewis --
13. (0.50885) LV (1)Cu 1 s(95.89%)p 0.00(0.29%)d 0.04(3.81%)
14. (0.50885) LV (1)Cu 4 s(95.89%)p 0.00(0.29%)d 0.04(3.81%)

[(IPr*Cl)CuH] monomer
1. (1.99863) LP (1)Cu 1 s(0.01%)p 0.01(0.00%)d99.99(99.99%)
2. (1.99832) LP (2)Cu 1 s(0.13%)p 0.00(0.00%)d99.99(99.87%)
3. (1.98639) LP (3)Cu 1 s(0.00%)p 1.00(0.05%)d99.99(99.95%)
4. (1.98566) LP (4)Cu 1 s(8.46%)p 0.00(0.01%)d10.82(91.53%)
5. (1.95236) LP (5)Cu 1 s(0.00%)p 1.00(0.03%)d99.99(99.97%)
6. (1.97963) BD (1)Cu 1- H121
(29.73%) 0.5452*Cu 1 s(90.05%)p 0.01(1.03%)d 0.10(8.91%)
(70.27%) 0.8383* H121 s(99.91%)p 0.00(0.09%)
---------------- non-Lewis --
7. (0.20143) BD*(1)Cu 1- H121
(70.27%) 0.8383*Cu 1 s(90.05%)p 0.01(1.03%)d 0.10(8.91%)
(29.73%) -0.5452* H121 s(99.91%)p 0.00(0.09%)

[(IPr*Cl)CuH] propene complex
1. (1.99714) LP (1)Cu 8 s(0.00%)p 0.00(0.01%)d 1.00(99.99%)
2. (1.99496) LP (2)Cu 8 s(1.30%)p 0.00(0.00%)d75.90(98.70%)
3. (1.98860) LP (3)Cu 8 s(0.00%)p 1.00(0.01%)d99.99(99.98%)
4. (1.96456) LP (4)Cu 8 s(0.01%)p 0.30(0.00%)d99.99(99.98%)
5. (1.79351) LP (5)Cu 8 s(0.35%)p 0.11(0.04%)d99.99(99.61%)
6. (1.75296) BD (1)Cu 8- H 11
(15.07%) 0.3882*Cu 8 s(93.11%)p 0.04(3.55%)d 0.04(3.33%)
(84.93%) 0.9216* H 11 s(99.95%)p 0.00(0.05%)
---------------- non-Lewis ---
7. (0.19855) BD*(1)Cu 8- H 11
(84.93%) 0.9216*Cu 8 s(93.11%)p 0.04(3.55%)d 0.04(3.33%)
(15.07%) -0.3882* H 11 s(99.95%)p 0.00(0.05%)

[(IPr*Cl)CuH] propene insertion TS
1. (1.99780) LP (1)Cu 8 s(0.00%)p 0.00(0.00%)d 1.00(100.00%)
2. (1.99512) LP (2)Cu 8 s(1.29%)p 0.00(0.00%)d76.58(98.71%)
3. (1.98511) LP (3)Cu 8 s(0.01%)p 0.00(0.01%)d 1.00(99.98%)
4. (1.92654) LP (4)Cu 8 s(1.46%)p 0.02(0.03%)d67.69(98.52%)
5. (1.90179) LP (5)Cu 8 s(0.98%)p 0.04(0.04%)d99.99(98.97%)
6. (1.24918) LP (1) H 11 s(99.91%)p 0.00(0.09%)
---------------- non-Lewis --
7. (0.37152) LV (1)Cu 8 s(95.80%)p 0.00(0.23%)d 0.04(3.96%)

[(IPr*Cl)CuH] HCOMe complex
1. (1.99814) LP (1)Cu 8 s(0.00%)p 0.00(0.00%)d 1.00(100.00%)
2. (1.99485) LP (2)Cu 8 s(1.40%)p 0.00(0.00%)d70.59(98.60%)
3. (1.98884) LP (3)Cu 8 s(0.00%)p 1.00(0.02%)d99.99(99.98%)
4. (1.97047) LP (4)Cu 8 s(0.01%)p 0.79(0.01%)d99.99(99.97%)
5. (1.74815) LP (5)Cu 8 s(0.11%)p 0.36(0.04%)d99.99(99.85%)
6. (1.72246) BD (1)Cu 8- H 12
(17.97%) 0.4239*Cu 8 s(94.09%)p 0.03(2.93%)d 0.03(2.96%)
(82.03%) 0.9057* H 12 s(99.93%)p 0.00(0.07%)
---------------- non-Lewis --------
7. (0.18016) BD*(1)Cu 8- H 12
(82.03%) 0.9057*Cu 8 s(94.09%)p 0.03(2.93%)d 0.03(2.96%)
(17.97%) -0.4239* H 12 s(99.93%)p 0.00(0.07%)

[(IPr*Cl)CuH] HCOMe insertion TS
1. (1.99843) LP (1)Cu 8 s(0.00%)p 0.00(0.00%)d 1.00(100.00%)
2. (1.99510) LP (2)Cu 8 s(1.57%)p 0.00(0.00%)d62.88(98.43%)
3. (1.98772) LP (3)Cu 8 s(0.01%)p 1.00(0.01%)d99.99(99.98%)
4. (1.93465) LP (4)Cu 8 s(0.81%)p 0.12(0.10%)d99.99(99.09%)

5. (1.90290) LP (5)Cu 8 s(1.45%)p 0.01(0.01%)d67.74(98.53%)
6. (1.75755) BD (1) C 10- H 12
(34.02%) 0.5832* C 10 s(6.76%)p13.78(93.12%)d 0.02(0.12%)
(65.98%) 0.8123* H 12 s(99.89%)p 0.00(0.11%)
---------------- non-Lewis --
7. (0.41728) LV (1)Cu 8 s(95.11%)p 0.01(0.59%)d 0.05(4.29%)
8. (0.42311) BD*(1) C 10- H 12
(65.98%) 0.8123* C 10 s(6.76%)p13.78(93.12%)d 0.02(0.12%)
(34.02%) -0.5832* H 12 s(99.89%)p 0.00(0.11%)

Ligand = IPr*OMe

[(IPr*OMe)CuH]$_2$ dimer
1. (1.99891) LP (1)Cu 1 s(0.00%)p 0.00(0.00%)d 1.00(100.00%)
2. (1.99493) LP (2)Cu 1 s(1.25%)p 0.00(0.00%)d79.07(98.75%)
3. (1.98778) LP (3)Cu 1 s(0.00%)p 0.00(0.01%)d 1.00(99.99%)
4. (1.98494) LP (4)Cu 1 s(1.88%)p 0.00(0.01%)d52.09(98.11%)
5. (1.90985) LP (5)Cu 1 s(0.00%)p 1.00(0.09%)d99.99(99.91%)
6. (1.56462) LP (1) H 2 s(99.93%)p 0.00(0.07%)
7. (1.56653) LP (1) H 3 s(99.93%)p 0.00(0.07%)
8. (1.99889) LP (1)Cu 4 s(0.00%)p 0.00(0.00%)d 1.00(100.00%)
9. (1.99500) LP (2)Cu 4 s(1.26%)p 0.00(0.00%)d78.38(98.74%)
10. (1.98787) LP (3)Cu 4 s(0.00%)p 0.00(0.01%)d 1.00(99.99%)
11. (1.98488) LP (4)Cu 4 s(1.87%)p 0.00(0.01%)d52.36(98.12%)
12. (1.91040) LP (5)Cu 4 s(0.00%)p 1.00(0.09%)d99.99(99.91%)
---------------- non-Lewis --
13. (0.50864) LV (1)Cu 1 s(95.91%)p 0.00(0.30%)d 0.04(3.78%)
14. (0.50830) LV (1)Cu 4 s(95.91%)p 0.00(0.30%)d 0.04(3.78%)

[(IPr*OMe)CuH] monomer
1. (1.99870) LP (1)Cu 1 s(0.00%)p 0.00(0.00%)d 1.00(100.00%)
2. (1.99834) LP (2)Cu 1 s(0.16%)p 0.00(0.00%)d99.99(99.84%)
3. (1.98686) LP (3)Cu 1 s(0.00%)p 1.00(0.04%)d99.99(99.95%)
4. (1.98605) LP (4)Cu 1 s(8.47%)p 0.00(0.01%)d10.81(91.52%)
5. (1.95413) LP (5)Cu 1 s(0.00%)p 1.00(0.03%)d99.99(99.97%)
6. (1.97961) BD (1)Cu 1- H131
(29.48%) 0.5429*Cu 1 s(90.04%)p 0.01(1.03%)d 0.10(8.93%)
(70.52%) 0.8398* H131 s(99.91%)p 0.00(0.09%)
---------------- non-Lewis --
7. (0.20345) BD*(1)Cu 1- H131
(70.52%) 0.8398*Cu 1 s(90.04%)p 0.01(1.03%)d 0.10(8.93%)
(29.48%) -0.5429* H131 s(99.91%)p 0.00(0.09%)

TD-DFT Calculations

Table S14. Assignment of TD-DFT predicted UV-Visible transitions in benzene for $[(IPr^*R)CuH]_2$ (eclipsed conformation) from TD-DFT.

	R = H		R = Cl		R = Me
λ (nm)	Transition type	λ (nm)	Transition type	λ (nm)	Transition type
464	HOMO → LUMO	472	HOMO → LUMO	455	HOMO → LUMO
450	HOMO → LUMO + 2	463	HOMO → LUMO + 2	441	HOMO → LUMO + 2
382	HOMO → LUMO (high)	388	HOMO → LUMO (high)	383	HOMO → LUMO (high)
366	HOMO → LUMO (high)	381	HOMO → LUMO (high)	372	HOMO → LUMO (high)
		367	HOMO → LUMO (high)	370	HOMO → LUMO (high)

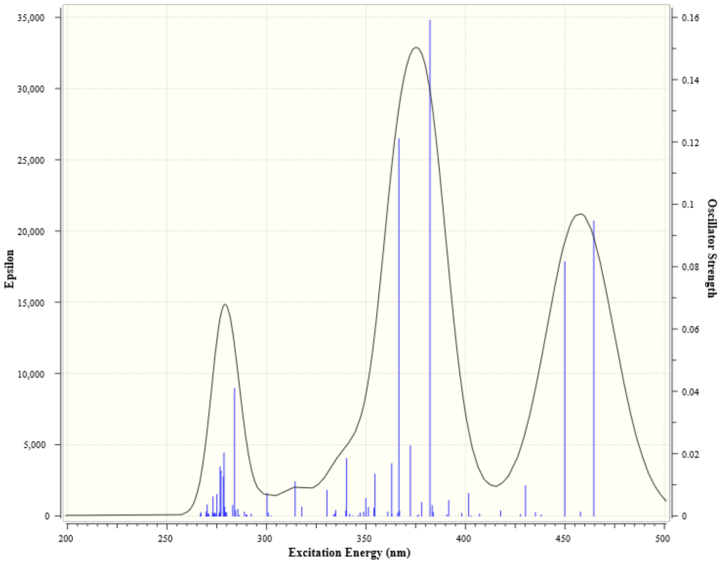

Figure S89. TD-DFT predicted UV-Visible spectrum for eclipsed $[(IPr^*H)CuH]_2$ in benzene.

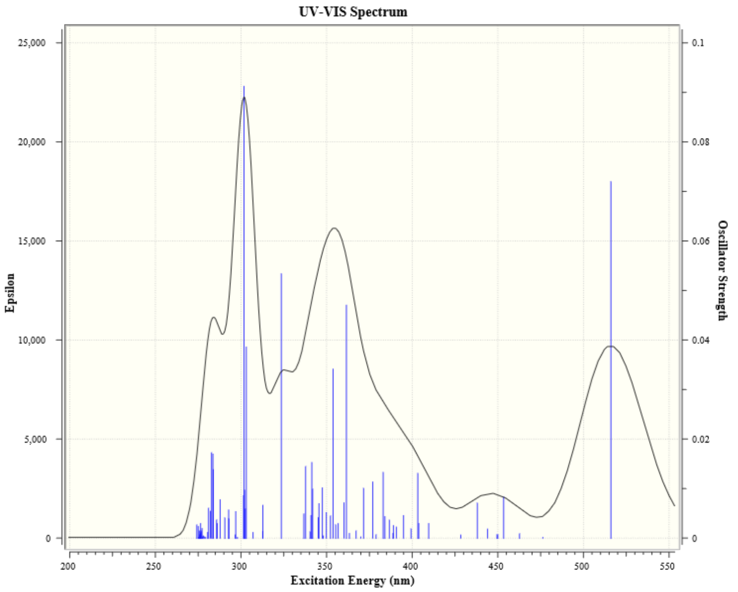

Figure S90. TD-DFT predicted UV-Visible spectrum for staggered $[(IPr^*H)CuH]_2$ in benzene.

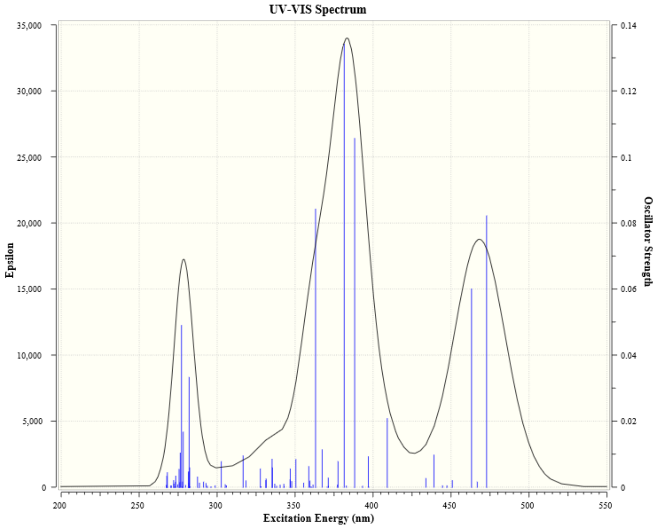

Figure S91. TD-DFT UV-Visible spectrum for eclipsed [(IPr*Cl)CuH]$_2$ in benzene.

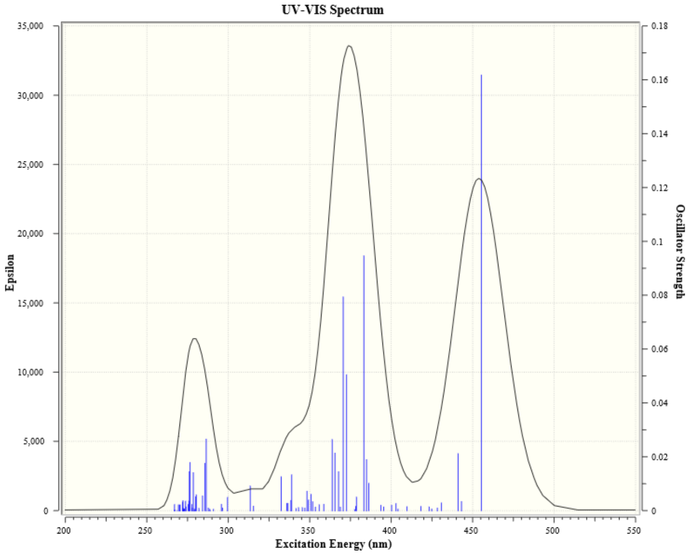

Figure S92. TD-DFT UV-Visible spectrum for eclipsed [(IPr*Me)CuH]$_2$ in benzene.

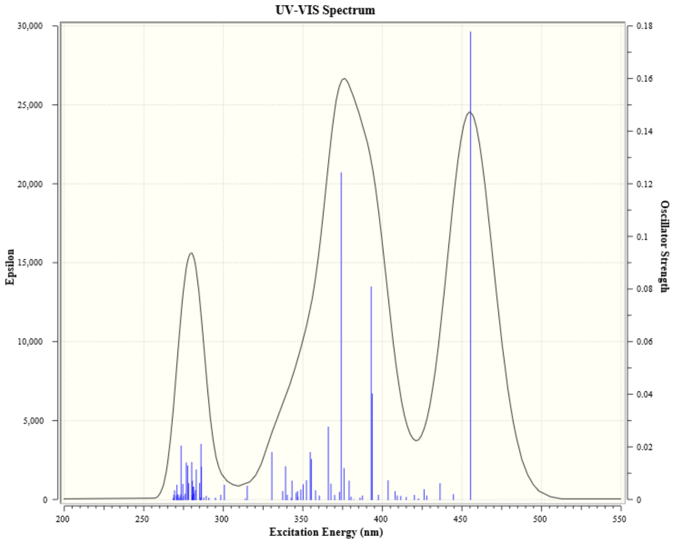

Figure S93. TD-DFT UV-Visible spectrum for eclipsed [(IPr*OMe)CuH]$_2$ in benzene.

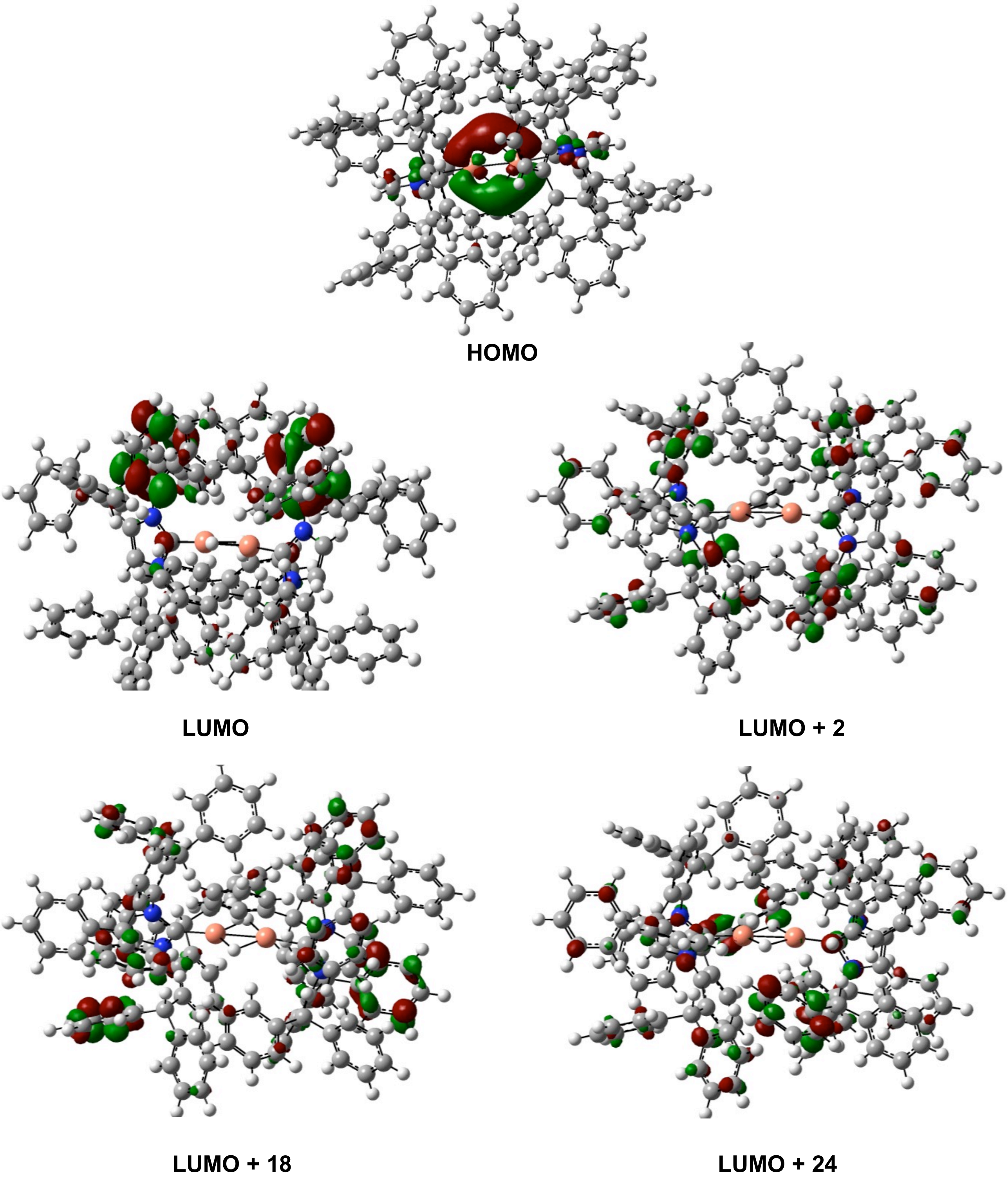

Figure S94. Orbitals relevant to TD-DFT predicted UV-Visible spectra for eclipsed $[(IPr^*H)CuH]_2$ in benzene.

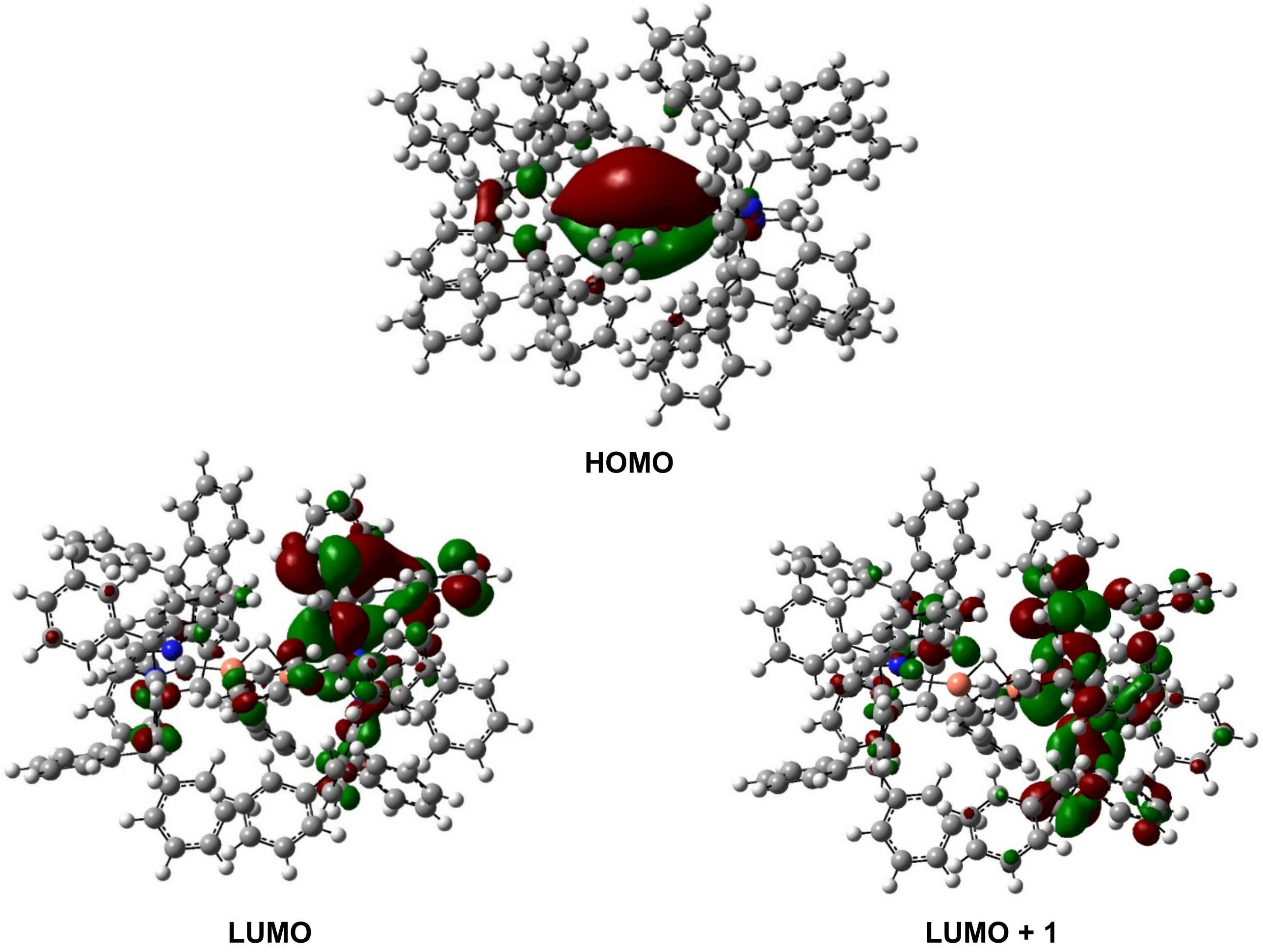

Figure S95. Orbitals relevant to TD-DFT predicted UV-Visible spectra for staggered [(IPr*H)CuH]$_2$ in benzene.

References

1 S. Okumura, S. Tang, T. Saito, K. Semba, S. Sakaki and Y. Nakao, *J. Am. Chem. Soc.*, 2016, **138**, 14699-14704.

2 G. Berthon-Gelloz, M. A. Siegler, A. L. Spek, B. Tinant, J. N. H. Reek and I. E. Markó, *Dalton Trans.*, 2010, **39**, 1444-1446.

3 V. S. Saberov, D. A. Evans, N. I. Korotkikh, A. H. Cowley, T. M. Pekhtereva, A. F. Popov and O. P. Shvaika, *Dalton Trans.*, 2014, **43**, 18117-18122.

4 S. Meiries, K. Speck, D. B. Cordes, A. M. Z. Slawin and S. P. Nolan, *Organometallics*, 2013, **32**, 330-339.

5 C. A. Citadelle, E. Le Nouy, F. Bisaro, A. M. Slawin and C. S. Cazin, *Dalton Trans.*, 2010, **39**, 4489-4491.

6 O. Santoro, A. Collado, A. M. Z. Slawin, S. P. Nolan and C. S. J. Cazin, *Chem. Commun.*, 2013, **49**, 10483-10485.

7 W.-J. Yoo, T. V. Q. Nguyen and S. Kobayashi, *Angew. Chem. Int. Ed.* , 2014, **53**, 10213-10217.

8 A. Gómez-Suárez, R. S. Ramón, O. Songis, A. M. Z. Slawin, C. S. J. Cazin and S. P. Nolan, *Organometallics*, 2011, **30**, 5463-5470.

9 F. Falivene, R. Credendino, A. Poater, A. Petta, L. Serra, R. Oliva, V. Scarano and L. Cavallo, *Organometallics*, 2016, **35**, 2286-2293.

10 A. Gomez-Suarez, D. J. Nelson and S. P. Nolan, *Chem. Commun.*, 2017, **53**, 2650-2660.

11 I. A. Guzei and M. Wendt, *Dalton Trans.*, 2006, 3991-3999.

12 B. R. Dible, R. E. Cowley and P. L. Holland, *Organometallics*, 2011, **30**, 5123-5132.

13 B. L. Tran, B. D. Neisen, A. L. Speelman, T. Gunasekara, E. S. Wiedner and R. M. Bullock, *Angew. Chem. Int. Ed.*, 2020, **132**, 8723-8731.

14 S. Bagherzadeh and N. P. Mankad, *Chem. Commun.*, 2018, **54**, 1097-1100.

15 J. S. Cha, *Org. Process Res. Dev.*, 2006, **10**, 1032-1053.

16 R. G. Parr and W. Yang, *Density-Functional Theory of Atoms and Molecules*, Oxford University Press, New York, 1989.

17 A. D. Becke, *J. Chem. Phys.*, 1993, **98**, 5648-5652.

18 C. Lee, W. Yang and R. G. Parr, *Phys. Rev. B.*, 1988, **37**, 785-789.

19 N. Godbout, D. R. Salahub, J. Andzelm and E. Wimmer, *Can. J. Chem.*, 1992, **70**, 560-571.

20 K. A. Peterson and C. Puzzarini, *Theor. Chem. Acc.*, 2005, **114**, 283-296.

21 Y. Zhao and D. G. Truhlar, *Theor. Chem. Acc.*, 2008, **120**, 214-241.

22 S. Grimme, *J. Comput. Chem.*, 2006, **27**, 1787-1799.

23 J. D. Chai and M. Head-Gordon, *Phys. Chem. Chem. Phys.*, 2008, **10**, 6615-6620.

24 H. Hu, M. Vasiliu, T. H. Stein, F. Qu, D. L. Gerlach, D. A. Dixon and K. H. Shaughnessy, *Inorg. Chem.*, 2019, **58**, 13299-13313.

25 Gaussian 16, Revision A.03, M. J. Frisch, G. W. Trucks, H. B. Schlegel, G. E. Scuseria, M. A. Robb, J. R. Cheeseman, G. Scalmani, V. Barone, G. A. Petersson, H. Nakatsuji, X. Li, M. Caricato, A. V. Marenich, J. Bloino, B. G. Janesko, R. Gomperts, B. Mennucci, H. P. Hratchian, J. V. Ortiz, A. F. Izmaylov, J. L. Sonnenberg, D. Williams-Young, F. Ding, F. Lipparini, F. Egidi, J. Goings, B. Peng, A. Petrone, T. Henderson, D. Ranasinghe, V. G. Zakrzewski, J. Gao, N. Rega, G. Zheng, W. Liang, M. Hada, M. Ehara, K. Toyota, R. Fukuda, J. Hasegawa, M. Ishida, T. Nakajima, Y. Honda, O. Kitao, H. Nakai, T. Vreven, K. Throssell, J. A. Montgomery, Jr., J. E. Peralta, F. Ogliaro, M. J. Bearpark, J. J. Heyd, E. N. Brothers, K. N. Kudin, V. N. Staroverov, T. A. Keith, R. Kobayashi, J. Normand, K. Raghavachari, A. P. Rendell, J. C. Burant, S. S. Iyengar, J. Tomasi, M. Cossi, J. M. Millam, M. Klene, C. Adamo, R. Cammi, J. W. Ochterski, R. L. Martin, K. Morokuma, O. Farkas, J. B. Foresman, and D. J. Fox, Gaussian, Inc., Wallingford CT, 2016.

26 R. F. Ribeiro, A. V. Marenich, C. J. Cramer and D. G. Truhlar, *J. Phys. Chem. B*, 2011, **115**, 14556-14562.

27 J. Tomasi, B. Mennucci and R. Cammi, *Chem. Rev.*, 2005, **105**, 2999-3093.

28 A. Klamt, *Quantum Chemistry to Fluid Phase Thermodynamics and Drug Design*, Elsevier, Amsterdam, 2005.

29 A. Klamt and G. J. Schüürmann, *J. Chem. Soc., Perkin Trans. 2*, 1993, 799-805.

30 A. E. Reed, L. A. Curtiss and F. Weinhold, *Chem. Rev.*, 1998, **88**, 899-926.

31 F. Weinhold and C. R. Landis, *Valency and Bonding: A Natural Bond Orbital Donor-Acceptor Perspective*, University Press, Cambridge, U.K., 2005.

32 E. D. Glendening, J. K. Badenhoop, A. E. Reed, J. E. Carpenter, J. A. Bohmann, C. M. Morales, K. P., C. R. Landis and F. Weinhold, *Journal*, 2018.

33 E. D. Glendening, C. R. Landis and F. Weinhold, *J. Comput. Chem.*, 2019, **40**, 2234-2241.

34 R. Bauernschmitt and R. Ahlrichs, *Chem. Phys. Lett.*, 1996, **256**, 454-464.

35 M. E. Casida, C. Jamorski, K. C. Casida and D. R. Salahub, *J. Chem. Phys.*, 1998, **108**, 4439-4449.
